# Ligand-controlled regioselective and chemodivergent defluorinative functionalization of *gem*-difluorocyclopropanes with simple ketones†

**DOI:** 10.1039/d1sc05451a

**Published:** 2021-11-08

**Authors:** Leiyang Lv, Huijun Qian, Yangyang Ma, Shiqing Huang, Xiaoyu Yan, Zhiping Li

**Affiliations:** Department of Chemistry, Renmin University of China Beijing 100872 China lvleiyang2008@ruc.edu.cn zhipingli@ruc.edu.cn

## Abstract

Modulating the reaction selectivity is highly attractive and pivotal to the rational design of synthetic regimes. The defluorinative functionalization of *gem*-difluorocyclopropanes constitutes a promising route to construct β-vinyl fluorine scaffolds, whereas chemo- and regioselective access to α-substitution patterns remains a formidable challenge. Presented herein is a robust Pd/NHC ligand synergistic strategy that could enable the C–F bond functionalization with exclusive α-regioselectivity with simple ketones. The key design adopted enolates as π-conjugated ambident nucleophiles that undergo inner-sphere 3,3′-reductive elimination warranted by the sterically hindered-yet-flexible Pd-PEPPSI complex. The excellent branched mono-defluorinative alkylation was achieved with a sterically highly demanding IHept ligand, while subtly less bulky SIPr acted as a bifunctional ligand that not only facilitated α-selective C(sp^3^)–F cleavage, but also rendered the newly-formed C(sp^2^)–F bond as the linchpin for subsequent C–O bond formation. These examples represented an unprecedented ligand-controlled regioselective and chemodivergent approach to various mono-fluorinated terminal alkenes and/or furans from the same readily available starting materials.

## Abstract 2

A robust Pd/NHC ligand synergistic strategy that enables the exquisite regioselective and chemodivergent C–F bond functionalization of *gem*-difluorocyclopropanes with simple ketones, is reported.

## Graphical abstract

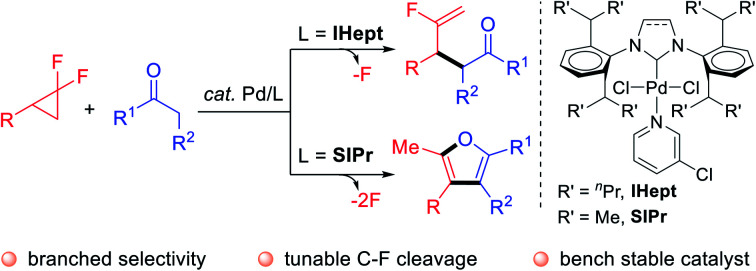

## Introduction

**Scheme 1:**
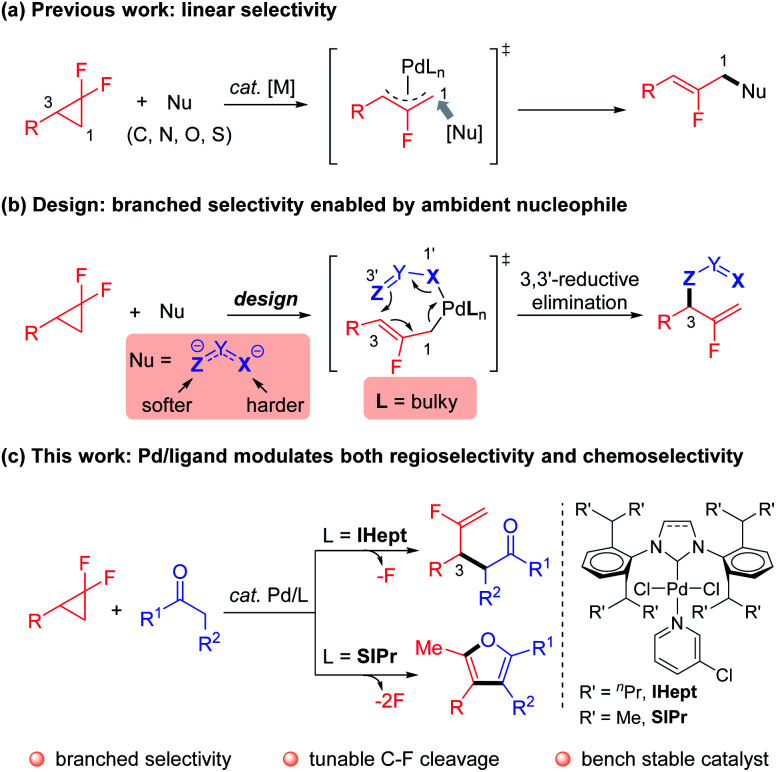
Strategies for the regio-and chemoselective C–F bond functionalization of *gem*-difluorocyclopropanes.

Controlling reaction selectivity to outcompete the intrinsic bias and innate reactivity, to achieve a specific bond-forming mode is a formidable challenge.^1^ Nature exquisitely exploits enzymes as powerful catalysts to achieve incredibly selective transformations.^2^ Inspired by this phenomenon, synthetic chemists have devoted great effort to developing reliable and efficient approaches for the creative manipulation of inherent reactivity and selectivity.^3^ In this context, transition-metal catalyzed regio- and chemoselective functionalization of inert C–F bonds is a synthetically useful yet challenging research topic.^4,5^ For example, Pd-catalyzed ring-opening functionalization of *gem*-difluorocyclopropanes,^6^ which acts as a novel and reliable fluorine-containing building block, predominantly proceeds through cleavage of a C–F bond to afford an intrinsically stable β-monofluorinated alkene structure, with linear selectivity (Scheme 1a).^7–9^ Despite the flourishing advances, the deconstructive transformation of *gem*-difluorocyclopropanes that incorporate functionalities into the sterically hindered internal position, delivering kinetically favored α-monofluorinated alkenes with branched selectivity, remains a challenging task. In addition, α-monofluorinated alkenes are attractive structures, which mimic amides and enols in drug discovery and medicinal chemistry,^10^ particularly with the presence of synthetically versatile fluorinated terminal C=C bonds.^11^ Therefore, an alternative strategy that allows for the incorporation of the functional group into the *gem*-difluorocyclopropanes with complementary regioselectivity and an innovative reaction manifold is highly desirable. Notably, the highly regioselective ring-opening cross-couplings of relevant aziridines have been elegantly realized by Doyle^12^ and Takeda.^13^

Motivated by the elegant accomplishments of Hou,^14^ Echavarren^15^ and Morken^16^*et al.* on allyl–allyl couplings, we aimed to take advantage of the flexible coordination character of ambident nucleophiles coupled with Pd/NHC ligand cooperative catalysis. According to the hard–soft-acid–base (HSAB) principle,^17^ heteroatoms (*e.g.* nitrogen and oxygen) in such nucleophiles are harder Lewis bases that preferentially coordinate to the metal center, thus ensuring the softer carbon attack at the sterically more hindered internal position *via* inner-sphere 3,3′-reductive elimination warranted by the congested NHC ligand (Scheme 1b). Besides, the strong binding affinity of carbene to the metal center contributes to the thermal stability of the Pd–NHC complex, which renders a longer catalyst lifetime and consistent reactivity.^18^ Guided by this concept, we have recently reported that simple hydrazones could act as ambident nucleophiles to realize the anticipated branched regioselectivity in the Pd-catalyzed mono-defluorinative alkylation reactions facilitated by denitrogenation.^19^

Ketones are a cheap and naturally abundant feedstock that have been widely used in both academia and industry.^20^ Inspired by the inner-sphere allylic alkylations, and given the ketone–enolate tautomerization, we envisioned that if enolates could serve as π-conjugated ambident nucleophiles (*e.g.* oxygen as the harder coordination anchor, carbon as the softer coupling site) to couple with *gem*-difluorocyclopropanes under Pd/NHC catalysis. The advantage of the incorporated carbonyl functionality may allow for further interconversions. However, compared with stabilized carbon nucleophiles (*e.g.* malonates or β-ketoesters), destabilized simple ketone enolates, especially methyl ketones [p*K*_a_ ≈ 25 (DMSO)],^21^ are challenging coupling partners in such transformations^22^ due to: (1) direct outer-sphere attack *vs.* inner-sphere coordination to afford competitive linear products; (2) overalkylation of the ketone commonly occurring due to the mono-alkylated ketones featuring more acid C–H bonds; (3) aldol-type condensations prevailing in the case of destabilized enolates. Herein, our strategy to circumvent these challenges takes advantage of the cooperative Pd catalysis with tunable, sterically hindered yet flexible NHC ligands. The excellent branched regioselective mono-defluorinative alkylation was achieved with the bulky IHept ligand, while sterically less hindered SIPr acted as an unexpected bifunctional ligand,^23^ that not only enabled the exquisitely branched selective C(sp^3^)–F cleavage, but also facilitated further manipulation of the newly-formed C(sp^2^)–F bond. This powerful ligand-controlled regio- and chemoselective strategy afforded a variety of mono-fluorinated terminal alkenes or advantageous furans from the same readily available *gem*-difluorocyclopropanes and ketones.

## Results and discussion

As a proof of concept, we began our investigations with the reaction of *gem*-difluorocyclopropane 1a as a limiting reagent in the presence of 2.0 equiv. of acetophenone 2a, base, and 5 mol% of bench-stable Pd-PEPPSI catalyst at 100 °C for 1 h (Table 1). Among the various Pd 1–6 complexes examined, the desired branched product 3a and unexpected furan product 4a were obtained, in which the yields and proportions were sensitive to the steric hindrance of the side chain of the NHC ligands. Notably, the thermodynamically stable linear product 5 was detected in only trace amounts (<1%) in all cases. The *gem*-difluorocyclopropane 1a was recovered quantitatively when Pd-1 was tested (entry 1). In the case of bulkier Pd-2 and Pd-3, furan 4a was obtained selectively in calc. ∼30% yields *via* twofold defluorinative functionalization (entries 2 and 3). With further increase of the ligand steric hindrance (*e.g.*Pd-4 to Pd-6), an intriguing complete shift of the chemoselectivity was observed, and the branched mono-defluorination product 3a was afforded predominantly (entries 4–6). The best result was for the branched fluoroalkene product 3a which was selectively obtained in 95% yield (3a : 4a = 32 : 1) when Pd-6 was used in the presence of NaOH (entry 6). The effect of various bases was then examined for the defluorinative functionalization reaction. For example, 3a was obtained in a slightly decreased yield (84%) yet excellent chemoselectivity (28 : 1) when LiO*^t^*Bu was tested (entry 7), while KOH was inferior in terms of both reactivity and selectivity (entry 8).

**Table 1:** Optimization of the reaction conditions (N.D. = not detected)^a^

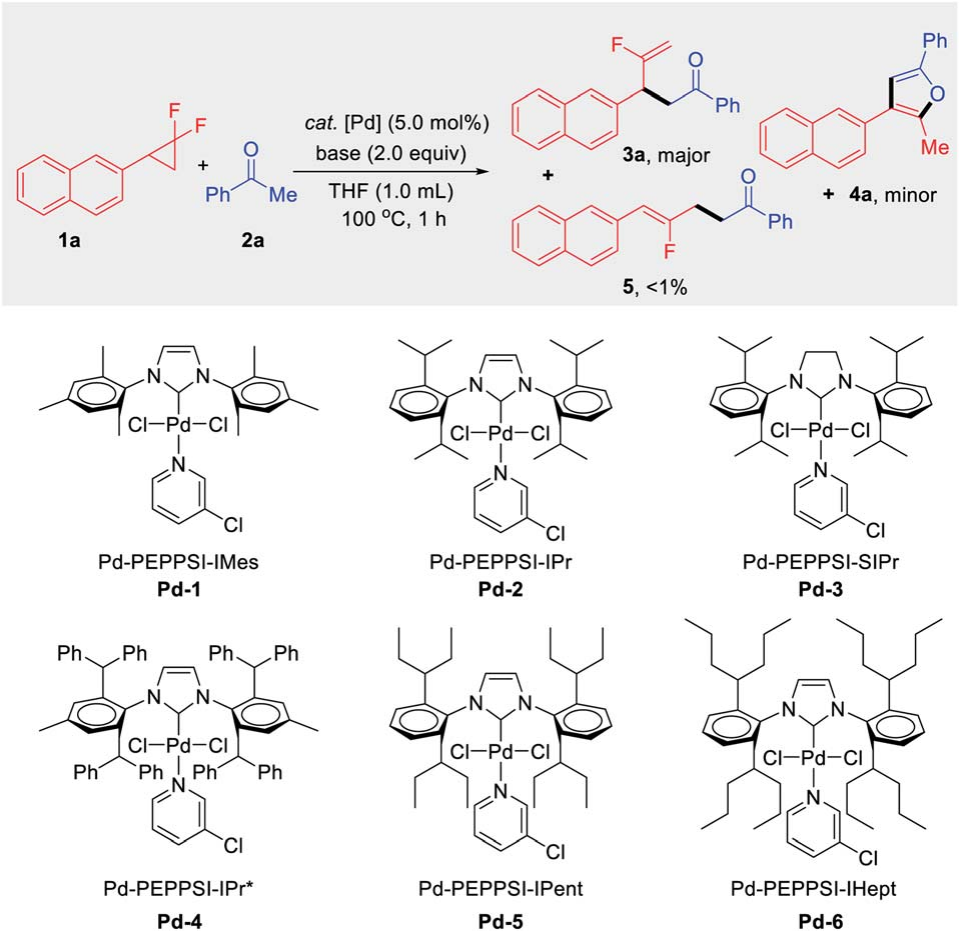					
Entry	Cat. [Pd]	Base	3a^b^ (%)	4a^b^ (%)	3a/4a^c^
1	Pd-1	NaOH	N.D.	N.D.	—
2	Pd-2	NaOH	<1	32	<1 : 32
3	Pd-3	NaOH	<1	31	<1 : 31
4	Pd-4	NaOH	65	6	11 : 1
5	Pd-5	NaOH	82	7	12 : 1
6	Pd-6	**NaOH**	**95**	**3**	**32** **:** **1**
7	Pd-6	LiO^*t*^Bu	84	3	28 : 1
8	Pd-6	KOH	65	17	3.8 : 1
9	Pd-6	Cs_2_CO_3_	34	1	34 : 1
10^d^	Pd-6	Cs_2_CO_3_	91	3	30 : 1
11	Pd-6	K_3_PO_4_	N.D.	N.D.	—
12	Pd-6	K_2_CO_3_	N.D.	N.D.	—
13	Pd-6	—	N.D.	N.D.	—
14	—	NaOH	N.D.	N.D.	—
15^e^	Pd-6	NaOH	93	3	30 : 1

a Reaction conditions: 1a (0.1 mmol), 2a (0.2 mmol), Pd-PEPPSI catalyst (5.0 mol%), base (0.2 mmol), THF (1.0 mL), 100 °C, 1 h under N_2_ unless otherwise noted.

b NMR yields were based on 1a and determined by ^1^H NMR using CH_2_Br_2_ as an internal standard.

c The 3a/4a ratio was determined by ^1^H NMR analysis of the crude mixtures.

d 6 h.

e 1a (1.0 mmol), 2a (2.0 mmol), Pd-6 (2.5 mol%), NaOH (1.5 mmol), THF (6.0 mL).

When the transformation was carried out in the presence of relatively weak Cs_2_CO_3_ for 1 h, 3a was afforded in 34% yield with 55% of starting material remaining. Gratifyingly, the excellent result (91% yield, 3a/4a = 30 : 1) was obtained when extending the reaction time to 6 h (entry 10). In the case of weak bases such as K_2_CO_3_ and K_3_PO_4_, no desired product was detected along with the quantitative recovered 1a (entries 11 and 12). Control experiments confirmed that no product 3a, 4a or 5 were detected in the absence of either base or palladium catalyst (entries 13 and 14). Besides, the catalytic transformation was amenable to 1.0 mmol scale synthesis with a lower catalyst loading (5.0 mol% to 2.5 mol% of Pd-6), affording 3a in 93% yield without noticeable diminution in regio- and chemoselectivity (entry 15).

With the optimal conditions identified, we sought to explore the scope of palladium-catalyzed branched selective mono-defluorinative alkylations. As shown in Table 2 (A) aromatic methyl ketones assembled with a variety of functional groups, including both the electron-donating (–Me, –Ph, –OMe, –NMe_2_) and electron-withdrawing (–F, –CF_3_, –CN) groups, proceeded efficiently to deliver 3b–i in 65–92% yields with excellent regioselectivity (>100 : 1) and chemoselectivity (>20 : 1). An increase of the *ortho* steric congestion adjacent to the carbonyl of the aryl ring, reduced the reactivity (3j, 66%). The desired product 3k was obtained in 89% yield when 2-acetyl naphthalene was tested. Extension to substrates bearing cyclic ether functionalities 3l and 3m also proved to be successful under the optimized conditions. In the case of base-sensitive functional groups, such as esters and amides, Cs_2_CO_3_ was used instead of NaOH to ensure good reaction efficiency (3n and 3o). Given the prevalence of heterocycles in medicinal chemistry, different heterocyclic ketone substrates were examined accordingly. To our delight, different heterocycles such as furan (3p), thiophene (3q), *N*-methyl pyrrole (3r), *N*-methyl indole (3s) and even pyridine (3t) were all tolerated without deactivation of the Pd catalyst by coordinating heteroatoms, affording the desired products in 74–96% yield. Other aromatic ketones such as propiophenone and 1-indanone also worked well and provided the desired products 3u and 3v in excellent yields with 1.6 : 1 and 2 : 1 diastereomeric ratios, respectively. It should be noteworthy that this protocol not only renders the challenging transformation being carried out with aromatic ketones, but also allows the efficient reaction activity with relatively inert aliphatic ketones. For example, methyl *tert*-butyl ketone performed well with 1a and gave the corresponding product 3w in 80% yield. In the case of non-symmetric alkyl ketones with two reactive sites such as methyl iso-butyl ketone and methyl cyclohexyl ketone, the coupling reactions occurred preferentially at the sterically less hindered CH_3_ instead of CH, affording the major products 3x and 3y in 61% and 79% yields, respectively. In addition, it is well-known that mono-alkylation of simple and readily available acetone is challenging due to: (1) the much less acidic C–H bond compared with other carbonyl compounds; (2) increased susceptibility to undertake overalkylations; (3) the large excess loading and/or as solvent.^24^ However, we were delighted to find that our developed Pd/NHC catalytic protocol could well address these challenges, affording the corresponding mono-alkylation product 3z in 75% yield, which showcased the great potential of this method in the selective functionalization of destabilized carbon nucleophiles.

**Table 2:** Substrate scope of mono-defluorinative alkylations^a^^,^^b^

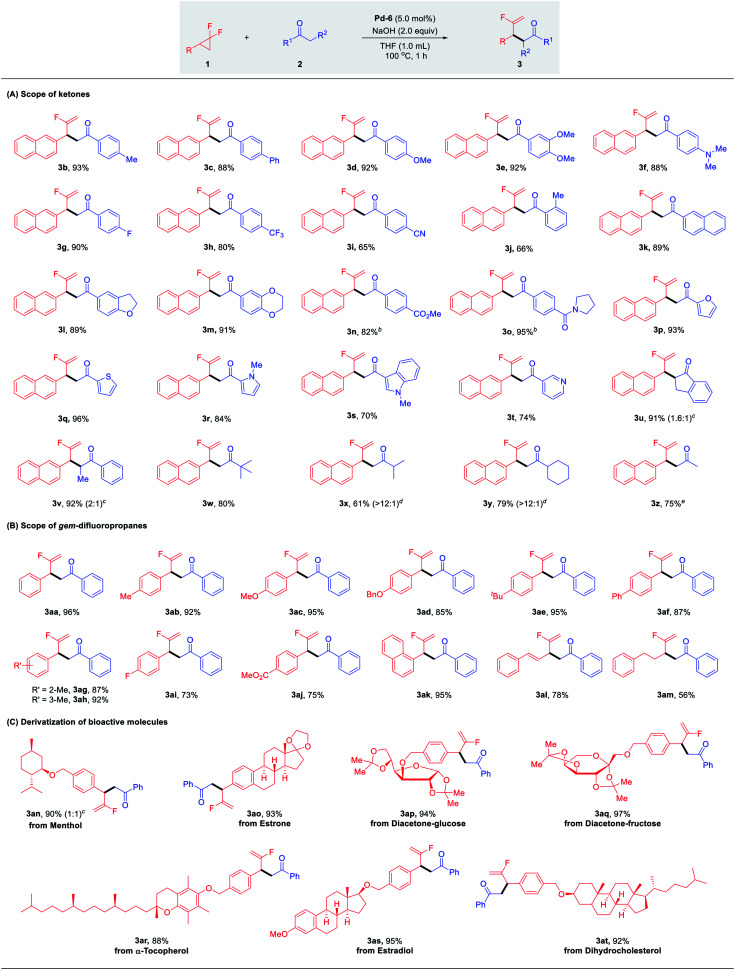

a Reaction conditions: 1 (0.1 mmol), 2 (0.2 mmol), Pd-PEPPSI-Hept (5.0 mol%), NaOH (0.2 mmol), THF (1.0 mL), 100 °C, 1 h under N_2_, isolated yields.

b Cs_2_CO_3_ used instead of NaOH.

c The diastereomeric ratio was determined by ^1^H NMR analysis of the crude mixtures.

d Coupling at CH_3_/CH ratio.

e Acetone (0.5 mmol).

Next, we turned our attention to the substrate spectrum with respect to *gem*-difluorinated cyclopropanes (Table 2, B). The aromatic substrates bearing substituents with versatile electronic and steric properties, were highly involved in this transformation. For example, substrates bearing electron-donating (–Me, –OMe, –OBn, –^*t*^Bu, –Ph) groups underwent the reaction smoothly, delivering the corresponding α-fluorinated alkenes 3aa–3af in good to excellent yields (85–96%). The reactivity was not hampered by the *ortho* or *meta* substitution on the aryl ring (3ag, 3ah). Electron-withdrawing substituents (–F, CO_2_Me) were also compatible, affording the desired branched products 3ai and 3aj in good yields. Furthermore, 1-(2,2-difluorocyclopropyl)naphthalene was readily coupled with acetophenone under the optimized conditions to give 3ak in 95% yield. Gratifyingly, the desired products 3al and 3am were also attained efficiently in the case of alkyl- and alkenyl-substituted *gem*-difluorocyclopropanes.

This robust protocol enables facile manipulation of derivatives of natural products and drug molecules with structural diversity. As shown in Table 2, C, various natural product derivatives, such as menthol (3an), estrone (3ao), diacetone-glucose (3ap), diacetone-fructose (3aq), α-tocopherol (3ar), estradiol (3as) and dihydrocholesterol (3at) could be smoothly assembled with carbonyl and terminal fluorinated olefin functional groups in excellent yields (88–97%). These efficient and expedient modifications of bioactive molecules shows the potential of this method for the preparation of fluorine-containing candidates in drug discovery.

As outlined in Table 1, sterically less hindered catalysts Pd-2 (IPr) and Pd-3 (SIPr) induced completely chemodivergent reactivity to yield the unexpected furan products, which encouraged us to conduct a critical survey of the reaction conditions for these twofold defluorinative transformations (see Fig. S1 in ESI† for details). The best conditions were obtained using PEPPSI-SIPr (5.0 mol%), KOH (2.0 equiv.) and ketone (2.0 equiv.) in THF at 100 °C for 12 h, providing 4a in 73% yield. The generality of this transformation was assessed next (Table 3, A). An array of acetophenone derivatives possessing either electron-donating or electron-withdrawing groups, no matter the *para*-, *meta*- or *ortho*-substitution patterns on the aromatic ring, were all suitable substrates to afford the furan products 4a–4j in 63–72% yields, with slightly decreased yields observed in the case of electron-withdrawing substituents (4f, 4g, 4j). The functional groups, such as alkyne (4k), pyrrolidine (4l) and *N*,*N*-dimethyl (4m) were well tolerated. Reactions were also compatible with 2,3-dihydrobenzofuran (4n) and 2,3-dihydrobenzo[*b*][1,4]dioxin (4o) derived ketones. π-Extended acetophenone derivatives such as naphthalene and fluorene also reacted smoothly with 1a to deliver furan products 4p and 4q in 63% and 65% yields, respectively. Notably, propiophenone could undergo the transformation as well, affording the desired fully-substituted furan 4r in 44% yield.

**Table 3:** The substrate scope of two fold defluorinative functionalizations^a^^,^^b^

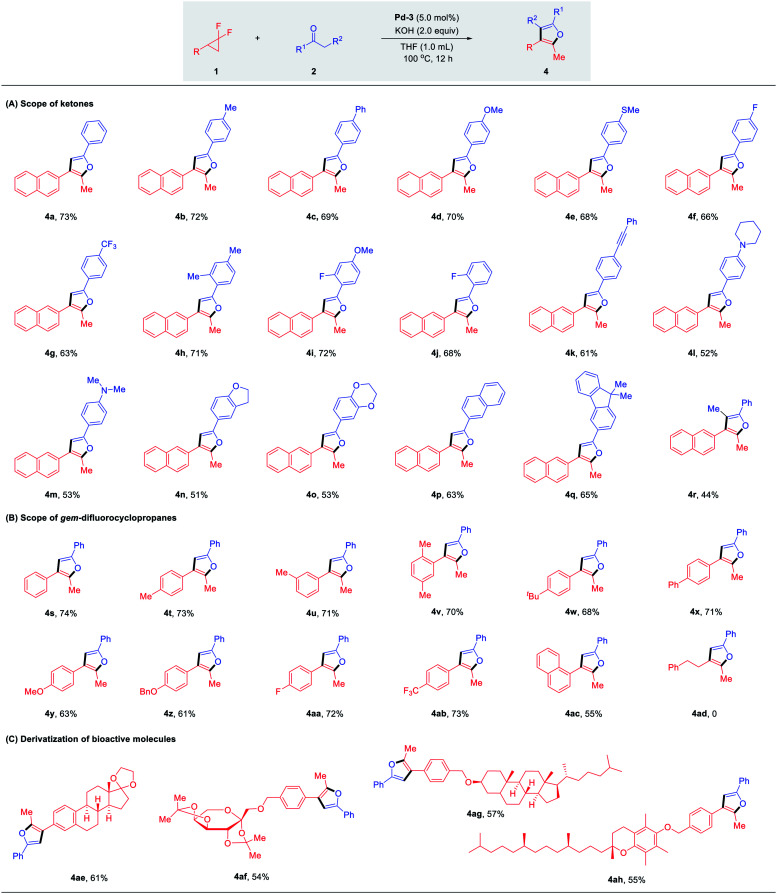

a Reaction conditions: 1 (0.1 mmol), 2 (0.2 mmol), Pd-PEPPSI-SIPr (5.0 mol%), KOH (0.2 mmol), THF (1.0 mL), 100 °C, 12 h under N_2_.

b Isolated yields.

The compatibility of *gem*-difluoro cyclopropanes in this transformation was examined accordingly. As shown in Table 3, B, the substitution patterns of the substrates proved to be versatile. Good yields were obtained ranging from 61–74% in the case of the electron-donating (–Me, –^*t*^Bu, –Ph, –OMe, –OBn) and electron-withdrawing groups (–F, –CF_3_) tested. 1-Naphthalene substituted *gem*-difluorocyclopropane was also identified as a suitable reaction partner, affording the desired product 4ac in 55% yield. Moreover, *gem*-difluoro cyclopropanes derived from bioactive molecules – estrone, diacetone-fructose, dihydrocholesterol and α-tocopherol – were successfully converted to the corresponding furan products 4ae–ah in moderate yields (Table 3, C). These examples demonstrated that by employing different ligands or bases, diverse products could be implemented selectively from the identical starting materials under similar reaction conditions.

**Scheme 2:**
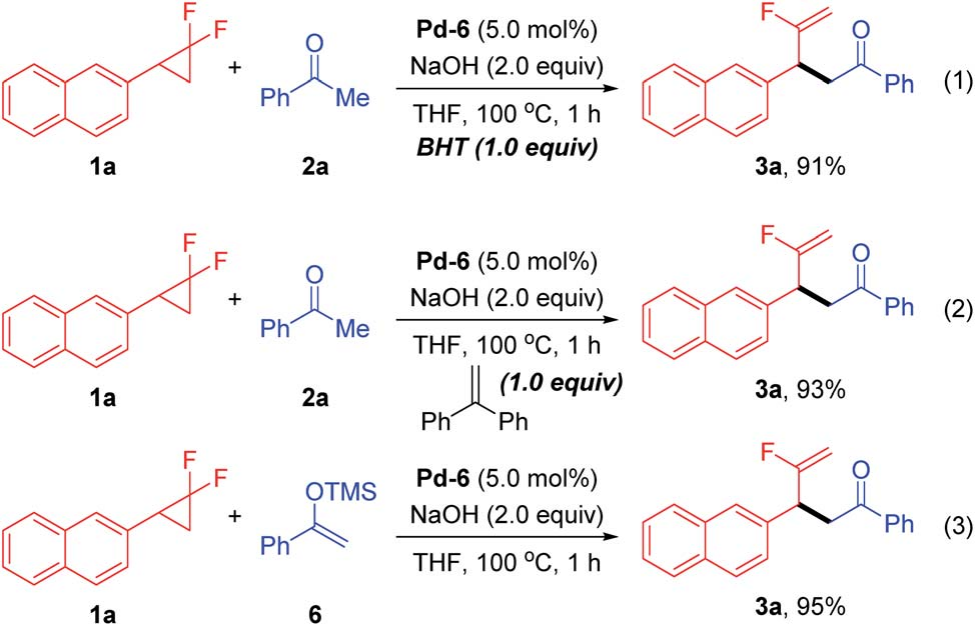
Control experiments.

To probe the preliminary insight into the reaction mechanism, several control experiments were carried out (Scheme 2). The yield of 3a was not affected when radical inhibitors such as butylated hydroxytoluene (BHT) or 1,1-diphenylethylene were added under the standard conditions, which ruled out the involvement of a radical pathway (eqn (1) and (2)). Besides, silyl enol ether 6 also reacted with 1a to afford the desired product 3a in comparable yield (eqn (3)). Moreover, a time-course reaction indicated that there was a short induction period related to activation of the Pd precatalyst (calc. 10 min), after which the transformation was faster and could be completed in 1 h (see ESI† for details).

**Scheme 3:**
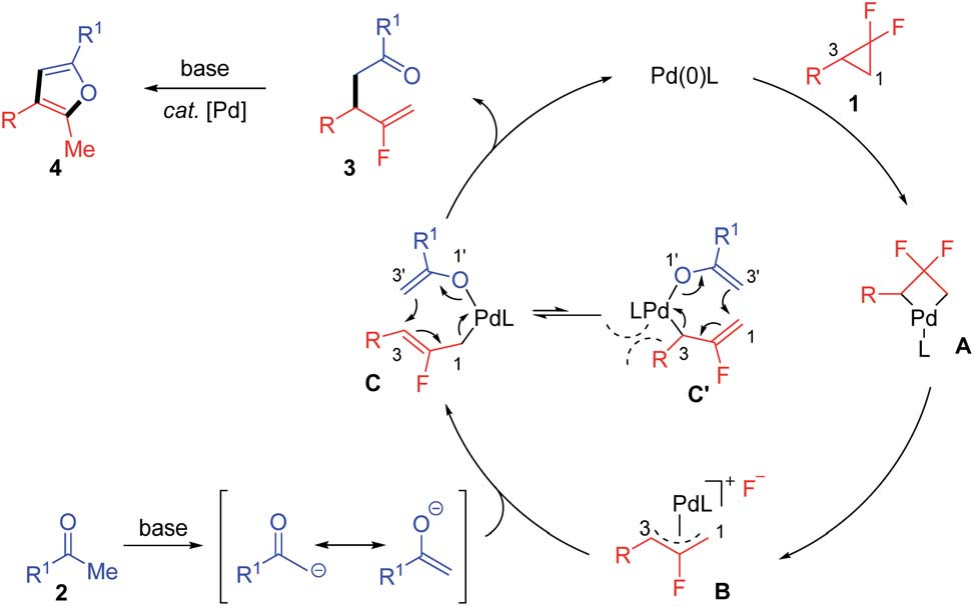
Proposed reaction mechanism.

Taking the above results and literature reports together, a plausible reaction mechanism was proposed to illustrate the regioselectivity and chemodivergent reactivity of this transformation (Scheme 3). Initially, the Pd(0) catalyst reacts with *gem*-difluorocyclopropane *via* C–C bond activation and C–F bond cleavage to generate the allyl-Pd(ii) complex B. The two fluorine atoms on the cyclopropane ring make the C1–C2–C3 bond angle increase and distal (C1–C3) bond lengthen, thus leading to the distal bond in the *gem*-difluorocyclopropane more easily cleaving during the ring-opening process.^26^ This highly selective ring-opening mode was also calculated by Fu and coworkers with the lower energy barrier of the oxidative addition of the C1–C3 bond (7.9 kcal mol^−1^) *versus* oxidation of the C2–F bond (46.5 kcal mol^−1^).^7^ Also, considering alkyl substrates such as 1m are also active in the mono-defluorinative alkylation, we suspected that it was less possible for the current ring-opening of *gem*-difluorocyclopropane in the SN_2_ fashion, where the interactions existed between the aryl unit of the aziridines and Pd catalyst.^13*b*^ Then, the transmetalation of B with enolate as the π-ambident nucleophile derived from deprotonated ketone 2, affords the crucial bis(η^1^-allyl) intermediate C, which undergoes inner-sphere 3,3′-reductive elimination,^25^ guaranteed by the sterically encumbered NHC ligand to deliver the branched product 3 and regenerate the Pd(0) catalyst. The furan product 4 was formed *via* the base-mediated enolization/nucleophilic substitution/rearomatization sequence promoted by the less-congested Pd-3 catalyst. Therefore, the elaborate design of ligand structure and modification of reaction conditions (*e.g.* base, reaction time) could enable the exquisitely chemodivergent synthesis of β-monofluorinated alkenes and/or corresponding furan products *via* selective C–F bond cleavage.

## Conclusion

In summary, we have developed a powerful ligand-controlled regioselective and chemodivergent defluorinative functionalization of *gem*-difluorocyclopropanes to deliver mono-fluorinated terminal alkenes and/or furans. This potent Pd/NHC ligand synergistic strategy enabled the C–F bond functionalization in an exclusive α-regioselectivity with simple ketones as π-conjugated ambident nucleophiles, which was difficult to access by conventional approaches. The excellent branched regioselective mono-defluorinative alkylations were achieved with sterically highly demanding IHept ligands, while less bulky SIPr acted as an unexpected bifunctional ligand that not only enabled the exquisitely branched selective C(sp^3^)–F cleavage, but also facilitated further manipulation of the newly-formed C(sp^2^)–F bond. The robustness of this protocol was demonstrated by the wide substrate scope, excellent regio- and chemoselectivity, good functional group compatibility, efficient modification of bioactive molecules and natural products as well as stable and user-friendly precatalysts. Further efforts will be made to develop the enantioselective version. Our study is dedicated to the art of tuning ligands to achieve selective catalysis by utilizing identical starting materials to produce different products, and also enriches the toolbox of chemists enabling cross-couplings with destabilized carbon nucleophiles.

## Data availability

All experimental data in this manuscript are available in the ESI.†

## Author contributions

L. L. conceived the idea for this work, supervised and designed the experiments. L. Z. co-supervised the project. L. L. and Q. H. performed the experiments and analyzed the data. Ma, Y. synthesized some of the catalysts. L. L. wrote the manuscript. All authors discussed the experimental results and commented on the manuscript.

## Conflicts of interest

There are no conflicts to declare.

## Supplementary Material

SC-012-D1SC05451A-s001
