# Supplementary material for: Ligand-controlled regioselective and chemodivergent defluorinative functionalization of gem-difluorocyclopropanes with simple ketones
Source: Chem Sci. 2021 Nov 8;12(47):15511–8. doi: 10.1039/d1sc05451a (PMC8654029; doi:10.1039/d1sc05451a)

Electronic Supplementary Material (ESI) for Chemical Science.
This journal is © The Royal Society of Chemistry 2021

Supporting Information

Ligand-Controlled Regioselective and Chemodivergent Defluorinative Functionalization of *gem*-Difluorocyclopropanes with Simple Ketones

Leiyang Lv,*[a] Huijun Qian,[a] Yangyang Ma,[a] Shiqing Huang,[a] Xiaoyu Yan[a] and Zhiping Li*[a]

Contents

1)	General information	S2
2)	Synthesis of *gem*-difluorocyclopropane **1**	S2
3)	General procedure and characterization data for product **3**	S5
4)	Scale up synthesis of product **3a**	S24
5)	Optimization of the reaction conditions for furan product	S25
6)	General procedure and characterization data for product **4**	S26
7)	Control experiments	S39
8)	Time-course reaction	S39
9)	References	S40
10)	Copies of ^{1}H NMR, ^{13}C NMR and ^{19}F NMR spectra for new compounds	S41

1. General information

^{1}H NMR spectra were recorded on Bruker 400 or 600 MHz spectrometer and the chemical shifts were reported in parts per million (*δ*) relative to internal standard TMS (0 ppm) for $CDCl_3$. The peak patterns are indicated as follows: s, singlet; d, doublet; dd, doublet of doublet; t, triplet; q, quartet; m, multiplet. The coupling constants, *J*, are reported in Hertz (Hz). ^{13}C NMR spectra were obtained at Bruker 100 or 150 MHz and referenced to the internal solvent signals. ^{19}F NMR spectra were obtained at Bruker 376 or 565 MHz. $CDCl_3$ was used as the NMR solvent. High-resolution mass spectra (HRMS) were acquired on Thermo Q-Exactive instrument (quadrupole mass analyzer) using electrospray ionization mode (ESI). Flash column chromatography was performed over silica gel 200-300. All other reagents were purchased from Alfa, Aldrich, TCI, Energy, Innochem and Sinocompound Catalysts used without further purification. The Pd-PEPPSI catalyst were synthesized according to the reported literature.[1]

2. Synthesis of *gem*-difluorocyclopropane 1

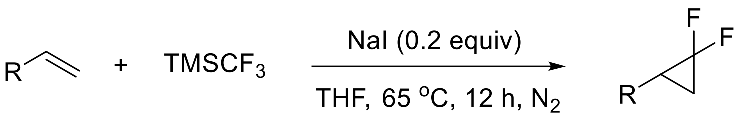

The *gem*-difluorocyclopropanes were prepared according to the known procedure.[2] A flame-dried reaction tube (50 cm^3) equipped with a magnetic stir bar was charged with anhydrous NaI (0.2 equiv), dry THF (10.0 mL), $TMSCF_3$ (2.5 equiv) and corresponding alkenes (4.0 mmol) under nitrogen atmosphere. The reaction tube was sealed with Teflon septum and stirred at 65 °C for 12 h. After the mixture was cooled to room temperature, the resulting solution was directly filtered through a pad of celite, and then evaporated to dryness under reduce pressure. The crude mixture was extracted with ethyl acetate (20.0 mL), washed with saturated $Na_2S_2O_3$ (10.0 mL) and brine (10.0 mL). The organic layer was dried over Na_2SO_4, filtered and concentrated, the residue was purified by column chromatography on silica gel to give the pure corresponding *gem*-difluorocyclopropanes. Characterization of new *gem*-difluorocyclopropanes **1n**, **1p-1t** are listed below.

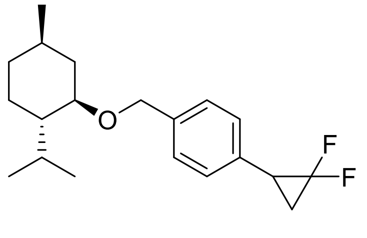

1-(2,2-Difluorocyclopropyl)-4-((((1R,2S,5R)-2-isopropyl-5-methylcyclohexyl)oxy)methyl)benzene (1n).

(petroleum ether: ethyl acetate = 50:1, R_f = 0.4); ^{1}H NMR (600 MHz, $CDCl_3$) δ 7.31 (d, J = 8.0 Hz, 2H), 7.19 (d, J = 8.0 Hz, 2H), 4.64 (d, J = 11.4 Hz, 1H), 4.37 (d, J = 11.4 Hz, 1H), 3.16 (dt, J = 10.6, 4.0 Hz, 1H), 2.77-2.71 (m, 1H), 2.30-2.26 (m, 1H), 2.20-2.16 (m, 1H), 1.83-1.77 (m, 1H), 1.67-1.58 (m, 3H), 1.39-1.32 (m, 1H), 1.31-1.25 (m, 1H), 0.98-0.82 (m, 9H), 0.70 (dd, J = 7.0, 1.8 Hz, 3H); ^{13}C NMR (150 MHz, $CDCl_3$) δ 138.1, 132.7, 128.0, 127.9, 112.6 (dd, $J_{C\text{-}F}$ = 284.4, 284.2 Hz), 78.8, 70.0, 48.3, 40.3, 34.5, 31.6, 27.0 (t, $J_{C\text{-}F}$ = 11.4 Hz), 25.5, 23.2, 22.4, 21.0, 17.0 (d, $J_{C\text{-}F}$ = 11.4 Hz), 16.0; ^{19}F NMR (565 MHz, $CDCl_3$) δ -125.9 (dtd, J = 153.9, 12.8, 3.9 Hz, 1F), -142.3 (ddd, J = 153.9, 12.8, 4.8 Hz, 1F). HRMS (APCI) calcd for $C_{20}H_{29}F_2O$ [M + H $^+$], 323.2181; found: 323.2178.

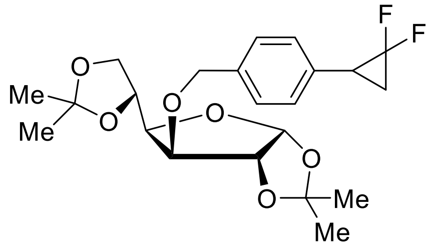

(3aR,5R)-6-((4-(2,2-Difluorocyclopropyl)benzyl)oxy)-5-((R)-2,2-dimethyl-1,3-dioxolan-4-yl)-2,2-dimethyltetrahydrofuro[2,3-d][1,3]dioxole (1p). (petroleum ether: ethyl acetate = 4:1, R_f = 0.6); ^{1}H NMR (600 MHz, $CDCl_3$) δ 7.31 (d, J = 8.0 Hz, 2H), 7.20 (d, J = 8.0 Hz, 2H), 5.89 (d, J = 3.6 Hz, 1H), 4.67 (d, J = 11.8 Hz, 1H), 4.62 (d, J = 11.8 Hz, 1H), 4.58 (d, J = 3.6 Hz, 1H), 4.37-4.34 (m, 1H), 4.15-4.10 (m, 2H), 4.01-3.99 (m, 2H), 2.77-2.72 (m, 1H), 1.85-1.79 (m, 1H), 1.65-1.59 (m, 1H), 1.49 (s, 3H), 1.43 (s, 3H), 1.37 (s, 3H), 1.31 (s, 3H); ^{13}C NMR (150 MHz, $CDCl_3$) δ 136.6, 133.3, 128.1, 127.8, 112.5 (dd, $J_{C\text{-}F}$ = 284.4, 284.2 Hz), 117.8, 109.0, 105.3, 82.6, 81.7, 81.3, 72.4, 72.0, 67.4, 26.9 (t, $J_{C\text{-}F}$ = 11.4 Hz), 26.8, 26.7, 26.2, 25.4, 17.0 (t, $J_{C\text{-}F}$ = 11.4 Hz); ^{19}F NMR (565 MHz, $CDCl_3$) δ -125.9 (dtd, J = 154.7, 12.6, 3.9 Hz, 1F), -142.3 (ddd, J = 154.7, 12.6, 5.0 Hz, 1F). HRMS (ESI) calcd for $C_{22}H_{28}F_2NaO_6$ [M + Na $^+$], 449.1746; found: 449.1745.

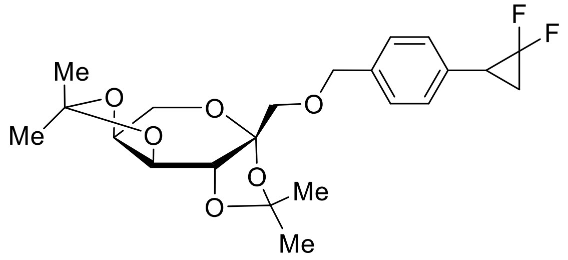

(3aR,5aS,8aS,8bR)-3a-(((4-(2,2-Difluorocyclopropyl)benzyl)oxy)methyl)-2,2,7,7-tetramethyltetrahydro-5H-bis([1,3]dioxolo)[4,5-b:4',5'-d]pyran (1q). (petroleum ether: ethyl acetate = 4:1, R_f = 0.5); ^{1}H NMR (600 MHz, $CDCl_3$) δ 7.30 (d, J = 8.0 Hz, 2H), 7.19 (d, J = 8.0 Hz, 2H), 4.66 (d, J = 12.2 Hz, 1H), 4.59 (dd, J = 8.0, 2.6 Hz, 1H), 4.56 (d, J = 12.2 Hz, 1H), 4.42 (d, J = 2.0 Hz, 1H), 4.23 (dd, J = 8.0, 0.8 Hz, 1H), 3.91 (dd, J = 12.8, 1.8 Hz,

1H), 3.73 (d, J = 12.8 Hz, 1H), 3.62 (d, J = 10.6 Hz, 1H), 3.58 (d, J = 10.6 Hz, 1H), 2.76-2.71 (m, 1H), 1.84-1.78 (m, 1H), 1.63-1.60 (m, 1H), 1.55 (s, 3H), 1.42 (s, 3H), 1.40 (s, 3H), 1.33 (s, 3H); ^{13}C NMR (150 MHz, $CDCl_3$) δ 137.1, 132.9, 127.9, 127.7, 112.5 (dd, $J_{C\text{-}F}$ = 284.4, 284.2 Hz), 108.9, 108.5, 102.7, 73.3, 71.6, 71.0, 70.2, 70.1, 61.0, 27.0 (t, $J_{C\text{-}F}$ = 11.4 Hz), 26.6, 25.8, 25.4, 24.0, 17.0 (t, $J_{C\text{-}F}$ = 11.4 Hz); ^{19}F NMR (565 MHz, $CDCl_3$) δ -125.9 (dtd, J = 154.7, 12.6, 3.9 Hz, 1F), -142.3 (ddd, J = 154.7, 12.6, 5.0 Hz, 1F). HRMS (ESI) calcd for $C_{22}H_{28}F_2NaO_6$ [M + Na $^+$], 449.1746; found: 449.1741.

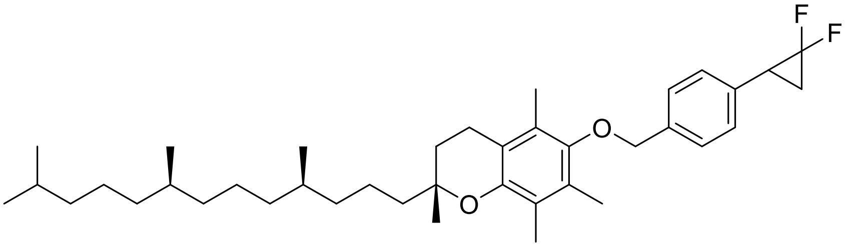

(2R)-6-((4-(2,2-Difluorocyclopropyl)benzyl)oxy)-2,5,7,8-tetramethyl-2-((4R,8R)-4,8,12-trimethyltridecyl)chromane (1r). (petroleum ether: ethyl acetate = 20:1, R_f = 0.6); ^{1}H NMR (600 MHz, $CDCl_3$) δ 7.46 (d, J = 7.8 Hz, 2H), 7.25 (d, J = 7.8 Hz, 2H), 4.67 (s, 2H), 2.79-2.73 (m, 1H), 2.58 (d, J = 6.6 Hz, 2H), 2.21 (s, 3H), 2.15 (s, 3H), 2.10 (s, 3H), 1.84-1.74 (m, 3H), 1.65-1.49 (m, 4H), 1.39-1.06 (m, 20H), 0.87-0.83 (m, 13H); ^{13}C NMR (150 MHz, $CDCl_3$) δ 148.0, 147.9, 137.0, 133.1, 128.1, 127.9, 127.8, 125.9, 122.9, 117.6, 112.5 (dd, $J_{C\text{-}F}$ = 284.4, 284.2 Hz), 74.8, 74.2, 40.0, 39.4, 37.6, 37.5, 37.4, 37.3, 32.8, 32.7, 31.3, 31.2, 28.0, 27.0 (t, $J_{C\text{-}F}$ = 11.2 Hz), 24.8, 24.4, 23.9, 22.7, 22.6, 21.0, 20.7, 19.7, 19.6, 17.0 (t, $J_{C\text{-}F}$ = 11.4 Hz), 12.8, 12.0, 11.8; ^{19}F NMR (565 MHz, $CDCl_3$) δ -125.8 (dtd, J = 154.7, 12.6, 3.9 Hz, 1F), -142.2 (ddd, J = 154.7, 12.6, 5.0 Hz, 1F). HRMS (APCI) calcd for $C_{39}H_{59}F_2O_2$ [M + H $^+$], 597.4478; found: 597.4480.

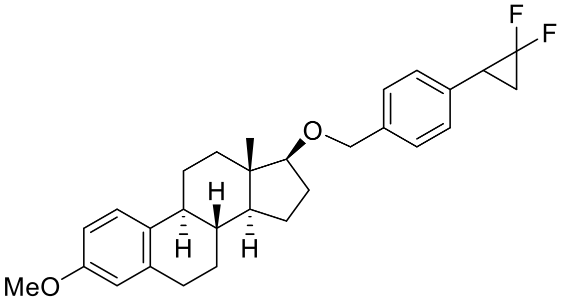

(8R,9S,13S,14S,17S)-17-((4-(2,2-Difluorocyclopropyl)benzyl)oxy)-3-methoxy-13-methyl-7,8,9,11,12,13,14,15,16,17-decahydro-6H-cyclopenta[a]phenanthrene (1s). (petroleum ether: ethyl acetate = 20:1, R_f = 0.4); ^{1}H NMR (600 MHz, $CDCl_3$) δ 7.33-7.31 (m, 2H), 7.20-7.19 (m, 3H), 6.71 (dd, J = 8.6, 2.4 Hz, 1H), 6.62 (d, J = 2.4 Hz, 1H), 4.55 (s, 2H), 3.77 (s, 3H), 3.49 (t, J = 8.4 Hz, 1H), 2.89-2.80 (m, 2H), 2.77-2.72 (m, 1H), 2.30-2.27 (m, 1H), 2.19-2.15 (m, 1H), 2.09-2.01 (m, 2H), 1.88-1.78 (m, 2H), 1.70-1.65 (m, 1H), 1.64-1.60 (m, 2H), 1.55-1.48 (m, 1H),

1.47-1.41 (m, 1H), 1.40-1.29 (m, 3H), 1.21-1.16 (m, 1H), 0.86 (s, 3H); ^{13}C NMR (150 MHz, $CDCl_3$) δ 157.4, 138.3, 138.0, 132.7, 127.9, 127.4, 126.3, 113.8, 112.6 (dd, $J_{C\text{-}F}$ = 284.4, 284.2 Hz), 111.5, 88.4, 71.3, 55.2, 50.2, 43.9, 43.4, 38.6, 37.9, 29.8, 28.0, 27.2, 27.0 (t, $J_{C\text{-}F}$ = 11.4 Hz), 26.4, 23.1, 17.0 (t, $J_{C\text{-}F}$ = 11.4 Hz), 11.8; ^{19}F NMR (565 MHz, $CDCl_3$) δ -125.4 (dtd, J = 154.7, 12.6, 3.9 Hz, 1F), -142.0 (ddd, J = 154.7, 12.6, 5.0 Hz, 1F). HRMS (APCI) calcd for $C_{29}H_{35}F_2O_2$ [M + H $^+$], 453.2600; found: 453.2597.

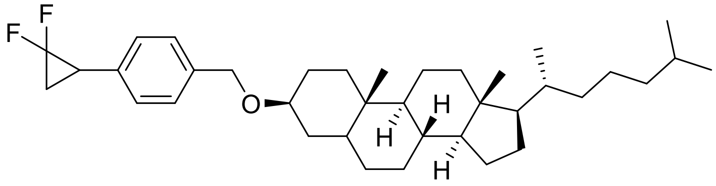

(3S,8R,9S,10S,13R,14S,17R)-3-((4-(2,2-Difluorocyclopropyl)benzyl)oxy)-10,13-dimethyl-17-((R)-6-methylheptan-2-yl)hexadecahydro-1H-cyclopenta[a]phenanthrene (1t). (petroleum ether: ethyl acetate = 50:1, R_f = 0.4); ^{1}H NMR (600 MHz, $CDCl_3$) δ 7.30 (d, J = 8.0 Hz, 2H), 7.19 (d, J = 8.0 Hz, 2H), 4.54 (d, J = 12.0 Hz, 1H), 4.62 (d, J = 12.0 Hz, 1H), 3.34-3.29 (m, 1H), 2.76-2.71 (m, 1H), 1.97-1.95 (m, 1H), 1.91-1.88 (m, 1H), 1.83-1.77 (m, 2H), 1.74-1.59 (m, 4H), 1.54-1.40 (m, 4H), 1.37-1.20 (m, 9H), 1.14-0.94 (m, 9H), 0.92-0.80 (m, 14H), 0.64-0.58 (m, 4H); ^{13}C NMR (150 MHz, $CDCl_3$) δ 138.2, 132.7, 128.0, 127.7, 112.6 (dd, $J_{C\text{-}F}$ = 284.4, 284.2 Hz), 78.1, 69.4, 56.5, 56.3, 54.4, 44.8, 42.6, 40.0, 39.5, 37.0, 36.2, 35.8, 35.5, 34.8, 32.1, 28.9, 28.3, 28.2, 28.0, 27.0 (t, $J_{C\text{-}F}$ = 11.4 Hz), 24.2, 23.8, 22.8, 22.6, 21.2, 18.7, 17.0 (t, $J_{C\text{-}F}$ = 11.4 Hz), 12.3, 12.1; ^{19}F NMR (565 MHz, $CDCl_3$) δ -125.9 (dtd, J = 154.7, 12.6, 3.9 Hz, 1F), -142.3 (ddd, J = 154.7, 12.6, 5.0 Hz, 1F). HRMS (APCI) calcd for $C_{37}H_{56}F_2NaO$ [M + H $^+$], 577.4191; found: 577.4186.

3. General procedure and characterization data for product 3

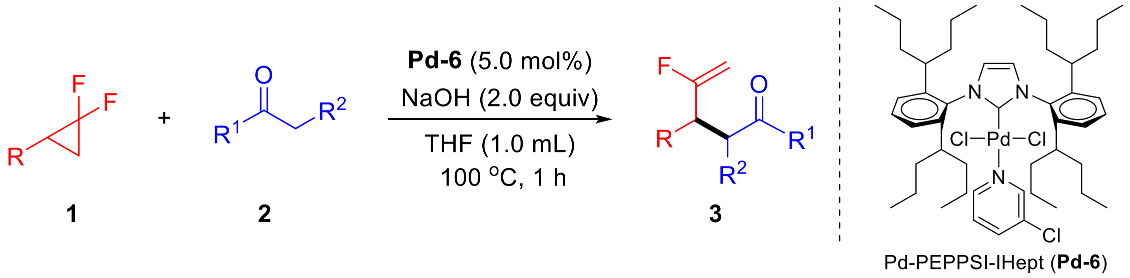

A flame-dried reaction tube (10 cm^3) equipped with a magnetic stir bar was charged with Pd-PEPPSI-IHept (4.5 mg, 5 mol%). The reaction tube was transferred to the glovebox and charged with **1** (0.1 mmol), **2** (0.2 mmol), NaOH (8.0 mg) and THF (1.0 mL). After that, the reaction mixture was sealed with aluminum cap, moved out of glovebox, and stirred at 100 °C for 1 h. After the mixture was cooled to room temperature, the resulting solution was directly filtered through a pad of silica gel by EtOAc (3.0 mL). The crude mixture was analyzed by GC-MS.

The solvent was evaporated *in vacuo* to give the crude product. NMR yield was determined by ^{1}H NMR using CH_2Br_2 as an internal standard. The residue was purified by column chromotography on silica gel to give the pure product **3**. **Note**: Use of the glovebox is not necessary. The reaction can be successfully performed with standard Schlenk line procedure.

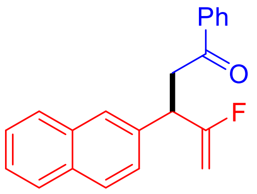

4-Fluoro-3-(naphthalen-2-yl)-1-phenylpent-4-en-1-one (3a). (29.0 mg, 95%). Isolated by column chromatography (petroleum ether: ethyl acetate = 20:1, R_f = 0.5); ^{1}H NMR (600 MHz, $CDCl_3$) δ 7.95-7.94 (m, 2H), 7.81-7.79 (m, 4H), 7.55-7.53 (m, 1H), 7.49-7.46 (m, 1H), 7.45-7.42 (m, 4H), 4.68 (dd, J = 17.6, 3.2 Hz, 1H), 4.51 (dt, J = 16.6, 7.0 Hz, 1H), 4.42 (dd, J = 49.8, 3.2 Hz, 1H), 3.70 (dd, J = 17.4, 7.0 Hz, 1H), 3.55 (dd, J = 17.4, 7.0 Hz, 1H); ^{13}C NMR (150 MHz, $CDCl_3$) δ 196.9, 166.8 (d, J_{C-F} = 259.8 Hz), 137.3, 136.6, 133.4, 133.3, 132.6, 128.6, 128.4, 128.0, 127.8, 127.6, 126.6, 126.1, 126.0, 125.8, 91.3 (d, J_{C-F} = 20.2 Hz), 43.5 (d, J_{C-F} = 26.6 Hz), 41.4 (d, J_{C-F} = 2.8 Hz); ^{19}F NMR (565 MHz, $CDCl_3$) δ -98.9 (1F). HRMS (APCI) calcd for $C_{21}H_{18}FO$ [M + H $^{+}$], 305.1336; found: 305.1334.

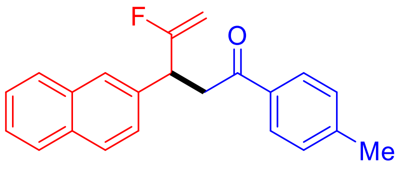

4-Fluoro-3-(naphthalen-2-yl)-1-(p-tolyl)pent-4-en-1-one (3b). (29.6 mg, 93%). Isolated by column chromatography (petroleum ether: ethyl acetate = 20:1, R_f = 0.5); ^{1}H NMR (600 MHz, $CDCl_3$) δ 7.85 (d, J = 8.0 Hz, 2H), 7.81-7.78 (m, 4H), 7.48 (d, J = 8.4 Hz, 1H), 7.46-7.41 (m, 2H), 7.23 (d, J = 8.0 Hz, 2H), 4.67 (dd, J = 17.5, 3.0 Hz, 1H), 4.50 (dt, J = 16.4, 7.0 Hz, 1H), 4.42 (dd, J = 49.9, 3.0 Hz, 1H), 3.66 (dd, J = 17.2, 7.0 Hz, 1H), 3.51 (dd, J = 17.2, 7.0 Hz, 1H), 2.38 (s, 3H); ^{13}C NMR (150 MHz, $CDCl_3$) δ 196.5, 167.0 (d, J_{C-F} = 260.0 Hz), 144.1, 137.4, 134.2, 133.4, 132.6, 129.3, 128.4, 128.2, 127.8, 127.6, 126.6, 126.1, 126.0, 125.8, 91.2 (d, J_{C-F} = 20.2 Hz), 43.5 (d, J_{C-F} = 26.6 Hz), 41.2 (d, J_{C-F} = 2.8 Hz), 21.6; ^{19}F NMR (565 MHz, $CDCl_3$) δ -98.8 (1F). HRMS (APCI) calcd for $C_{22}H_{20}FO$ [M + H $^{+}$], 319.1493; found: 319.1490.

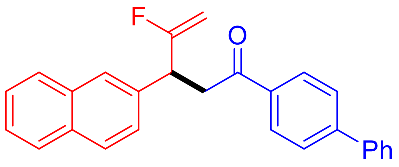

1-([1,1'-Biphenyl]-4-yl)-4-fluoro-3-(naphthalen-2-yl)pent-4-en-1-one (3c). (33.4 mg, 88%). Isolated by column chromatography (petroleum ether: ethyl acetate = 10:1, $R_f = 0.5$); ^{1}H NMR (600 MHz, $CDCl_3$) δ 8.01 (d, J = 8.4 Hz, 2H), 7.82-7.79 (m, 4H), 7.65 (d, J = 8.4 Hz, 2H), 7.59 (d, J = 7.4 Hz, 2H), 7.50 (d, J = 7.8 Hz, 1H), 7.46-7.41 (m, 4H), 7.38 (t, J = 7.4 Hz, 1H), 4.70 (dd, J = 17.5, 3.0 Hz, 1H), 4.53 (dt, J = 16.4, 7.0 Hz, 1H), 4.44 (dd, J = 49.9, 3.0 Hz, 1H), 3.72 (dd, J = 17.2, 7.0 Hz, 1H), 3.47 (dd, J = 17.2, 7.0 Hz, 1H); ^{13}C NMR (150 MHz, $CDCl_3$) δ 196.5, 166.9 (d, $J_{C\text{-}F}$ = 260.4 Hz), 145.9, 139.7, 137.3, 135.3, 133.4, 132.6, 128.9, 128.6, 128.4, 128.3, 127.8, 127.6, 127.3, 127.2, 126.6, 126.1, 126.0, 125.8, 91.3 (d, $J_{C\text{-}F}$ = 20.2 Hz), 43.6 (d, $J_{C\text{-}F}$ = 26.6 Hz), 41.4 (d, $J_{C\text{-}F}$ = 2.2 Hz); ^{19}F NMR (565 MHz, $CDCl_3$) δ -99.1 (1F). HRMS (APCI) calcd for $C_{27}H_{22}FO$ [M + H$^+$], 381.1649; found: 381.1643.

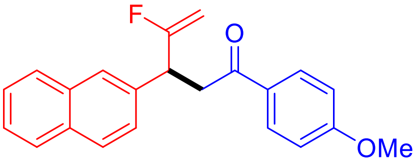

4-Fluoro-1-(4-methoxyphenyl)-3-(naphthalen-2-yl)pent-4-en-1-one (3d). (30.7 mg, 92%). Isolated by column chromatography (petroleum ether: ethyl acetate = 10:1, $R_f = 0.4$); ^{1}H NMR (600 MHz, $CDCl_3$) δ 7.93 (d, J = 8.6 Hz, 2H), 7.80-7.78 (m, 4H), 7.48 (d, J = 8.4 Hz, 1H), 7.45-7.41 (m, 2H), 6.89 (d, J = 8.6 Hz, 2H), 4.67 (dd, J = 17.5, 3.0 Hz, 1H), 4.50 (dt, J = 16.4, 7.0 Hz, 1H), 4.42 (dd, J = 49.9, 3.0 Hz, 1H), 3.82 (s, 3H), 3.64 (dd, J = 17.2, 7.0 Hz, 1H), 3.48 (dd, J = 17.2, 7.0 Hz, 1H); ^{13}C NMR (150 MHz, $CDCl_3$) δ 195.4, 167.0 (d, $J_{C\text{-}F}$ = 260.4 Hz), 163.6, 137.5, 133.4, 132.6, 130.3, 129.8, 128.3, 127.8, 127.5, 126.5, 126.1, 126.0, 125.8, 113.7, 91.2 (d, $J_{C\text{-}F}$ = 20.2 Hz), 55.4, 43.6 (d, $J_{C\text{-}F}$ = 26.8 Hz), 41.0 (d, $J_{C\text{-}F}$ = 2.8 Hz); ^{19}F NMR (565 MHz, $CDCl_3$) δ -98.8 (1F). HRMS (APCI) calcd for $C_{22}H_{19}FNaO_2$ [M + Na$^+$], 357.1261; found: 357.1270.

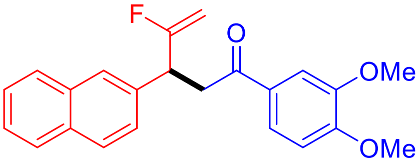

1-(3,4-Dimethoxyphenyl)-4-fluoro-3-(naphthalen-2-yl)pent-4-en-1-one (3e). (33.5 mg, 92%). Isolated by column chromatography (petroleum ether: ethyl acetate = 3:1, $R_f = 0.4$); ^{1}H NMR (600 MHz, $CDCl_3$) δ 7.82-7.79 (m, 4H), 7.60 (dd, J = 8.4, 1.6 Hz, 1H), 7.49-7.48 (m, 2H), 7.47-7.42 (m, 2H), 6.86 (d, J = 8.4 Hz, 1H), 4.69 (dd, J = 17.4, 3.0 Hz, 1H), 4.50 (dt, J = 16.8, 7.2 Hz, 1H), 4.43 (dd, J = 49.8, 3.0 Hz, 1H), 3.92 (s, 3H), 3.89 (s, 3H), 3.65 (dd, J = 17.0, 6.8 Hz, 1H), 3.51 (dd, J = 17.0, 6.8 Hz, 1H); ^{13}C NMR (150 MHz, $CDCl_3$) δ 195.5, 167.0 (d, $J_{C\text{-}F}$ = 260.0 Hz), 153.4, 149.0, 137.4, 133.4, 132.6, 129.9, 128.4, 127.8, 127.6, 126.5, 126.1, 126.0, 125.8, 122.7, 110.1, 109.9, 91.2 (d, $J_{C\text{-}F}$ = 20.6 Hz), 56.0, 55.9, 43.8 (d, $J_{C\text{-}F}$ = 27.0 Hz), 40.8 (d, $J_{C\text{-}F}$ = 2.8 Hz); ^{19}F NMR (565 MHz,

$CDCl_3$) δ -98.9 (1F). HRMS (APCI) calcd for $C_{23}H_{22}FO_3$ [M + H $^+$], 365.1548; found: 365.1544.

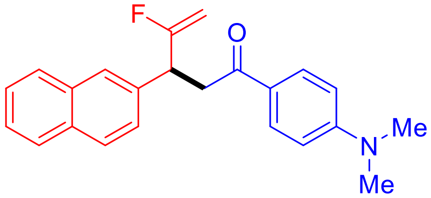

1-(4-(Dimethylamino)phenyl)-4-fluoro-3-(naphthalen-2-yl)pent-4-en-1-one (3f). (30.5 mg, 87%). Isolated by column chromatography (petroleum ether: ethyl acetate = 10:1, R_f = 0.4); ^{1}H NMR (600 MHz, $CDCl_3$) δ 7.88 (d, J = 9.0 Hz, 2H), 7.81-7.79 (m, 4H), 7.49 (d, J = 8.8 Hz, 1H), 7.46-7.42 (m, 2H), 6.62 (d, J = 9.0 Hz, 2H), 4.66 (dd, J = 17.6, 3.0 Hz, 1H), 4.51 (dt, J = 16.6, 7.2 Hz, 1H), 4.43 (dd, J = 49.9, 2.9 Hz, 1H), 3.60 (dd, J = 16.8, 7.2 Hz, 1H), 3.44 (dd, J = 16.8, 7.2 Hz, 1H), 3.04 (s, 6H); ^{13}C NMR (150 MHz, $CDCl_3$) δ 194.8, 167.2 (d, J_{C-F} = 260.2 Hz), 153.5, 137.8, 133.4, 132.5, 130.3, 128.3, 127.8, 127.5, 126.5, 126.1, 126.0, 125.7, 124.7, 110.6, 91.0 (d, J_{C-F} = 20.2 Hz), 43.8 (d, J_{C-F} = 26.8 Hz), 40.6 (d, J_{C-F} = 2.2 Hz), 40.0; ^{19}F NMR (565 MHz, $CDCl_3$) δ -98.6 (1F). HRMS (APCI) calcd for $C_{23}H_{23}FNO$ [M + H $^+$], 348.1758; found: 348.1751.

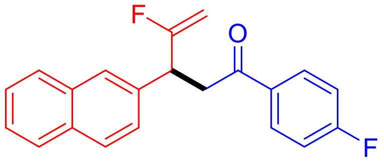

4-Fluoro-1-(4-fluorophenyl)-3-(naphthalen-2-yl)pent-4-en-1-one (3g). (29.0 mg, 90%). Isolated by column chromatography (petroleum ether: ethyl acetate = 20:1, R_f = 0.5); ^{1}H NMR (600 MHz, $CDCl_3$) δ 7.98-7.95 (m, 2H), 7.81-7.78 (m, 4H), 7.48-7.42 (m, 3H), 7.10 (t, J = 8.6 Hz, 2H), 4.68 (dd, J = 17.4, 3.0 Hz, 1H), 4.49 (dt, J = 16.4, 3.0 Hz, 1H), 4.42 (dd, J = 49.9, 3.0 Hz, 1H), 3.66 (dd, J = 17.4, 7.2 Hz, 1H), 3.48 (dd, J = 17.4, 7.2 Hz, 1H); ^{13}C NMR (150 MHz, $CDCl_3$) δ 195.3, 166.8 (d, J_{C-F} = 260.4 Hz), 165.8 (d, J_{C-F} = 255.4 Hz), 137.2, 133.4, 133.1 (d, J_{C-F} = 3.0 Hz), 132.6, 130.7, 130.6, 128.5, 127.8, 127.6, 126.6, 126.2, 125.9, 115.8 (d, J_{C-F} = 22.0 Hz), 91.4 (d, J_{C-F} = 20.2 Hz), 43.5 (d, J_{C-F} = 26.6 Hz), 41.3 (d, J_{C-F} = 2.2 Hz); ^{19}F NMR (565 MHz, $CDCl_3$) δ -98.8 (1F). HRMS (APCI) calcd for $C_{21}H_{17}F_2O$ [M + H $^+$], 323.1242; found: 323.1237.

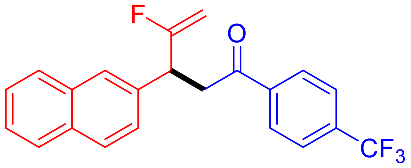

4-Fluoro-3-(naphthalen-2-yl)-1-(4-(trifluoromethyl)phenyl)pent-4-en-1-one (3h). (29.8 mg, 80%). Isolated by column chromatography (petroleum ether: ethyl acetate = 20:1, R_f = 0.5); ^{1}H NMR (600 MHz, $CDCl_3$) δ 8.04 (d, J = 8.2 Hz, 2H), 7.83-7.78 (m, 4H), 7.71 (d, J = 8.2 Hz, 2H), 7.48-7.44 (m, 3H), 4.70 (dd, J = 17.5, 3.2 Hz, 1H), 4.50

(dt, J = 16.4, 3.0 Hz, 1H), 4.43 (dd, J = 49.9, 3.2 Hz, 1H), 3.72 (dd, J = 17.4, 6.8 Hz, 1H), 3.57 (dd, J = 17.4, 6.8 Hz, 1H); ^{13}C NMR (150 MHz, $CDCl_3$) δ 196.1, 166.6 (d, J_{C-F} = 260.8 Hz), 139.3, 136.9, 134.6 (q, J_{C-F} = 32.4 Hz), 133.4, 132.6, 128.5, 128.4, 127.8, 127.6, 126.6, 126.3, 126.0, 125.8, 125.7 (q, J_{C-F} = 3.4 Hz), 123.4 (q, J_{C-F} = 272.4 Hz), 91.6 (d, J_{C-F} = 20.4 Hz), 43.5 (d, J_{C-F} = 26.6 Hz), 41.7 (d, J_{C-F} = 2.6 Hz); ^{19}F NMR (565 MHz, $CDCl_3$) δ -98.8 (1F). HRMS (APCI) calcd for $C_{22}H_{17}F_4O$ [M + H $^+$], 373.1210; found: 373.1204.

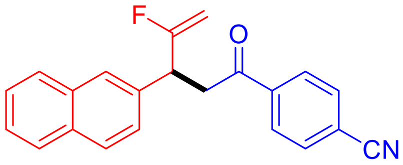

4-(4-Fluoro-3-(naphthalen-2-yl)pent-4-enoyl)benzonitrile (3i). (21.4 mg, 65%). Isolated by column chromatography (petroleum ether: ethyl acetate = 10:1, R_f = 0.4); ^1H NMR (600 MHz, $CDCl_3$) δ 8.01 (d, J = 8.4 Hz, 2H), 7.83-7.78 (m, 3H), 7.77 (s, 1H), 7.74 (d, J = 8.4 Hz, 2H), 7.48-7.44 (m, 3H), 4.70 (dd, J = 17.6, 3.2 Hz, 1H), 4.48 (dt, J = 16.4, 7.1 Hz, 1H), 4.42 (dd, J = 49.6, 3.2 Hz, 1H), 3.70 (dd, J = 17.4, 7.2 Hz, 1H), 3.56 (dd, J = 17.4, 7.2 Hz, 1H); ^{13}C NMR (150 MHz, $CDCl_3$) δ 195.8, 166.4 (d, J_{C-F} = 260.0 Hz), 139.5, 136.8, 133.4, 132.6, 132.5, 128.6, 128.4, 127.8, 127.6, 126.6, 126.3, 126.0, 125.8, 117.8, 116.6, 91.7 (d, J_{C-F} = 20.2 Hz), 43.5 (d, J_{C-F} = 27.1 Hz), 41.7 (d, J_{C-F} = 2.6 Hz); ^{19}F NMR (565 MHz, $CDCl_3$) δ -99.4 (1F). HRMS (APCI) calcd for $C_{22}H_{16}FNNaO$ [M + Na $^+$], 352.1108; found: 352.1108.

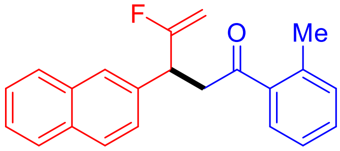

4-Fluoro-3-(naphthalen-2-yl)-1-(o-tolyl)pent-4-en-1-one (3j). (21.0 mg, 66%). Isolated by column chromatography (petroleum ether: ethyl acetate = 20:1, R_f = 0.5); ^1H NMR (600 MHz, $CDCl_3$) δ 7.82-7.78 (m, 3H), 7.75 (s, 1H), 7.60 (d, J = 7.7 Hz, 1H), 7.48-7.44 (m, 3H), 7.36 (t, J = 7.4 Hz, 1H), 7.26-7.23 (m, 1H), 7.21 (d, J = 7.5 Hz, 1H), 4.69 (dd, J = 17.6, 3.2 Hz, 1H), 4.47-4.36 (m, 2H), 3.60 (dd, J = 17.0, 7.0 Hz, 1H), 3.46 (dd, J = 17.0, 7.0 Hz, 1H), 2.33 (s, 3H); ^{13}C NMR (150 MHz, $CDCl_3$) δ 201.1, 166.9 (d, J_{C-F} = 260.2 Hz), 138.2, 137.8, 137.1, 133.4, 132.6, 132.0, 131.4, 128.4, 128.2, 127.8, 127.6, 126.7, 126.2, 125.9, 125.8, 125.7, 91.3 (d, J_{C-F} = 20.2 Hz), 44.4 (d, J_{C-F} = 2.6 Hz), 43.9 (d, J_{C-F} = 26.6 Hz), 20.9; ^{19}F NMR (565 MHz, $CDCl_3$) δ -98.6 (1F). HRMS (APCI) calcd for $C_{22}H_{20}FO$ [M + H $^+$], 319.1493; found: 319.1490.

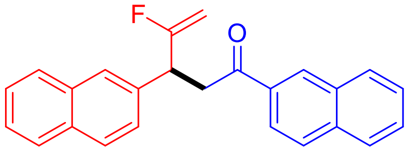

4-Fluoro-1,3-di(naphthalen-2-yl)pent-4-en-1-one (3k). (31.5 mg, 89%). Isolated by column chromatography (petroleum ether: ethyl acetate = 20:1, R_f = 0.4); ^{1}H NMR (600 MHz, $CDCl_3$) δ 8.48 (s, 1H), 8.01 (dd, J = 8.6, 1.5 Hz, 1H), 7.95 (d, J = 8.2 Hz, 1H), 7.87 (t, J = 8.6 Hz, 2H), 7.83-7.80 (m, 4H), 7.60 (t, J = 7.2 Hz, 1H), 7.55 (d, J = 7.4 Hz, 1H), 7.53 (dd, J = 8.6, 1.2 Hz, 1H), 7.47-7.43 (m, 2H), 4.71 (dd, J = 17.5, 3.0 Hz, 1H), 4.57 (dt, J = 16.4, 7.0 Hz, 1H), 4.46 (dd, J = 49.8, 3.0 Hz, 1H), 3.85 (dd, J = 17.2, 7.0 Hz, 1H), 3.69 (dd, J = 17.2, 7.0 Hz, 1H); ^{13}C NMR (150 MHz, $CDCl_3$) δ 196.9, 166.9 (d, $J_{C\text{-}F}$ = 260.0 Hz), 137.4, 135.7, 134.0, 133.4, 132.6, 132.4, 129.8, 129.6, 128.6, 128.5, 128.4, 127.8, 127.7, 127.6, 126.9, 126.6, 126.2, 126.0, 125.9, 123.7, 91.4 (d, $J_{C\text{-}F}$ = 20.1 Hz), 43.3 (d, $J_{C\text{-}F}$ = 27.0 Hz), 41.5 (d, $J_{C\text{-}F}$ = 2.8 Hz); ^{19}F NMR (565 MHz, $CDCl_3$) δ -98.9 (1F). HRMS (APCI) calcd for $C_{25}H_{20}FO$ [M + H $^+$], 355.1493; found: 355.1494.

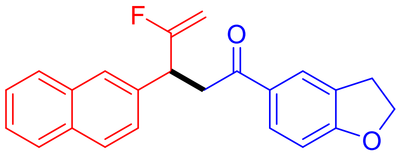

1-(2,3-Dihydrobenzofuran-6-yl)-4-fluoro-3-(naphthalen-2-yl)pent-4-en-1-one (3l). (30.8 mg, 89%). Isolated by column chromatography (petroleum ether: ethyl acetate = 20:1, R_f = 0.4); ^{1}H NMR (600 MHz, $CDCl_3$) δ 7.83 (s, 1H), 7.81-7.80 (m, 1H), 7.80-7.78 (m, 4H), 7.48 (d, J = 8.4 Hz, 1H), 7.46-7.42 (m, 2H), 6.78 (d, J = 8.4 Hz, 1H), 4.67 (dd, J = 17.5, 3.0 Hz, 1H), 4.63 (t, J = 8.8 Hz, 2H), 4.50 (dt, J = 16.5, 7.0 Hz, 1H), 4.42 (dd, J = 49.8, 3.0 Hz, 1H), 3.62 (dd, J = 17.0, 7.0 Hz, 1H), 3.47 (dd, J = 17.0, 7.0 Hz, 1H), 3.20 (t, J = 8.8 Hz, 2H); ^{13}C NMR (150 MHz, $CDCl_3$) δ 195.3, 167.0 (d, $J_{C\text{-}F}$ = 260.2 Hz), 164.5, 137.5, 133.4, 132.6, 130.1, 130.0, 128.3, 127.8, 127.6, 126.5, 126.1, 126.0, 125.8, 125.4, 109.0, 91.2 (d, $J_{C\text{-}F}$ = 20.6 Hz), 72.2, 43.7 (d, $J_{C\text{-}F}$ = 26.9 Hz), 41.0 (d, $J_{C\text{-}F}$ = 2.9 Hz), 28.9; ^{19}F NMR (565 MHz, $CDCl_3$) δ -98.8 (1F). HRMS (APCI) calcd for $C_{20}H_{19}FNaO_2$ [M + Na $^+$], 369.1261; found: 369.1259.

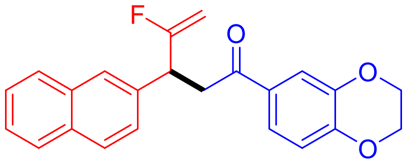

1-(2,3-Dihydrobenzo[b][1,4]dioxin-6-yl)-4-fluoro-3-(naphthalen-2-yl)pent-4-en-1-one (3m). (32.9 mg, 91%). Isolated by column chromatography (petroleum ether: ethyl acetate = 20:1, R_f = 0.4); ^{1}H NMR (600 MHz, $CDCl_3$) δ 7.81-7.80 (m, 2H), 7.79-7.78 (m, 2H), 7.51-7.49 (m, 2H), 7.48-7.42 (m, 3H), 6.89 (d, J = 9.0 Hz, 1H), 4.67 (dd, J = 17.5, 3.0 Hz, 1H), 4.48 (dt, J = 16.4, 7.0 Hz, 1H), 4.41 (dd, J = 49.9, 3.0 Hz, 1H), 4.30-4.28 (m, 2H), 4.26-4.25 (m, 2H), 3.61 (dd, J = 17.0, 7.0 Hz, 1H), 3.47 (dd, J = 17.0, 7.0 Hz, 1H); ^{13}C NMR (150 MHz, $CDCl_3$) δ 195.3, 167.0 (d, $J_{C\text{-}F}$ = 260.2 Hz), 148.2, 143.3, 137.4, 133.4, 132.6, 130.6, 128.4, 127.8, 127.6, 126.6, 126.1, 126.0, 125.8,

122.2, 117.6, 117.3, 91.2 (d, J_{C-F} = 20.1 Hz), 64.7, 64.1, 43.6 (d, J_{C-F} = 26.6 Hz), 41.0 (d, J_{C-F} = 2.9 Hz); ^{19}F NMR (565 MHz, $CDCl_3$) δ -98.8 (1F). HRMS (APCI) calcd for $C_{23}H_{20}FO_3$ [M + H $^+$], 363.1391; found: 363.1387.

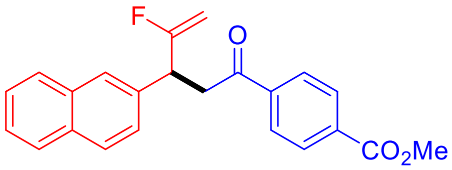

Methyl-4-(4-fluoro-3-(naphthalen-2-yl)pent-4-enoyl)benzoate (3n). (29.7 mg, 82%). Isolated by column chromatography (petroleum ether: ethyl acetate = 5:1, R_f = 0.5); ^{1}H NMR (600 MHz, $CDCl_3$) δ 8.10 (d, J = 8.4 Hz, 2H), 7.99 (d, J = 8.4 Hz, 2H), 7.82-7.79 (m, 4H), 7.48-7.43 (m, 3H), 4.70 (dd, J = 17.6, 3.2 Hz, 1H), 4.49 (dt, J = 16.4, 7.0 Hz, 1H), 4.42 (dd, J = 49.8, 3.2 Hz, 1H), 3.93 (s, 3H), 3.72 (dd, J = 17.4, 6.8 Hz, 1H), 3.57 (dd, J = 17.4, 6.8 Hz, 1H); ^{13}C NMR (150 MHz, $CDCl_3$) δ 196.5, 166.7 (d, J_{C-F} = 260.4 Hz), 166.1, 139.8, 137.0, 134.0, 133.4, 132.6, 129.9, 128.5, 127.9, 127.8, 127.6, 126.6, 126.2, 125.9, 125.8, 91.5 (d, J_{C-F} = 20.6 Hz), 52.5, 43.5 (d, J_{C-F} = 26.9 Hz), 41.7 (d, J_{C-F} = 2.2 Hz); ^{19}F NMR (565 MHz, $CDCl_3$) δ -99.1 (1F). HRMS (APCI) calcd for $C_{23}H_{19}FNaO_3$ [M + Na $^+$], 385.1210; found: 385.1204.

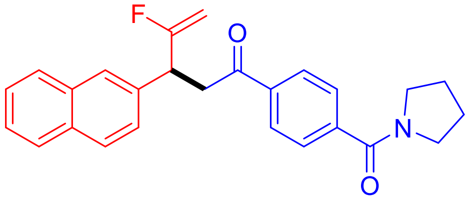

4-Fluoro-3-(naphthalen-2-yl)-1-(4-(pyrrolidine-1-carbonyl)phenyl)pent-4-en-1-one (3o). (38.1 mg, 95%). Isolated by column chromatography (petroleum ether: ethyl acetate = 2:1, R_f = 0.4); ^{1}H NMR (600 MHz, $CDCl_3$) δ 7.97 (d, J = 8.0 Hz, 2H), 7.82-7.78 (m, 4H), 7.57 (d, J = 8.0 Hz, 2H), 7.48-7.43 (m, 3H), 4.69 (dd, J = 17.4, 2.6 Hz, 1H), 4.49 (dt, J = 17.0, 7.0 Hz, 1H), 4.42 (dd, J = 49.8, 2.6 Hz, 1H), 3.69 (dd, J = 17.4, 6.6 Hz, 1H), 3.64 (t, J = 6.4 Hz, 2H), 3.56 (dd, J = 17.4, 6.6 Hz, 1H), 3.34 (t, J = 6.4 Hz, 2H), 1.97-1.93 (m, 2H), 1.89-1.85 (m, 2H); ^{13}C NMR (150 MHz, $CDCl_3$) δ 196.5, 168.4, 166.7 (d, J_{C-F} = 260.2 Hz), 141.7, 137.4, 137.1, 133.4, 132.6, 128.4, 128.1, 127.8, 127.6, 127.3, 126.6, 126.2, 125.9, 91.4 (d, J_{C-F} = 20.6 Hz), 49.4, 46.2, 43.6 (d, J_{C-F} = 26.8 Hz), 41.5 (d, J_{C-F} = 2.6 Hz), 26.3, 24.3; ^{19}F NMR (565 MHz, $CDCl_3$) δ -99.1 (1F). HRMS (ESI) calcd for $C_{26}H_{24}FNNaO_2$ [M + Na $^+$], 424.1683; found: 424.1681.

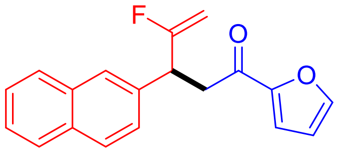

4-Fluoro-1-(furan-2-yl)-3-(naphthalen-2-yl)pent-4-en-1-one (3p). (27.3 mg, 87%). Isolated by column

chromatography (petroleum ether: ethyl acetate = 20:1, R_f = 0.3); ^{1}H NMR (600 MHz, $CDCl_3$) δ 7.81-7.79 (m, 4H), 7.56 (s, 1H), 7.48-7.43 (m, 3H), 7.19 (d, J = 3.6 Hz, 1H), 6.50 (d, J = 3.2 Hz, 1H), 4.68 (dd, J = 17.5, 3.0 Hz, 1H), 4.47 (dt, J = 16.4, 7.0 Hz, 1H), 4.44 (dd, J = 49.8, 3.0 Hz, 1H), 3.54 (dd, J = 17.2, 7.0 Hz, 1H), 3.40 (dd, J = 17.2, 7.0 Hz, 1H); ^{13}C NMR (150 MHz, $CDCl_3$) δ 186.1, 166.6 (d, $J_{C\text{-}F}$ = 260.2 Hz), 152.5, 146.5, 137.0, 133.4, 132.6, 128.4, 127.8, 127.6, 126.7, 126.1, 125.9, 125.8, 117.4, 112.4, 91.3 (d, $J_{C\text{-}F}$ = 20.2 Hz), 43.3 (d, $J_{C\text{-}F}$ = 26.7 Hz), 41.4 (d, $J_{C\text{-}F}$ = 3.0 Hz); ^{19}F NMR (565 MHz, $CDCl_3$) δ -99.0 (1F). HRMS (APCI) calcd for $C_{19}H_{16}FO_2$ [M + H $^+$], 295.1129; found: 295.1128.

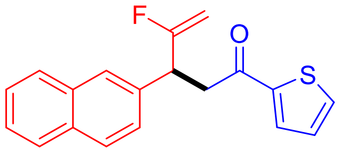

4-Fluoro-3-(naphthalen-2-yl)-1-(thiophen-2-yl)pent-4-en-1-one (3q). (27.8 mg, 96%). Isolated by column chromatography (petroleum ether: ethyl acetate = 20:1, R_f = 0.4); ^{1}H NMR (600 MHz, $CDCl_3$) δ 7.72-7.70 (m, 4H), 7.64 (d, J = 3.7 Hz, 1H), 7.51 (d, J = 4.8 Hz, 1H), 7.39 (d, J = 8.4 Hz, 1H), 7.37-7.33 (m, 2H), 7.00 (t, J = 4.2 Hz, 1H), 4.60 (dd, J = 17.4, 3.0 Hz, 1H), 4.40 (dt, J = 16.4, 7.0 Hz, 1H), 4.35 (dd, J = 49.9, 3.0 Hz, 1H), 3.53 (dd, J = 16.7, 7.0 Hz, 1H), 3.47 (dd, J = 16.7, 7.0 Hz, 1H); ^{13}C NMR (150 MHz, $CDCl_3$) δ 189.8, 166.5 (d, $J_{C\text{-}F}$ = 260.4 Hz), 143.9, 137.0, 134.0, 133.4, 132.6, 132.0, 128.4, 128.1, 127.8, 127.6, 126.6, 126.2, 125.9, 125.8, 91.5 (d, $J_{C\text{-}F}$ = 20.0 Hz), 43.7 (d, $J_{C\text{-}F}$ = 26.9 Hz), 42.0 (d, $J_{C\text{-}F}$ = 2.9 Hz); ^{19}F NMR (565 MHz, $CDCl_3$) δ -99.1 (1F). HRMS (APCI) calcd for $C_{19}H_{15}FNaOS$ [M + Na $^+$], 333.0720; found: 333.0717.

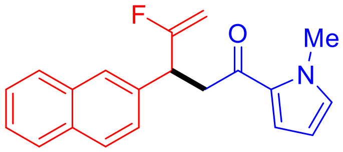

4-Fluoro-1-(1-methyl-1H-pyrrol-2-yl)-3-(naphthalen-2-yl)pent-4-en-1-one (3r). (25.8 mg, 84%). Isolated by column chromatography (petroleum ether: ethyl acetate = 10:1, R_f = 0.4); ^{1}H NMR (600 MHz, $CDCl_3$) δ 7.81-7.78 (m, 4H), 7.48-7.42 (m, 3H), 7.02 (dd, J = 4.0, 1.2 Hz, 1H), 6.77 (br, 1H), 6.11 (dd, J = 4.0, 2.5 Hz, 1H), 4.66 (dd, J = 17.6, 3.0 Hz, 1H), 4.44 (dt, J = 16.4, 7.2 Hz, 1H), 4.43 (dd, J = 49.6, 2.4 Hz, 1H), 3.87 (s, 3H), 3.48 (dd, J = 16.4, 7.2 Hz, 1H), 3.35 (dd, J = 16.4, 7.2 Hz, 1H); ^{13}C NMR (150 MHz, $CDCl_3$) δ 187.4, 167.1 (d, $J_{C\text{-}F}$ = 260.0 Hz), 137.4, 133.4, 132.6, 131.3, 130.6, 128.3, 127.8, 127.6, 126.6, 126.1, 126.0, 125.8, 119.2, 108.1, 91.1 (d, $J_{C\text{-}F}$ = 20.5 Hz), 43.8 (d, $J_{C\text{-}F}$ = 26.8 Hz), 41.6 (d, $J_{C\text{-}F}$ = 2.4 Hz), 37.7; ^{19}F NMR (565 MHz, $CDCl_3$) δ -98.4 (1F). HRMS (APCI) calcd for $C_{20}H_{19}FNO$ [M + H $^+$], 308.1445; found: 308.1441.

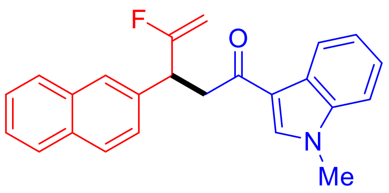

4-Fluoro-1-(1-methyl-1H-indol-3-yl)-3-(naphthalen-2-yl)pent-4-en-1-one (3s). (25.0 mg, 70%). Isolated by column chromatography (petroleum ether: ethyl acetate = 2:1, R_f = 0.5); ^{1}H NMR (600 MHz, $CDCl_3$) δ 8.37-8.35 (m, 1H), 7.81-7.76 (m, 4H), 7.64 (s, 1H), 7.51-7.49 (m, 1H), 7.44-7.39 (m, 2H), 7.28-7.24 (m, 3H), 4.66 (dd, J = 17.6, 2.8 Hz, 1H), 4.56 (dt, J = 17.2, 7.2 Hz, 1H), 4.47 (dd, J = 49.8, 2.8 Hz, 1H), 3.70 (s, 3H), 3.54 (dd, J = 16.0, 7.0 Hz, 1H), 3.35 (dd, J = 16.0, 7.0 Hz, 1H); ^{13}C NMR (150 MHz, $CDCl_3$) δ 191.6, 167.1 (d, $J_{C\text{-}F}$ = 260.0 Hz), 137.8, 137.4, 135.4, 133.4, 132.5, 128.3, 127.8, 127.5, 126.5, 126.2, 126.0, 125.7, 123.4, 122.6, 122.5, 116.5, 109.6, 91.2 (d, $J_{C\text{-}F}$ = 20.2 Hz), 43.8 (d, $J_{C\text{-}F}$ = 26.6 Hz), 42.5 (d, $J_{C\text{-}F}$ = 2.0 Hz), 33.4; ^{19}F NMR (565 MHz, $CDCl_3$) δ -99.0 (1F). HRMS (APCI) calcd for $C_{24}H_{21}FNO$ [M + H $^+$], 358.1602; found: 358.1602.

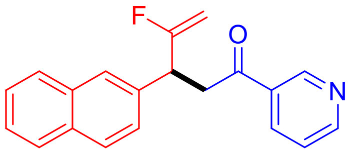

4-Fluoro-3-(naphthalen-2-yl)-1-(pyridin-3-yl)pent-4-en-1-one (3t). (22.6 mg, 74%). Isolated by column chromatography (petroleum ether: ethyl acetate = 1:2, R_f = 0.6); ^{1}H NMR (600 MHz, $CDCl_3$) δ 9.10 (s, 1H), 8.67 (d, J = 4.2 Hz, 1H), 8.09 (d, J = 7.8 Hz, 1H), 7.74-7.70 (m, 4H), 7.40-7.35 (m, 3H), 7.29 (dd, J = 7.6, 5.0 Hz, 1H), 4.62 (dd, J = 17.4, 2.8 Hz, 1H), 4.41 (dt, J = 16.4, 7.0 Hz, 1H), 4.34 (dd, J = 49.9, 3.0 Hz, 1H), 3.62 (dd, J = 17.2, 7.0 Hz, 1H), 3.48 (dd, J = 17.2, 7.0 Hz, 1H); ^{13}C NMR (150 MHz, $CDCl_3$) δ 195.9, 166.5 (d, $J_{C\text{-}F}$ = 260.2 Hz), 153.9, 149.5, 136.8, 135.3, 133.3, 132.6, 131.9, 128.5, 127.8, 127.6, 126.6, 126.2, 126.0, 125.8, 123.6, 91.6 (d, $J_{C\text{-}F}$ = 20.0 Hz), 43.4 (d, $J_{C\text{-}F}$ = 27.2 Hz), 41.6 (d, $J_{C\text{-}F}$ = 2.6 Hz); ^{19}F NMR (565 MHz, $CDCl_3$) δ -99.2 (1F). HRMS (APCI) calcd for $C_{20}H_{17}FNO$ [M + H $^+$], 306.1289; found: 306.1286.

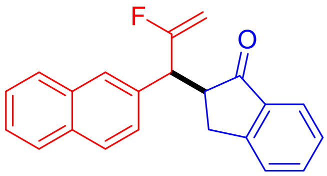

2-(2-Fluoro-1-(naphthalen-2-yl)allyl)-2,3-dihydro-1H-inden-1-one (3u). (dr = 1.6:1, 28.8 mg, 91%). Isolated by column chromatography (petroleum ether: ethyl acetate = 20:1, R_f = 0.3); **Major** and **Minor**: ^{1}H NMR (600 MHz, $CDCl_3$) δ 7.86-7.70 (m, 5H), 7.62-7.48 (m, 2H), 7.46-7.40 (m, 2H), 7.39-7.28 (m, 2H), 4.85 (dd, J = 17.6, 3.2 Hz, 0.62H), 4.67 (dd, J = 18.4, 3.0 Hz, 0.38H), 4.60 (dd, J = 49.8, 3.2 Hz, 0.62H), 4.59 (dd, J = 12.6, 4.2 Hz, 0.38H),

4.49 (dd, J = 18.8, 5.2 Hz, 0.64H), 4.19 (dd, J = 50.6, 3.0 Hz, 0.38H), 3.42-3.36 (m, 1.38H), 3.30-3.24 (m, 1H), 2.97 (dd, J = 17.2, 4.4 Hz, 0.62H); **Major** and **Minor**: ^{13}C NMR (150 MHz, $CDCl_3$) δ 205.7, 205.6, 166.5 (d, $J_{C\text{-}F}$ = 258.8 Hz), 165.7 (d, $J_{C\text{-}F}$ = 261.4 Hz), 153.4, 153.3, 136.6, 136.3, 134.9, 133.4, 133.2, 132.6, 132.5, 128.4, 128.2, 127.9, 127.6, 127.5, 127.4, 127.1, 126.5, 126.4, 126.3, 126.1, 126.0, 125.9, 124.2, 124.0, 93.8 (d, $J_{C\text{-}F}$ = 19.6 Hz), 92.1 (d, $J_{C\text{-}F}$ = 20.2 Hz), 50.0, 49.5 (d, $J_{C\text{-}F}$ = 2.0 Hz), 48.5 (d, $J_{C\text{-}F}$ = 25.2 Hz), 48.1 (d, $J_{C\text{-}F}$ = 26.4 Hz), 30.4, 30.0; ^{19}F NMR (565 MHz, $CDCl_3$) δ -96.2, -97.0. HRMS (APCI) calcd for $C_{22}H_{18}FO$ [M + H $^+$], 317.1336; found: 317.1335.

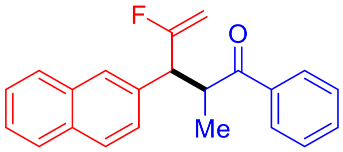

4-Fluoro-2-methyl-3-(naphthalen-2-yl)-1-phenylpent-4-en-1-one (3v). (dr = 2:1, 29.3 mg, 92%). Isolated by column chromatography (petroleum ether: ethyl acetate = 20:1, R_f = 0.4); **Major**: ^{1}H NMR (600 MHz, $CDCl_3$) δ 8.07 (d, J = 7.4 Hz, 1H), 7.87-7.82 (m, 3H), 7.73-7.68 (m, 3H), 7.53-7.45 (m, 2H), 7.39-7.34 (m, 3H), 4.67 (dd, J = 17.4, 3.0 Hz, 1H), 4.61 (dd, J = 49.8, 3.0 Hz, 1H), 4.41-4.34 (m, 1H), 4.20-4.14 (m, 1H), 1.41 (d, J = 7.0 Hz, 3H); **Major** and **Minor**: ^{13}C NMR (150 MHz, $CDCl_3$) δ 202.7, 202.1, 166.1 (d, $J_{C\text{-}F}$ = 261.2 Hz), 165.5 (d, $J_{C\text{-}F}$ = 261.4 Hz), 137.0, 136.3, 136.1, 133.5, 133.3, 133.1, 132.8, 132.5, 128.8, 128.6, 128.5, 128.3, 128.1, 127.8, 127.6, 127.4, 126.7, 126.3, 126.2, 126.1, 125.9, 125.6, 92.7 (d, $J_{C\text{-}F}$ = 20.2 Hz), 91.4 (d, $J_{C\text{-}F}$ = 20.6 Hz), 51.5 (d, $J_{C\text{-}F}$ = 23.2 Hz), 51.3 (d, $J_{C\text{-}F}$ = 23.2 Hz), 42.3 (d, $J_{C\text{-}F}$ = 3.2 Hz), 42.0 (d, $J_{C\text{-}F}$ = 3.2 Hz), 17.6, 17.1; ^{19}F NMR (565 MHz, $CDCl_3$) δ -104.4, -98.1. HRMS (APCI) calcd for $C_{22}H_{20}FO$ [M + H $^+$], 319.1493; found: 319.1491.

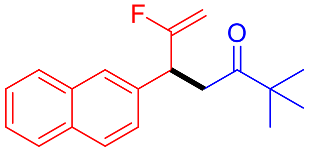

6-Fluoro-2,2-dimethyl-5-(naphthalen-2-yl)hept-6-en-3-one (3w). (22.7 mg, 80%). Isolated by column chromatography (petroleum ether: ethyl acetate = 20:1, R_f = 0.6); ^{1}H NMR (600 MHz, $CDCl_3$) δ 7.80-7.79 (m, 3H), 7.73 (s, 1H), 7.47-7.41 (m, 3H), 4.64 (dd, J = 17.5, 3.0 Hz, 1H), 4.42-4.31 (m, 2H), 3.17 (dd, J = 17.6, 6.8 Hz, 1H), 3.07 (dd, J = 17.6, 6.8 Hz, 1H), 1.07 (s, 9H); ^{13}C NMR (150 MHz, $CDCl_3$) δ 212.3, 167.1 (d, $J_{C\text{-}F}$ = 260.1 Hz), 137.5, 133.4, 132.5, 128.3, 127.8, 127.6, 126.6, 126.1, 125.9, 125.8, 91.0 (d, $J_{C\text{-}F}$ = 20.7 Hz), 44.1, 43.3 (d, $J_{C\text{-}F}$ = 26.6 Hz), 39.7 (d, $J_{C\text{-}F}$ = 2.2 Hz), 26.1; ^{19}F NMR (565 MHz, $CDCl_3$) δ -99.3 (1F). HRMS (APCI) calcd for $C_{19}H_{22}FO$ [M + H $^+$], 285.1649; found: 285.1647.

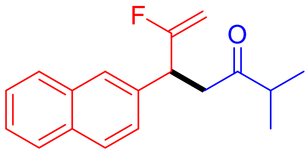

6-Fluoro-2-methyl-5-(naphthalen-2-yl)hept-6-en-3-one (3x). (16.5 mg, 61%). Isolated by column chromatography (petroleum ether: ethyl acetate = 20:1, R_f = 0.5); ^{1}H NMR (600 MHz, $CDCl_3$) δ 7.80 (d, J = 8.0 Hz, 3H), 7.73 (s, 1H), 7.48-7.44 (m, 2H), 7.42 (d, J = 8.4 Hz, 1H), 4.65 (dd, J = 17.6, 3.2 Hz, 1H), 4.38 (dd, J = 49.8, 3.2 Hz, 1H), 4.31 (dt, J = 16.6, 7.6 Hz, 1H), 3.15 (dd, J = 17.2, 6.8 Hz, 1H), 3.02 (dd, J = 17.2, 6.8 Hz, 1H), 2.55 (m, 1H). 1.07 (d, J = 7.0 Hz, 3H), 0.98 (d, J = 7.0 Hz, 3H); ^{13}C NMR (150 MHz, $CDCl_3$) δ 211.3, 167.0(d, $J_{C\text{-}F}$ = 260.0 Hz), 137.3, 133.4, 132.6, 128.4, 127.8, 127.6, 126.6, 126.2, 125.9, 125.8, 91.1 (d, $J_{C\text{-}F}$ = 20.2 Hz), 43.3 (d, $J_{C\text{-}F}$ = 26.6 Hz), 43.2 (d, $J_{C\text{-}F}$ = 2.4 Hz), 41.3, 17.9, 17.8; ^{19}F NMR (565 MHz, $CDCl_3$) δ -99.2 (1F). HRMS (ESI) calcd for $C_{18}H_{19}FNaO$ [M + Na^+], 293.1312; found: 393.1312.

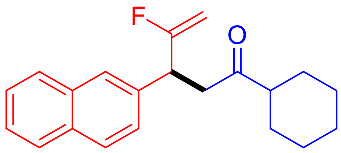

1-Cyclohexyl-4-fluoro-3-(naphthalen-2-yl)pent-4-en-1-one (3y). (24.5 mg, 79%). Isolated by column chromatography (petroleum ether: ethyl acetate = 50:1, R_f = 0.3); ^{1}H NMR (600 MHz, $CDCl_3$) δ 7.80-7.78 (m, 3H), 7.72 (s, 1H), 7.48-7.43 (m, 2H), 7.41 (dd, J = 8.6, 1.4 Hz, 1H), 4.64 (dd, J = 17.4, 3.0 Hz, 1H), 4.36 (dd, J = 49.8, 3.2 Hz, 1H), 4.31 (dt, J = 16.4, 7.0 Hz, 1H), 3.14 (dd, J = 17.4, 7.0 Hz, 1H), 3.00 (dd, J = 17.4, 7.0 Hz, 1H), 2.31-2.27 (m, 1H), 1.83-1.81 (m, 1H), 1.77-1.70 (m, 3H), 1.64-1.61 (m, 1H), 1.32-1.13 (m, 5H); ^{13}C NMR (150 MHz, $CDCl_3$) δ 210.6, 167.0 (d, $J_{C\text{-}F}$ = 260.5 Hz), 137.4, 133.4, 132.6, 128.3, 127.8, 127.6, 126.6, 126.1, 125.9, 125.8, 90.1 (d, $J_{C\text{-}F}$ = 20.7 Hz), 51.1, 43.4 (d, $J_{C\text{-}F}$ = 2.2 Hz), 43.1 (d, $J_{C\text{-}F}$ = 26.6 Hz), 28.1, 28.0, 25.7, 25.5; ^{19}F NMR (565 MHz, $CDCl_3$) δ -99.1 (1F). HRMS (APCI) calcd for $C_{21}H_{24}FO$ [M + H $^+$], 311.1806; found: 311.1803.

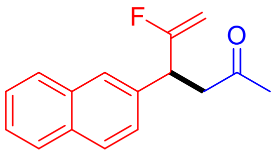

5-Fluoro-4-(naphthalen-2-yl)hex-5-en-2-one (3z). (18.2 mg, 75%). Isolated by column chromatography (petroleum ether: ethyl acetate = 20:1, R_f = 0.6); ^{1}H NMR (600 MHz, $CDCl_3$) δ 7.81-7.80 (m, 3H), 7.73 (s, 1H), 7.48-7.44 (m, 2H), 7.41 (d, J = 8.4 Hz, 1H), 4.66 (dd, J = 17.4, 2.8 Hz, 1H), 4.36 (dd, J = 49.8, 2.8 Hz, 1H), 4.27 (dt, J = 17.4, 7.2 Hz, 1H), 3.13 (dd, J = 17.4, 7.0 Hz, 1H), 2.99 (dd, J = 17.4, 7.0 Hz, 1H), 2.12 (s, 3H); ^{13}C NMR (150 MHz, $CDCl_3$) δ 205.5, 166.7 (d, $J_{C\text{-}F}$ = 260.2 Hz), 137.0, 133.4, 132.6, 128.4, 127.8, 127.6, 126.6, 126.2, 125.9, 125.8, 91.2 (d, $J_{C\text{-}F}$ = 20.6 Hz), 46.2 (d, $J_{C\text{-}F}$ = 2.4 Hz), 43.3 (d, $J_{C\text{-}F}$ = 26.8 Hz), 30.5; ^{19}F NMR (565 MHz,

$CDCl_3$) δ -99.0 (1F). HRMS (APCI) calcd for $C_{16}H_{16}FO$ [M + H $^+$], 243.1180; found: 243.1170.

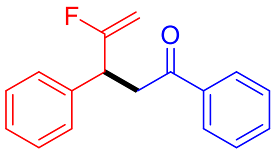

4-Fluoro-1,3-diphenylpent-4-en-1-one (3aa). (24.4 mg, 96%). Isolated by column chromatography (petroleum ether: ethyl acetate = 20:1, R_f = 0.5); ^{1}H NMR (600 MHz, $CDCl_3$) δ 7.93 (d, J = 7.6 Hz, 2H), 7.55 (t, J = 7.4 Hz, 1H), 7.44 (t, J = 7.3 Hz, 2H), 7.35-7.30 (m, 4H), 7.24 (t, J = 7.4 Hz, 1H), 4.64 (dd, J = 17.6, 3.0 Hz, 1H), 4.38 (dd, J = 49.8, 3.0 Hz, 1H), 4.33 (dt, J = 17.0, 7.0 Hz, 1H), 3.61 (dd, J = 17.4, 7.0 Hz, 1H), 3.44 (dd, J = 17.4, 7.0 Hz, 1H); ^{13}C NMR (150 MHz, $CDCl_3$) δ 197.1, 167.0 (d, J_{C-F} = 260.0 Hz), 140.0, 136.7, 133.3, 128.8, 128.7, 128.1, 127.9, 127.3, 91.1 (d, J_{C-F} = 21.0 Hz), 43.5 (d, J_{C-F} = 26.4 Hz), 41.5 (d, J_{C-F} = 3.2 Hz); ^{19}F NMR (565 MHz, $CDCl_3$) δ -99.2 (1F). HRMS (APCI) calcd for $C_{17}H_{16}FO$ [M + H $^+$], 255.1180; found: 255.1178.

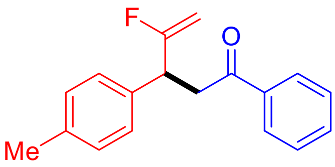

4-Fluoro-1-phenyl-3-(*p*-tolyl)pent-4-en-1-one (3ab) (24.7 mg, 92%). Isolated by column chromatography (petroleum ether: ethyl acetate = 20:1, R_f = 0.5); ^{1}H NMR (600 MHz, $CDCl_3$) δ 7.93 (d, J = 7.6 Hz, 2H), 7.54 (t, J = 7.4 Hz, 1H), 7.44 (t, J = 7.6 Hz, 2H), 7.23 (d, J = 7.6 Hz, 2H), 7.12 (d, J = 7.6 Hz, 2H), 4.62 (dd, J = 17.6, 3.0 Hz, 1H), 4.36 (dd, J = 49.8, 3.0 Hz, 1H), 4.29 (dt, J = 17.0, 7.2 Hz, 1H), 3.59 (dd, J = 17.2, 7.0 Hz, 1H), 3.42 (dd, J = 17.2, 7.0 Hz, 1H), 2.30 (s, 3H); ^{13}C NMR (150 MHz, $CDCl_3$) δ 197.1, 167.1 (d, J_{C-F} = 260.0 Hz), 136.9, 136.8, 133.2, 129.3, 128.6, 128.0, 127.6, 90.8 (d, J_{C-F} = 20.8 Hz), 43.0 (d, J_{C-F} = 26.8 Hz), 41.4 (d, J_{C-F} = 2.4 Hz), 21.1; ^{19}F NMR (565 MHz, $CDCl_3$) δ -99.3 (1F). HRMS (APCI) calcd for $C_{18}H_{18}FO$ [M + H $^+$], 269.1336; found: 269.1332.

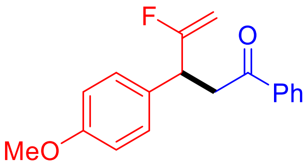

4-Fluoro-3-(4-methoxyphenyl)-1-phenylpent-4-en-1-one (3ac). (27.0 mg, 95%). Isolated by column chromatography (petroleum ether: ethyl acetate = 20:1, R_f = 0.3); ^{1}H NMR (600 MHz, $CDCl_3$) δ 7.85 (d, J = 7.6 Hz, 2H), 7.47 (t, J = 7.4 Hz, 1H), 7.36 (t, J = 7.6 Hz, 2H), 7.18 (d, J = 8.4 Hz, 2H), 6.76 (d, J = 8.4 Hz, 2H), 4.53 (dd, J = 17.5, 3.0 Hz, 1H), 4.26 (dd, J = 49.8, 3.0 Hz, 1H), 4.19 (dt, J = 16.4, 7.2 Hz, 1H), 3.68 (s, 3H), 3.49 (dd, J = 17.4, 6.8 Hz, 1H), 3.34 (dd, J = 17.4, 6.8 Hz, 1H); ^{13}C NMR (150 MHz, $CDCl_3$) δ 197.1, 167.3 (d, J_{C-F} = 260.4 Hz),

158.6, 136.7, 133.2, 131.9, 128.8, 128.6, 128.0, 114.0, 90.7 (d, $J_{C\text{-}F}$ = 20.5 Hz), 55.2, 42.6 (d, $J_{C\text{-}F}$ = 26.8 Hz), 41.5 (d, $J_{C\text{-}F}$ = 2.2 Hz); ^{19}F NMR (565 MHz, $CDCl_3$) δ -99.2 (1F). HRMS (APCI) calcd for $C_{18}H_{18}FO_2$ [M + H $^+$], 285.1285; found: 285.1279.

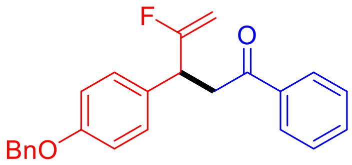

3-(4-(Benzyloxy)phenyl)-4-fluoro-1-phenylpent-4-en-1-one (3ad). (30.6 mg, 85%). Isolated by column chromatography (petroleum ether: ethyl acetate = 20:1, R_f = 0.3); ^{1}H NMR (600 MHz, $CDCl_3$) δ 7.92 (d, J = 7.4 Hz, 2H), 7.54 (t, J = 7.4 Hz, 1H), 7.43 (t, J = 7.6 Hz, 2H), 7.40 (d, J = 7.4 Hz, 2H), 7.36 (t, J = 7.6 Hz, 2H), 7.30 (t, J = 7.2 Hz, 1H), 7.25 (d, J = 8.6 Hz, 2H), 6.92 (d, J = 8.6 Hz, 2H), 5.01 (s, 2H), 4.61 (dd, J = 17.5, 3.0 Hz, 1H), 4.34 (dd, J = 49.8, 3.0 Hz, 1H), 4.27 (dt, J = 16.4, 7.2 Hz, 1H), 3.56 (dd, J = 17.4, 6.8 Hz, 1H), 3.40 (dd, J = 17.4, 6.8 Hz, 1H); ^{13}C NMR (150 MHz, $CDCl_3$) δ 197.1, 167.2 (d, $J_{C\text{-}F}$ = 260.4 Hz), 157.9, 136.9, 136.7, 133.2, 132.2, 128.8, 128.6, 128.5, 128.0, 127.9, 127.4, 114.9, 90.7 (d, $J_{C\text{-}F}$ = 20.5 Hz), 70.0, 42.6 (d, $J_{C\text{-}F}$ = 26.8 Hz), 41.5 (d, $J_{C\text{-}F}$ = 2.4 Hz); ^{19}F NMR (565 MHz, $CDCl_3$) δ -99.3 (1F). HRMS (ESI) calcd for $C_{24}H_{21}FNaO_2$ [M + Na $^+$], 383.1418; found: 383.1417.

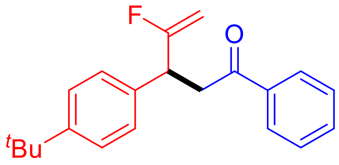

3-(4-(*Tert*-butyl)phenyl)-4-fluoro-1-phenylpent-4-en-1-one (3ae). (29.5 mg, 95%). Isolated by column chromatography (petroleum ether: ethyl acetate = 20:1, R_f = 0.5); ^{1}H NMR (600 MHz, $CDCl_3$) δ 7.94 (d, J = 7.9 Hz, 2H), 7.55 (t, J = 7.5 Hz, 1H), 7.44 (t, J = 7.5 Hz, 2H), 7.33 (d, J = 8.2 Hz, 2H), 7.27 (d, J = 8.2 Hz, 2H), 4.62 (dd, J = 17.6, 3.0 Hz, 1H), 4.37 (dd, J = 49.8, 2.9 Hz, 1H), 4.31 (dt, J = 17.8, 7.0 Hz, 1H), 3.61 (dd, J = 17.4, 7.4 Hz, 1H), 3.42 (dd, J = 17.4, 7.4 Hz, 1H), 1.29 (s, 9H); ^{13}C NMR (150 MHz, $CDCl_3$) δ 196.1, 166.0 (d, $J_{C\text{-}F}$ = 260.0 Hz), 149.0, 135.8, 135.7, 132.2, 127.6, 127.0, 126.3, 124.6, 89.9 (d, $J_{C\text{-}F}$ = 20.9 Hz), 41.9 (d, $J_{C\text{-}F}$ = 26.6 Hz), 40.5 (d, $J_{C\text{-}F}$ = 2.2 Hz), 33.4, 30.3; ^{19}F NMR (565 MHz, $CDCl_3$) δ -99.5 (1F). HRMS (APCI) calcd for $C_{21}H_{24}FO$ [M + H $^+$], 311.1806; found: 311.1805.

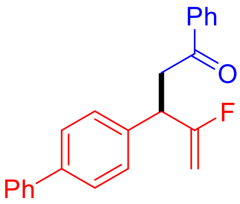

3-([1,1'-Biphenyl]-4-yl)-4-fluoro-1-phenylpent-4-en-1-one (3af). (28.7 mg, 87%). Isolated by column chromatography (petroleum ether: ethyl acetate = 10:1, $R_f = 0.4$); ^{1}H NMR (600 MHz, $CDCl_3$) δ 7.95 (d, J = 7.6 Hz, 2H), 7.56-7.53 (m, 5H), 7.45-7.39 (m, 6H), 7.32 (t, J = 7.3 Hz, 1H), 4.66 (dd, J = 17.6, 2.9 Hz, 1H), 4.42 (dd, J = 49.8, 2.9 Hz, 1H), 4.40-4.35 (m, 1H), 3.63 (dd, J = 17.4, 7.0 Hz, 1H), 3.48 (dd, J = 17.4, 6.8 Hz, 1H); ^{13}C NMR (150 MHz, $CDCl_3$) δ 196.9, 166.8 (d, J_{C-F} = 259.8 Hz), 140.6, 140.1, 138.9, 136.6, 133.3, 128.7, 128.6, 128.2, 128.0, 127.4, 127.2, 127.0, 91.3 (d, J_{C-F} = 20.2 Hz), 43.1 (d, J_{C-F} = 26.6 Hz), 41.4 (d, J_{C-F} = 2.7 Hz); ^{19}F NMR (565 MHz, $CDCl_3$) δ -99.2 (1F). HRMS (APCI) calcd for $C_{23}H_{20}FO$ [M + H $^+$], 331.1493; found: 331.1493.

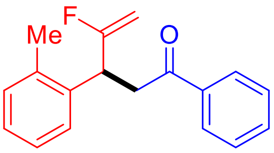

4-Fluoro-1-phenyl-3-(o-tolyl)pent-4-en-1-one (3ag). (23.3 mg, 87%). Isolated by column chromatography (petroleum ether: ethyl acetate = 20:1, $R_f = 0.5$); ^{1}H NMR (600 MHz, $CDCl_3$) δ 7.96 (d, J = 7.4 Hz, 2H), 7.58 (t, J = 7.4 Hz, 1H), 7.47 (t, J = 7.6 Hz, 2H), 7.31 (d, J = 7.2 Hz, 1H), 7.20-7.15 (m, 3H), 4.64 (dd, J = 17.6, 3.0 Hz, 1H), 4.60 (dt, J = 17.0, 7.4 Hz, 1H), 4.28 (dd, J = 49.8, 3.0 Hz, 1H), 3.65 (dd, J = 17.6, 7.0 Hz, 1H), 3.48 (dd, J = 17.6, 7.0 Hz, 1H), 2.47 (s, 3H); ^{13}C NMR (150 MHz, $CDCl_3$) δ 197.1, 166.8 (d, J_{C-F} = 260.0 Hz), 137.9, 136.7, 136.5, 133.3, 130.7, 128.6, 128.0, 127.0, 126.4, 126.2, 91.0 (d, J_{C-F} = 20.8 Hz), 41.4 (d, J_{C-F} = 2.2 Hz), 38.8 (d, J_{C-F} = 26.4 Hz), 19.6; ^{19}F NMR (565 MHz, $CDCl_3$) δ -98.6 (1F). HRMS (APCI) calcd for $C_{18}H_{18}FO$ [M + H $^+$], 269.1336; found: 269.1332.

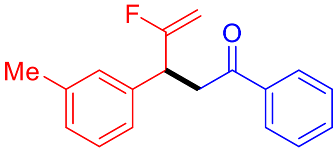

4-Fluoro-1-phenyl-3-(m-tolyl)pent-4-en-1-one (3ah). (24.7 mg, 92%). Isolated by column chromatography (petroleum ether: ethyl acetate = 50:1, $R_f = 0.5$); ^{1}H NMR (600 MHz, $CDCl_3$) δ 7.94 (d, J = 7.6 Hz, 2H), 7.55 (t, J = 7.4 Hz, 1H), 7.45 (t, J = 7.6 Hz, 2H), 7.20 (t, J = 7.6 Hz, 1H), 7.15-7.13 (m, 2H), 7.06 (d, J = 7.2 Hz, 1H), 4.63 (dd, J = 17.6, 2.8 Hz, 1H), 4.37 (dd, J = 49.6, 2.8 Hz, 1H), 4.29 (dt, J = 17.2, 7.2 Hz, 1H), 3.60 (dd, J = 17.2, 7.2 Hz, 1H), 3.41 (dd, J = 17.2, 7.2 Hz, 1H), 2.33 (s, 3H); ^{13}C NMR (150 MHz, $CDCl_3$) δ 197.0, 167.0 (d, J_{C-F} = 260.0

Hz), 139.9, 138.3, 136.7, 133.2, 128.6, 128.5, 128.4, 128.1, 128.0, 124.7, 90.9 (d, $J_{C\text{-}F}$ = 20.2 Hz), 43.4 (d, $J_{C\text{-}F}$ = 26.6 Hz), 41.5 (d, $J_{C\text{-}F}$ = 2.4 Hz), 21.4; ^{19}F NMR (565 MHz, $CDCl_3$) δ -99.2 (1F). HRMS (ESI) calcd for $C_{18}H_{17}FNaO$ [M + Na$^+$], 291.1156; found: 291.1153.

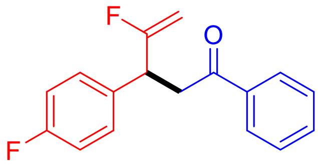

4-Fluoro-3-(4-fluorophenyl)-1-phenylpent-4-en-1-one (3ai). (19.9 mg, 73%). Isolated by column chromatography (petroleum ether: ethyl acetate = 20:1, R_f = 0.5); ^{1}H NMR (600 MHz, $CDCl_3$) δ 7.94-7.92 (m, 2H), 7.57 (t, J = 7.5 Hz, 1H), 7.45 (t, J = 7.5 Hz, 2H), 7.32-7.30 (m, 2H), 7.01-6.99 (m, 2H), 4.65 (dd, J = 17.6, 3.2 Hz, 1H), 4.37 (dd, J = 49.8, 3.2 Hz, 1H), 4.32 (dt, J = 17.0, 7.0 Hz, 1H), 3.58 (dd, J = 17.4, 6.5 Hz, 1H), 3.43 (dd, J = 17.4, 6.5 Hz, 1H); ^{13}C NMR (150 MHz, $CDCl_3$) δ 196.8, 166.8 (d, $J_{C\text{-}F}$ = 260.0 Hz), 161.9 (d, $J_{C\text{-}F}$ = 245.2 Hz), 136.6, 135.6 (d, $J_{C\text{-}F}$ = 3.2 Hz), 133.4, 129.4 (d, $J_{C\text{-}F}$ = 8.0 Hz), 128.7, 128.0, 115.5 (d, $J_{C\text{-}F}$ = 21.4 Hz), 91.1 (d, $J_{C\text{-}F}$ = 20.6 Hz), 42.7 (d, $J_{C\text{-}F}$ = 26.6 Hz), 41.4 (d, $J_{C\text{-}F}$ = 2.2 Hz); ^{19}F NMR (565 MHz, $CDCl_3$) δ -99.5 (1F), -115.4 (1F). HRMS (APCI) calcd for $C_{17}H_{15}F_2O$ [M + H $^+$], 273.1086; found: 273.1084.

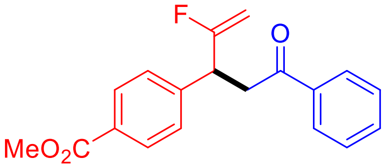

Methyl -4-(2-fluoro-5-oxo-5-phenylpent-1-en-3-yl)benzoate (3aj). (23.4 mg, 75%). Isolated by column chromatography (petroleum ether: ethyl acetate = 20:1, R_f = 0.3); ^{1}H NMR (600 MHz, $CDCl_3$) δ 7.99 (d, J = 8.2 Hz, 2H), 7.94-7.92 (m, 2H), 7.56 (t, J = 7.4 Hz, 1H), 7.45 (t, J = 7.8 Hz, 2H), 7.43 (d, J = 8.2 Hz, 2H), 4.68 (dd, J = 17.4, 3.2 Hz, 1H), 4.40 (dd, J = 49.8, 3.2 Hz, 1H), 4.40 (dt, J = 17.0, 7.2 Hz, 1H), 3.89 (s, 3H), 3.63 (dd, J = 17.4, 6.6 Hz, 1H), 3.48 (dd, J = 17.4, 6.6 Hz, 1H); ^{13}C NMR (150 MHz, $CDCl_3$) δ 196.5, 166.7, 166.1 (d, $J_{C\text{-}F}$ = 260.0 Hz), 145.1, 136.5, 133.4, 130.0, 129.1, 128.7, 128.0, 127.9, 91.5 (d, $J_{C\text{-}F}$ = 20.2 Hz), 52.0, 43.3 (d, $J_{C\text{-}F}$ = 26.6 Hz), 41.1 (d, $J_{C\text{-}F}$ = 2.2 Hz); ^{19}F NMR (565 MHz, $CDCl_3$) δ -97.2 (1F). HRMS (ESI) calcd for $C_{19}H_{18}FO_3$ [M + H $^+$], 313.1235; found: 313.1237.

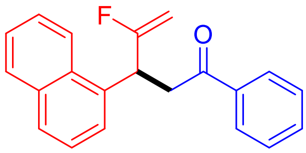

4-Fluoro-3-(naphthalen-1-yl)-1-phenylpent-4-en-1-one (3ak). (28.9 mg, 95%). Isolated by column

chromatography (petroleum ether: ethyl acetate = 20:1, R_f = 0.5); ^{1}H NMR (600 MHz, $CDCl_3$) δ 8.17 (d, J = 8.5 Hz, 1H), 7.94 (d, J = 7.4 Hz, 2H), 7.85 (d, J = 8.2 Hz, 1H), 7.76 (d, J = 8.2 Hz, 1H), 7.54-7.51 (m, 3H), 7.47 (t, J = 7.4 Hz, 1H), 7.43-7.40 (m, 3H), 5.26-5.21 (m, 1H), 4.72 (dd, J = 17.6, 3.2 Hz, 1H), 4.37 (dd, J = 49.8, 3.0 Hz, 1H), 3.78 (dd, J = 17.6, 6.4 Hz, 1H), 3.48 (dd, J = 17.6, 6.4 Hz, 1H); ^{13}C NMR (150 MHz, $CDCl_3$) δ 197.0, 166.5 (d, $J_{C\text{-}F}$ = 260.0 Hz), 136.6, 135.9, 134.1, 133.3, 131.2, 129.0, 128.6, 128.0, 127.9, 126.5, 125.7, 125.4, 124.5, 122.9, 91.9 (d, $J_{C\text{-}F}$ = 20.6 Hz), 41.5 (d, $J_{C\text{-}F}$ = 3.0 Hz), 38.5 (d, $J_{C\text{-}F}$ = 27.0 Hz); ^{19}F NMR (565 MHz, $CDCl_3$) δ -97.2 (1F). HRMS (ESI) calcd for $C_{21}H_{18}FO$ [M + H $^{+}$], 305.1336; found: 305.1338.

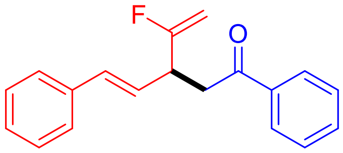

(E)-4-Fluoro-1-phenyl-3-styrylpent-4-en-1-one (3al). (21.8 mg, 78%). Isolated by column chromatography (petroleum ether: ethyl acetate = 20:1, R_f = 0.4); ^{1}H NMR (600 MHz, $CDCl_3$) δ 7.92 (d, J = 8.5 Hz, 1H), 7.54 (t, J = 7.4 Hz, 1H), 7.44 (t, J = 7.6 Hz, 2H), 7.31 (t, J = 7.6 Hz, 2H), 7.26-7.25 (m, 2H), 7.22 (t, J = 7.2 Hz, 2H), 6.26 (dd, J = 15.6, 7.4 Hz, 1H), 5.84 (dd, J = 25.4, 15.6 Hz, 1H), 4.59 (dd, J = 16.4, 2.4 Hz, 1H), 4.33 (dd, J = 48.8, 2.4 Hz, 1H), 4.24-4.21 (m, 1H), 3.43 (dd, J = 7.4, 1.8 Hz, 2H); ^{13}C NMR (150 MHz, $CDCl_3$) δ 197.6, 161.4 (d, $J_{C\text{-}F}$ = 251.6 Hz), 142.5, 137.0, 135.1 (d, $J_{C\text{-}F}$ = 2.8 Hz), 133.1, 128.7, 128.6, 128.0, 127.7, 126.8, 122.0 (d, $J_{C\text{-}F}$ = 24.2 Hz), 92.7 (d, $J_{C\text{-}F}$ = 21.4 Hz), 44.2, 43.1; ^{19}F NMR (565 MHz, $CDCl_3$) δ -111.2 (1F). HRMS (ESI) calcd for $C_{19}H_{18}FO$ [M + H $^{+}$], 281.1336; found: 281.1334.

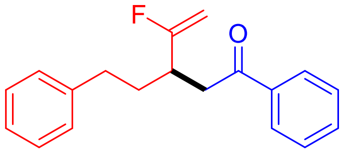

4-Fluoro-3-phenethyl-1-phenylpent-4-en-1-one (3am). (15.8 mg, 56%). Isolated by column chromatography (petroleum ether: ethyl acetate = 20:1, R_f = 0.5); ^{1}H NMR (600 MHz, $CDCl_3$) δ 7.94 (d, J = 7.6 Hz, 2H), 7.57 (t, J = 7.4 Hz, 1H), 7.46 (t, J = 7.3 Hz, 2H), 7.29-7.26 (m, 2H), 7.19-7.16 (m, 3H), 4.62 (dd, J = 17.6, 2.8 Hz, 1H), 4.37 (dd, J = 50.6, 2.8 Hz, 1H), 3.30-3.25 (m, 1H), 3.09-3.00 (m, 2H), 2.78-2.73 (m, 1H), 2.66-2.62 (m, 1H), 1.90-1.79 (m, 2H); ^{13}C NMR (150 MHz, $CDCl_3$) δ 198.0, 166.8 (d, $J_{C\text{-}F}$ = 260.0 Hz), 141.6, 137.0, 133.2, 128.6, 128.4, 128.1, 125.9, 91.4 (d, $J_{C\text{-}F}$ = 20.0 Hz), 41.0, 38.0 (d, $J_{C\text{-}F}$ = 25.6 Hz), 33.4, 33.3; ^{19}F NMR (565 MHz, $CDCl_3$) δ -106.7 (1F). HRMS (ESI) calcd for $C_{19}H_{19}FNaO$ [M + Na $^{+}$], 305.1312; found: 305.1308.

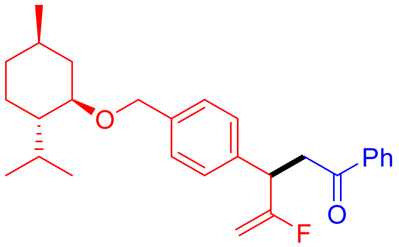

4-Fluoro-3-(4-((((2S,5R)-2-isopropyl-5-methylcyclohexyl)oxy)methyl)phenyl)-1-phenylpent-4-en-1-one (3an). (42.2 mg, 90%, dr = 1:1). Isolated by column chromatography (petroleum ether: ethyl acetate = 20:1, R_f = 0.4); ^{1}H NMR (600 MHz, $CDCl_3$) δ 7.92 (d, J = 7.6 Hz, 2H), 7.54 (t, J = 7.3 Hz, 1H), 7.44 (t, J = 7.6 Hz, 2H), 7.32 (d, J = 8.4 Hz, 2H), 7.30 (d, J = 8.4 Hz, 2H), 4.64-4.60 (m, 2H), 4.38 (dd, J = 49.8, 3.0 Hz, 1H), 4.35-4.30 (m, 2H), 3.61-3.56 (m, 1H), 3.45-3.41 (m, 1H), 3.17-3.13 (m, 1H), 2.30-2.26 (m, 1H), 2.19-2.17 (m, 1H), 1.67-1.60 (m, 2H), 1.38-1.32 (m, 1H), 1.29-1.26 (m, 1H), 0.99-0.82 (m, 9H), 0.68 (t, J = 7.1 Hz, 3H); ^{13}C NMR (150 MHz, $CDCl_3$) δ 196.9, 167.0 (d, J_{C-F} = 259.8 Hz), 139.0, 138.1, 136.6, 133.2, 128.6, 128.2, 128.1, 128.0, 127.8, 90.9 (d, J_{C-F} = 20.2 Hz), 78.7, 70.0, 48.2, 43.1 (d, J_{C-F} = 26.6 Hz), 41.4 (d, J_{C-F} = 2.7 Hz), 40.2, 34.5, 31.5, 25.4, 23.1, 22.3, 21.0, 16.0; ^{19}F NMR (565 MHz, $CDCl_3$) δ -99.0 (0.5F), -99.1 (0.5F). HRMS (APCI) calcd for $C_{28}H_{36}FO_2$ [M + H $^+$], 423.2694; found: 423.2689.

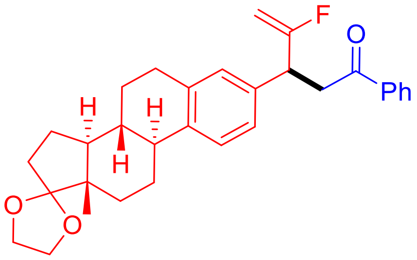

4-Fluoro-3-((8R,9S,13S,14S)-13-methyl-6,7,8,9,11,12,13,14,15,16-decahydrospiro[cyclopenta[a]phenanthrene-17,2'-[1,3]dioxolan]-3-yl)-1-phenylpent-4-en-1-one (3ao). (44.1 mg, 93%). Isolated by column chromatography (petroleum ether: ethyl acetate = 10:1, R_f = 0.3); ^{1}H NMR (600 MHz, $CDCl_3$) δ 7.95 (d, J = 7.6 Hz, 2H), 7.55 (t, J = 7.4 Hz, 1H), 7.44 (t, J = 7.7 Hz, 2H), 7.24 (d, J = 8.0 Hz, 1H), 7.11 (d, J = 8.0 Hz, 1H), 7.05 (s, 1H), 4.62 (dd, J = 17.4, 2.8 Hz, 1H), 4.38 (dd, J = 49.8, 2.8 Hz, 1H), 4.26 (dt, J = 17.4, 7.0 Hz, 1H), 3.96-3.86 (m, 4H), 3.60 (dd, J = 17.3, 7.4 Hz, 1H), 3.39 (dd, J = 17.3, 7.4 Hz, 1H), 2.85-2.84 (m, 2H), 2.33-2.30 (m, 1H), 2.27-2.23 (m, 1H), 2.04-2.00 (m, 1H), 1.90-1.88 (m, 1H), 1.86-1.81 (m, 1H), 1.79-1.73 (m, 2H), 1.65-1.60 (m, 1H), 1.54-1.32 (m, 5H), 0.87 (s, 3H); ^{13}C NMR (150 MHz, $CDCl_3$) δ 197.0, 167.0 (d, J_{C-F} = 260.0 Hz), 139.4, 137.1, 137.0, 136.7, 133.2, 128.6, 128.2, 128.0, 125.6, 124.8, 119.3, 90.8 (d, J_{C-F} = 20.6 Hz), 65.2, 64.5, 49.4, 46.1, 43.9, 42.9 (d, J_{C-F} = 27.0 Hz), 41.5 (d, J_{C-F} = 2.4 Hz), 38.7, 34.2, 30.7, 29.5, 26.9, 25.8, 22.3, 14.3; ^{19}F NMR (565 MHz, $CDCl_3$) δ -99.3 (1F). HRMS (APCI) calcd for $C_{31}H_{36}FO_3$ [M + H $^+$], 475.2643; found: 475.2641.

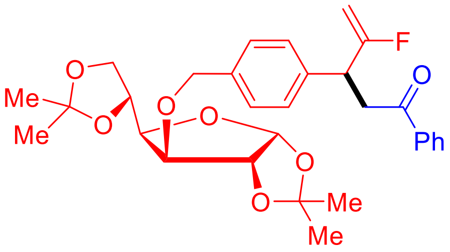

3-(4-((((3aR,5R)-5-((R)-2,2-Dimethyl-1,3-dioxolan-4-yl)-2,2-dimethyltetrahydrofuro[2,3-d][1,3]dioxol-6-yl)oxy)methyl)phenyl)-4-fluoro-1-phenylpent-4-en-1-one (3ap). (49.4 mg, 94%). Isolated by column chromatography (petroleum ether: ethyl acetate = 5:1, R_f = 0.3); ^{1}H NMR (600 MHz, $CDCl_3$) δ 7.94 (d, J = 7.8 Hz, 2H), 7.56 (t, J = 7.2 Hz, 1H), 7.45 (t, J = 7.6 Hz, 2H), 7.33 (d, J = 8.0 Hz, 2H), 7.30 (d, J = 8.0 Hz, 2H), 5.89 (s, 1H), 4.66-4.57 (m, 4H), 4.42-4.30 (m, 3H), 4.14-4.09 (m, 2H), 4.01-3.98 (m, 2H), 3.60 (dd, J = 17.4, 6.8 Hz, 1H), 3.45 (dd, J = 17.4, 6.8 Hz, 1H), 1.49 (s, 3H), 1.42 (d, J = 2.0 Hz, 3H), 1.36 (d, J = 5.8 Hz, 3H), 1.30 (s, 3H); ^{13}C NMR (150 MHz, $CDCl_3$) δ 196.8, 166.8 (d, $J_{C\text{-}F}$ = 260.2 Hz), 139.5, 136.6, 133.3, 128.6, 128.0, 127.9, 111.7, 108.9, 105.2, 91.0 (d, $J_{C\text{-}F}$ = 20.4 Hz), 82.6, 81.6, 81.2, 72.4, 71.9, 67.3, 43.1 (d, $J_{C\text{-}F}$ = 26.8 Hz), 41.3 (d, $J_{C\text{-}F}$ = 2.9 Hz), 26.8, 26.7, 26.2, 25.4; ^{19}F NMR (565 MHz, $CDCl_3$) δ -99.3 (1F). HRMS (APCI) calcd for $C_{30}H_{35}FNaO_7$ [M + Na $^+$], 549.2259; found: 549.2253.

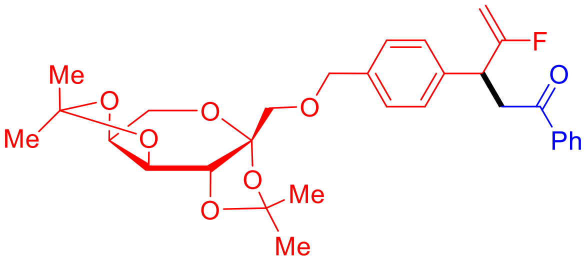

4-Fluoro-1-phenyl-3-(4-((((3aR,5aS,8aS,8bR)-2,2,7,7-tetramethyltetrahydro-3aH-bis([1,3]dioxolo)[4,5-b:4',5'-d]pyran-3a-yl)methoxy)methyl)phenyl)pent-4-en-1-one (3aq). (51 mg, 97%). Isolated by column chromatography (petroleum ether: ethyl acetate = 5:1, R_f = 0.3); ^{1}H NMR (600 MHz, $CDCl_3$) δ 7.93 (d, J = 7.8 Hz, 2H), 7.55 (t, J = 7.4 Hz, 1H), 7.45 (t, J = 7.8 Hz, 2H), 7.31 (d, J = 8.2 Hz, 2H), 7.29 (d, J = 8.2 Hz, 2H), 4.66-4.61 (m, 2H), 4.58 (dd, J = 8.0, 2.2 Hz, 1H), 4.55 (dd, J = 12.4, 2.4 Hz, 1H), 4.42-4.41 (m, 1H), 4.40-4.30 (m, 2H), 4.22 (d, J = 8.0 Hz, 1H), 3.91 (dd, J = 13.0, 1.6 Hz, 1H), 3.72 (d, J = 13.0 Hz, 1H), 3.62-3.56 (m, 3H), 3.43 (dd, J = 17.4, 4.2 Hz, 1H), 1.54 (s, 3H), 1.41 (s, 3H), 1.39 (s, 3H), 1.31 (s, 3H); ^{13}C NMR (150 MHz, $CDCl_3$) δ 196.9, 166.9 (d, $J_{C\text{-}F}$ = 260.2 Hz), 139.2, 137.1, 136.6, 133.2, 128.6, 128.0, 127.8, 127.7, 108.8, 108.5, 102.6, 91.0 (d, $J_{C\text{-}F}$ = 20.4 Hz), 73.3, 73.2, 71.5, 71.4, 70.9, 70.1, 70.0, 60.9, 43.1 (d, $J_{C\text{-}F}$ = 26.8 Hz), 41.4 (d, $J_{C\text{-}F}$ = 2.9 Hz), 26.5, 25.7, 25.4, 23.9; ^{19}F NMR (565 MHz, $CDCl_3$) δ -99.2 (1F). HRMS (APCI) calcd for $C_{30}H_{35}FNaO_7$ [M + Na $^+$], 549.2259;

found: 549.2254.

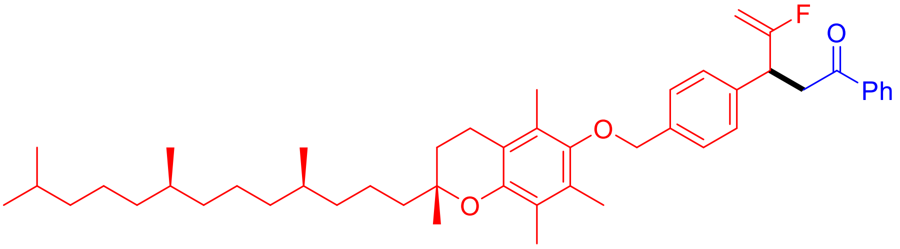

4-Fluoro-1-phenyl-3-(4-((((R)-2,5,7,8-tetramethyl-2-((4R,8R)-4,8,12-trimethyltridecyl)chroman-6-yl)oxy)methyl)phenyl)pent-4-en-1-one (3ar). (61.2 mg, 88%). Isolated by column chromatography (petroleum ether: ethyl acetate = 5:1, R_f = 0.6); ^{1}H NMR (600 MHz, $CDCl_3$) δ 7.94 (d, J = 7.6 Hz, 2H), 7.55 (t, J = 7.4 Hz, 1H), 7.46-7.43 (m, 4H), 7.38 (d, J = 7.8 Hz, 2H), 4.65 (s, 2H), 4.64 (dd, J = 17.4, 3.0 Hz, 1H), 4.38 (dd, J = 49.8, 3.0 Hz, 1H), 4.36 (dt, J = 17.4, 7.0 Hz, 1H), 3.61 (dd, J = 17.4, 7.0 Hz, 1H), 3.47 (dd, J = 17.4, 7.0 Hz, 1H), 2.57 (t, J = 6.7 Hz, 2H), 2.20 (s, 3H), 2.15 (s, 3H), 2.10 (s, 3H), 1.84-1.79 (m, 1H), 1.77-1.73 (m, 1H), 1.61-1.49 (m, 3H), 1.47-1.24 (m, 15H), 1.20-1.07 (m, 6H), 0.87 (s, 3H), 0.86 (s, 3H), 0.85 (s, 3H), 0.84 (s, 3H); ^{13}C NMR (150 MHz, $CDCl_3$) δ 196.9, 167.0 (d, $J_{C\text{-}F}$ = 260.2 Hz), 148.1, 148.0, 139.5, 137.1, 136.8, 133.4, 128.7, 128.1, 128.0, 127.9, 126.0, 123.0, 117.6, 91.1 (d, $J_{C\text{-}F}$ = 20.2 Hz), 74.9, 74.4, 43.3 (d, $J_{C\text{-}F}$ = 26.7 Hz), 41.5 (d, $J_{C\text{-}F}$ = 2.2 Hz), 40.1, 40.0, 39.4, 37.6, 37.5, 37.4, 37.3, 32.9, 32.8, 32.7, 31.4, 31.3, 28.0, 24.9, 24.5, 24.0, 22.8, 22.7, 21.1, 20.7, 19.9, 19.8, 19.7, 12.9, 12.0, 11.9; ^{19}F NMR (565 MHz, $CDCl_3$) δ -99.1 (1F). HRMS (APCI) calcd for $C_{47}H_{65}FNaO_3$ [M + Na $^+$], 719.4810; found: 719.4800.

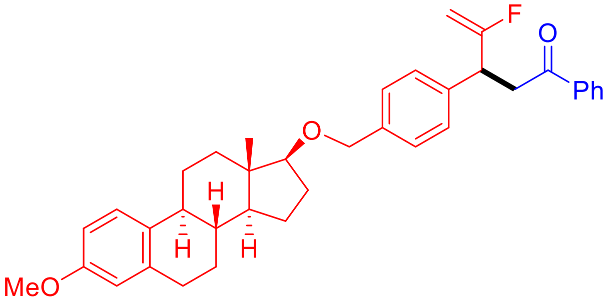

4-Fluoro-3-(4-(((((8R,9S,13S,14S,17S)-3-methoxy-13-methyl-7,8,9,11,12,13,14,15,16,17-decahydro-6H-cyclopenta[a]phenanthren-17-yl)oxy)methyl)phenyl)-1-phenylpent-4-en-1-one (3as). (52.5 mg, 95%). Isolated by column chromatography (petroleum ether: ethyl acetate = 10:1, R_f = 0.5); ^{1}H NMR (600 MHz, $CDCl_3$) δ 7.93 (d, J = 7.6 Hz, 2H), 7.55 (t, J = 7.3 Hz, 1H), 7.44 (t, J = 7.3 Hz, 2H), 7.32 (d, J = 8.2 Hz, 2H), 7.30 (d, J = 8.2 Hz, 2H), 7.19 (d, J = 8.3 Hz, 1H), 6.70 (dd, J = 8.6, 2.0 Hz, 1H), 6.62 (d, J = 1.6 Hz, 1H), 4.63 (dd, J = 17.4, 2.8 Hz, 1H), 4.53 (s, 2H), 4.38 (dd, J = 17.4, 2.8 Hz, 1H), 4.33 (dt, J = 15.8, 7.2 Hz, 1H), 3.76 (s, 3H), 3.59 (dd, J = 17.4, 6.8 Hz, 1H), 3.48 (t, J = 8.3 Hz, 1H), 3.44 (dd, J = 17.4, 6.8 Hz, 1H), 2.88-2.79 (m, 2H), 2.28-2.25 (m, 1H), 2.18-2.14 (m,

1H), 2.06-2.00 (m, 2H), 1.87-1.84 (m, 1H), 1.68-1.64 (m, 1H), 1.61-1.28 (m, 6H), 2.00-1.15 (m, 1H), 0.85 (s, 3H); ^{13}C NMR (150 MHz, $CDCl_3$) δ 196.9, 167.0 (d, J_{C-F} = 260.0 Hz), 157.4, 138.9, 138.3, 137.9, 136.7, 133.2, 132.6, 128.6, 128.0, 127.7, 127.6, 126.3, 113.7, 111.4, 91.0 (d, J_{C-F} = 19.8 Hz), 88.4, 71.3, 55.1, 50.2, 43.9, 43.4, 43.1 (d, J_{C-F} = 26.4 Hz), 41.4 (d, J_{C-F} = 2.4 Hz), 38.6, 37.9, 29.8, 28.0, 27.2, 26.4, 23.1, 11.8; ^{19}F NMR (565 MHz, $CDCl_3$) δ -98.6 (1F). HRMS (ESI) calcd for $C_{37}H_{41}FNaO_3$ [M + Na $^+$], 575.2932; found: 575.2938.

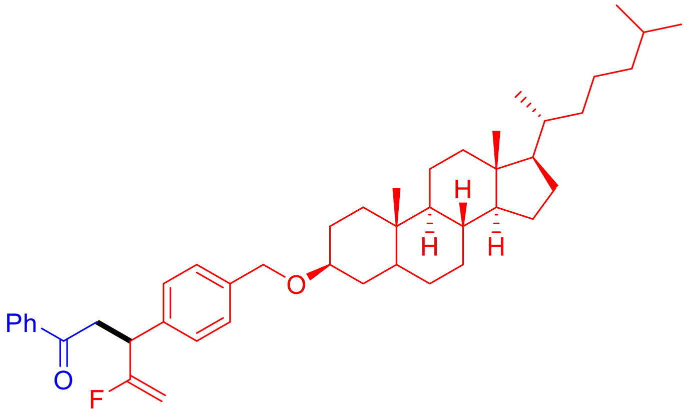

3-(4-((((3S,8R,9S,10S,13R,14S,17R)-10,13-Dimethyl-17-((R)-6-methylheptan-2-yl)hexadecahydro-1H-cyclopenta[a]phenanthren-3-yl)oxy)methyl)phenyl)-4-fluoro-1-phenylpent-4-en-1-one (3at). (60.2 mg, 92%). Isolated by column chromatography (petroleum ether: ethyl acetate = 10:1, R_f = 0.6); ^{1}H NMR (600 MHz, $CDCl_3$) δ 7.93 (d, J = 7.6 Hz, 2H), 7.55 (t, J = 7.4 Hz, 1H), 7.44 (t, J = 7.7 Hz, 2H), 7.31 (d, J = 8.4 Hz, 2H), 7.29 (d, J = 8.4 Hz, 2H), 4.62 (dd, J = 17.4, 3.0 Hz, 1H), 4.52 (d, J = 12.4 Hz, 1H), 4.49 (d, J = 12.4 Hz, 1H), 4.36 (dd, J = 49.9, 3.0 Hz, 1H), 4.35-4.29 (m, 1H), 3.59 (dd, J = 17.3, 6.9 Hz, 1H), 3.43 (dd, J = 17.3, 6.9 Hz, 1H), 3.34-3.29 (m, 1H), 1.97-1.94 (m, 1H), 1.90-1.88 (m, 1H), 1.83-1.77 (m, 1H), 1.72-1.63 (m, 3H), 1.57-1.40 (m, 4H), 1.37-1.19 (m, 9H), 1.14-0.93 (m, 9H), 0.90-0.83 (m, 11H), 0.80 (s, 3H), 0.64 (s, 3H), 0.62-0.57 (m, 1H); ^{13}C NMR (150 MHz, $CDCl_3$) δ 196.9, 167.0 (d, J_{C-F} = 260.2 Hz), 139.0, 138.2, 136.7, 133.2, 128.6, 128.0, 127.9, 127.8, 91.0 (d, J_{C-F} = 20.4 Hz), 78.1, 69.4, 56.5, 56.2, 44.8, 43.1 (d, J_{C-F} = 27.0 Hz), 42.6, 41.4 (d, J_{C-F} = 2.4 Hz), 40.0, 39.5, 37.0, 36.1, 35.8, 35.7, 35.4, 34.8, 32.1, 28.8, 28.2, 28.0, 24.2, 23.8, 22.8, 22.5, 21.2, 18.6, 12.3, 12.0; ^{19}F NMR (565 MHz, $CDCl_3$) δ -99.2 (1F). HRMS (APCI) calcd for $C_{45}H_{64}FO_2$ [M + H $^+$], 655.4885; found: 655.4870.

4. Scale up synthesis of product 3a

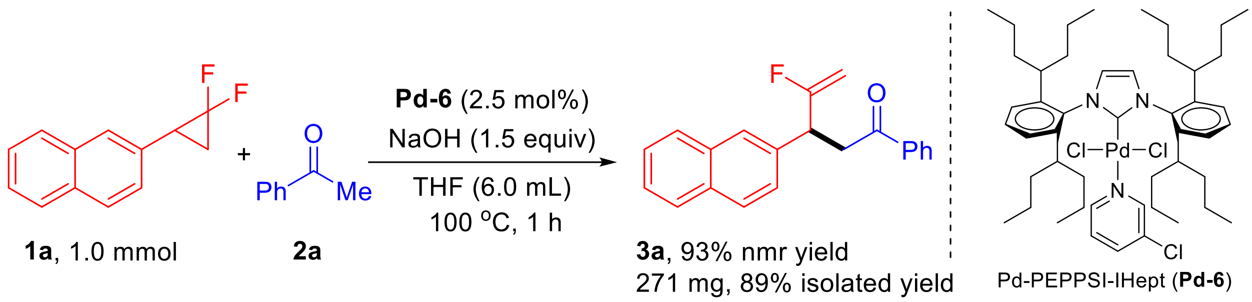

A flame-dried reaction tube (50 cm^3) equipped with a magnetic stir bar was charged with Pd-PEPPSI-IHept (22.6 mg, 2.5 mol%). The reaction tube was transferred to the glovebox and charged with **1a** (1.0 mmol), **2a** (2.0 mmol), NaOH (60.0 mg) and THF (6.0 mL). After that, the reaction mixture was sealed with Teflon cap, moved out of glovebox, and stirred at 100 °C for 1 h. After the mixture was cooled to room temperature, the resulting solution was directly filtered through a pad of silica gel by EtOAc (10.0 mL). The solvent was evaporated *in vacuo* to give the crude product. NMR yield (93%) was determined by ^{1}H NMR using CH_2Br_2 as an internal standard. The residue was purified by column chromotography on silica gel to give the pure product **3a** (271 mg, 89% isolated yield).

5. Optimization of the reaction conditions for furan product

Table S1. Optimization of the reaction conditions[a]

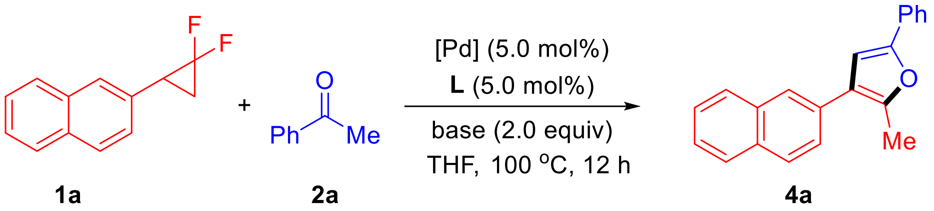

entry	[Pd]	L	base	yield **4a** (%)[b]
1	$Pd(OAc)_2$	XanPhos	KOH	trace
2	$Pd(OAc)_2$	RuPhos	KOH	N.D.
3	$Pd(OAc)_2$	DPEPhos	KOH	N.D.
4[c]	$Pd(OAc)_2$	PPh_3	KOH	N.D.
5	$Pd(OAc)_2$	dppe	KOH	N.D.
6	$Pd(OAc)_2$	dppf	KOH	N.D.
7	$Pd(OAc)_2$	dtbbpy	KOH	N.D.
8	$Pd(OAc)_2$	IMes•HCl	KOH	trace
9	$Pd(OAc)_2$	SIMes•HCl	KOH	trace
10	$Pd(OAc)_2$	ICy•HCl	KOH	N.D.
11	$Pd(OAc)_2$	IPr•HCl	KOH	54

12	$Pd(OAc)_2$	SIPr•HCl	KOH	75
13	Pd-PEPPSI-SIPr	-	KOH	75
14	Pd-PEPPSI-SIPr	-	NaOH	67
15	Pd-PEPPSI-SIPr	-	NaOMe	58
16	Pd-PEPPSI-SIPr	-	LiO^{t}Bu	N.D.
17	Pd-PEPPSI-SIPr	-	DBU	N.D.
18	Pd-PEPPSI-SIPr	-	K_3PO_4	N.D.
19	Pd-PEPPSI-SIPr	-	K_2CO_3	N.D.
20	Pd-PEPPSI-SIPr	-	Cs_2CO_3	N.D.
21	$Pd(OAc)_2$	-	KOH	N.D.

[a]Reaction conditions: **1a** (0.1 mmol), **2a** (0.2 mmol), palladium catalyst (5.0 mol%), ligand (5.0 mol%), base (0.2 mmol), THF (1.0 mL), 100 °C, 12 h under N_2 unless otherwise noted. [b]NMR yields were based on **1a** and determined by ^1H NMR using CH_2Br_2 as an internal standard. [c]ligand (10%). N.D. = not detected.

6. General procedure and characterization data for product 4

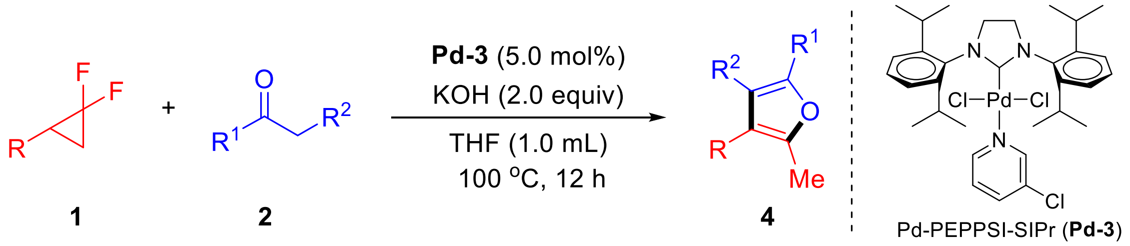

A flame-dried reaction tube (10 cm^3) equipped with a magnetic stir bar was charged with Pd-PEPPSI-SIPr (3.4 mg, 5 mol%). The reaction tube was transferred to the glovebox and charged with **1** (0.1 mmol), **2** (0.2 mmol), KOH (11.2 mg) and THF (1.0 mL). After that, the reaction mixture was sealed with aluminum cap, moved out of glovebox, and stirred at 100 °C for 12 h. After the mixture was cooled to room temperature, the resulting solution was directly filtered through a pad of silica gel by EtOAc (3.0 mL). The solvent was evaporated *in vacuo* to give the crude product. The crude mixture was analyzed by GC-MS. NMR yield was determined by ^1H NMR using CH_2Br_2 as an internal standard. The residue was purified by column chromotography on silica gel to give the pure product **4**. **Note**: Use of the glovebox is not necessary. The reaction can be successfully performed with standard Schlenk line procedure.

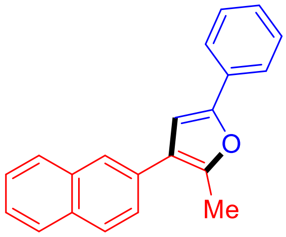

2-Methyl-3-(naphthalen-2-yl)-5-phenylfuran (4a). (21 mg, 73%). Isolated by column chromatography (petroleum ether, R_f = 0.5); ^{1}H NMR (600 MHz, $CDCl_3$) δ 7.93-7.88 (m, 4H), 7.76-7.74 (m, 2H), 7.63 (dd, J = 8.4, 1.7 Hz, 1H), 7.56-7.48 (m, 2H), 7.45-7.42 (m, 2H), 7.31-7.28 (m, 1H), 6.93 (s, 1H), 2.63 (s, 3H); ^{13}C NMR (150 MHz, $CDCl_3$) δ 151.8, 148.0, 133.6, 132.1, 131.5, 130.9, 128.7, 128.2, 127.8, 127.7, 127.1, 126.3, 126.1, 125.9, 125.7, 123.5, 123.1, 106.6, 13.4. HRMS (APCI) calcd for $C_{21}H_{17}O$ [M + H$^+$], 285.1274; found: 285.1270.

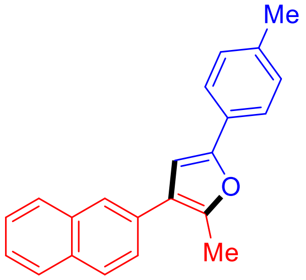

2-Methyl-3-(naphthalen-2-yl)-5-(p-tolyl)furan (4b). (21 mg, 72%). Isolated by column chromatography (petroleum ether, R_f = 0.5); ^{1}H NMR (600 MHz, $CDCl_3$) δ 7.87-7.83 (m, 4H), 7.60-7.57 (m, 3H), 7.50-7.44 (m, 2H), 7.20 (d, J = 8.0 Hz, 2H), 6.83 (s, 1H), 2.58 (s, 3H), 2.36 (s, 3H); ^{13}C NMR (150 MHz, $CDCl_3$) δ 152.0, 147.5, 136.9, 133.6, 132.1, 131.6, 129.3, 128.2, 128.1, 127.8, 127.7, 126.2, 126.1, 125.8, 125.6, 123.4, 122.9, 105.8, 21.3, 13.3; HRMS (APCI) calcd for $C_{22}H_{19}O$ [M + H$^+$], 299.1430; found: 299.1425.

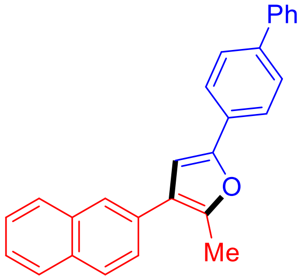

5-([1,1'-Biphenyl]-4-yl)-2-methyl-3-(naphthalen-2-yl)furan (4c). (25 mg, 69%). Isolated by column chromatography (petroleum ether: ethyl acetate = 60:1, R_f = 0.4); ^{1}H NMR (600 MHz, $CDCl_3$) δ 7.94-7.88 (m, 4H), 7.81 (d, J = 8.4 Hz, 2H), 7.69-7.66 (m, 4H), 7.64 (dd, J = 8.4, 1.8 Hz, 1H), 7.56-7.47 (m, 4H), 7.40-7.36 (m, 1H), 6.97 (s, 1H), 2.65 (s, 3H); ^{13}C NMR (150 MHz, $CDCl_3$) δ 151.6, 148.2, 140.7, 139.7, 133.6, 132.2, 131.5, 129.8, 128.8, 128.2, 127.8, 127.7, 127.4, 127.3, 126.9, 126.3, 126.1, 125.9, 125.7, 123.9, 123.2, 106.8, 13.4; HRMS (APCI) calcd for $C_{27}H_{21}O$ [M + H$^+$], 361.1587; found: 361.1582.

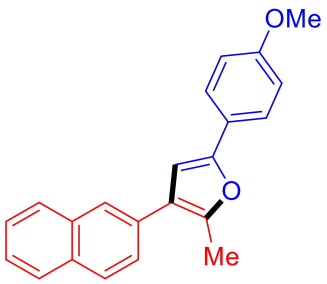

5-(4-Methoxyphenyl)-2-methyl-3-(naphthalen-2-yl)furan (4d). (22 mg, 70%). Isolated by column chromatography (petroleum ether: ethyl acetate = 100:1, R_f = 0.4); ^{1}H NMR (600 MHz, $CDCl_3$) δ 7.92-7.87 (m, 4H); 7.67 (d, J = 8.8 Hz, 2H), 7.62 (dd, J = 8.4, 1.7 Hz, 1H), 7.54-7.48 (m, 2H), 6.97 (d, J = 8.8 Hz, 2H), 6.79 (s, 1H), 3.88 (s, 3H), 2.61 (s, 3H); ^{13}C NMR (150 MHz, $CDCl_3$) δ 158.9, 151.8, 147.2, 133.6, 132.1, 131.7, 128.2, 127.8, 127.7, 126.3, 126.1, 125.8, 125.6, 124.9, 124.0, 122.9, 114.2, 105.0, 55.4, 13.3. HRMS (APCI) calcd for $C_{22}H_{19}O_2$ [M + H$^+$], 315.1380; found: 315.1379.

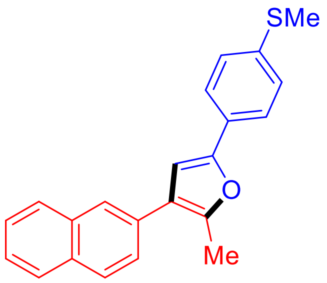

2-Methyl-5-(4-(methylthio)phenyl)-3-(naphthalen-2-yl)furan (4e). (22 mg, 68%). Isolated by column chromatography (petroleum ether: ethyl acetate = 100:1, R_f = 0.4); ^{1}H NMR (600 MHz, $CDCl_3$) δ 7.91 (d, J = 8.6 Hz, 1H), 7.90-7.87 (m, 3H), 7.65 (d, J = 8.6 Hz, 2H), 7.61 (dd, J = 8.4, 1.7 Hz, 1H), 7.54-7.48 (m, 2H), 7.32 (d, J = 8.6 Hz, 2H), 6.88 (s, 1H), 2.62 (s, 3H), 2.55 (s, 3H); ^{13}C NMR (150 MHz, $CDCl_3$) δ 151.5, 147.8, 137.1, 133.6, 132.1, 131.5, 128.2, 127.9, 127.8, 127.7, 126.9, 126.3, 126.1, 125.9, 125.7, 123.9, 123.1, 106.3, 15.9, 13.4. HRMS (APCI) calcd for $C_{22}H_{19}OS$ [M + H$^+$], 331.1151; found: 331.1157.

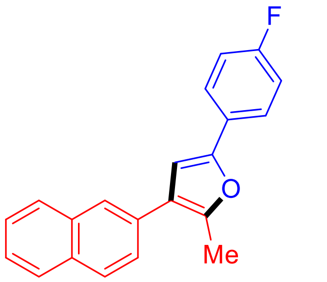

5-(4-Fluorophenyl)-2-methyl-3-(naphthalen-2-yl)furan (4f). (20 mg, 66%). Isolated by column chromatography (petroleum ether: ethyl acetate = 60:1, R_f = 0.4); ^{1}H NMR (600 MHz, $CDCl_3$) δ 7.93-7.88 (m, 4H), 7.72-7.68 (m, 2H), 7.61 (dd, J = 8.4, 1.6 Hz, 1H), 7.55-7.49 (m, 2H), 7.12 (t, J = 8.6 Hz, 2H), 6.85 (s, 1H), 2.62 (s, 3H); ^{13}C NMR

(150 MHz, $CDCl_3$) δ 162.0 (d, J_{C-F} = 246.4 Hz), 151.0, 147.9, 133.6, 132.1, 131.4, 128.2, 127.8, 127.7, 127.2 (d, J_{C-F} = 3.0 Hz), 126.3, 126.0, 125.9, 125.7, 125.2, 125.1, 123.1, 115.7 (d, J_{C-F} = 21.8 Hz), 106.2, 13.3; HRMS (APCI) calcd for $C_{21}H_{16}FO$ [M + H^+], 303.1180; found: 303.1179.

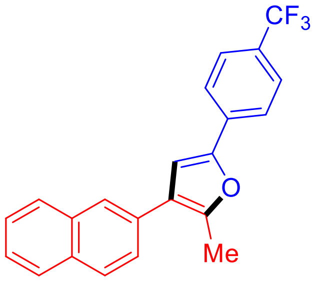

2-Methyl-3-(naphthalen-2-yl)-5-(4-(trifluoromethyl)phenyl)furan (4g). (22 mg, 63%). Isolated by column chromatography (petroleum ether: ethyl acetate = 60:1, R_f = 0.4); ^1H NMR (600 MHz, $CDCl_3$) δ 7.94-7.88 (m, 4H), 7.82 (d, J = 8.4 Hz, 2H), 7.67 (d, J = 8.4 Hz, 2H), 7.61 (dd, J = 8.4, 1.6 Hz, 1H), 7.56-7.50 (m, 2H), 7.04 (s, 1H), 2.64 (s, 3H); ^{13}C NMR (150 MHz, $CDCl_3$) δ 150.3, 149.2, 133.9, 133.6, 132.2, 131.0, 128.5 (q, J_{C-F} = 34.0 Hz), 128.3, 127.8, 127.7, 126.4, 126.0, 125.9, 125.8, 125.7 (q, J_{C-F} = 3.4 Hz), 124.2 (q, J_{C-F} = 272.0 Hz), 122.9, 108.6, 13.4; HRMS (ESI) calcd for $C_{22}H_{16}F_3O$ [M + H^+], 353.1148; found: 353.1144.

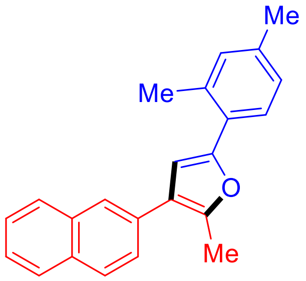

5-(2,4-Dimethylphenyl)-2-methyl-3-(naphthalen-2-yl)furan (4h). (22 mg, 71%). Isolated by column chromatography (petroleum ether, R_f = 0.5); ^1H NMR (600 MHz, $CDCl_3$) δ 7.93-7.88 (m, 4H), 7.69 (d, J = 8.6 Hz, 1H), 7.64 (dd, J = 8.6, 1.8 Hz, 1H), 7.55-7.48 (m, 2H), 7.13-7.11 (m, 2H), 6.75 (s, 1H), 2.63 (s, 3H), 2.57 (s, 3H), 2.39 (s, 3H); ^{13}C NMR (150 MHz, $CDCl_3$) δ 151.5, 147.1, 137.0, 134.2, 133.7, 132.1, 131.9, 131.8, 128.2, 127.8, 127.7, 127.4, 126.8, 126.7, 126.3, 126.2, 125.8, 125.6, 122.7, 109.6, 21.9, 21.1, 13.3. HRMS (APCI) calcd for $C_{23}H_{21}O$ [M + H^+], 313.1587; found: 313.1584.

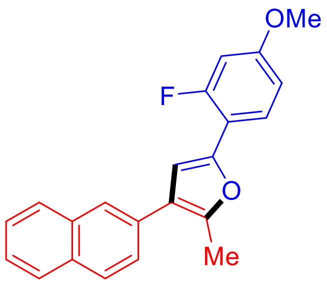

5-(2-Fluoro-4-methoxyphenyl)-2-methyl-3-(naphthalen-2-yl)furan (4i). (24 mg, 72%). Isolated by column chromatography (petroleum ether: ethyl acetate = 60:1, R_f = 0.6); ^{1}H NMR (600 MHz, $CDCl_3$) δ 7.92-7.87 (m, 4H), 7.79 (t, J = 8.8 Hz, 1H), 7.63 (dd, J = 8.6, 1.5 Hz, 1H), 7.55-7.47 (m, 2H), 6.98 (d, J = 3.6 Hz, 1H), 6.80 (dd, J = 8.6, 2.6 Hz, 1H), 6.74 (dd, J = 12.8, 2.6 Hz, 1H), 3.87 (s, 3H), 2.62 (s, 3H); ^{13}C NMR (150 MHz, $CDCl_3$) δ 159.1 (d, $J_{C\text{-}F}$ = 250.4 Hz), 159.6 (d, $J_{C\text{-}F}$ = 10.8 Hz), 147.1, 146.0 (d, $J_{C\text{-}F}$ = 3.2 Hz), 133.6, 131.8 (d, $J_{C\text{-}F}$ = 54.8 Hz), 128.2, 127.8, 127.7, 126.4 (d, $J_{C\text{-}F}$ = 5.2 Hz), 126.2, 126.1, 125.9, 125.6, 123.1, 112.1 (d, $J_{C\text{-}F}$ = 12.8 Hz), 110.2 (d, $J_{C\text{-}F}$ = 2.8 Hz), 109.7 (d, $J_{C\text{-}F}$ = 10.8 Hz), 102.1 (d, $J_{C\text{-}F}$ = 25.1 Hz), 55.6, 13.3; ^{19}F NMR (565 MHz, $CDCl_3$) δ -112.0. HRMS (APCI) calcd for $C_{22}H_{18}FO_2$ [M + H$^+$], 333.1285; found: 333.1291.

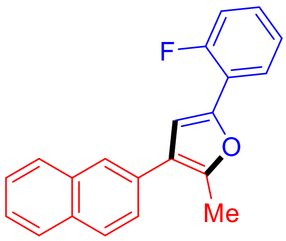

5-(2-Fluorophenyl)-2-methyl-3-(naphthalen-2-yl)furan (4j). (21 mg, 68%). Isolated by column chromatography (petroleum ether, R_f = 0.6); ^{1}H NMR (600 MHz, $CDCl_3$) δ 7.93-7.88 (m, 5H), 7.64 (dd, J = 8.6, 1.6 Hz, 1H), 7.55-7.48 (m, 2H), 7.27-7.21 (m, 2H), 7.19-7.14 (m, 1H), 7.13-7.12 (m, 1H), 2.64 (s, 3H); ^{13}C NMR (150 MHz, $CDCl_3$) δ 158.4 (d, $J_{C\text{-}F}$ = 250.0 Hz), 147.9 (d, $J_{C\text{-}F}$ = 1.0 Hz), 145.9 (d, $J_{C\text{-}F}$ = 3.0 Hz), 133.6, 132.2, 131.4, 128.2, 127.9 (d, $J_{C\text{-}F}$ = 8.2 Hz), 127.8, 127.7, 126.3, 126.1, 125.9, 125.7, 125.6 (d, $J_{C\text{-}F}$ = 3.4 Hz), 124.3 (d, $J_{C\text{-}F}$ = 3.4 Hz), 123.4 (d, $J_{C\text{-}F}$ = 1.6 Hz), 119.1 (d, $J_{C\text{-}F}$ = 12.1 Hz), 115.9 (d, $J_{C\text{-}F}$ = 21.5 Hz), 111.8 (d, $J_{C\text{-}F}$ = 11.6 Hz), 13.3; ^{19}F NMR (565 MHz, $CDCl_3$) δ -114.1. HRMS (APCI) calcd for $C_{21}H_{16}FO$ [M + H$^+$], 303.1180; found: 303.1170.

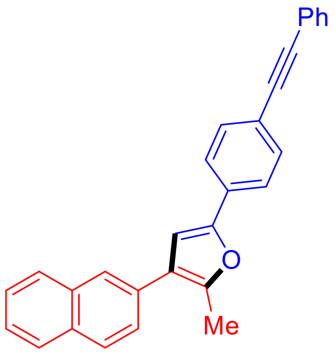

2-Methyl-3-(naphthalen-2-yl)-5-(4-(phenylethynyl)phenyl)furan (4k). (23 mg, 61%). Isolated by column chromatography (petroleum ether: ethyl acetate = 100:1, R_f = 0.4); ^{1}H NMR (600 MHz, $CDCl_3$) δ 7.92 (d, J = 8.6 Hz, 1H), 7.90-7.88 (m, 3H), 7.72 (d, J = 8.6 Hz, 2H), 7.63-7.56 (m, 5H), 7.54-7.49 (m, 2H), 7.41-7.36 (m, 3H), 6.97 (s, 1H), 2.63 (s, 3H); ^{13}C NMR (150 MHz, $CDCl_3$) δ 151.2, 148.5, 133.6, 132.2, 132.0, 131.6, 131.3, 130.5, 128.4, 128.3, 127.8, 127.7, 126.3, 126.1, 126.0, 125.8, 123.4, 123.3, 123.2, 121.6, 107.6, 90.2, 89.6, 13.4. HRMS (APCI) calcd for $C_{29}H_{21}O$ [M + H$^+$], 385.1587; found: 385.1588.

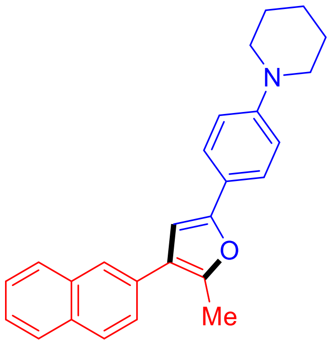

1-(4-(5-Methyl-4-(naphthalen-2-yl)furan-2-yl)phenyl)piperidine (4l). (19 mg, 52%). Isolated by column chromatography (petroleum ether: ethyl acetate = 60:1, R_f = 0.4); ^{1}H NMR (600 MHz, $CDCl_3$) δ 7.90-7.86 (m, 4H), 7.63-7.61 (m, 3H), 7.53-7.47 (m, 2H), 7.00 (br, 2H), 7.76 (s, 1H), 3.24 (t, J = 5.6 Hz, 4H), 2.61 (s, 3H), 1.78-1.73 (m, 4H), 1.65-1.61 (m, 2H); ^{13}C NMR (150 MHz, $CDCl_3$) δ 152.3, 146.8, 133.6, 132.0, 131.9, 128.1, 127.8, 127.7, 126.2, 126.1, 125.8, 125.6, 124.5, 122.8, 116.3, 104.4, 50.4, 25.7, 24.3, 13.3. HRMS (ESI) calcd for $C_{26}H_{26}NO$ [M + H$^+$], 368.2009; found: 368.2022.

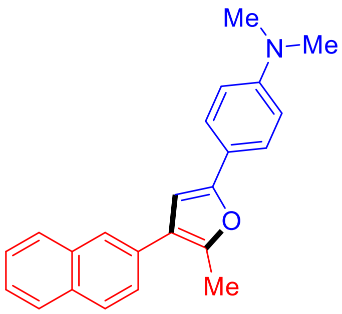

***N,N*-Dimethyl-4-(5-methyl-4-(naphthalen-2-yl)furan-2-yl)aniline (4m).** (17 mg, 53%). Isolated by column chromatography (petroleum ether:ethyl acetate = 20:1, R_f = 0.5); ^{1}H NMR (600 MHz, $CDCl_3$) δ 7.87-7.83 (m, 4H), 7.60-7.58 (m, 3H), 7.50-7.44 (m, 2H), 6.76 (d, J = 8.8 Hz, 2H), 6.69 (s, 1H), 2.99 (s, 6H), 2.58 (s, 3H); ^{13}C NMR (150 MHz, $CDCl_3$) δ 152.6, 149.7, 146.4, 133.6, 132.0, 131.9, 128.0, 127.8, 127.6, 126.2, 125.7, 125.5, 124.7, 122.7, 119.8, 112.5, 103.6, 40.5, 13.3; HRMS (APCI) calcd for $C_{23}H_{22}NO$ [M + H$^+$], 328.1696; found: 328.1692.

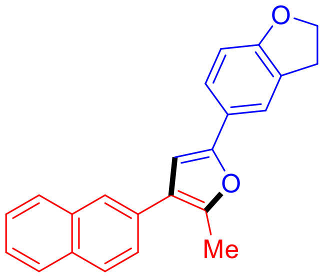

6-(5-Methyl-4-(naphthalen-2-yl)furan-2-yl)-2,3-dihydrobenzofuran (4n). (17 mg, 51%). Isolated by column chromatography (petroleum ether:ethyl acetate = 30:1, R_f = 0.5); ^{1}H NMR (600 MHz, $CDCl_3$) δ 7.88-7.84 (m, 4H), 7.58 (d, J = 8.4 Hz, 1H), 7.56 (s, 1H), 7.50-7.45 (m, 3H), 6.82 (d, J = 8.2 Hz, 1H), 6.72 (s, 1H), 4.60 (t, J = 8.6 Hz, 2H), 3.25 (t, J = 8.6 Hz, 2H), 2.58 (s, 3H); ^{13}C NMR (150 MHz, $CDCl_3$) δ 159.5, 152.2, 146.9, 133.6, 132.0, 131.7, 128.1, 127.8, 127.6, 127.5, 126.2, 126.1, 125.8, 125.6, 124.0, 123.9, 122.8, 120.5, 109.4, 104.6, 71.4, 29.7, 13.3; HRMS (ESI) calcd for $C_{23}H_{19}O_2$ [M + H$^+$], 327.1380; found: 327.1375.

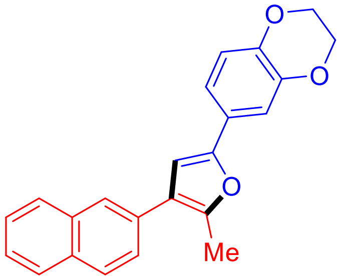

6-(5-Methyl-4-(naphthalen-2-yl)furan-2-yl)-2,3-dihydrobenzo[b][1,4]dioxine (4o) (18 mg, 53%). Isolated by column chromatography (petroleum ether:ethyl acetate = 30:1, R_f = 0.5); ^{1}H NMR (600 MHz, $CDCl_3$) δ 7.87-7.82 (m, 4H), 7.57 (dd, J = 8.4, 1.4 Hz, 1H), 7.49-7.44 (m, 2H), 7.23-7.18 (m, 2H), 6.88 (d, J = 8.4 Hz, 1H), 6.73 (s, 1H), 4.27 (s, 4H), 2.56 (s, 3H); ^{13}C NMR (150 MHz, $CDCl_3$) δ 151.5, 147.3, 143.7, 142.9, 133.6, 132.0, 131.6, 128.1, 127.8, 127.6, 126.2, 126.1, 125.8, 125.6, 124.8, 122.9, 117.5, 117.0, 112.5, 105.4, 64.5, 64.4, 13.3; HRMS (APCI) calcd for $C_{23}H_{19}O_3$ [M + H$^+$], 343.1329; found: 343.1322.

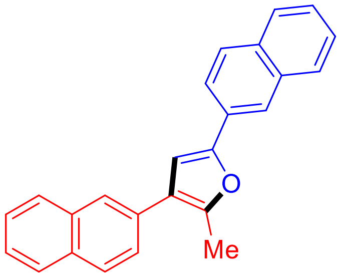

2-Methyl-3,5-di(naphthalen-2-yl)furan (4p). (21 mg, 63%). Isolated by column chromatography (petroleum ether: ethyl acetate = 60:1, R_f = 0.4); ^{1}H NMR (600 MHz, $CDCl_3$) δ 8.21 (s, 1H), 7.95-7.83 (m, 8H), 7.66 (dd, J = 8.5, 1.6 Hz, 1H), 7.57-7.47 (m, 4H), 7.05 (s, 1H), 2.68 (s, 3H); ^{13}C NMR (150 MHz, $CDCl_3$) δ 151.9, 148.3, 133.6, 132.6, 132.2, 131.5, 128.4, 128.2, 128.1, 127.8, 127.7, 126.5, 126.3, 126.1, 125.9, 125.8, 125.7, 123.3, 122.2, 121.6, 107.3, 13.4; HRMS (APCI) calcd for $C_{25}H_{19}O$ [M + H$^+$], 335.1430; found: 335.1427.

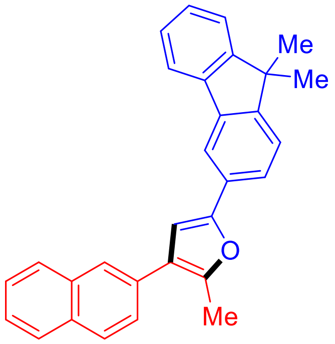

5-(9,9-Dimethyl-9H-fluoren-3-yl)-2-methyl-3-(naphthalen-2-yl)furan (4q). (26 mg, 65%). Isolated by column chromatography (petroleum ether, R_f = 0.4); ^{1}H NMR (600 MHz, $CDCl_3$) δ 7.88-7.84 (m, 4H), 7.78 (s, 1H), 7.73-7.68 (m, 3H), 7.60 (dd, J = 8.4, 1.4 Hz, 1H), 7.50-7.48 (m, 1H), 7.47-7.43 (m, 2H), 7.34-7.29 (m, 2H), 6.94 (s, 1H), 2.62 (s, 3H), 1.54 (s, 6H); ^{13}C NMR (150 MHz, $CDCl_3$) δ 154.1, 153.8, 152.3, 147.8, 138.9, 138.2, 133.6, 132.1, 131.5, 129.8, 128.2, 127.8, 127.7, 127.2, 127.0, 126.3, 126.1, 125.8, 125.6, 123.2, 122.6, 122.5, 120.3, 119.9, 117.6, 106.4, 46.9, 27.2, 13.4; HRMS (APCI) calcd for $C_{30}H_{25}O$ [M + H$^+$], 401.1890; found: 401.1895.

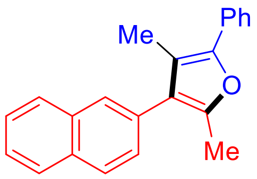

2,4-Dimethyl-3-(naphthalen-2-yl)-5-phenylfuran (4r). (16 mg, 44%). Isolated by column chromatography (petroleum ether, R_f = 0.5); ^{1}H NMR (600 MHz, $CDCl_3$) δ 7.89 (d, J = 8.4 Hz, 1H), 7.88-7.85 (m, 2H), 7.74 (s, 1H), 7.68 (d, J = 7.6 Hz, 2H), 7.52-7.47 (m, 2H), 7.45-7.41 (m, 3H), 7.27-7.25 (m, 1H), 2.40 (s, 3H), 2.24 (s, 3H); ^{13}C NMR (150 MHz, $CDCl_3$) δ 147.5, 147.0, 133.5, 132.2, 131.9, 131.1, 128.5, 128.3, 128.0, 127.9, 127.8, 127.7, 126.4, 126.1, 125.8, 125.3, 124.8, 116.6, 12.5, 10.9; HRMS (APCI) calcd for $C_{27}H_{21}O$ [M + H$^+$], 361.1587; found:

361.1589.

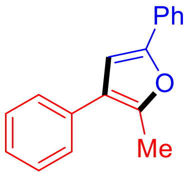

2-Methyl-3,5-diphenylfuran (4s).[3] (17 mg, 74%). Isolated by column chromatography (petroleum ether, $R_f = 0.5$); ^1H NMR (600 MHz, $CDCl_3$) δ 7.72-7.70 (m, 2H), 7.48-7.46 (m, 2H), 7.45-7.39 (m, 4H), 7.33-7.30 (m, 1H), 7.29-7.26 (m, 1H), 6.82 (s, 1H), 2.56 (s, 3H); ^{13}C NMR (150 MHz, $CDCl_3$) δ 151.8, 147.8, 134.3, 131.1, 128.9, 128.8, 127.7, 127.2, 126.7, 123.6, 123.2, 106.6, 13.4.

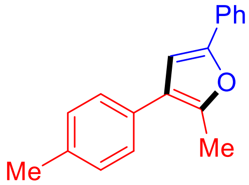

2-Methyl-5-phenyl-3-(*p*-tolyl)furan (4t).[4] (18 mg, 73%). Isolated by column chromatography (petroleum ether, $R_f = 0.5$); ^1H NMR (600 MHz, $CDCl_3$) δ 7.71-7.69 (m, 2H), 7.42-7.39 (m, 2H), 7.38-7.35 (m, 2H), 7.28-7.25 (m, 3H), 6.80 (s, 1H), 2.54 (s, 3H), 2.42 (s, 3H); ^{13}C NMR (150 MHz, $CDCl_3$) δ 151.5, 147.3, 136.1, 131.1, 130.9, 129.3, 128.6, 127.4, 127.0, 123.4, 122.9, 106.5, 21.2, 13.2.

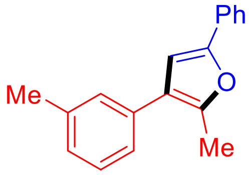

2-Methyl-5-phenyl-3-(m-tolyl)furan (4u). (17.6 mg, 71%). Isolated by column chromatography (petroleum ether, $R_f = 0.5$); ^1H NMR (600 MHz, $CDCl_3$) δ 7.68 (d, J = 7.6 Hz, 2H), 7.38 (t, J = 7.6 Hz, 2H), 7.31 (t, J = 7.6 Hz, 1H), 7.25-7.22 (m, 3H), 7.10 (d, J = 7.4 Hz, 1H), 6.78 (s, 1H), 2.52 (s, 3H), 2.41 (s, 3H); ^{13}C NMR (150 MHz, $CDCl_3$) δ 151.5, 147.5, 138.2, 134.0, 130.9, 128.6, 128.5, 128.2, 127.2, 127.0, 124.6, 123.4, 123.0, 106.5, 21.5, 13.2; HRMS (APCI) calcd for $C_{18}H_{17}O$ [M + H$^+$], 249.1274; found: 249.1271.

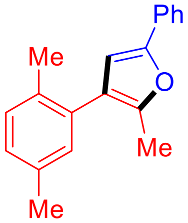

3-(2,5-Dimethylphenyl)-2-methyl-5-phenylfuran (4v). (18 mg, 70%). Isolated by column chromatography

(petroleum ether, R_f = 0.5); ^{1}H NMR (600 MHz, $CDCl_3$) δ 7.70-7.69 (m, 2H), 7.42-7.39 (m, 2H), 7.28-7.24 (m, 1H), 7.19 (d, J = 7.6 Hz, 1H), 7.09 (d, J = 7.6 Hz, 1H), 7.07 (s, 1H), 6.64 (s, 1H), 2.37 (s, 3H), 2.32 (s, 3H), 2.27 (s, 3H); ^{13}C NMR (150 MHz, $CDCl_3$) δ 151.2, 148.0, 135.1, 133.6, 133.4, 131.0, 130.8, 130.1, 128.6, 128.0, 126.9, 123.3, 122.7, 108.2, 20.9, 19.8, 12.5. HRMS (APCI) calcd for $C_{19}H_{19}O$ [M + H$^+$], 263.1430; found: 263.1434.

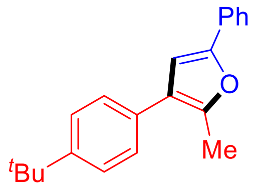

3-(4-(*tert*-Butyl)phenyl)-2-methyl-5-phenylfuran (4w). (20 mg, 68%). Isolated by column chromatography (petroleum ether, R_f = 0.5); ^{1}H NMR (600 MHz, $CDCl_3$) δ 7.72-7.70 (m, 2H), 7.49-7.47 (m, 2H), 7.43-7.39 (m, 4H), 7.28-7.25 (m, 1H), 6.81 (s, 1H), 2.56 (s, 3H), 1.40 (s, 9H); ^{13}C NMR (150 MHz, $CDCl_3$) δ 151.5, 149.4, 147.4, 131.1, 130.9, 128.6, 127.2, 127.0, 125.6, 123.4, 122.8, 106.5, 34.5, 31.4, 13.3. HRMS (APCI) calcd for $C_{21}H_{23}O$ [M + H$^+$], 291.1743; found: 291.1742.

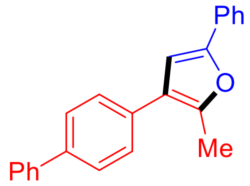

3-([1,1'-Biphenyl]-4-yl)-2-methyl-5-phenylfuran (4x). (22 mg, 71%). Isolated by column chromatography (petroleum ether, R_f = 0.5); ^{1}H NMR (600 MHz, $CDCl_3$) δ 7.69 (d, J = 7.9 Hz, 2H), 7.65-7.61 (m, 4H), 7.51 (d, J = 8.2 Hz, 2H), 7.45 (t, J = 7.6 Hz, 2H), 7.38 (t, J = 7.6 Hz, 2H), 7.35 (t, J = 7.4 Hz, 1H), 7.24 (t, J = 7.4 Hz, 1H), 6.82 (s, 1H), 2.55 (s, 3H); ^{13}C NMR (150 MHz, $CDCl_3$) δ 151.7, 147.7, 140.7, 139.2, 133.0, 130.8, 128.8, 128.6, 127.8, 127.3, 127.2, 127.0, 126.9, 123.4, 122.6, 106.3, 13.3; HRMS (APCI) calcd for $C_{23}H_{19}O$ [M + H$^+$], 311.1430; found: 311.1427.

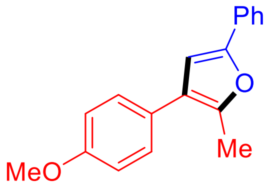

3-(4-Methoxyphenyl)-2-methyl-5-phenylfuran (4y). (17 mg, 63%). Isolated by column chromatography (petroleum ether: ethyl acetate = 100:1, R_f = 0.4); ^{1}H NMR (600 MHz, $CDCl_3$) δ 7.71-7.69 (m, 2H), 7.42-7.37 (m, 4H), 7.28-7.25 (m, 1H), 6.99 (d, J = 8.7 Hz, 2H), 6.77 (s, 1H), 3.87 (s, 3H), 2.52 (s, 3H); ^{13}C NMR (150 MHz, $CDCl_3$) δ 158.3, 151.4, 147.0, 130.9, 128.6, 126.9, 126.6, 123.4, 122.6, 114.1, 106.6, 55.3, 13.1. HRMS (APCI)

calcd for $C_{18}H_{17}O_2$ [M + H$^+$], 265.1223; found: 265.1219.

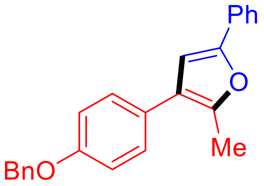

3-(4-(Benzyloxy)phenyl)-2-methyl-5-phenylfuran (4z). (21 mg, 61%). Isolated by column chromatography (petroleum ether: ethyl acetate = 100:1, R_f = 0.4); ^{1}H NMR (600 MHz, $CDCl_3$) δ 7.71-7.70 (m, 2H), 7.50-7.49 (m, 2H), 7.45-7.42 (m, 2H), 7.41-7.37 (m, 5H), 7.27-7.25 (m, 1H), 7.06 (d, J = 8.7 Hz, 2H), 6.78 (s, 1H), 5.14 (s, 2H), 2.53 (s, 3H); ^{13}C NMR (150 MHz, $CDCl_3$) δ 157.5, 151.5, 147.0, 137.0, 130.9, 128.7, 128.6, 128.0, 127.5, 127.0, 126.9, 123.4, 122.6, 115.0, 106.5, 70.1, 13.1. HRMS (APCI) calcd for $C_{24}H_{21}O_2$ [M + H$^+$], 341.1536; found: 341.1543.

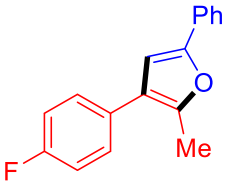

3-(4-Fluorophenyl)-2-methyl-5-phenylfuran (4aa).[3] (18 mg, 72%). Isolated by column chromatography (petroleum ether, R_f = 0.5); ^{1}H NMR (600 MHz, $CDCl_3$) δ 7.71-7.69 (m, 2H), 7.43-7.40 (m, 4H), 7.30-7.26 (m, 1H), 7.15-7.12 (m, 2H), 6.76 (s, 1H), 2.52 (s, 3H); ^{13}C NMR (150 MHz, $CDCl_3$) δ 161.6 (d, J_{C-F} = 245.8 Hz), 151.7, 141.4, 130.8, 130.1 (d, J_{C-F} = 3.2 Hz), 129.0 (d, J_{C-F} = 7.6 Hz), 128.7, 127.1, 123.4, 122.2, 115.5 (d, J_{C-F} = 21.2 Hz), 106.4, 13.1; ^{19}F NMR (565 MHz, $CDCl_3$) δ -116.1.

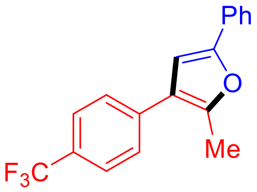

2-Methyl-5-phenyl-3-(4-(trifluoromethyl)phenyl)furan (4ab). (22 mg, 73%). Isolated by column chromatography (petroleum ether, R_f = 0.5); ^{1}H NMR (600 MHz, $CDCl_3$) δ 7.66 (d, J = 7.8 Hz, 2H), 7.66-7.64 (m, 2H), 7.52 (d, J = 7.8 Hz, 2H), 7.40-7.37 (m, 2H), 7.27-7.24 (m, 1H), 6.78 (s, 1H), 2.53 (s, 3H); ^{13}C NMR (150 MHz, $CDCl_3$) δ 152.1, 148.4, 137.7, 130.5, 128.7, 128.4 (q, J_{C-F} = 32.8 Hz), 127.5, 127.3, 125.5 (q, J_{C-F} = 3.4 Hz), 124.3 (q, J_{C-F} = 271.4 Hz), 123.5, 122.0, 105.9, 13.3. HRMS (APCI) calcd for $C_{18}H_{14}F_3O$ [M + H$^+$], 303.0991; found: 303.0987.

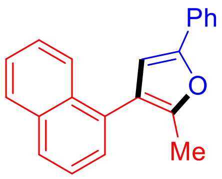

2-Methyl-3-(naphthalen-1-yl)-5-phenylfuran (4ac). (16 mg, 55%). Isolated by column chromatography (petroleum ether, R_f = 0.6); ^{1}H NMR (600 MHz, $CDCl_3$) δ 7.98 (d, J = 7.8 Hz, 1H), 7.90 (d, J = 7.8 Hz, 1H), 7.85 (d, J = 8.2 Hz, 1H), 7.72 (d, J = 7.4 Hz, 2H), 7.53-7.46 (m, 3H), 7.43-7.39 (m, 3H), 7.27-7.25 (m, 1H), 6.80 (s, 1H), 2.32 (s, 3H); ^{13}C NMR (150 MHz, $CDCl_3$) δ 151.5, 148.9, 133.8, 132.2, 131.8, 130.9, 128.7, 128.3, 127.6, 127.4, 127.0, 126.0, 125.8, 125.4, 123.4, 121.5, 108.9, 12.6; HRMS (ESI) calcd for $C_{21}H_{17}O$ [M + H$^+$], 285.1274; found: 285.1272.

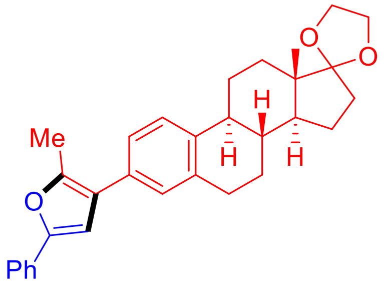

(8R,9S,13S,14S)-13-Methyl-3-(2-methyl-5-phenylfuran-3-yl)-6,7,8,9,11,12,13,14,15,16-decahydrospiro[cyclop enta[a]phenanthrene-17,2'-[1,3]dioxolane] (4ae). (27.7 mg, 61%). Isolated by column chromatography (petroleum ether: ethyl acetate = 50:1, R_f = 0.4); ^{1}H NMR (600 MHz, $CDCl_3$) δ 7.71-7.69 (m, 2H), 7.42-7.37 (m, 3H), 7.27-7.24 (m, 2H), 7.19 (s, 1H), 4.02-3.91 (m, 4H), 2.96-2.94 (m, 2H), 2.54 (s, 3H), 2.44-2.33 (m, 2H), 2.10-2.05 (m, 1H), 1.99-1.95 (m, 1H), 1.92-1.80 (m, 3H), 1.73-1.67 (m, 1H), 1.62-1.36 (m, 8H), 0.93 (s, 3H); ^{13}C NMR (150 MHz, $CDCl_3$) δ 151.5, 147.4, 138.8, 137.0, 131.3, 130.9, 128.6, 128.0, 126.9, 125.7, 124.8, 123.4, 122.8, 119.5, 106.6, 65.3, 64.6, 49.5, 46.2, 44.1, 38.9, 34.3, 30.8, 29.7, 27.0, 26.0, 22.4, 14.4, 13.2. HRMS (APCI) calcd for $C_{31}H_{35}O_3$ [M + H$^+$], 455.2581; found: 455.2573.

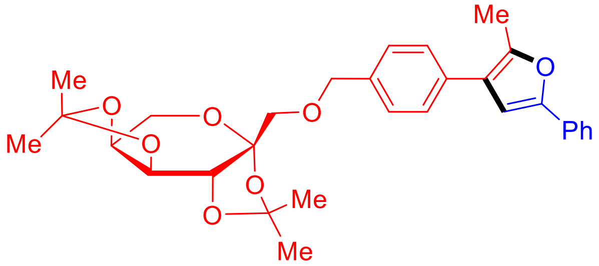

(3aR,5aS,8aS,8bR)-2,2,7,7-Tetramethyl-3a-(((4-(2-methyl-5-phenylfuran-3-yl)benzyl)oxy)methyl)tetrahydro-5H-bis([1,3]dioxolo)[4,5-b:4',5'-d]pyran (4af). (27.3 mg, 54%). Isolated by column chromatography (petroleum

ether: ethyl acetate = 4:1, R_f = 0.4); ^{1}H NMR (600 MHz, $CDCl_3$) δ 7.67 (d, J = 7.6 Hz, 2H), 7.41-7.36 (m, 6H), 7.24 (t, J = 7.6 Hz, 1H), 6.77 (s, 1H), 4.70 (d, J = 12.2 Hz, 1H), 4.62-4.60 (m, 2H), 4.46 (d, J = 1.8 Hz, 1H), 4.23 (d, J = 7.8 Hz, 1H), 3.93 (d, J = 13.0 Hz, 1H), 3.74 (d, J = 13.0 Hz, 1H), 3.66 (d, J = 10.6 Hz, 1H), 3.62 (d, J = 10.6 Hz, 1H), 2.51 (s, 3H), 1.56 (s, 3H), 1.45 (s, 3H), 1.42 (s, 3H), 1.33 (s, 3H); ^{13}C NMR (150 MHz, $CDCl_3$) δ 151.6, 147.6, 136.3, 133.3, 130.8, 128.6, 127.9, 127.4, 127.0, 123.4, 122.8, 108.9, 108.5, 106.4, 102.7, 73.5, 71.6, 71.0, 70.3, 70.2, 61.0, 26.6, 25.8, 24.0, 13.2; HRMS (ESI) calcd for $C_{30}H_{35}O_7$ [M + H$^+$], 507.2377; found: 507.2374.

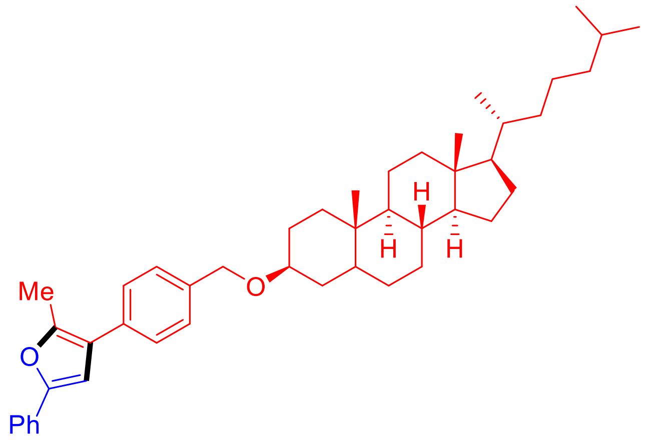

3-(4-((((3S,8R,9S,10S,13R,14S,17R)-10,13-Dimethyl-17-((R)-6-methylheptan-2-yl)hexadecahydro-1H-cyclopenta[a]phenanthren-3-yl)oxy)methyl)phenyl)-2-methyl-5-phenylfuran (4ag). (36.1 mg, 57%). Isolated by column chromatography (petroleum ether: ethyl acetate = 20:1, R_f = 0.4); ^{1}H NMR (600 MHz, $CDCl_3$) δ 7.67 (d, J = 7.6 Hz, 2H), 7.41-7.36 (m, 6H), 7.25-7.23 (m, 1H), 6.77 (s, 1H), 4.58 (d, J = 11.8 Hz, 1H), 4.56 (d, J = 11.8 Hz, 1H), 3.39-3.33 (m, 1H), 2.51 (s, 3H), 1.97-1.92 (m, 2H), 1.82-1.77 (m, 1H), 1.74-1.64 (m, 3H), 1.54-1.46 (m, 4H), 1.37-1.22 (m, 10H), 1.14-1.03 (m, 6H), 0.95-0.80 (m, 16H), 0.65-0.59 (m, 4H); ^{13}C NMR (150 MHz, $CDCl_3$) δ 151.6, 147.5, 137.4, 133.1, 130.8, 128.6, 127.9, 127.4, 127.0, 123.4, 122.8, 106.4, 78.1, 69.6, 56.5, 56.3, 54.4, 44.9, 42.6, 40.0, 39.5, 37.0, 36.2, 35.8, 35.7, 35.5, 34.9, 32.1, 28.9, 28.3, 28.2, 28.0, 24.2, 23.8, 22.8, 22.6, 21.2, 18.7, 13.2, 12.3, 12.1; HRMS (APCI) calcd for $C_{45}H_{63}O_2$ [M + H$^+$], 635.4823; found: 635.4821.

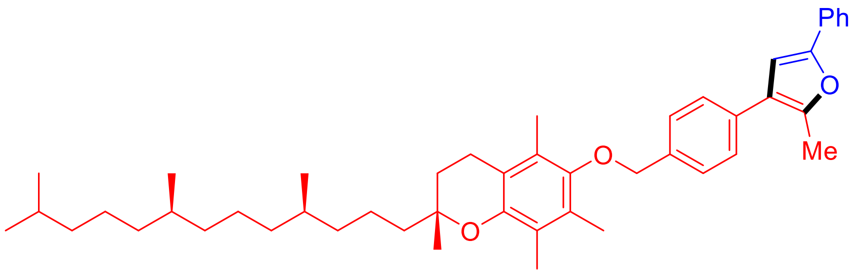

(R)-2,5,7,8-tetramethyl-6-((4-(2-Methyl-5-phenylfuran-3-yl)benzyl)oxy)-2-((4R,8R)-4,8,12-trimethyltridecyl)

chromane (4ah). (37.2 mg, 55%). Isolated by column chromatography (petroleum ether: ethyl acetate = 20:1, R_f = 0.4); ^{1}H NMR (600 MHz, $CDCl_3$) δ 7.68 (d, J = 7.6 Hz, 2H), 7.55 (d, J = 7.6 Hz, 2H), 7.47 (d, J = 7.6 Hz, 2H), 7.38 (t, J = 7.6 Hz, 2H), 7.24 (t, J = 7.6 Hz, 1H), 6.79 (s, 1H), 4.72 (s, 2H), 2.60 (t, J = 6.2 Hz, 2H), 2.53 (s, 3H), 2.25 (s, 3H), 2.19 (s, 3H), 2.12 (s, 3H), 1.86-1.75 (m, 2H), 1.54-1.08 (m, 26H), 0.87-0.84 (m, 12H); ^{13}C NMR (150 MHz, $CDCl_3$) δ 151.6, 148.1, 147.9, 147.6, 136.2, 133.6, 130.8, 128.6, 128.0, 127.9, 127.6, 127.0, 125.9, 123.4, 122.9, 122.8, 117.6, 106.4, 74.8, 74.5, 40.1, 39.4, 37.6, 37.5, 37.4, 37.3, 32.8, 32.7, 32.6, 31.3, 31.2, 28.0, 24.8, 24.4, 23.9, 22.7, 22.6, 21.0, 20.7, 19.8, 19.7, 19.6, 13.2, 12.9, 12.0, 11.8; HRMS (APCI) calcd for $C_{47}H_{65}O_3$ [M + H^+], 677.4928; found: 677.4925.

7. Control experiments

BHT (0.1 mmol) or 1,1-diphenylethylene (0.1 mmol) was added and the other operations were following the general procedure. The yield of product **3a** was not affected in either case.

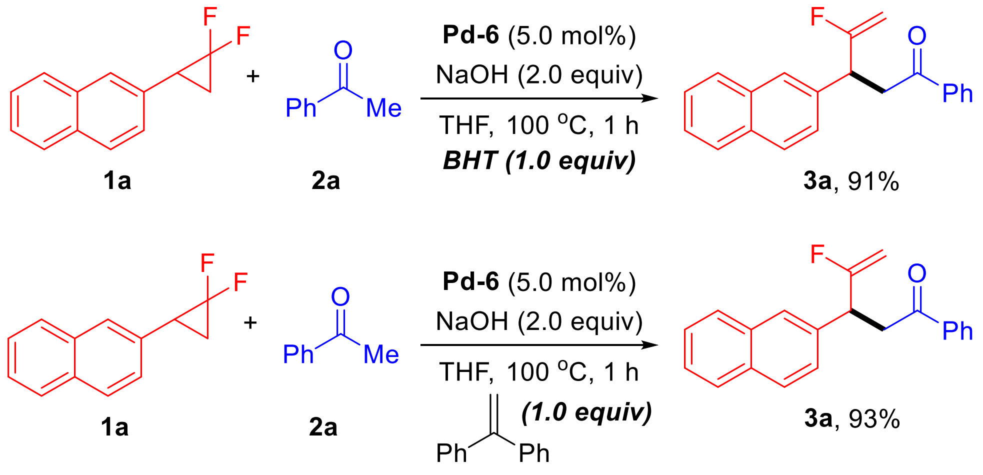

Scheme S1. Control experiements with BHT or 1,1-diphenylethylene as additive

8. Time-course reaction

A set of flame-dried reaction tubes (10 cm^3) equipped with a magnetic stir bar was charged with Pd-PEPPSI-IHept (4.5 mg, 5 mol%) respectively. These reaction tubes were transferred to the glovebox and charged with **1a** (0.1 mmol), **2a** (0.2 mmol), NaOH (8.0 mg) and THF (1.0 mL), respectively. After that, the corresponding reaction mixtures were sealed with aluminum cap, moved out of glovebox, and stirred at 100 °C for 10 min, 15 min, 17 min, 20 min, 30 min, 40 min, 50 min and 60 min, respectively. Once stirred at the indicated time, the reaction mixture was quickly cooled to room temperature, the resulting solution was directly filtered through a pad of silica gel by

EtOAc (3.0 mL). The solvent was evaporated *in vacuo* to give the crude product. The yield of **3a** and remaining **1a** were determined by ^{1}H NMR using CH_2Br_2 as an internal standard. The corresponding data and time-course plot were shown in Scheme S2.

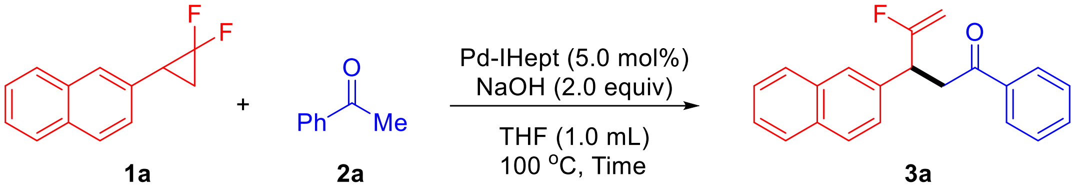

Entry	Time/min	**3a** (%)	**1a** (%)
1	0	0	100
2	10	2	96
3	15	8	90
4	17	44	55
5	20	55	40
6	30	67	32
7	40	85	5
8	50	90	2
9	60	95	0

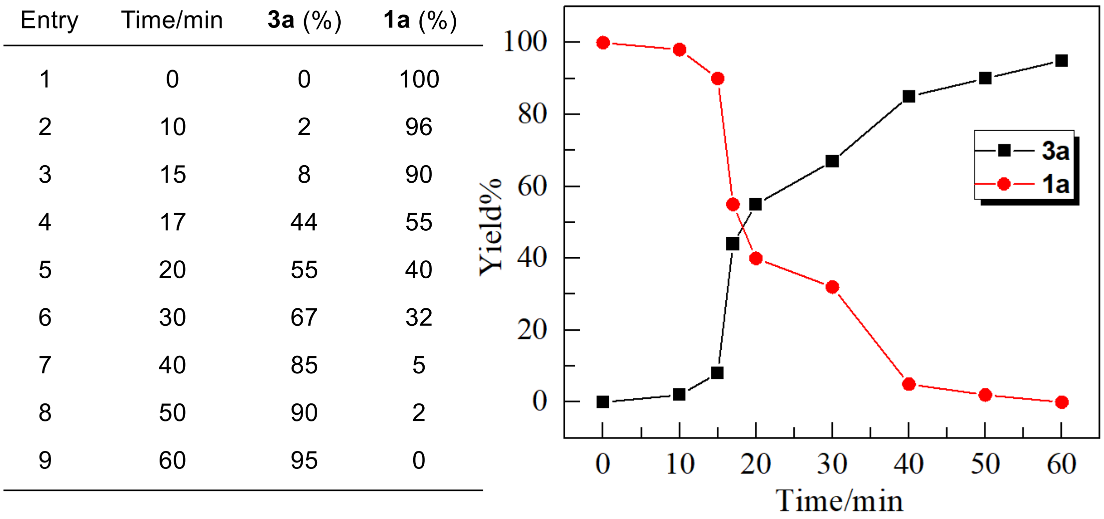

Scheme S2. Time-course reaction

9. References

[1] (a) Chartoire, A.; Frogneux, X.; Boreux, A.; Slawin, A. M. Z.; Nolan, S. P. [Pd(IPr*)(3-Cl-pyridinyl)Cl_2]: A Novel and Efficient PEPPSI Precatalyst. *Organometallics* **2012,** *31*, 6947-6951. (b) Valente, C.; Çalimsiz, S.; Hoi, K. H.; Mallik, D.; Sayah, M.; Organ, M. G. The Development of Bulky Palladium NHC Complexes for the Most-Challenging Cross-Coupling Reactions. *Angew. Chem. Int. Ed.* **2012,** *51*, 3314-3332. (c) Valente, C.; Pompeo, M.; Sayah, M.; Organ, M. G. Carbon–Heteroatom Coupling Using Pd-PEPPSI Complexes. *Org. Process Res. Dev.* **2014,** *18*, 180-190.

[2] (a) F. Wang, T. Luo, J. Hu, Y. Wang, H. S. Krishnan, P. V. Jog, S. K. Ganesh, G. K. S. Prakash, G. A. Olah, *Angew. Chem. Int. Ed.* **2011**, *50*, 7153-7157. (b) J. Xu, E.-A. Ahmed, B. Xiao, Q.-Q. Lu, Y.-L. Wang, C.-G. Yu, Y. Fu, *Angew. Chem. Int. Ed.* **2015**, *54*, 8231-8235. (c) Lv, L.; Li, C. J. Palladium-Catalyzed Defluorinative Alkylation of gem-Difluorocyclopropanes: Switching Regioselectivity via Simple Hydrazones. *Angew. Chem. Int. Ed.* **2021,**

60, 13098-13104.

[3] Tan, W. W.; Yoshikai, N. Copper-Catalyzed Coupling of 2-Siloxy-1-alkenes and Diazocarbonyl Compounds: Approach to Multisubstituted Furans, Pyrroles, and Thiophenes. *J. Org. Chem.* **2016,** *81*, 5566-5573.

[4] Xia, Y.; Xia, Y.; Ge, R.; Liu, Z.; Xiao, Q.; Zhang, Y.; Wang, J. Oxidative Cross-Coupling of Allenyl Ketones and Organoboronic Acids: Expeditious Synthesis of Highly Substituted Furans. *Angew. Chem. Int. Ed.* **2014,** *53*, 3917-3921.

10. Copies of ^{1}H NMR, ^{13}C NMR and ^{19}F NMR spectra for new compounds

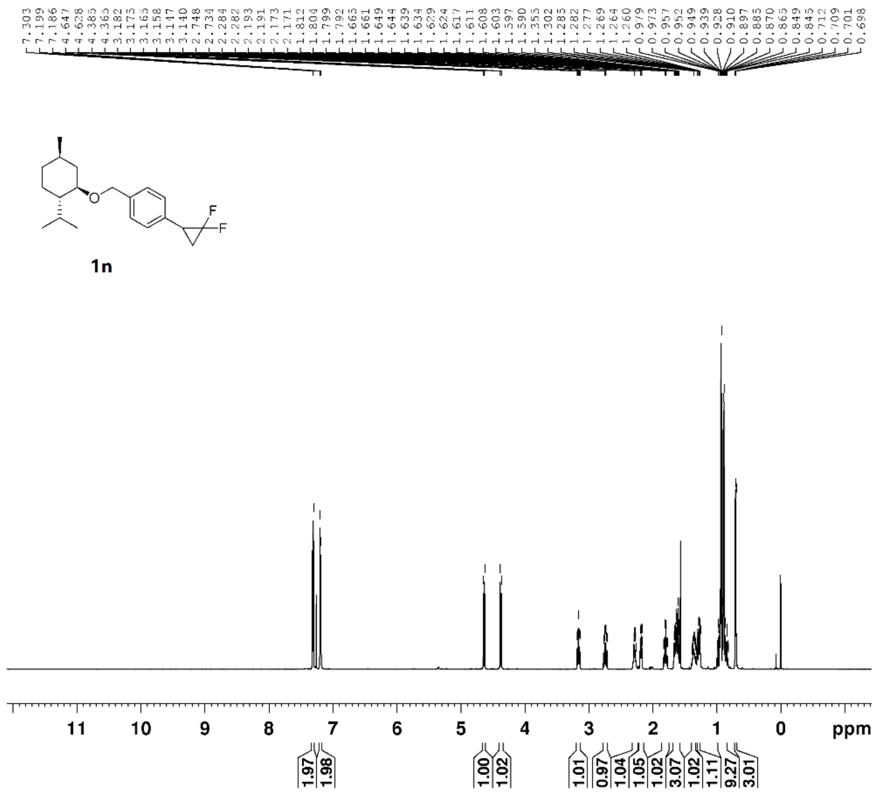

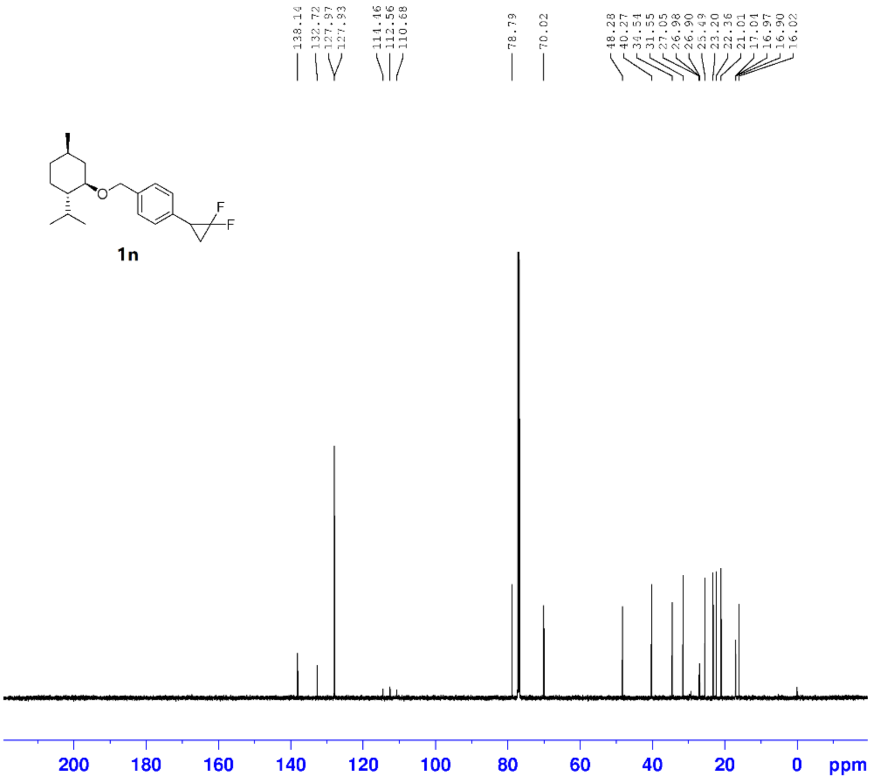

138.14
132.72
127.97
127.93
111.46
112.56
110.68
78.79
70.02
48.28
40.27
34.54
31.55
27.05
26.98
26.90
25.49
23.20
22.36
21.01
17.01
16.97
16.90
16.02
O
F
F
1n
200
180
160
140
120
100
80
60
40
20
0
ppm

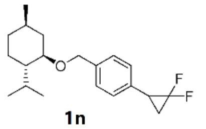
F
F
O

1n

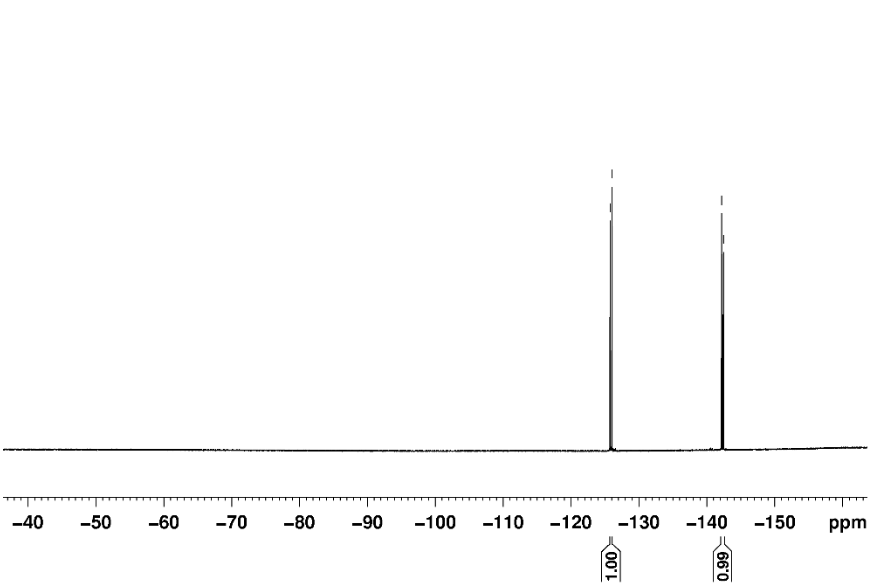
-125.735
-125.742
-125.760
-125.766
-125.782
-125.789
-126.007
-126.013
-126.032
-126.038
-126.053
-126.060
-142.163
-142.171
-142.185
-142.193
-142.203
-142.217
-142.225
-142.435
-142.442
-142.457
-142.465
-142.475
-142.489
-142.497
−40
−50
−60
−70
−80
−90
−100
−110
−120
−130
−140
−150
ppm
1.00
0.99

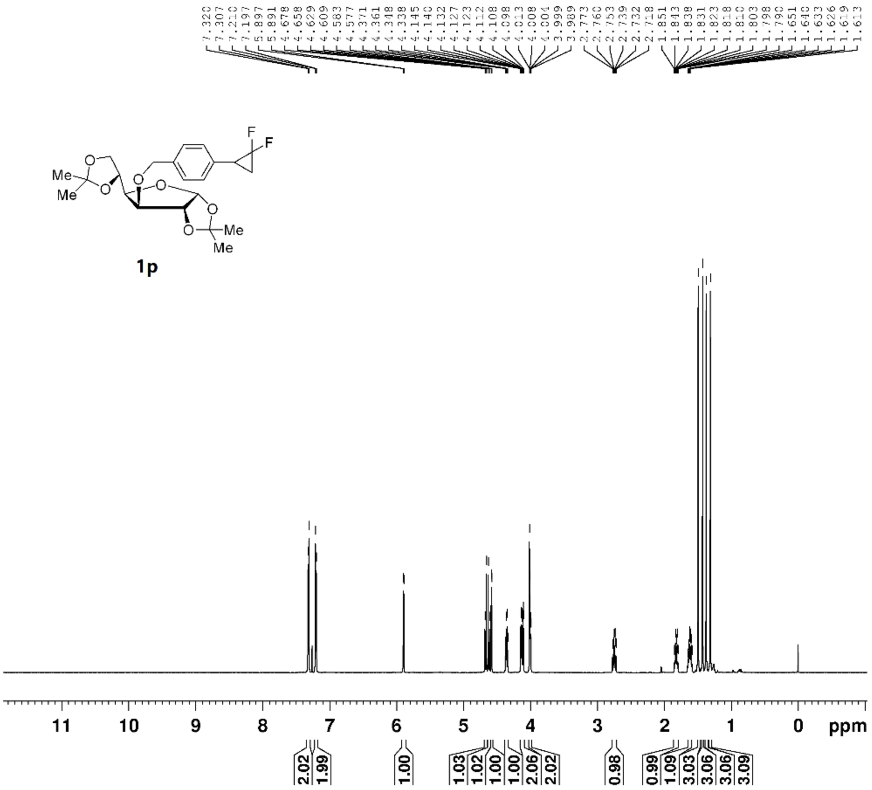

1p

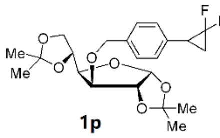

1p

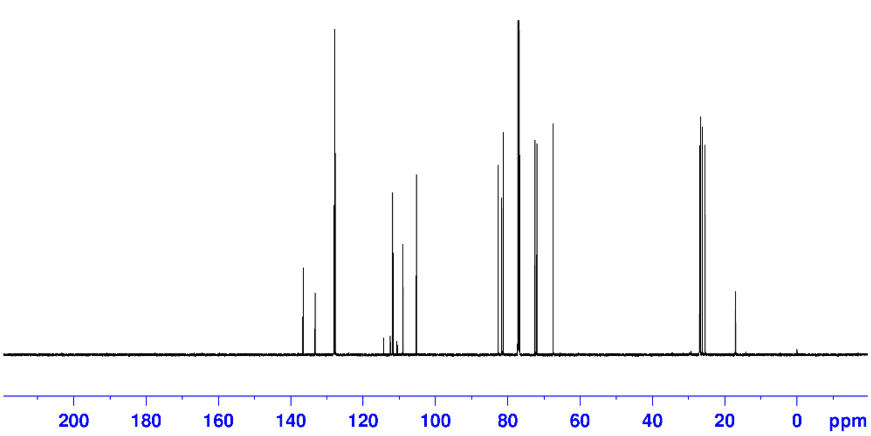
136.61
133.27
128.06
127.77
114.36
112.48
112.47
111.78
110.58
108.98
105.26
82.61
81.66
81.27
72.44
71.96
67.41
27.00
26.92
26.85
26.80
26.76
26.20
25.41
17.09
17.02
16.95
200
180
160
140
120
100
80
60
40
20
0
ppm

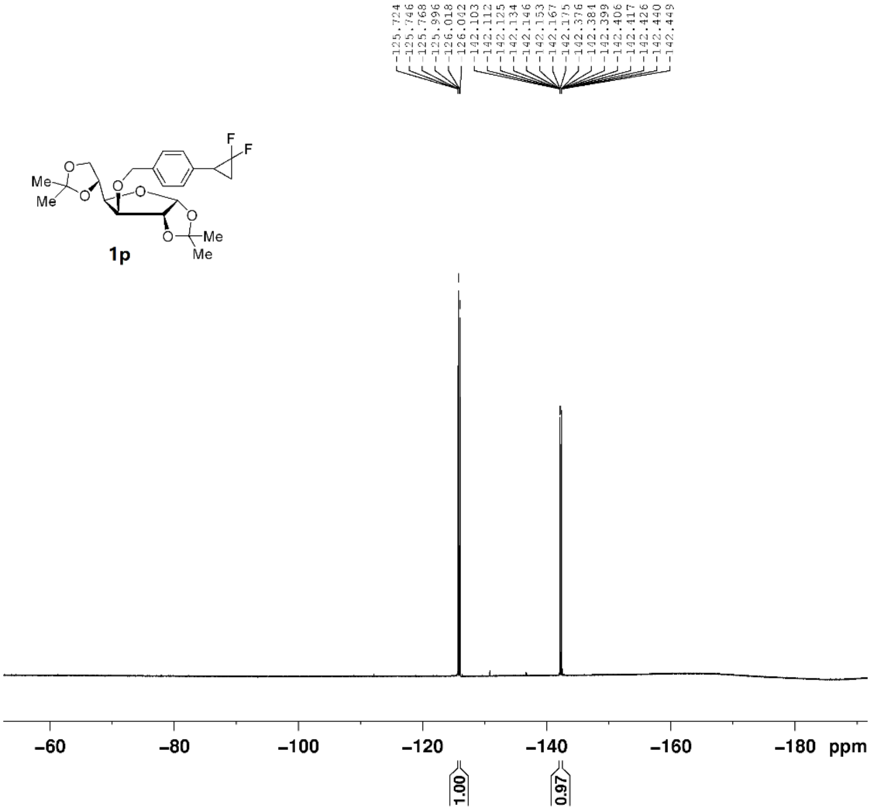

-125.724
-125.746
-125.768
-125.996
-126.018
-126.042
-142.103
-142.112
-142.125
-142.134
-142.146
-142.153
-142.167
-142.175
-142.376
-142.381
-142.399
-142.406
-142.417
-142.426
-142.440
-142.449
F
F
O
O
O
O
O
Me
Me
Me
Me
1p
−60
−80
−100
−120
−140
−160
−180
ppm
1.00
0.97

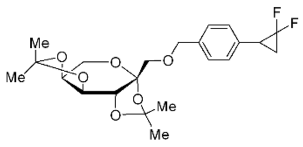

1q

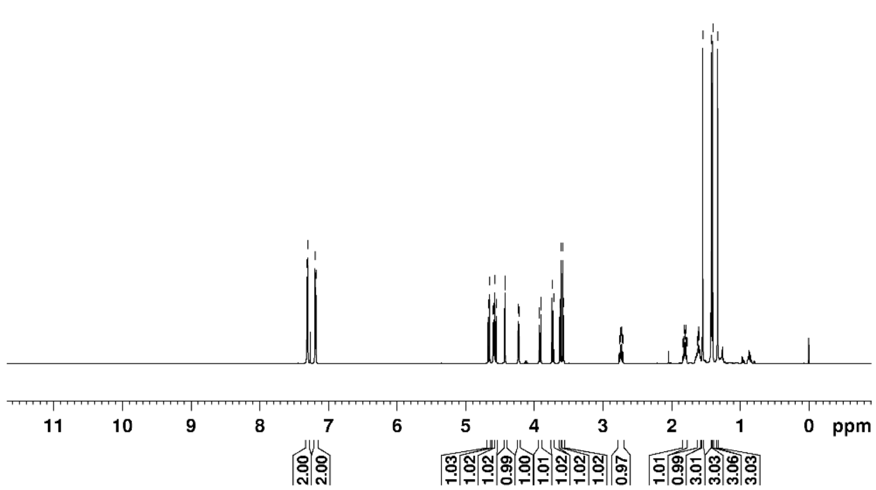

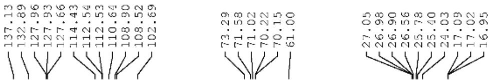

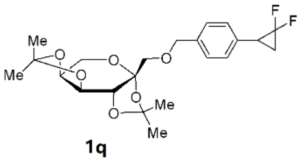

1q

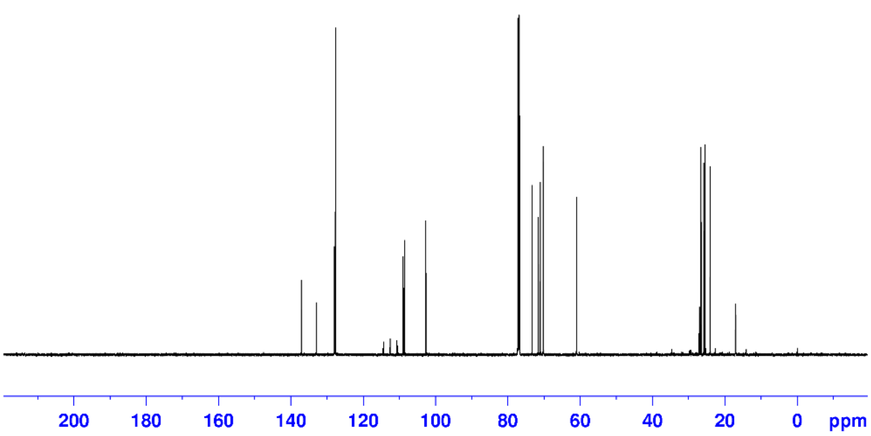

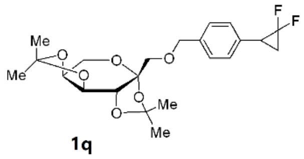

Me
Me
O
O
O
O
O
Me
Me
O
F
F

1q

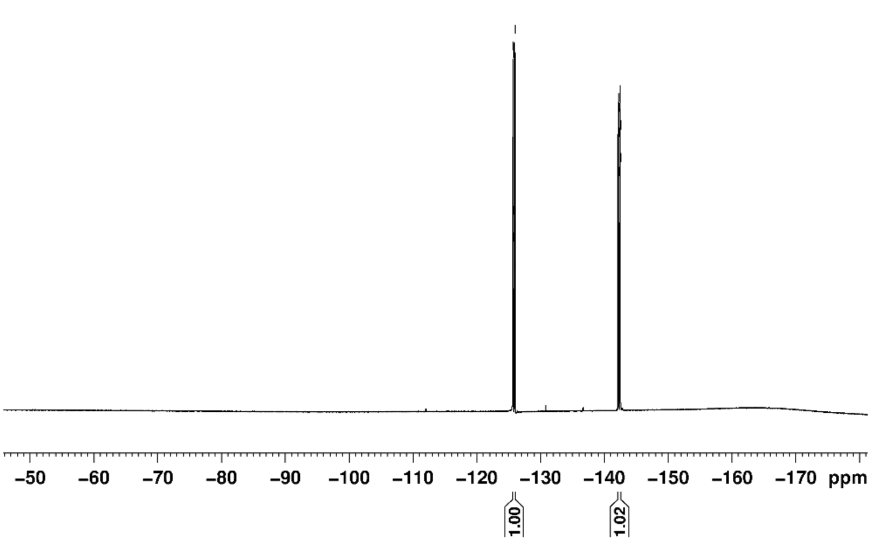

-125.700
-125.706
-125.714
-125.722
-125.734
-125.742
-125.752
-125.759
-125.975
-125.985
-125.995
-126.003
-126.009
-126.016
-126.031
-126.037
-142.172
-142.181
-142.196
-142.203
-142.240
-142.247
-142.262
-142.269
-142.446
-142.453
-142.468
-142.475
-142.512
-142.521
-142.535
-142.543
-50
-60
-70
-80
-90
-100
-110
-120
-130
-140
-150
-160
-170
ppm
1.00
1.02

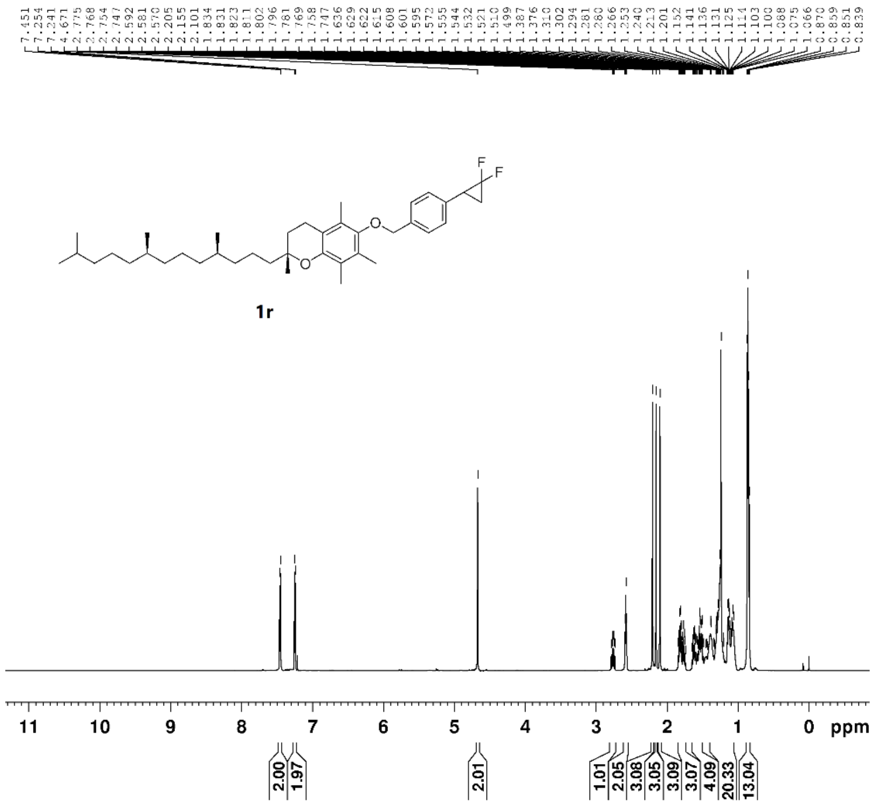

F
F
O
O
1r
11
10
9
8
7
6
5
4
3
2
1
0
ppm
2.00
1.97
2.01
1.01
2.05
3.08
3.05
3.09
3.07
4.09
20.33
13.04

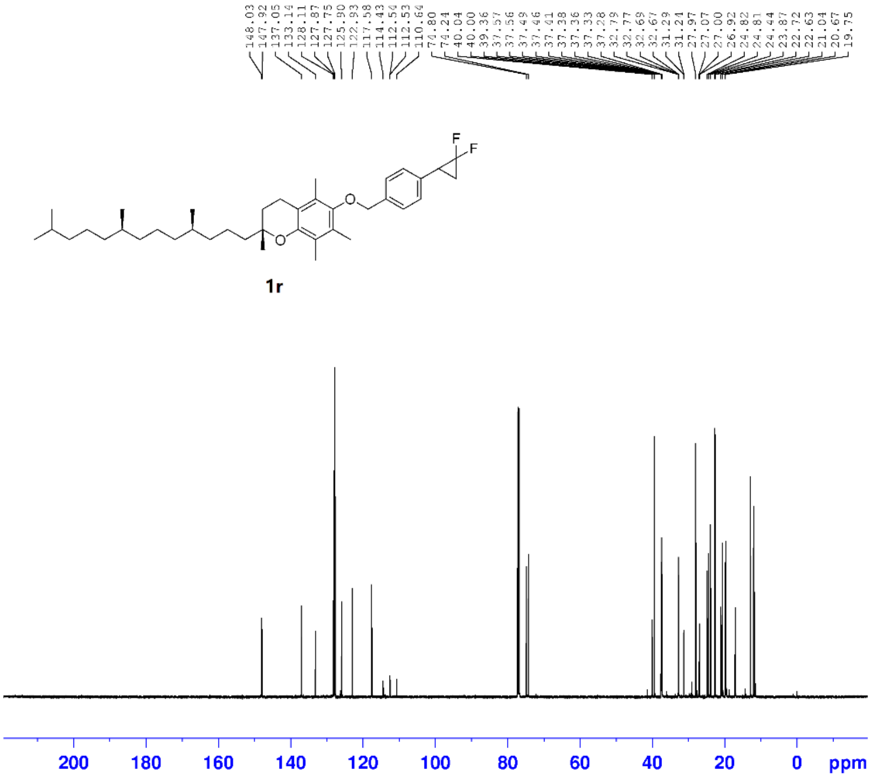

148.03
147.92
137.05
133.14
128.11
127.87
127.75
125.90
122.93
117.58
114.43
112.54
112.53
110.64
74.80
74.24
40.04
40.00
39.36
37.57
37.56
37.49
37.46
37.41
37.38
37.36
37.33
37.28
32.79
32.77
32.69
32.67
31.29
31.24
27.97
27.07
27.00
26.92
24.82
24.81
24.44
23.87
22.72
22.63
21.04
20.67
19.75
F
F
O
O
200
180
160
140
120
100
80
60
40
20
0
ppm

1r

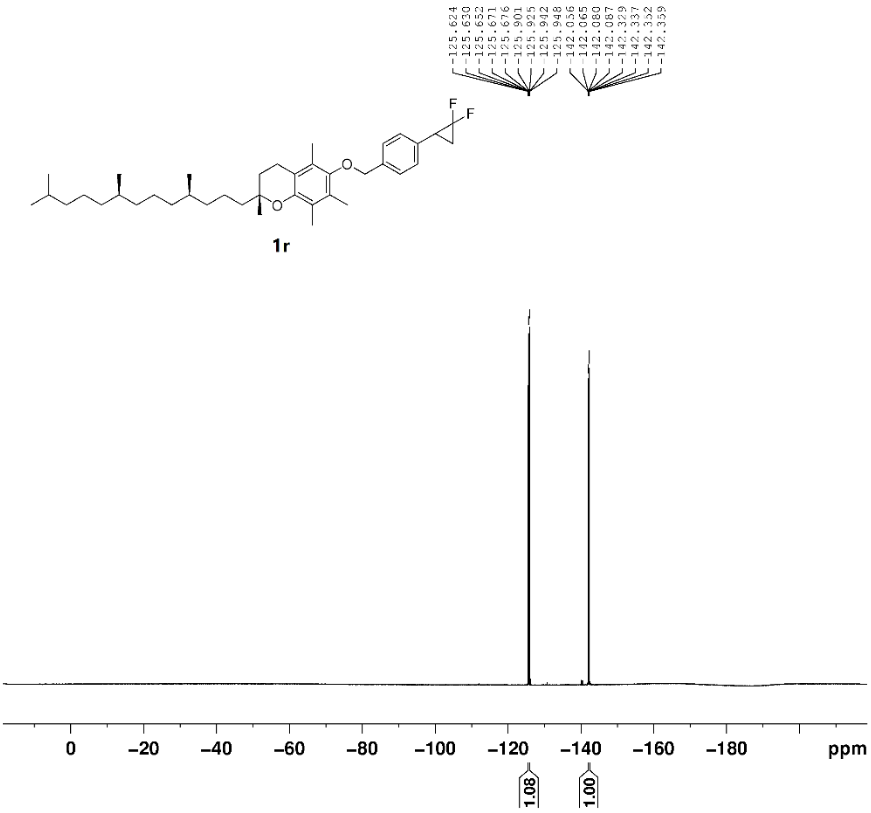
-125.624
-125.630
-125.652
-125.671
-125.676
-125.901
-125.925
-125.942
-125.948
-142.056
-142.065
-142.080
-142.087
-142.329
-142.337
-142.352
-142.359
F
F
O
O
1r
0
−20
−40
−60
−80
−100
−120
−140
−160
−180
ppm
1.08
1.00

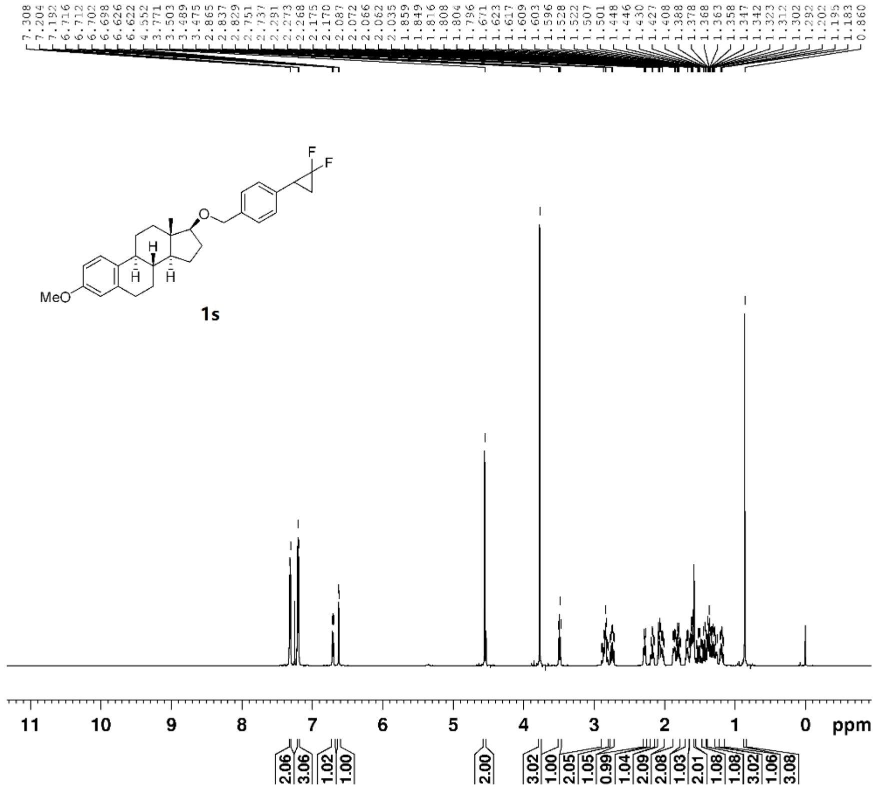

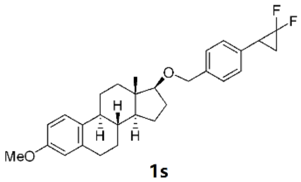

1s

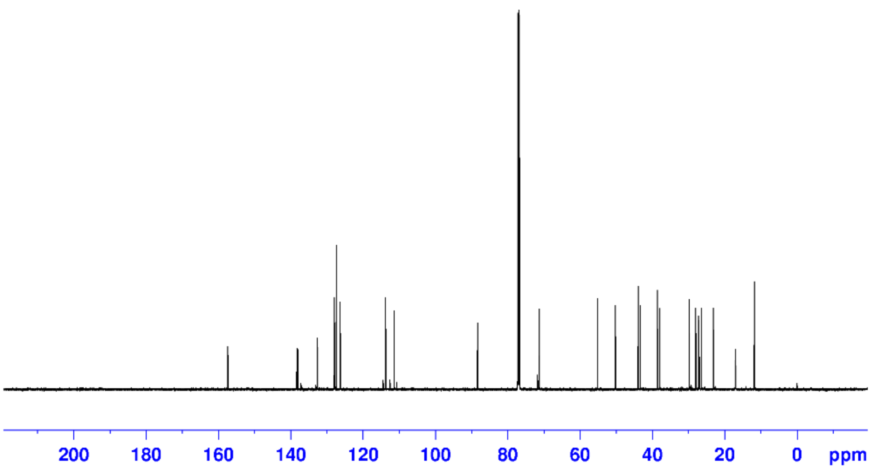

157.40
138.33
137.96
132.67
132.62
127.94
127.40
126.31
114.48
113.76
112.60
111.44
110.70
88.36
71.26
55.18
50.20
43.93
43.39
38.60
37.94
29.81
27.99
27.21
27.06
26.98
26.91
26.42
23.11
17.07
17.00
16.93
11.80
200
180
160
140
120
100
80
60
40
20
0
ppm

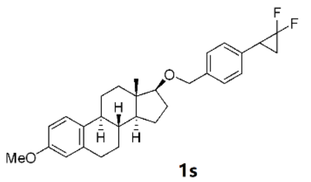

1s

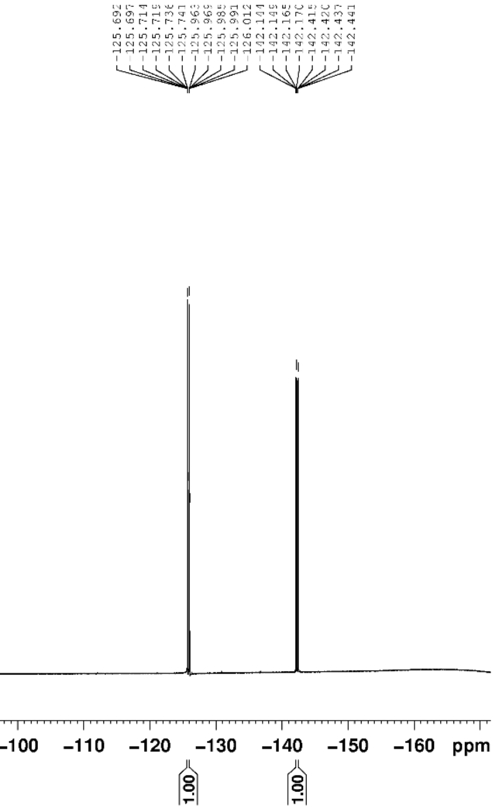

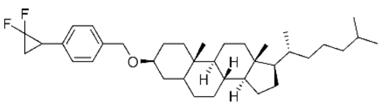
F
F
O
H
H
H

1t

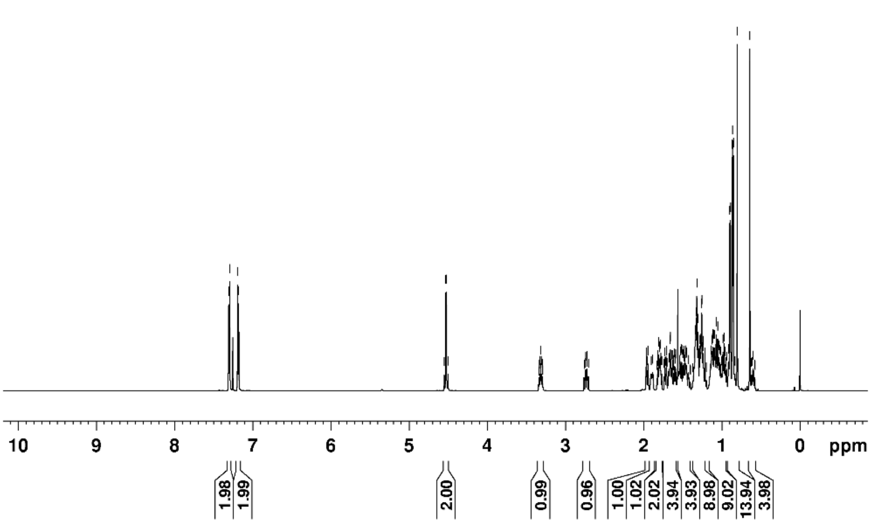
7.296
7.194
7.181
4.530
4.523
3.317
1.966
1.945
1.809
1.801
1.796
1.789
1.731
1.709
1.663
1.658
1.607
1.533
1.522
1.511
1.488
1.465
1.458
1.451
1.356
1.340
1.331
1.320
1.302
1.285
1.280
1.274
1.269
1.260
1.257
1.244
1.237
1.215
1.140
1.128
1.122
1.115
1.101
1.093
1.078
1.071
1.056
1.045
1.034
1.024
1.014
0.986
0.976
0.969
0.958
0.917
0.910
0.899
0.888
0.867
0.863
0.856
0.852
0.802
0.642
10
9
8
7
6
5
4
3
2
1
0
ppm
1.98
1.99
2.00
0.99
0.96
1.00
1.02
2.02
3.94
3.93
8.98
9.02
13.94
3.98

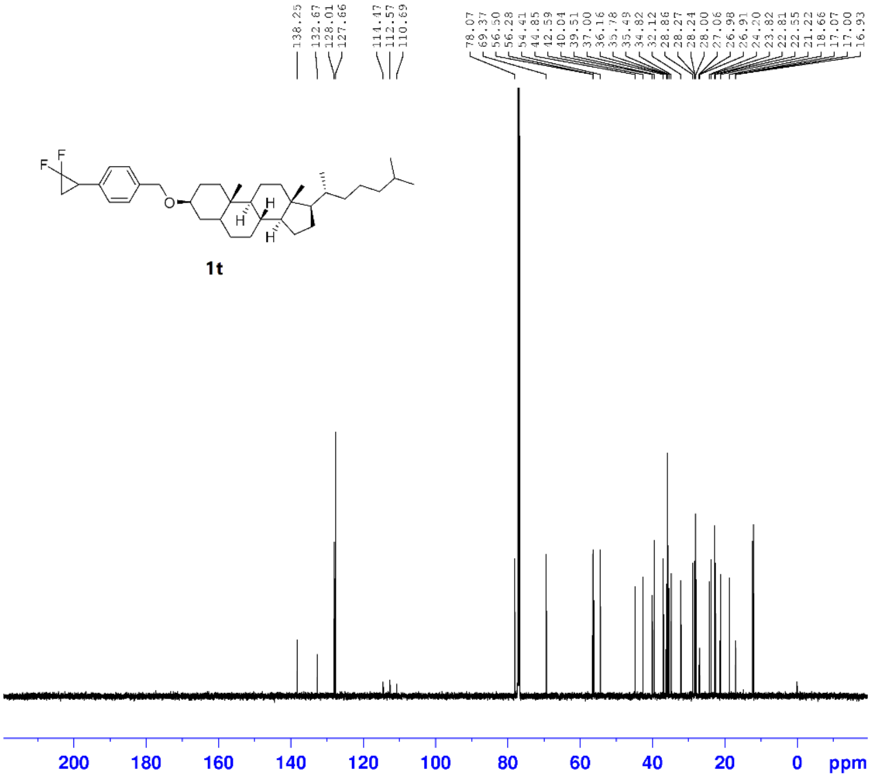
1t

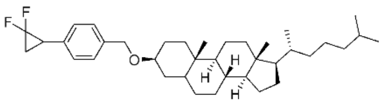
F
F
O
H
H
H

1t

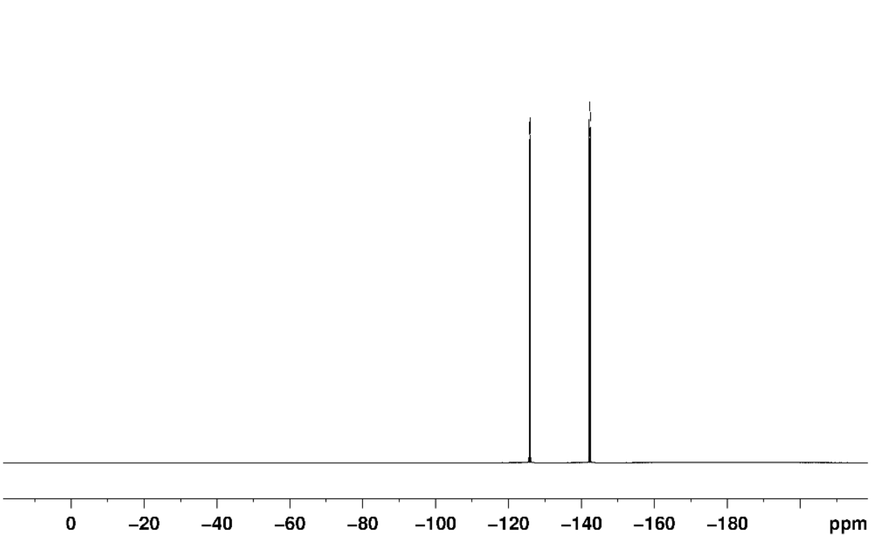
-125.727
-125.735
-125.999
-126.006
-142.152
-142.185
-142.424
-142.457
0
−20
−40
−60
−80
−100
−120
−140
−160
−180
ppm

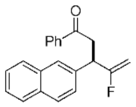
O
Ph
F

3a

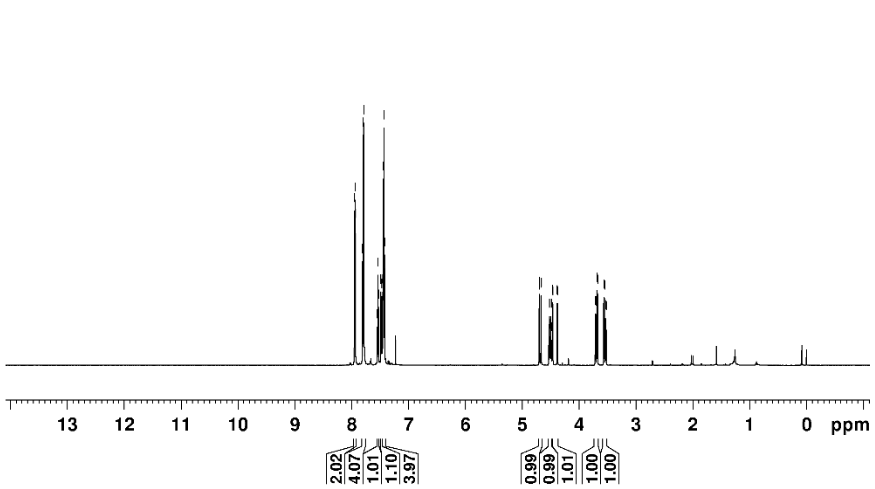
7.950
7.938
7.936
7.812
7.798
7.788
7.552
7.540
7.528
7.488
7.486
7.474
7.472
7.465
7.463
7.454
7.452
7.446
7.442
7.439
7.433
7.420
4.701
4.696
4.672
4.667
4.532
4.521
4.509
4.505
4.493
4.481
4.468
4.463
4.385
4.380
3.716
3.704
3.687
3.675
3.566
3.554
3.537
3.525
13
12
11
10
9
8
7
6
5
4
3
2
1
0
ppm
2.02
4.07
1.01
1.10
3.97
0.99
0.99
1.01
1.00
1.00

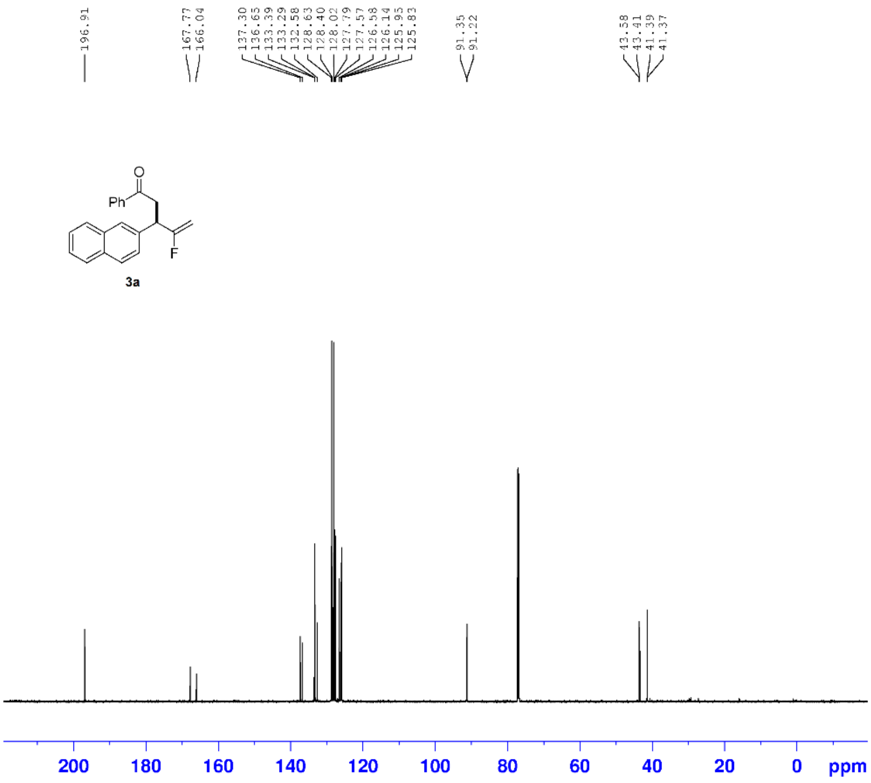
196.91
167.77
166.04
137.30
136.65
133.39
133.29
132.58
128.63
128.40
128.02
127.79
127.57
126.58
126.14
125.95
125.83
91.35
91.22
43.58
43.41
41.39
41.37
Ph
O
F
3a
200
180
160
140
120
100
80
60
40
20
0
ppm

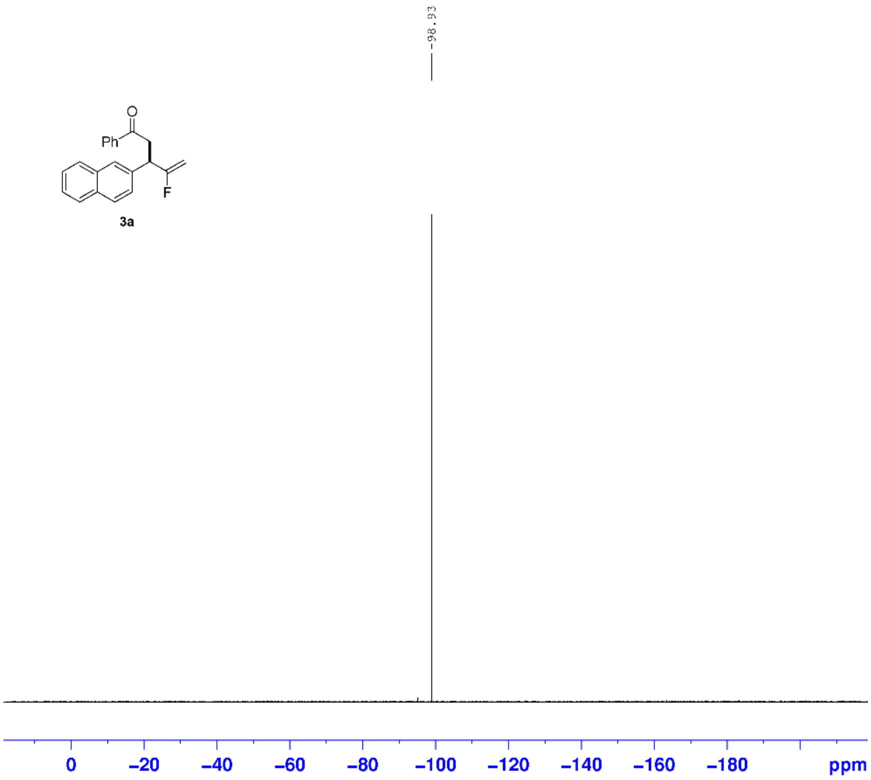

-98.93
Ph
O
F
3a
0
–20
–40
–60
–80
–100
–120
–140
–160
–180
ppm

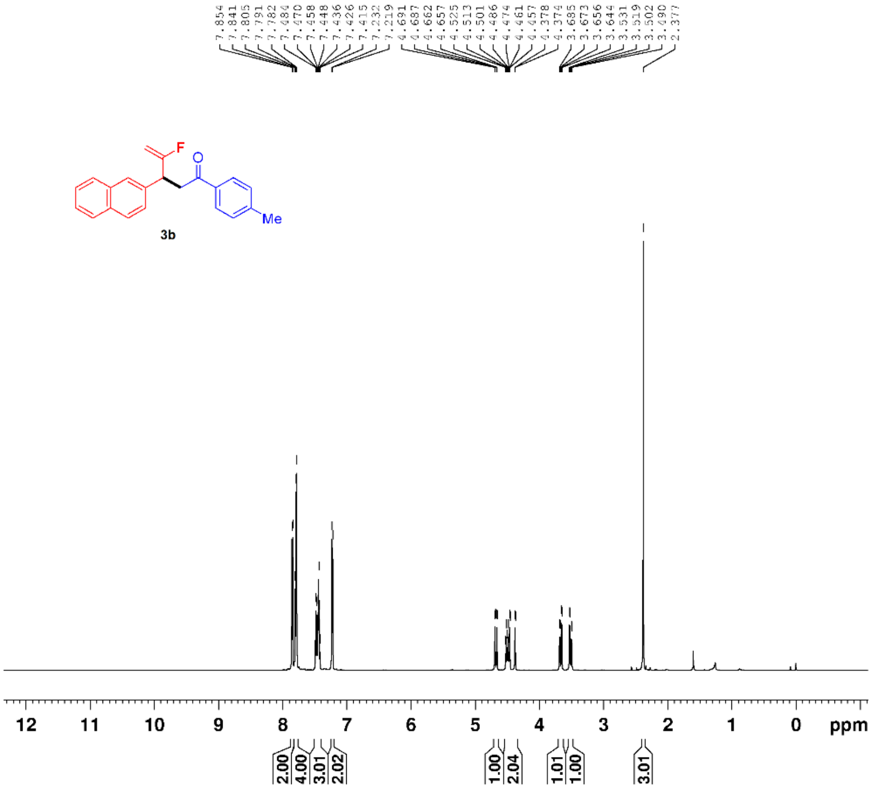

3b

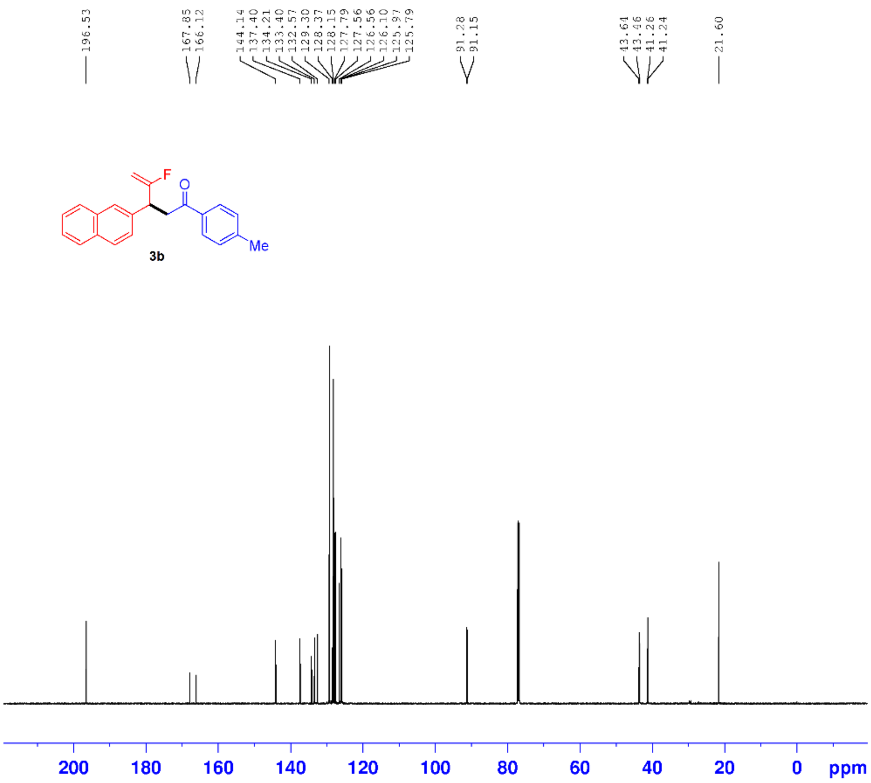
196.53
167.85
166.12
144.14
137.40
134.21
133.40
132.57
129.30
128.37
128.15
127.79
127.56
126.56
126.10
125.97
125.79
91.28
91.15
43.61
43.46
41.26
41.24
21.60
F
O
Me
3b
200
180
160
140
120
100
80
60
40
20
0
ppm

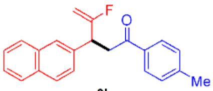

3b

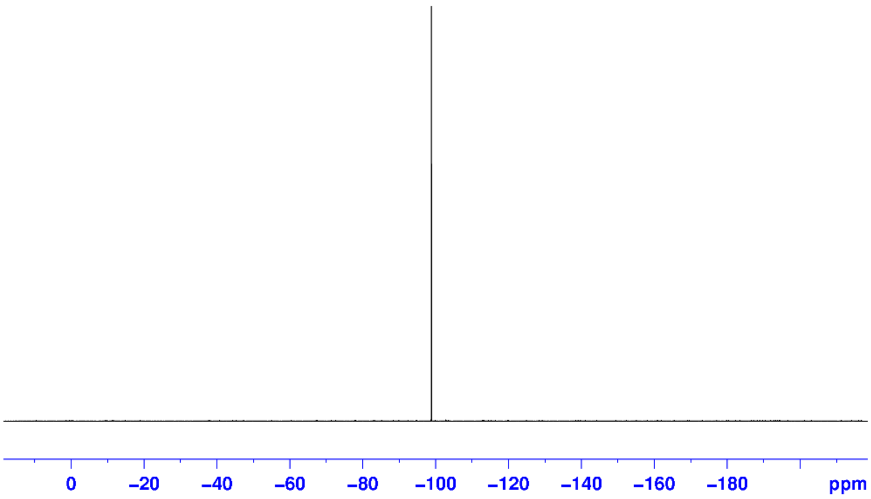

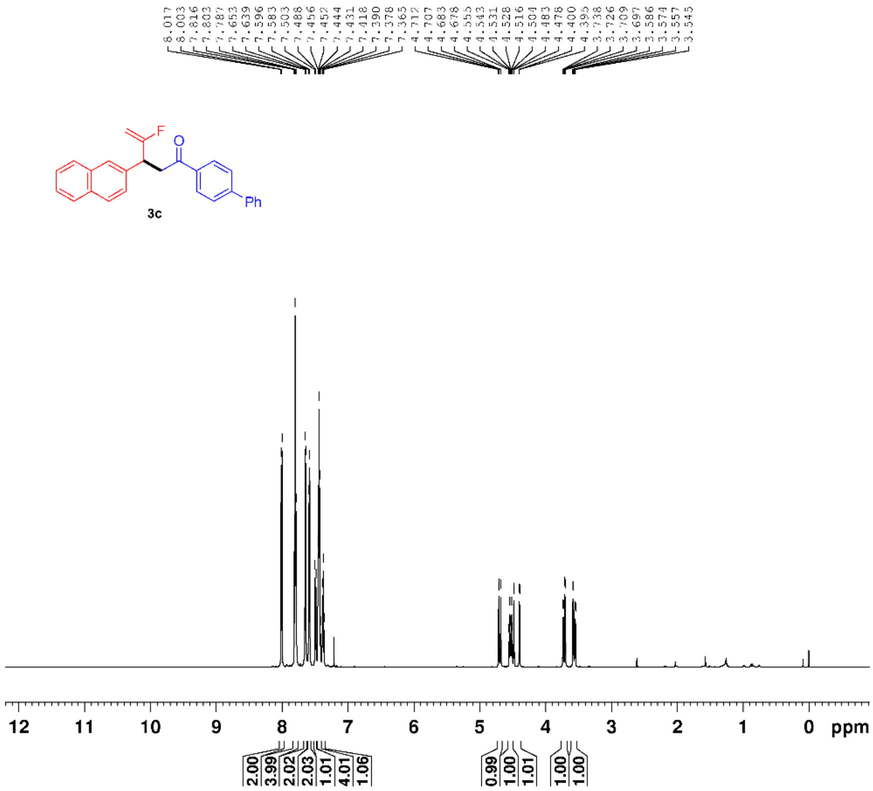

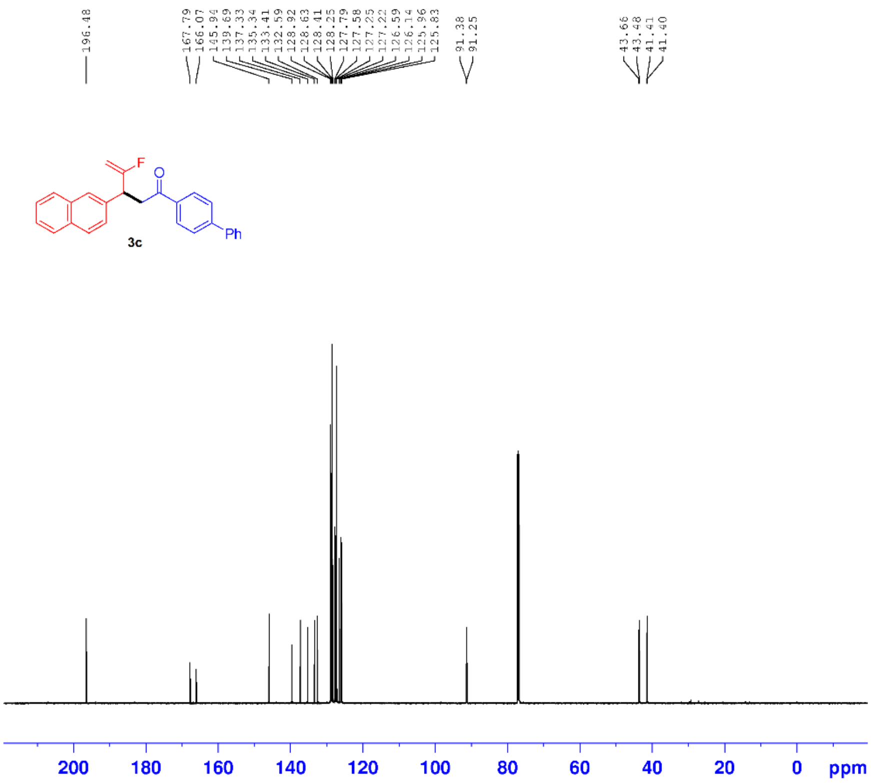
196.48
167.79
166.07
145.94
139.69
137.33
135.34
133.41
132.59
128.92
128.63
128.41
128.25
127.79
127.58
127.25
127.22
126.59
126.14
125.96
125.83
91.38
91.25
43.66
43.48
41.41
41.40
F
O
Ph
3c
200
180
160
140
120
100
80
60
40
20
0
ppm

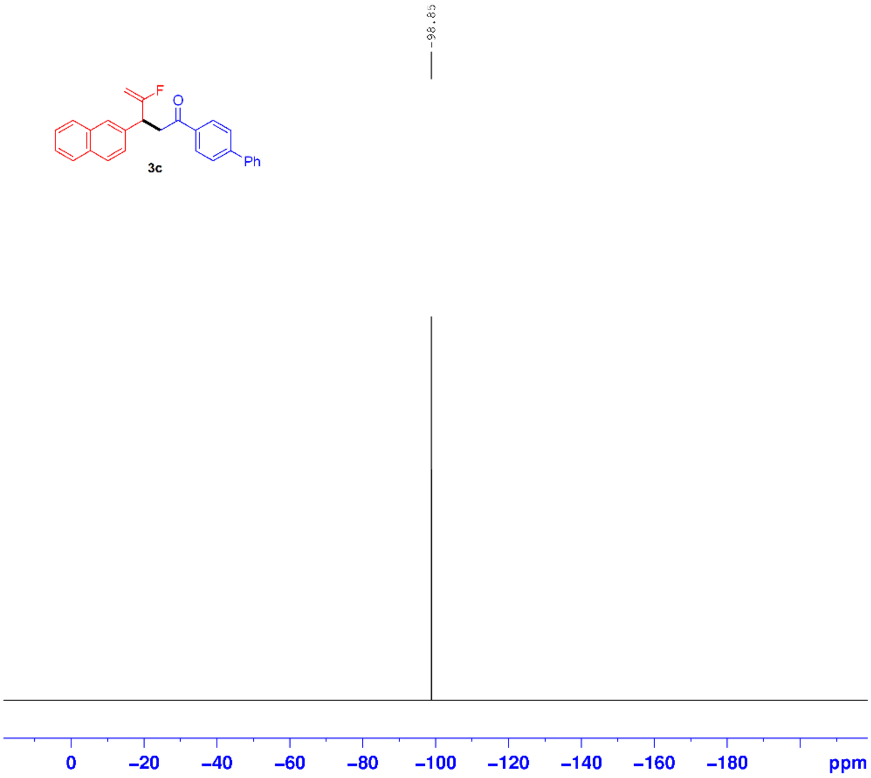

-98.85
F
O
Ph
3c
0
–20
–40
–60
–80
–100
–120
–140
–160
–180
ppm

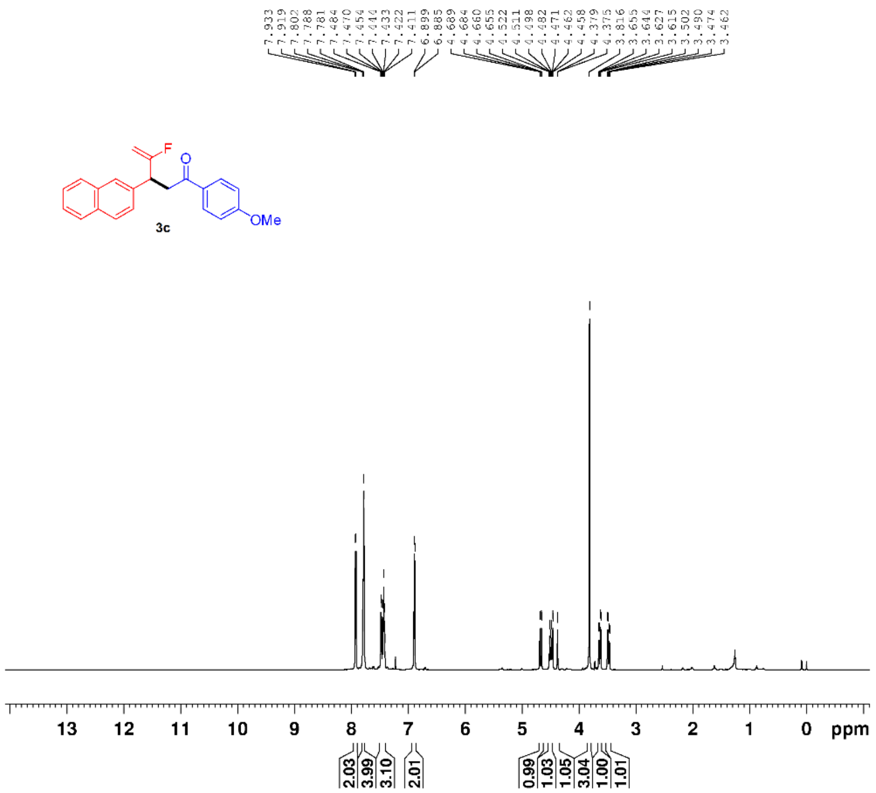

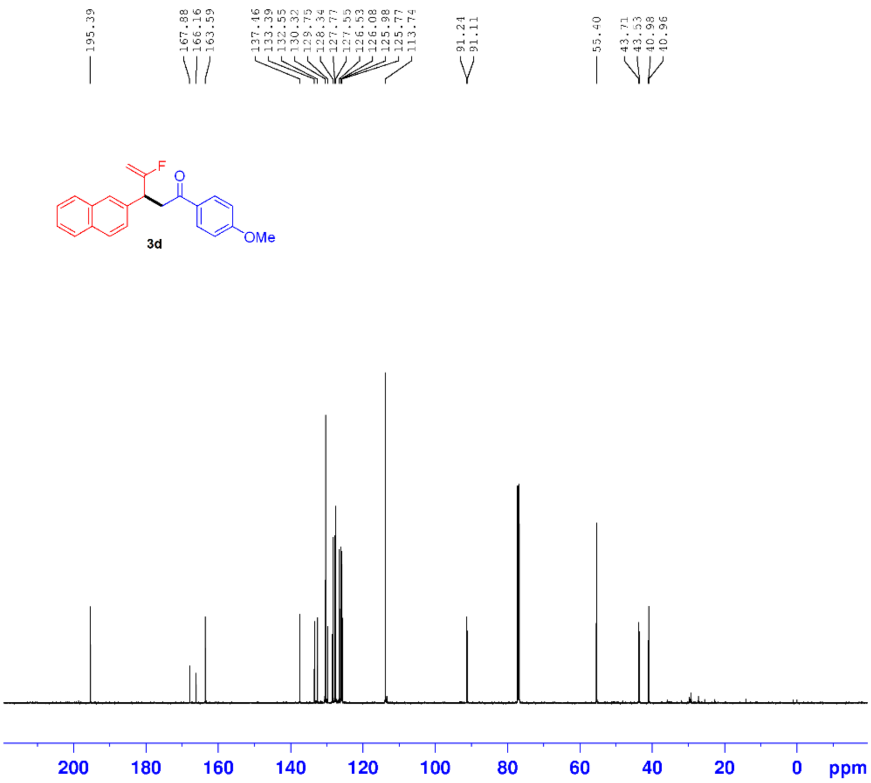
195.39
167.88
166.16
163.59
137.46
133.39
132.55
130.32
129.75
128.34
127.77
127.55
126.53
126.08
125.98
125.77
113.74
91.24
91.11
55.40
43.71
43.53
40.98
40.96
F
O
OMe
3d
200
180
160
140
120
100
80
60
40
20
0
ppm

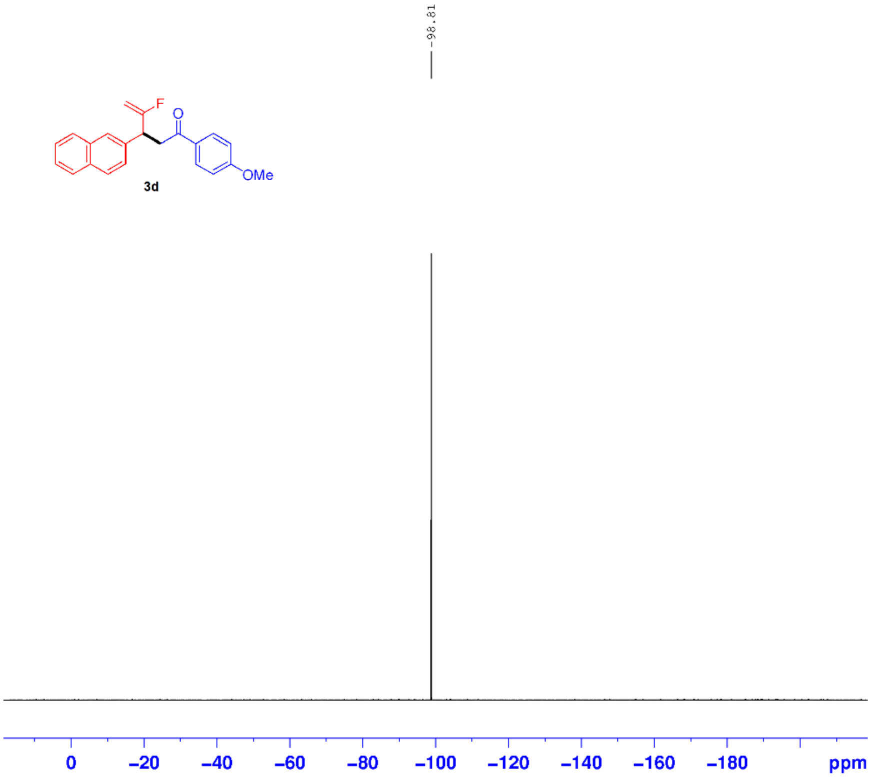
F
O
OMe
3d
-98.81
0
–20
–40
–60
–80
–100
–120
–140
–160
–180
ppm

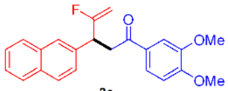

3e

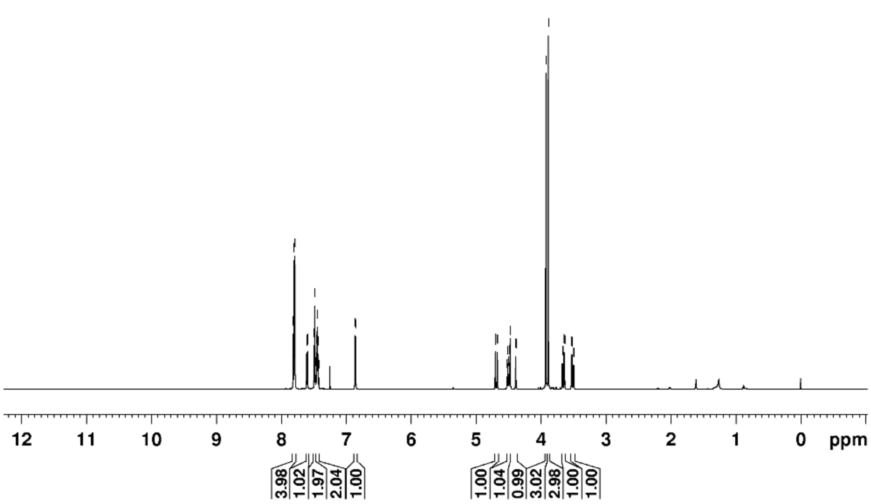

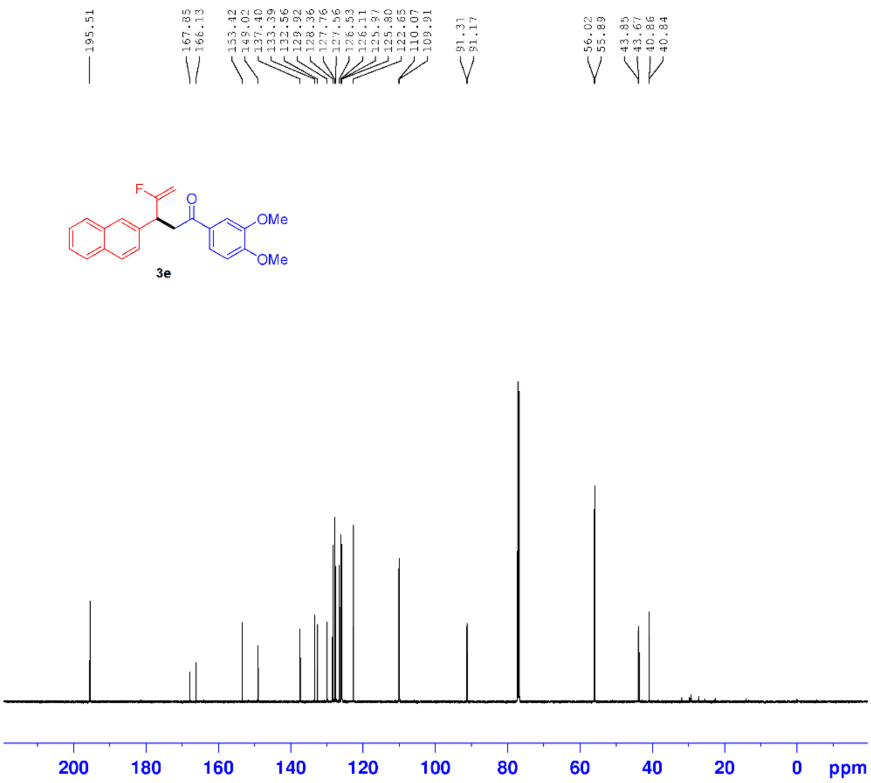

195.51
167.85
166.13
153.42
149.02
137.40
133.39
132.56
129.92
128.36
127.76
127.56
126.53
126.11
125.97
125.80
122.65
110.07
109.91
91.31
91.17
56.02
55.89
43.85
43.67
40.86
40.84
F
O
OMe
OMe
3e
200
180
160
140
120
100
80
60
40
20
0
ppm

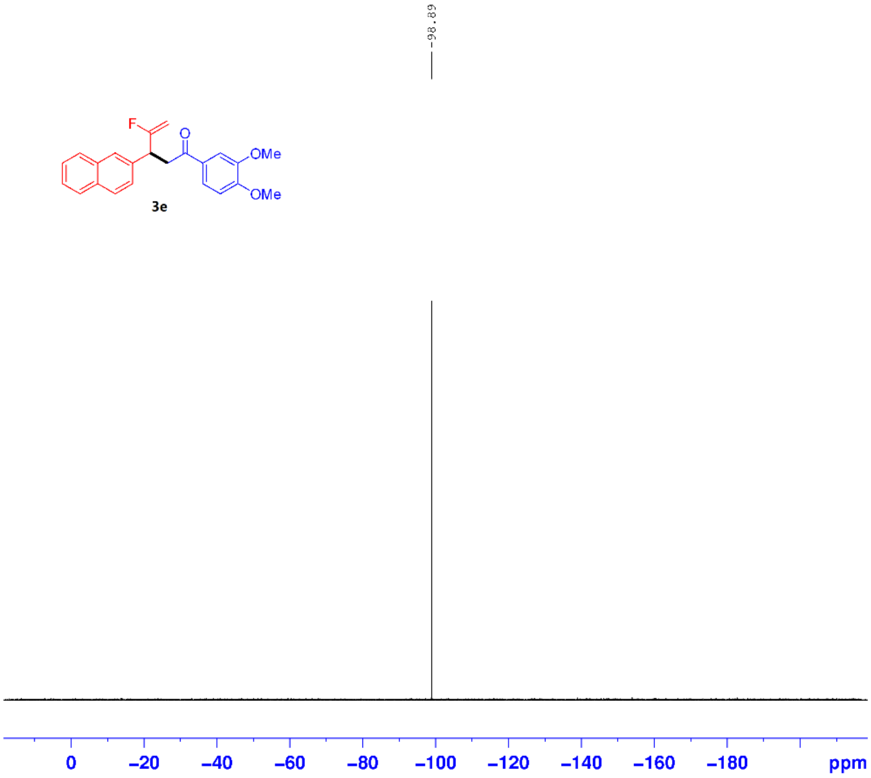
-98.89
F
O
OMe
OMe
3e
0
–20
–40
–60
–80
–100
–120
–140
–160
–180
ppm

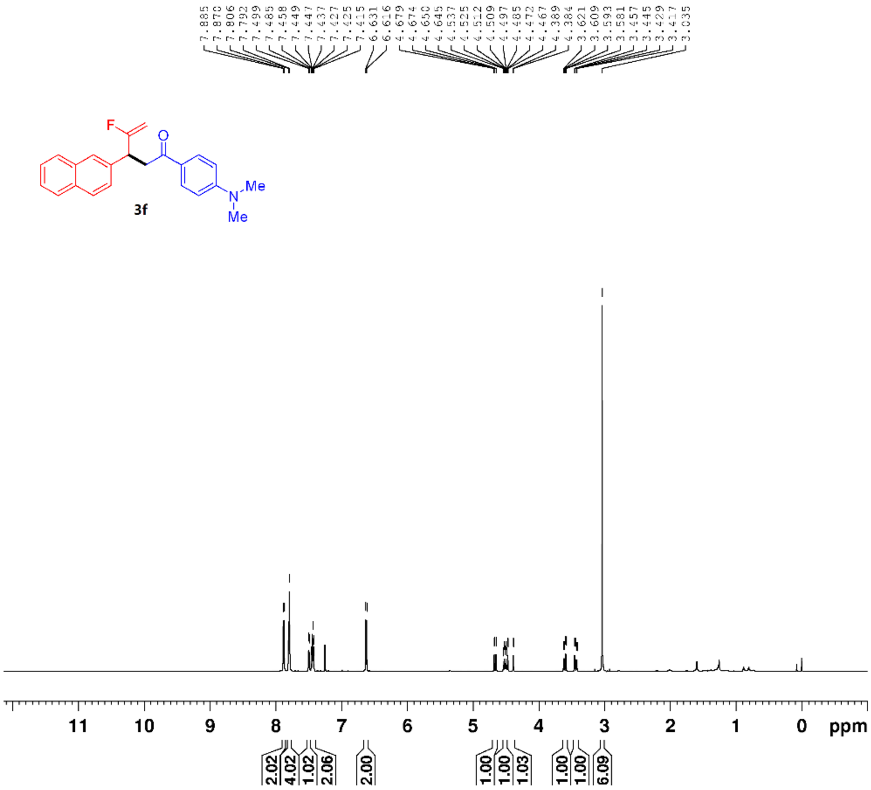
F
O
N
Me
Me
3f
7.885
7.870
7.806
7.792
7.499
7.485
7.458
7.449
7.447
7.437
7.427
7.425
7.415
6.631
6.616
4.679
4.674
4.650
4.645
4.537
4.525
4.512
4.509
4.497
4.485
4.472
4.467
4.389
4.384
3.621
3.609
3.593
3.581
3.457
3.445
3.429
3.417
3.035
11
10
9
8
7
6
5
4
3
2
1
0
ppm
2.02
4.02
1.02
2.06
2.00
1.00
1.00
1.03
1.00
1.00
6.09

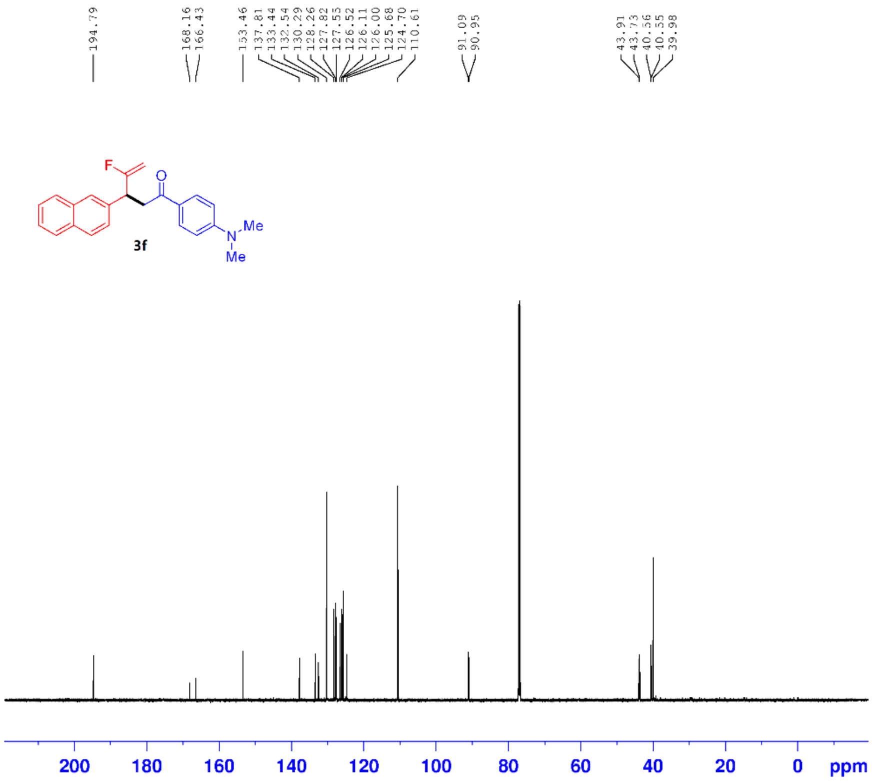

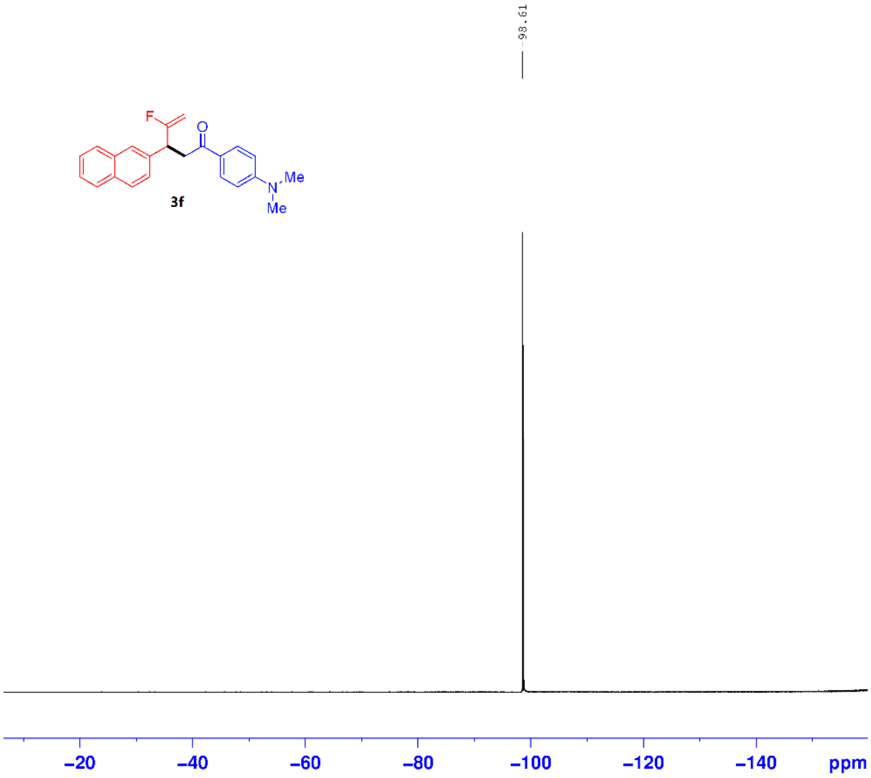
-98.61
F
O
N
Me
Me
3f
-20
-40
-60
-80
-100
-120
-140
ppm

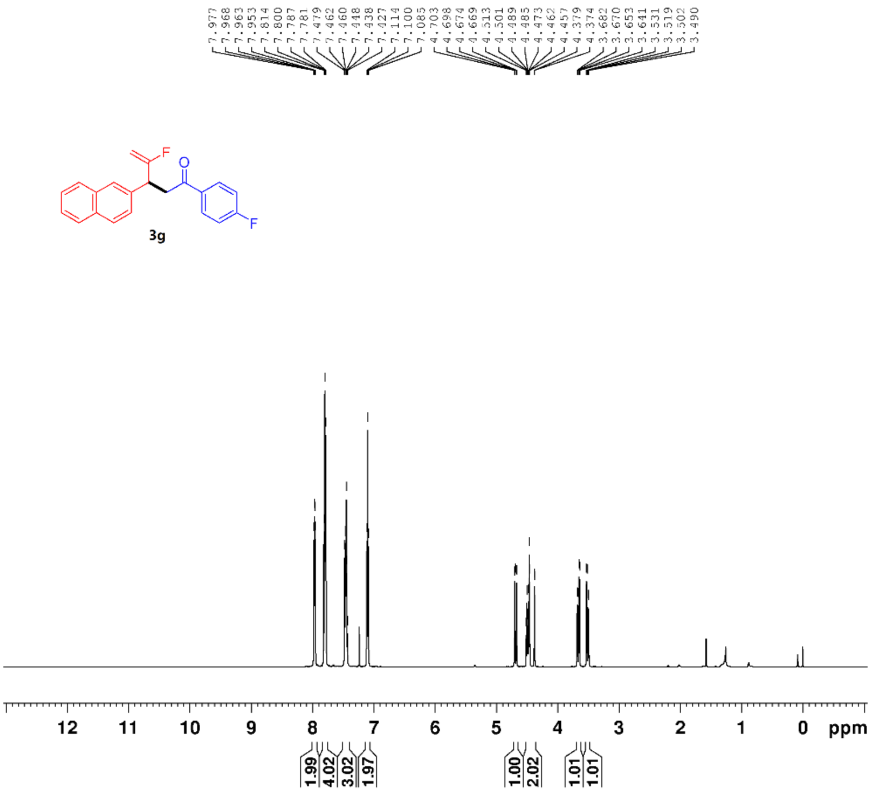
7.977
7.968
7.963
7.953
7.814
7.800
7.787
7.781
7.479
7.462
7.460
7.448
7.438
7.027
7.114
7.100
7.085
4.703
4.698
4.674
4.669
4.513
4.501
4.489
4.485
4.473
4.462
4.457
4.379
4.374
3.682
3.670
3.653
3.641
3.531
3.519
3.502
3.490
F
O
F
3g
12
11
10
9
8
7
6
5
4
3
2
1
0
ppm
1.99
4.02
3.02
1.97
1.00
2.02
1.01
1.01

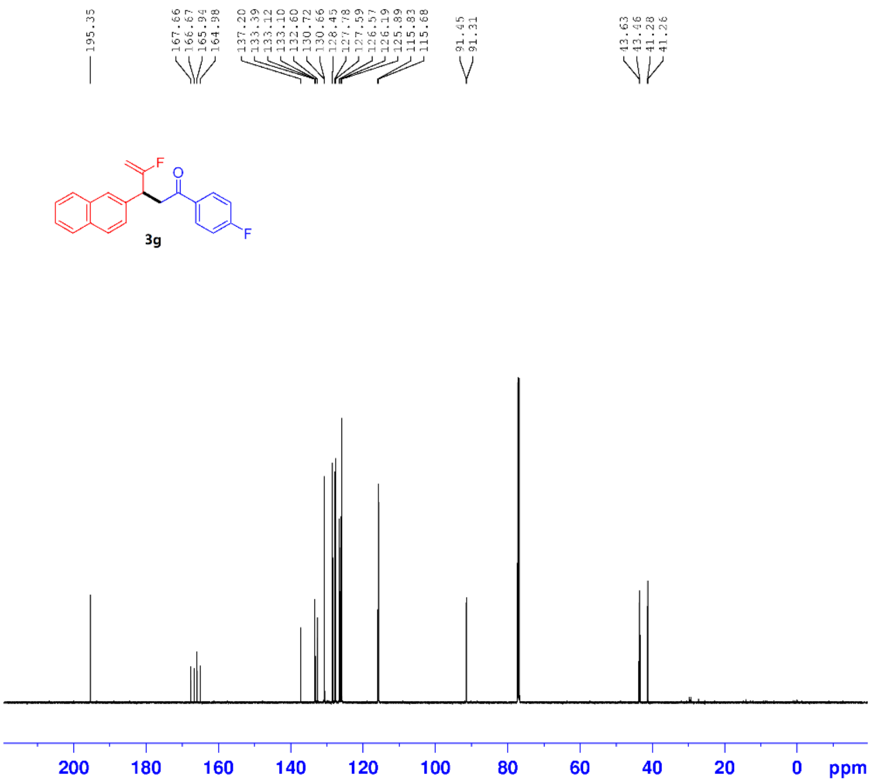
195.35
167.66
166.67
165.94
164.98
137.20
133.39
133.12
133.10
132.60
130.72
130.66
128.45
127.78
127.59
126.57
126.19
125.89
115.83
115.68
91.45
91.31
43.63
43.46
41.28
41.26
F
O
F
3g
200
180
160
140
120
100
80
60
40
20
0
ppm

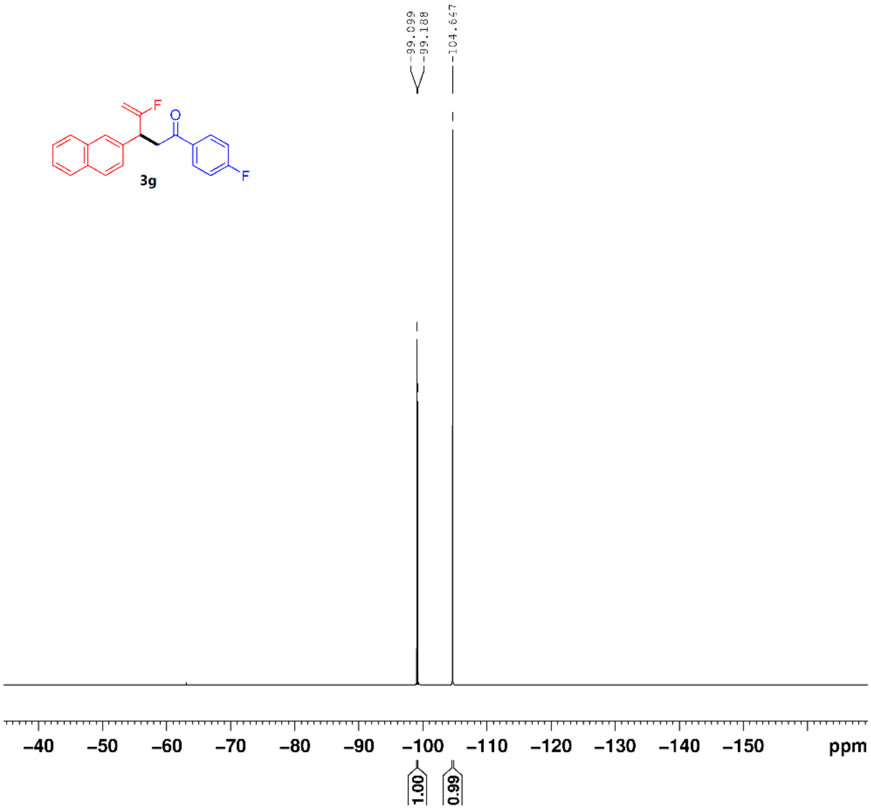
3g
-99.099
-99.188
-104.647
-40
-50
-60
-70
-80
-90
-100
-110
-120
-130
-140
-150
ppm
1.00
0.99

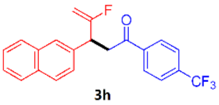

3h

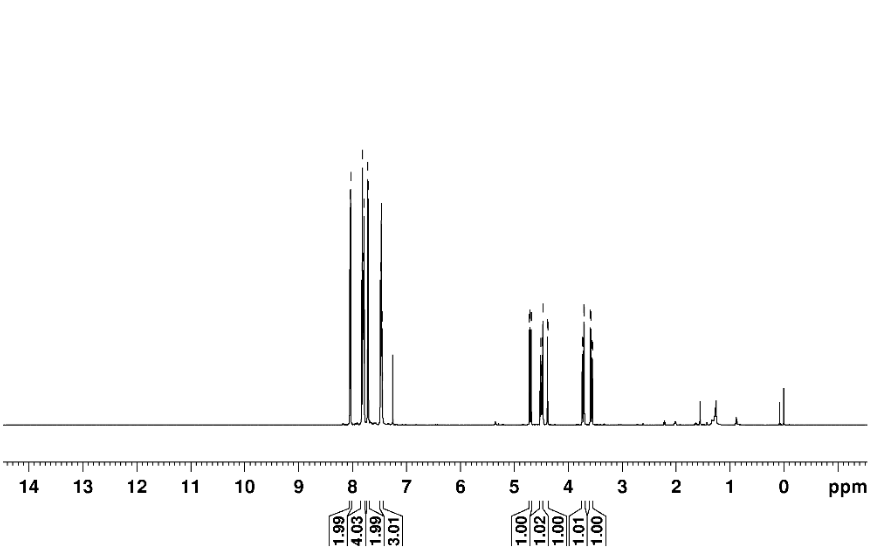

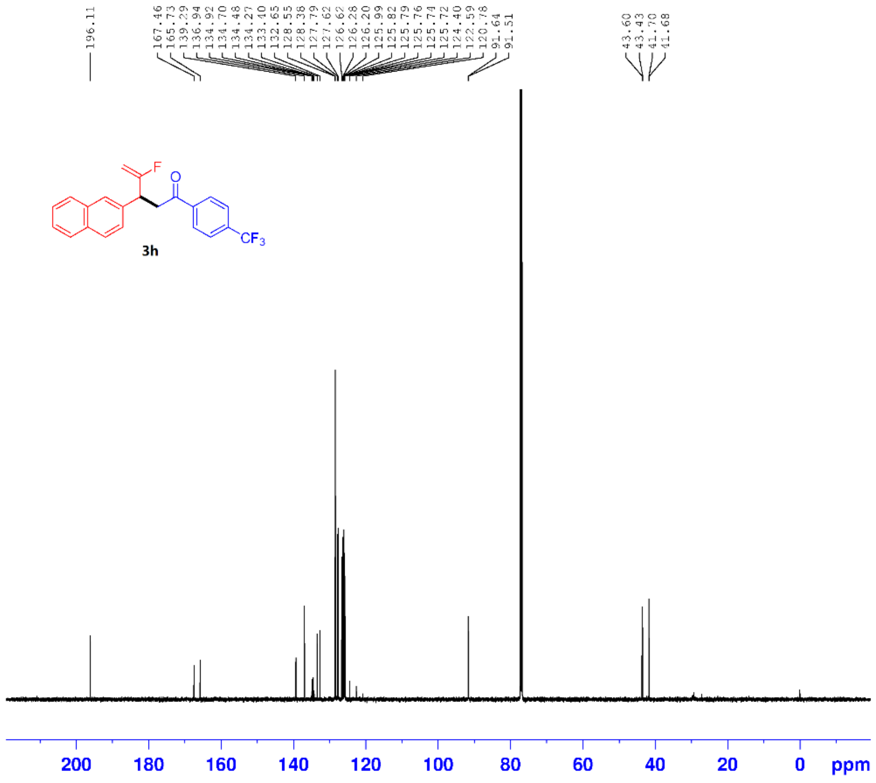
196.11
167.46
165.73
139.29
136.94
134.92
134.70
134.48
134.27
133.40
132.65
128.55
128.38
127.79
127.62
126.62
126.28
126.20
125.99
125.82
125.79
125.76
125.74
125.72
124.40
122.59
120.78
91.64
91.51
43.60
43.43
41.70
41.68
F
O
CF3
3h
200
180
160
140
120
100
80
60
40
20
0
ppm

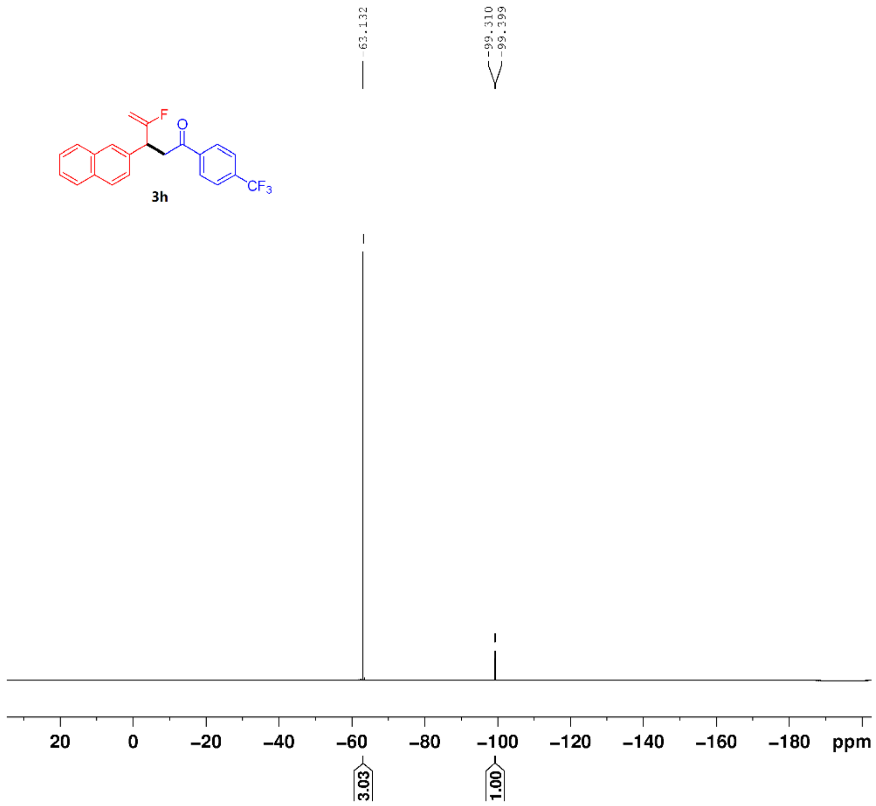
-63.132
-99.310
-99.399
F
O
CF3
3h
20
0
−20
−40
−60
−80
−100
−120
−140
−160
−180
ppm
3.03
1.00

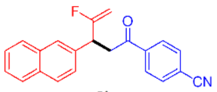
F
O
CN

3i

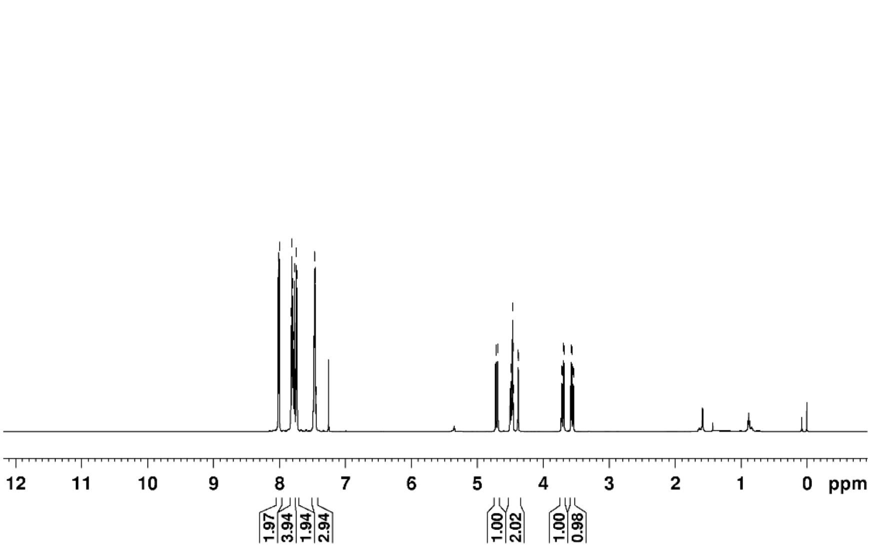
8.016
8.003
7.825
7.811
7.801
7.798
7.787
7.771
7.746
7.733
7.484
7.469
7.465
7.460
7.456
7.454
7.445
4.720
4.715
4.691
4.686
4.504
4.492
4.480
4.476
4.465
4.460
4.453
4.382
4.377
3.723
3.712
3.694
3.682
3.579
3.566
3.549
3.537
12
11
10
9
8
7
6
5
4
3
2
1
0
ppm
1.97
3.94
1.94
2.94
1.00
2.02
1.00
0.98

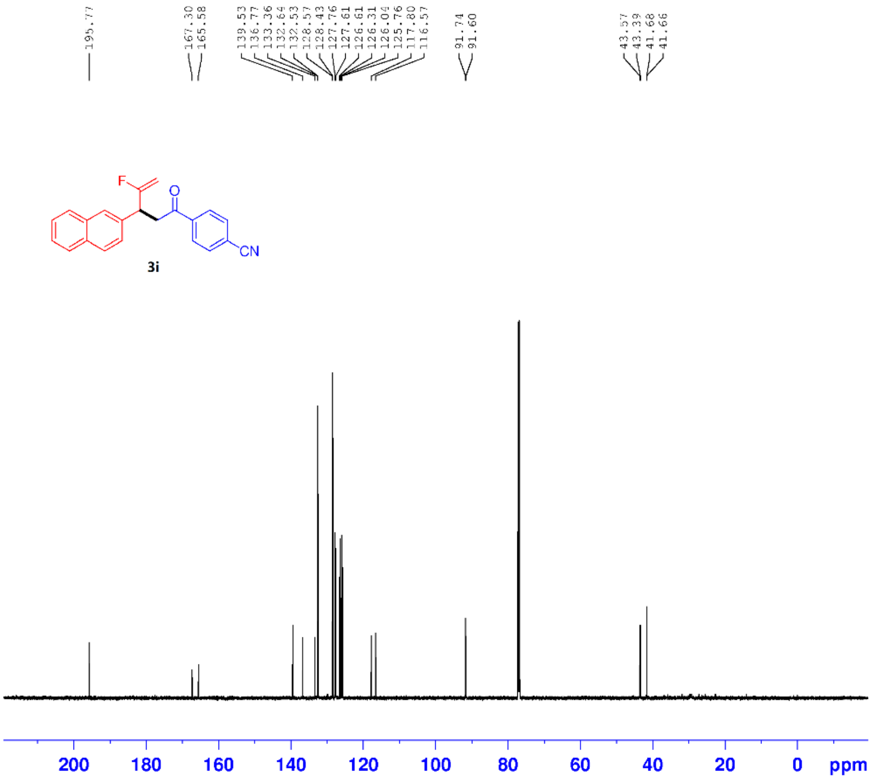
195.77
167.30
165.58
139.53
136.77
133.36
132.64
132.53
128.57
128.43
127.76
127.61
126.61
126.31
126.04
125.76
117.80
116.57
91.74
91.60
43.57
43.39
41.68
41.66
F
O
CN
3i
200
180
160
140
120
100
80
60
40
20
0
ppm

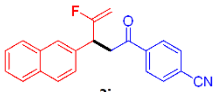

3i

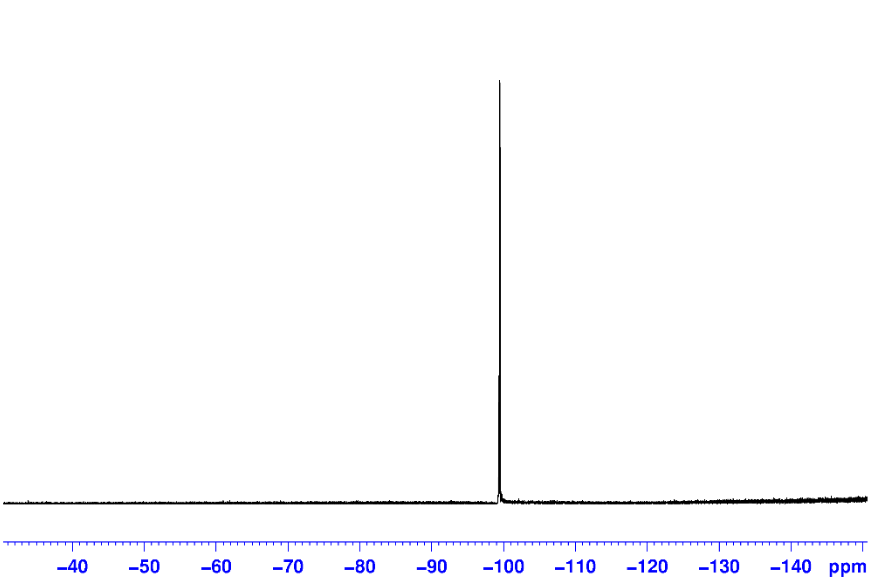

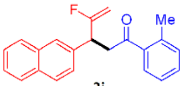
F
O
Me

3j

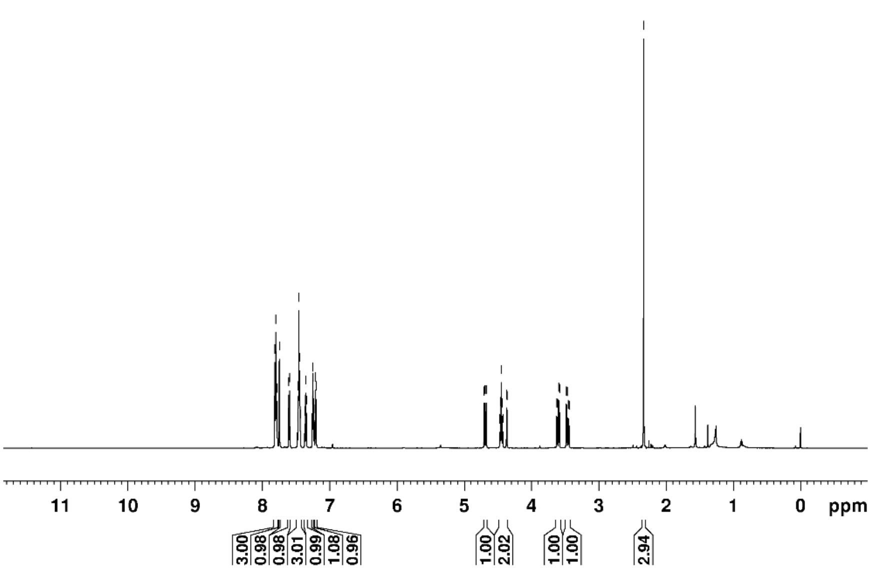
7.815
7.802
7.792
7.781
7.748
7.609
7.596
7.476
7.468
7.465
7.457
7.452
7.448
7.445
7.437
7.370
7.358
7.345
7.258
7.251
7.245
7.233
7.213
7.200
4.704
4.699
4.675
4.670
4.473
4.460
4.452
4.447
4.434
4.422
4.370
4.364
3.625
3.613
3.596
3.585
3.483
3.471
3.455
3.443
2.331
11
10
9
8
7
6
5
4
3
2
1
0
ppm
3.00
0.98
0.98
3.01
0.99
1.08
0.96
1.00
2.02
1.00
1.00
2.94

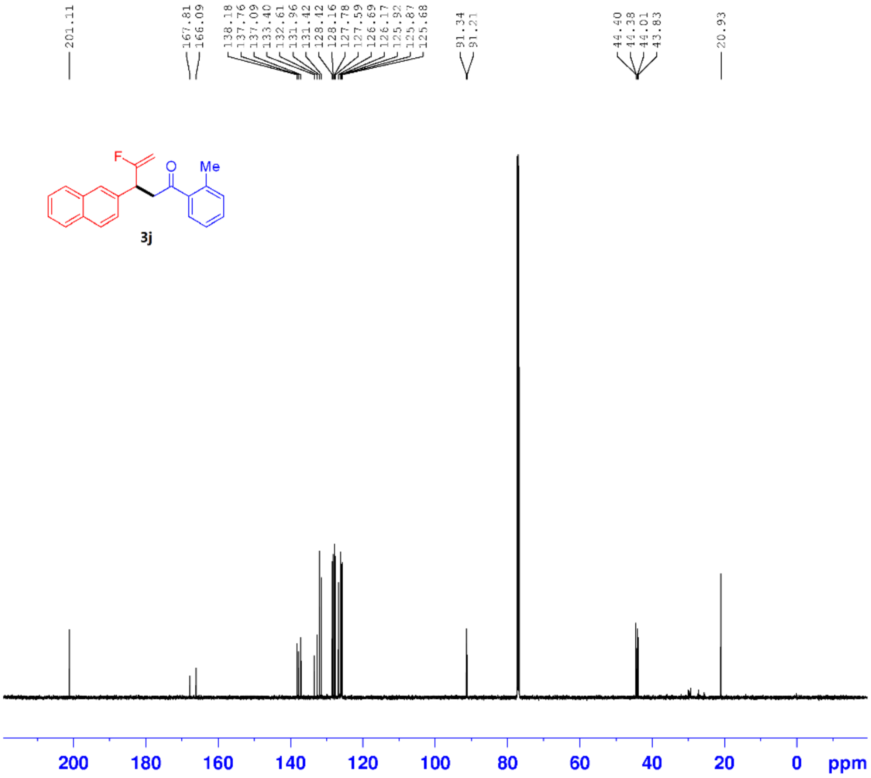
201.11
167.81
166.09
138.18
137.76
137.09
133.40
132.61
131.96
131.42
128.42
128.16
127.78
127.59
126.69
126.17
125.92
125.87
125.68
91.34
91.21
44.40
44.38
44.01
43.83
20.93
F
O
Me
3j
200
180
160
140
120
100
80
60
40
20
0
ppm

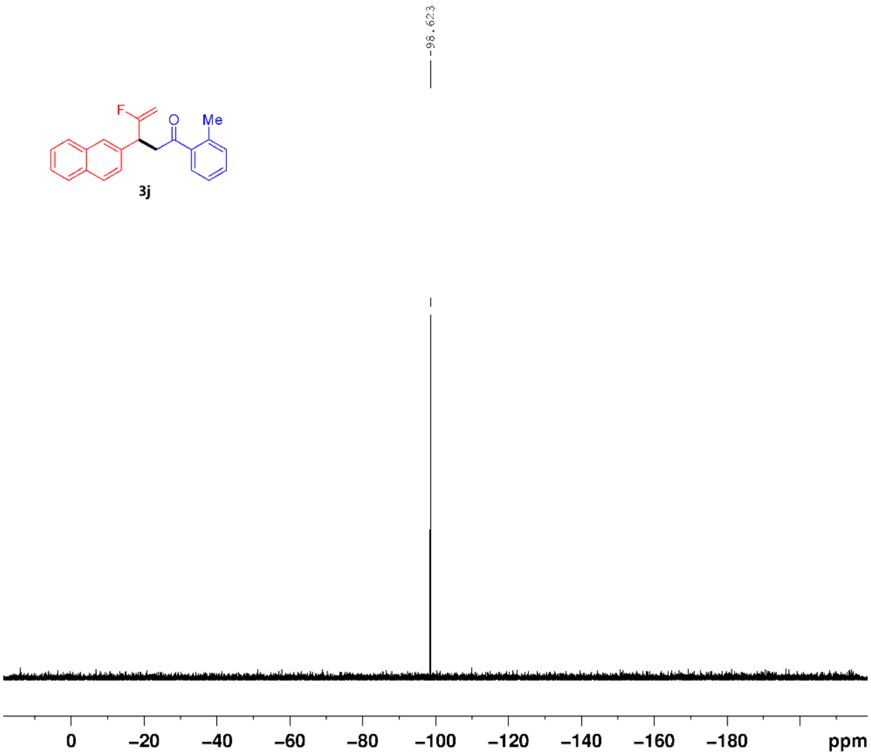
-98.623
F
O
Me
3j
0
–20
–40
–60
–80
–100
–120
–140
–160
–180
ppm

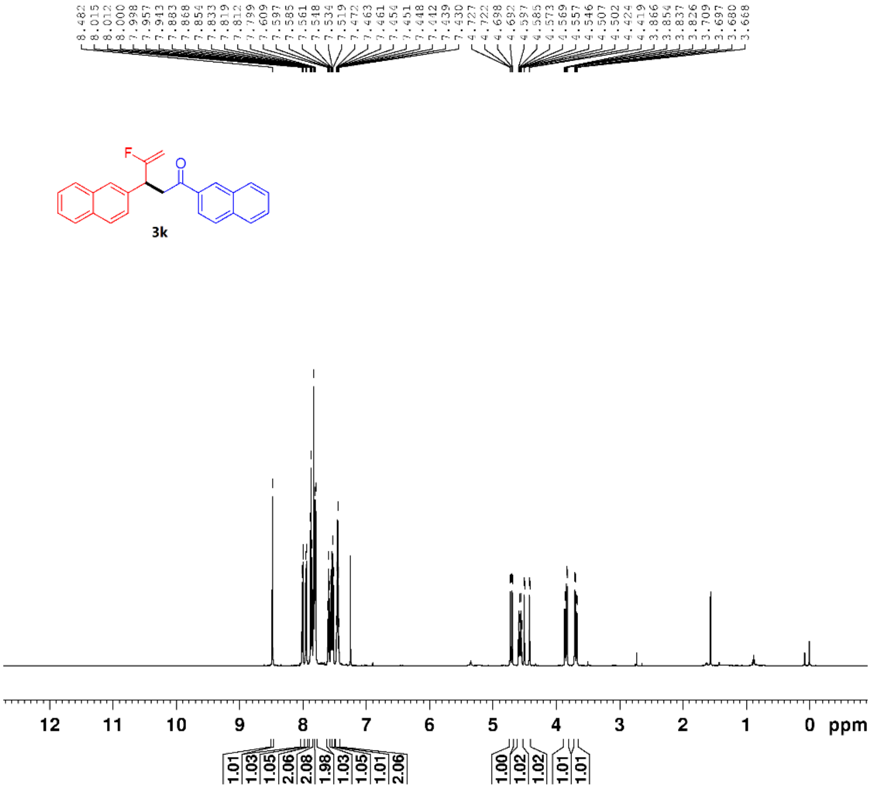

3k

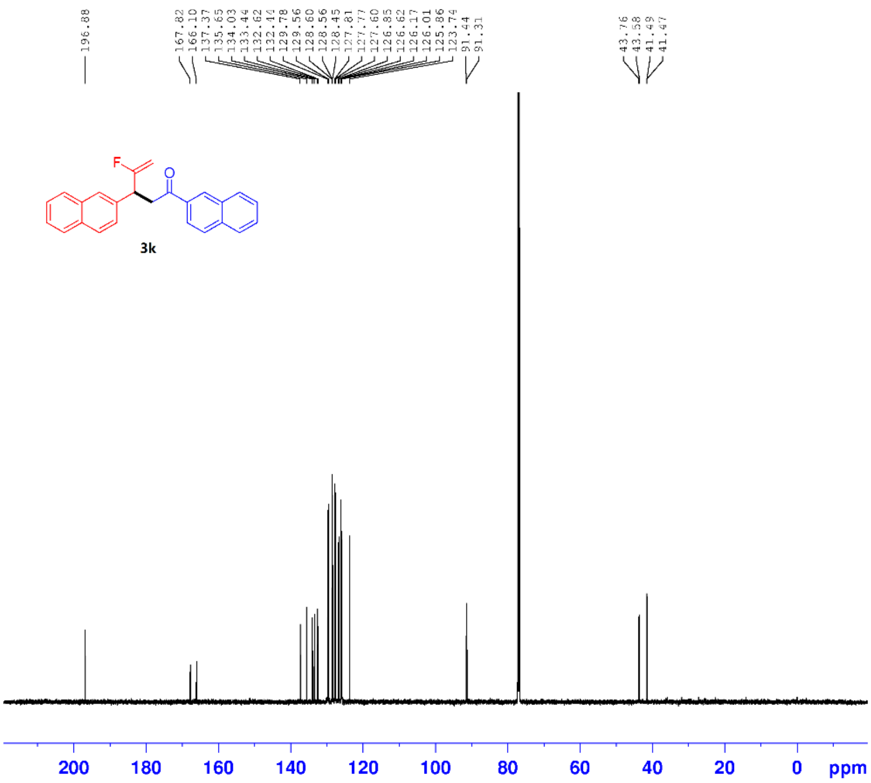
196.88
167.82
166.10
137.37
135.65
134.03
133.44
132.62
132.14
129.78
129.56
128.60
128.56
128.45
127.81
127.77
127.60
126.85
126.62
126.17
126.01
125.86
123.74
91.44
91.31
43.76
43.58
41.49
41.47
F
O
3k
200
180
160
140
120
100
80
60
40
20
0
ppm

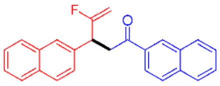

3k

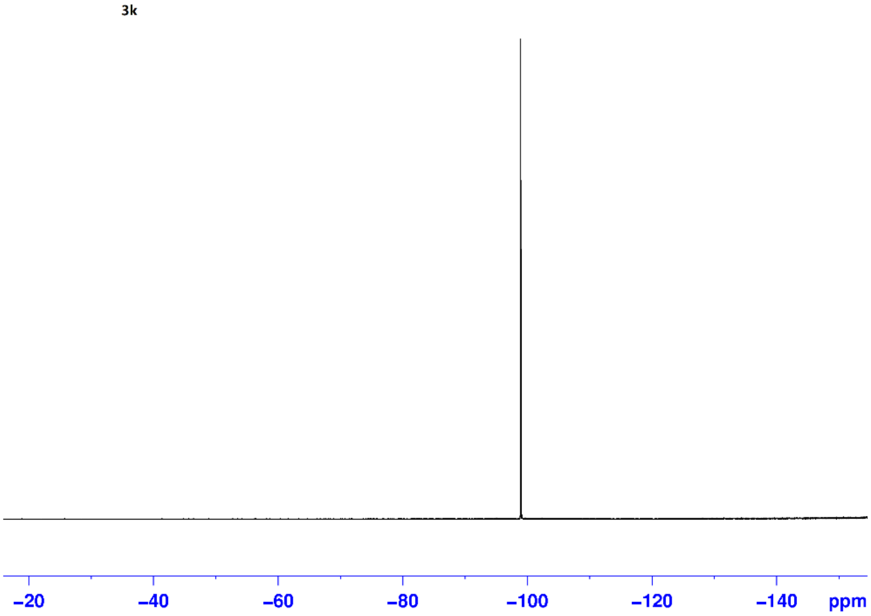

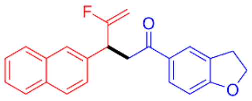
F
O
O

3l

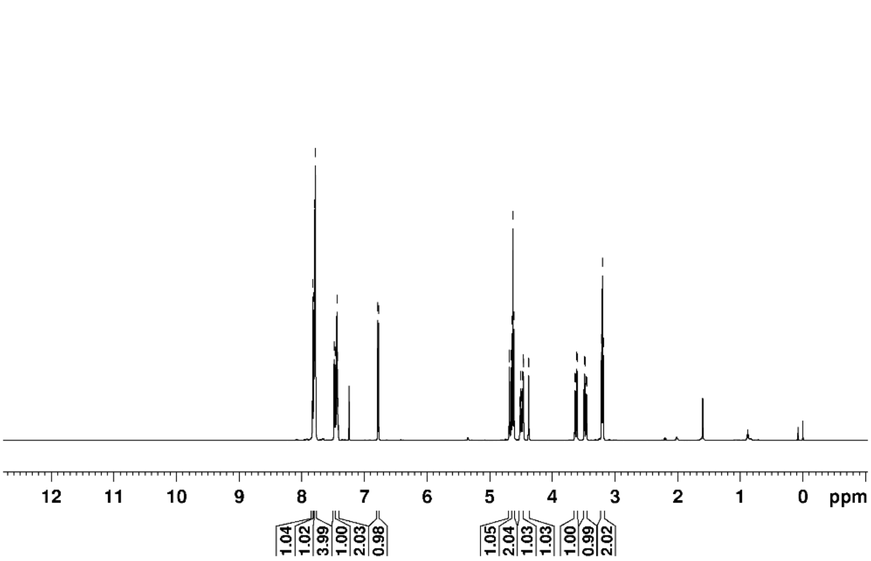
7.827
7.814
7.808
7.799
7.795
7.784
7.485
7.472
7.470
7.463
7.454
7.442
7.430
7.421
6.790
6.776
4.690
4.685
4.661
4.656
4.643
4.628
4.614
4.521
4.510
4.498
4.494
4.482
4.470
4.465
4.460
4.382
4.377
3.643
3.632
3.615
3.603
3.493
3.481
3.465
3.453
3.217
3.202
3.188
12 11 10 9 8 7 6 5 4 3 2 1 0 ppm
1.04
1.02
3.99
1.00
2.03
0.98
1.05
2.04
1.03
1.03
1.00
0.99
2.02

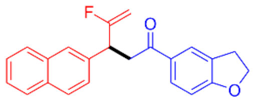

3l

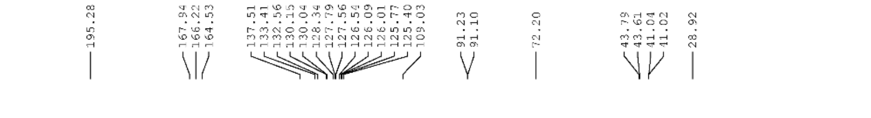

195.28
167.94
166.22
164.53
137.51
133.41
132.56
130.15
130.04
128.34
127.79
127.56
126.56
126.09
126.01
125.77
125.40
109.03
91.23
91.10
72.20
43.79
43.61
41.04
41.02
28.92
200
180
160
140
120
100
80
60
40
20
0
ppm

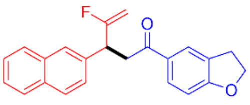

3l

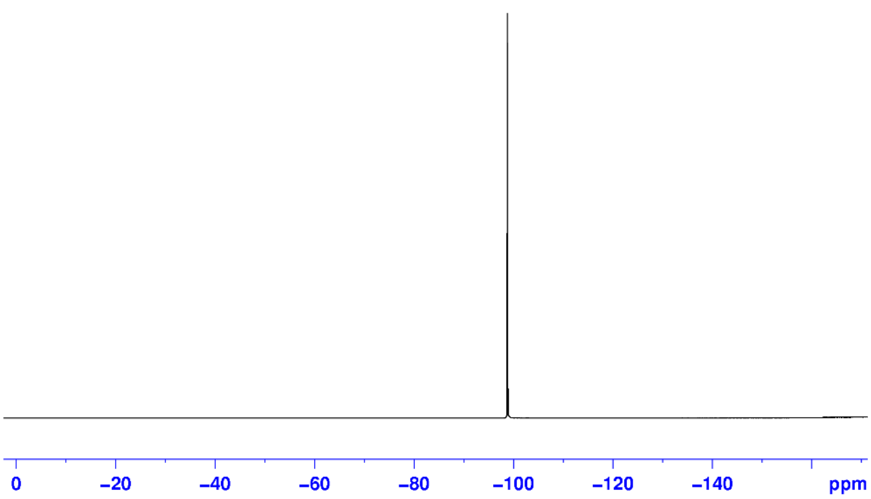

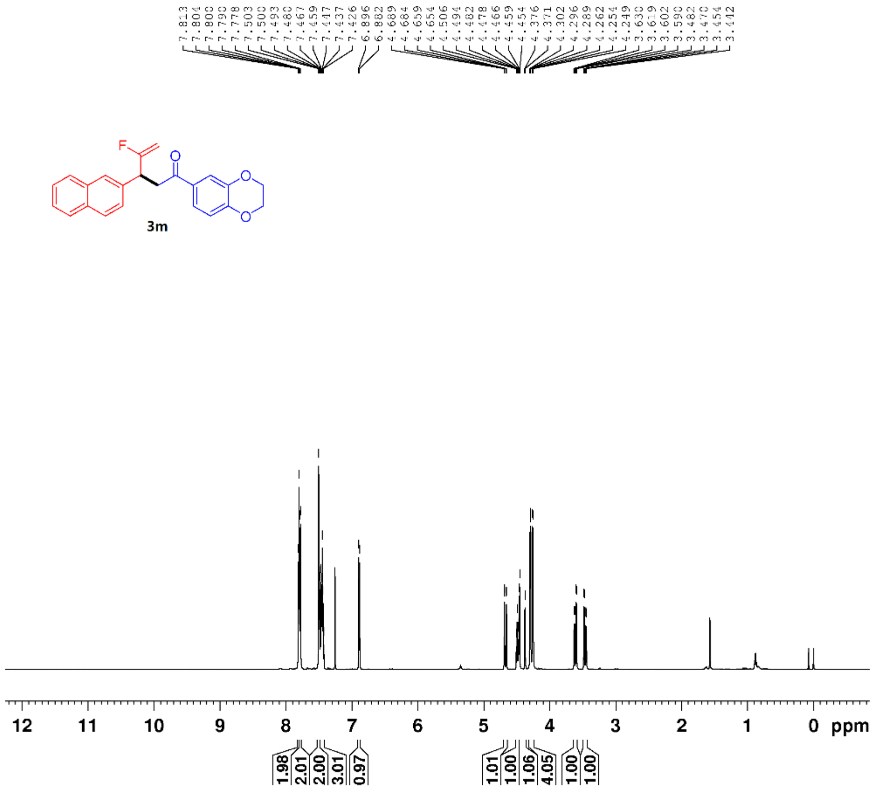
7.813
7.804
7.800
7.790
7.778
7.503
7.500
7.493
7.480
7.467
7.459
7.447
7.437
7.426
6.896
6.882
4.689
4.684
4.659
4.654
4.506
4.494
4.482
4.478
4.466
4.459
4.454
4.376
4.371
4.302
4.296
4.289
4.262
4.254
4.249
3.630
3.619
3.602
3.590
3.482
3.470
3.454
3.442
F
O
O
O
3m
12
11
10
9
8
7
6
5
4
3
2
1
0
ppm
1.98
2.01
2.00
3.01
0.97
1.01
1.00
1.06
4.05
1.00
1.00

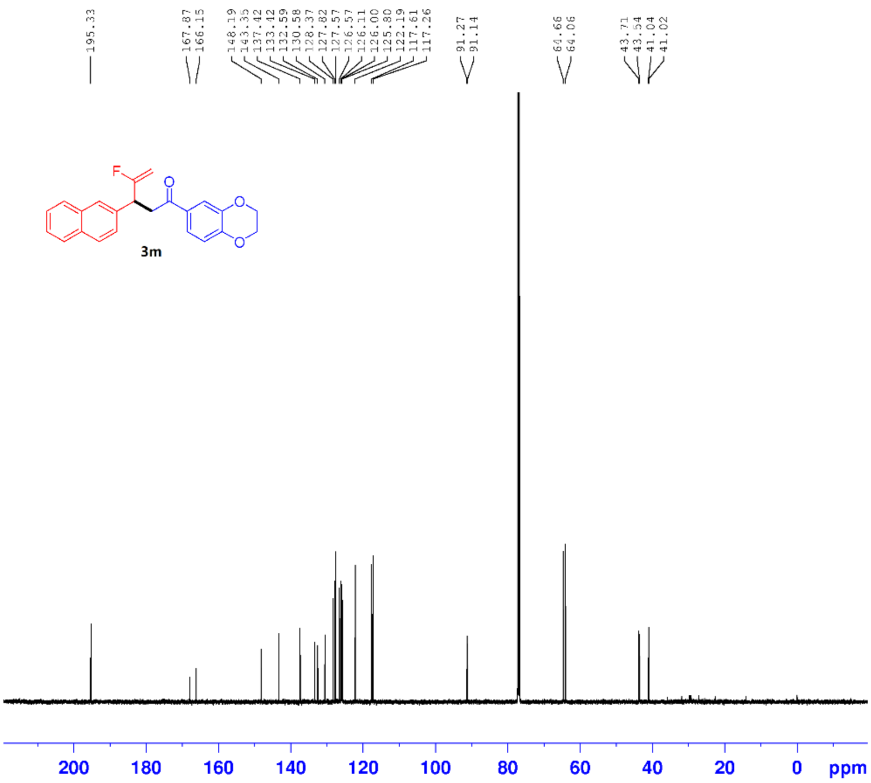
195.33
167.87
166.15
148.19
143.35
137.42
133.12
132.59
130.58
128.37
127.82
127.57
126.57
126.11
126.00
125.80
122.19
117.61
117.26
91.27
91.14
64.66
64.06
43.71
43.54
41.04
41.02
F
O
3m
200
180
160
140
120
100
80
60
40
20
0
ppm

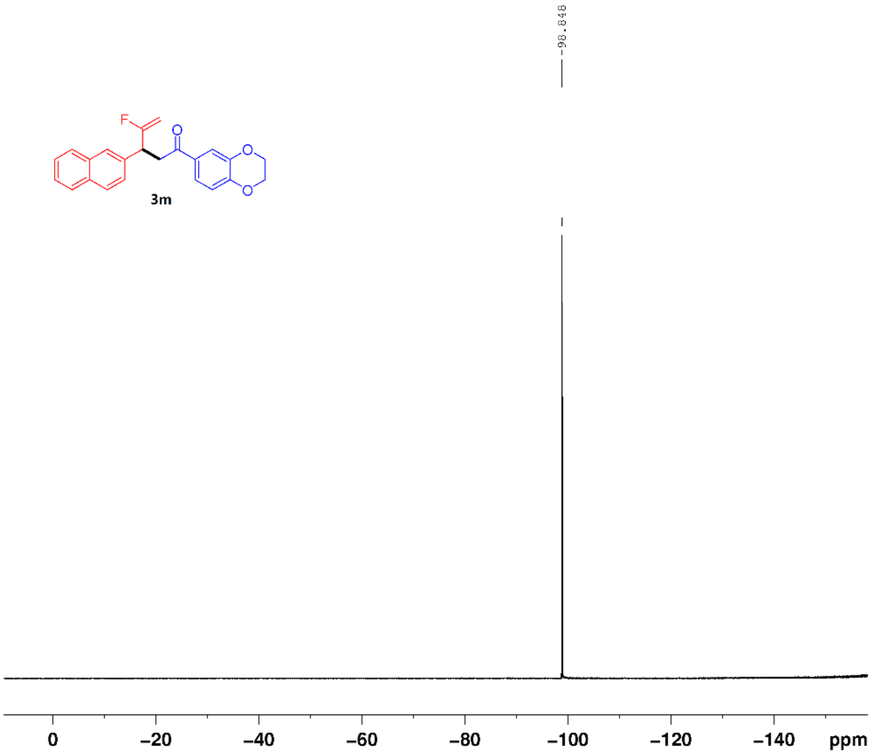
3m
-98.848
0
−20
−40
−60
−80
−100
−120
−140
ppm

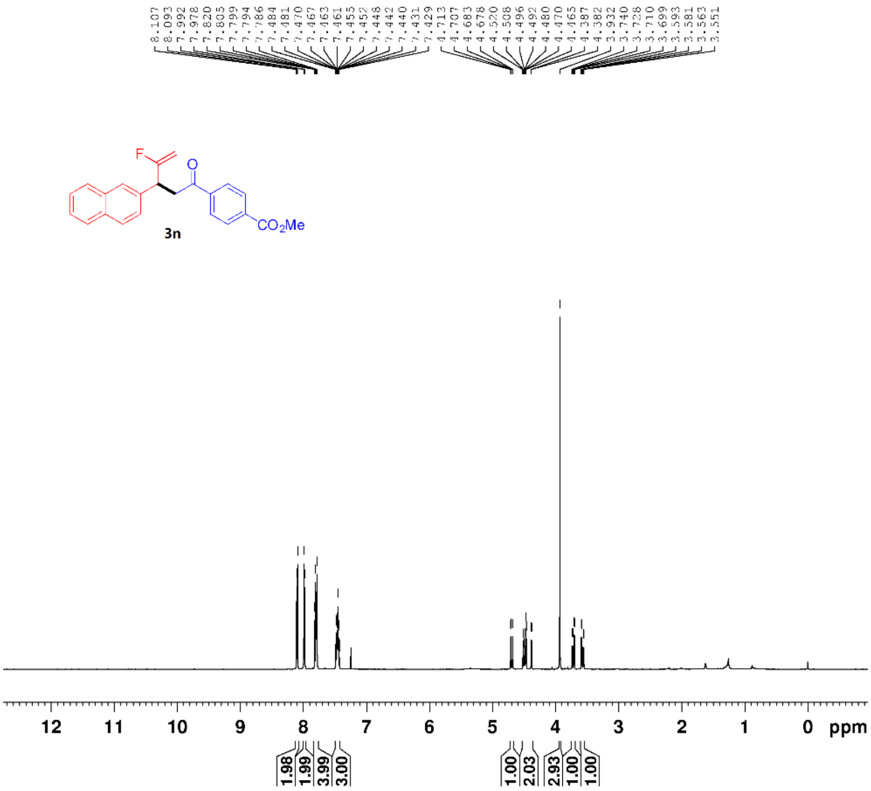
8.107
8.093
7.992
7.978
7.820
7.805
7.799
7.794
7.786
7.484
7.481
7.470
7.467
7.463
7.461
7.455
7.452
7.448
7.442
7.440
7.431
7.429
4.713
4.707
4.683
4.678
4.520
4.508
4.496
4.492
4.480
4.470
4.465
4.387
4.382
3.932
3.740
3.728
3.710
3.699
3.593
3.581
3.563
3.551
F
O
CO2Me
3n
12
11
10
9
8
7
6
5
4
3
2
1
0
ppm
1.98
1.99
3.99
3.00
1.00
2.03
2.93
1.00
1.00

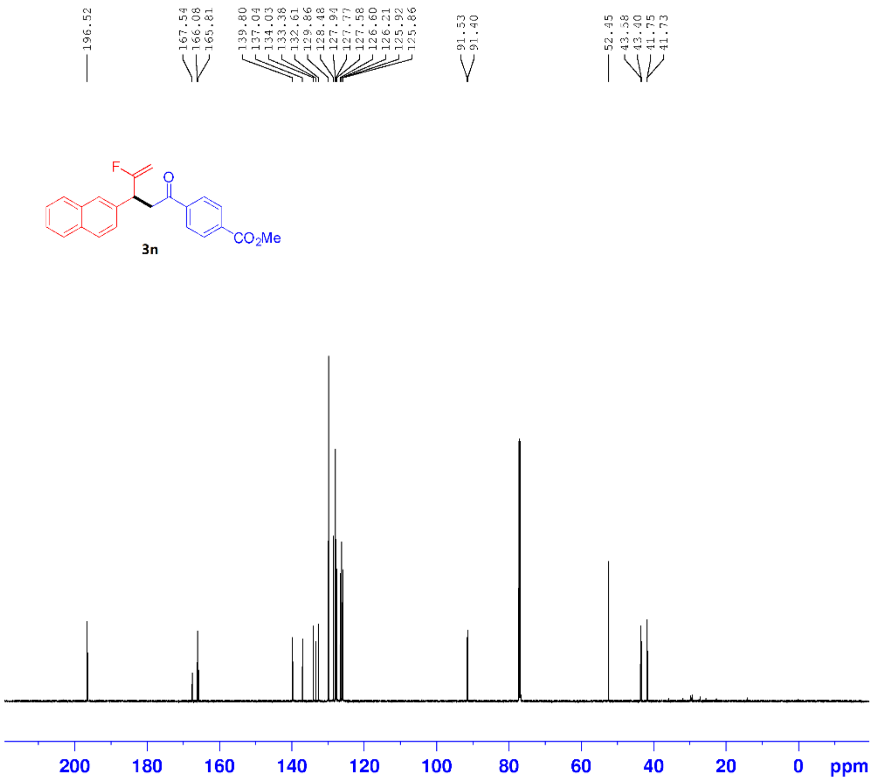
196.52
167.54
166.08
165.81
139.80
137.04
134.03
133.38
132.61
129.86
128.48
127.94
127.77
127.58
126.60
126.21
125.92
125.86
91.53
91.40
52.45
43.58
43.40
41.75
41.73
F
O
CO_2Me
3n
200
180
160
140
120
100
80
60
40
20
0
ppm

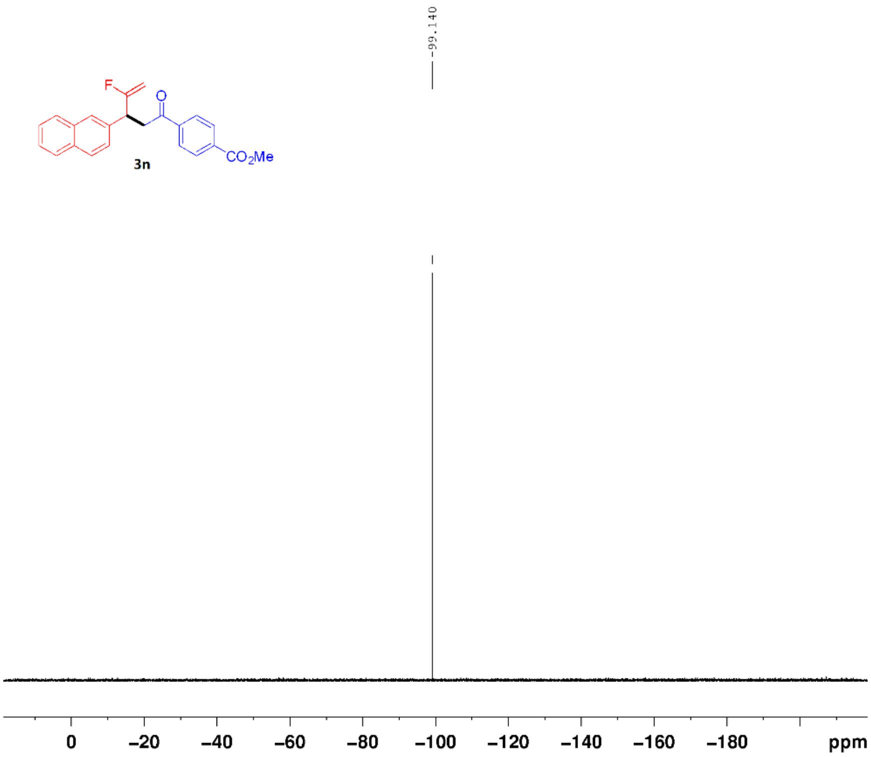
-99.140
F
O
CO2Me
3n
0
-20
-40
-60
-80
-100
-120
-140
-160
-180
ppm

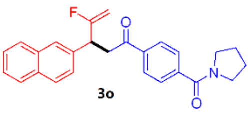

3o

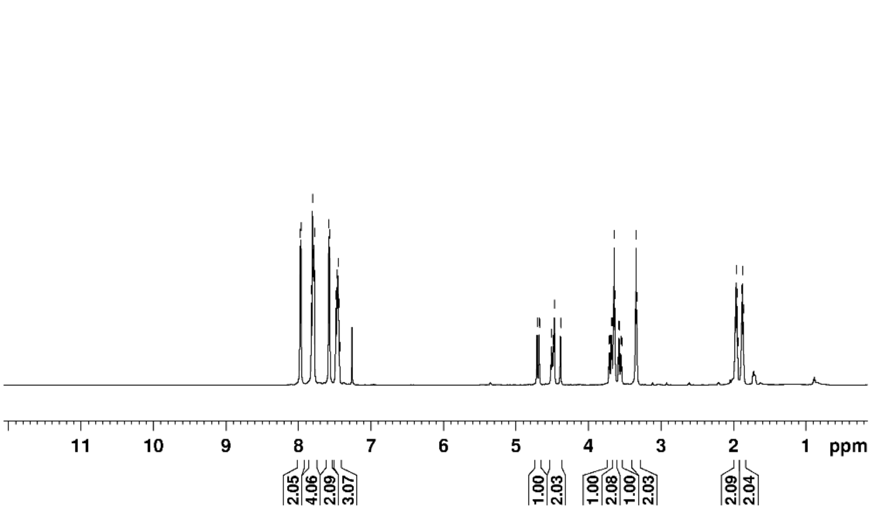

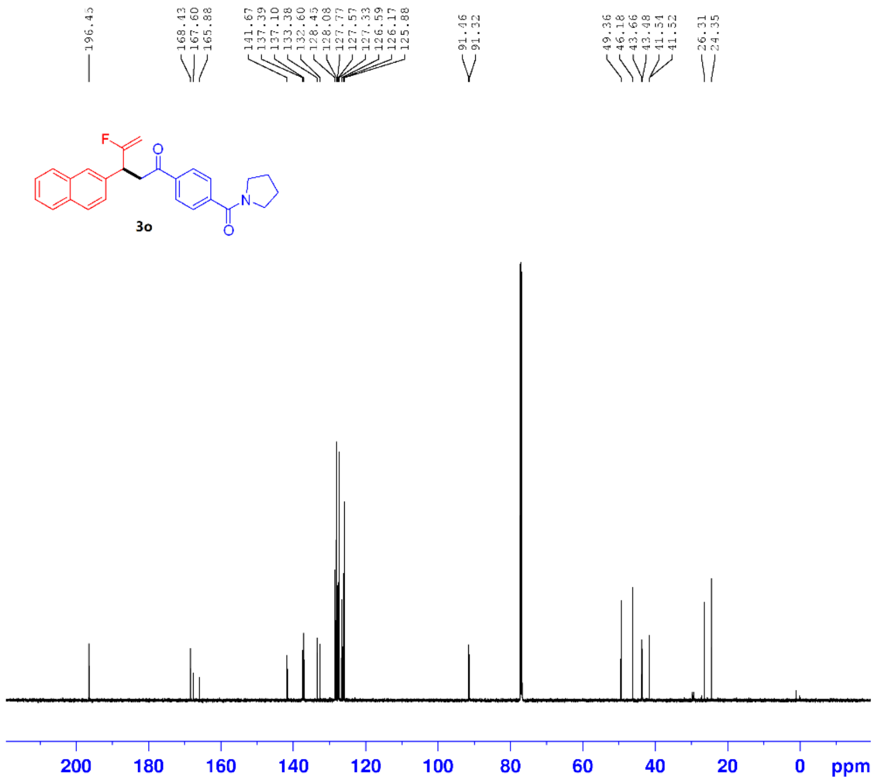

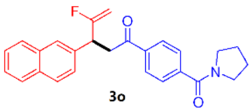

3o

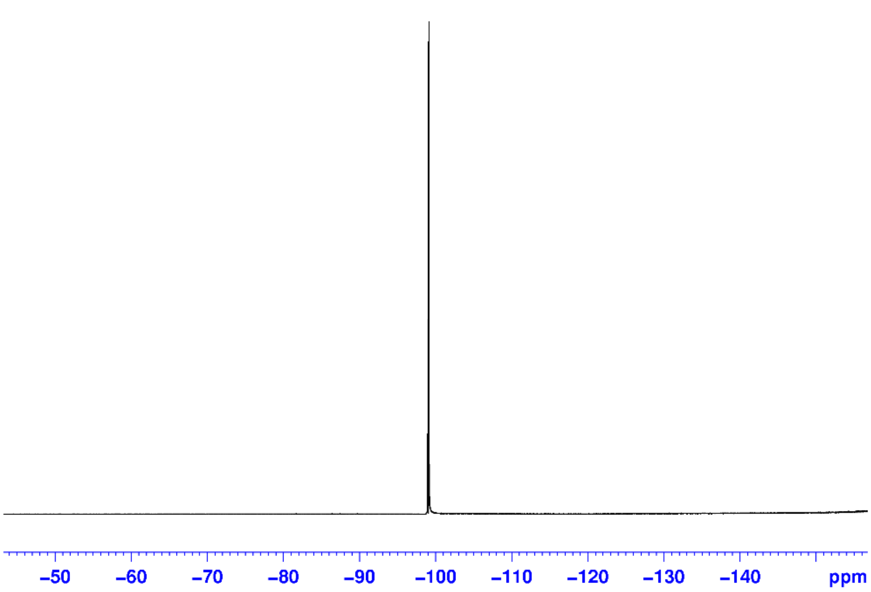

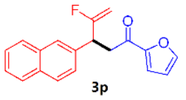
F
O
O

3p

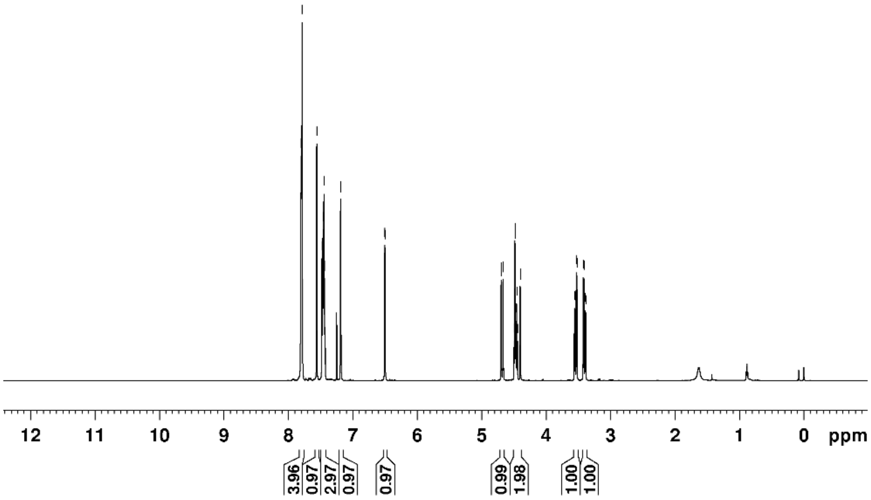
7.809
7.802
7.795
7.786
7.559
7.478
7.461
7.448
7.439
7.437
7.427
7.189
7.183
6.503
6.498
4.697
4.692
4.668
4.663
4.495
4.484
4.481
4.469
4.455
4.443
4.402
4.397
3.559
3.547
3.531
3.519
3.423
3.410
3.395
3.382
12
11
10
9
8
7
6
5
4
3
2
1
0
ppm
3.96
0.97
2.97
0.97
0.97
0.99
1.98
1.00
1.00

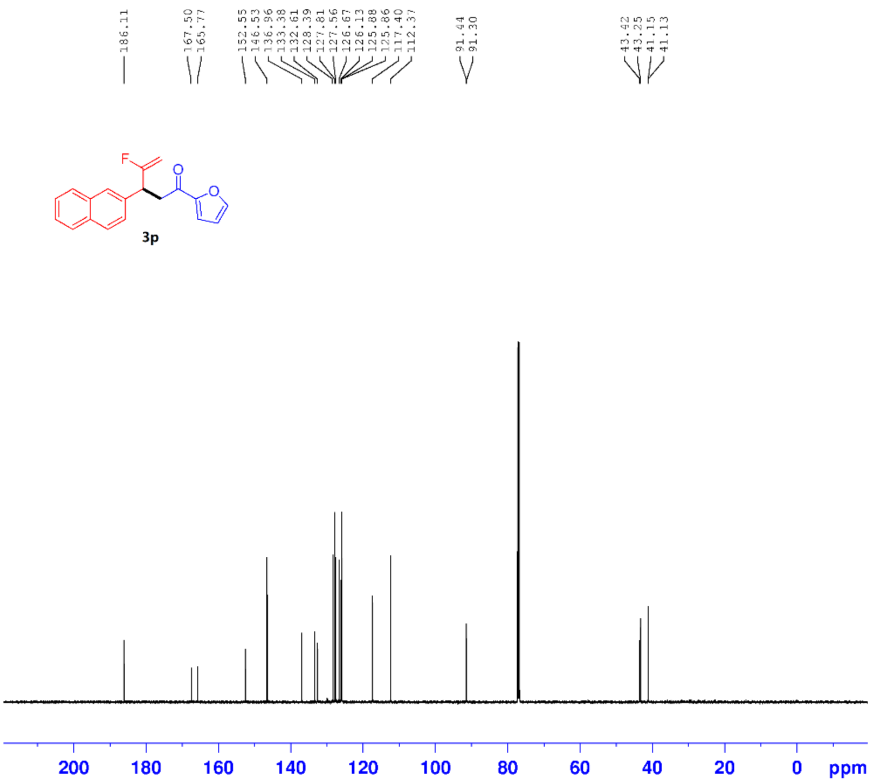

186.11
167.50
165.77
152.55
146.53
136.96
133.38
132.61
128.39
127.81
127.56
126.67
126.13
125.88
125.86
117.40
112.37
91.44
91.30
43.42
43.25
41.15
41.13
F
O
3p
200
180
160
140
120
100
80
60
40
20
0
ppm

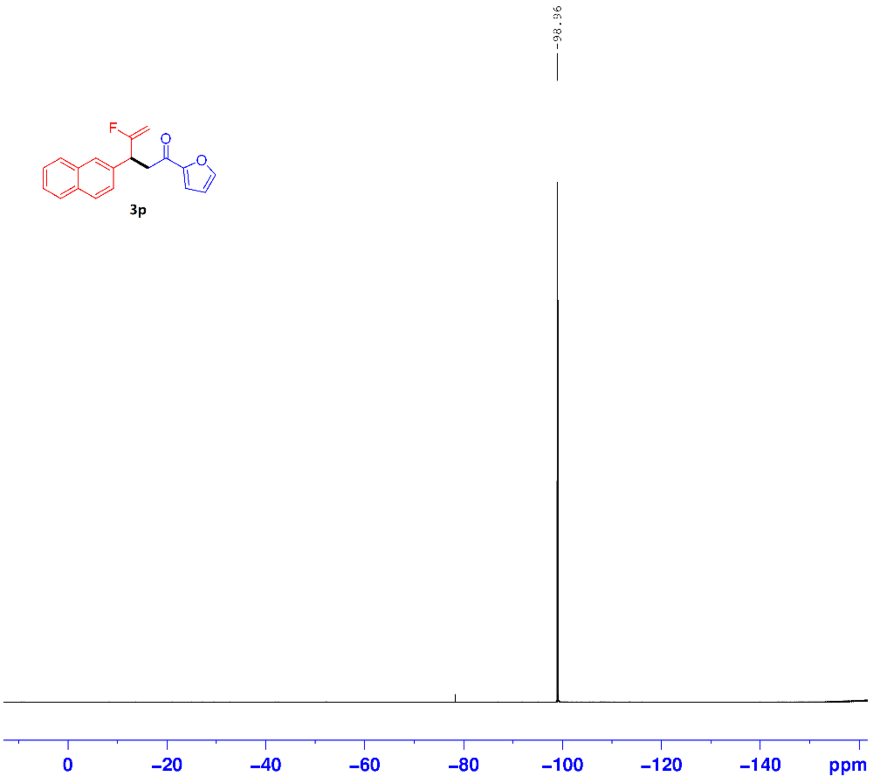
F
O
O
3p
-98.96
0
-20
-40
-60
-80
-100
-120
-140
ppm

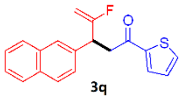
F
O
S

3q

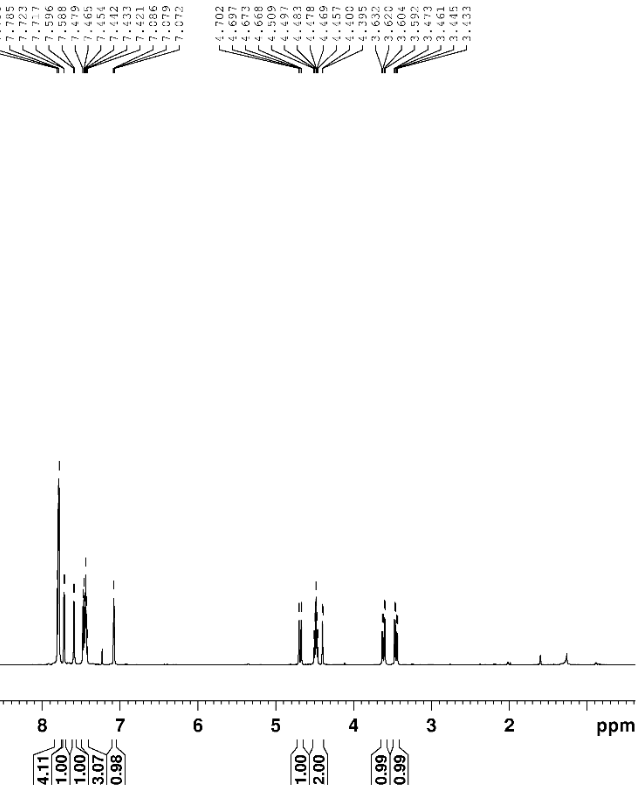
7.808
7.795
7.785
7.723
7.717
7.596
7.588
7.479
7.465
7.454
7.442
7.433
7.421
7.086
7.079
7.072
4.702
4.697
4.673
4.668
4.509
4.497
4.483
4.478
4.469
4.457
4.400
4.395
3.632
3.620
3.604
3.592
3.473
3.461
3.445
3.433
11
10
9
8
7
6
5
4
3
2
ppm
4.11
1.00
1.00
3.07
0.98
1.00
2.00
0.99
0.99

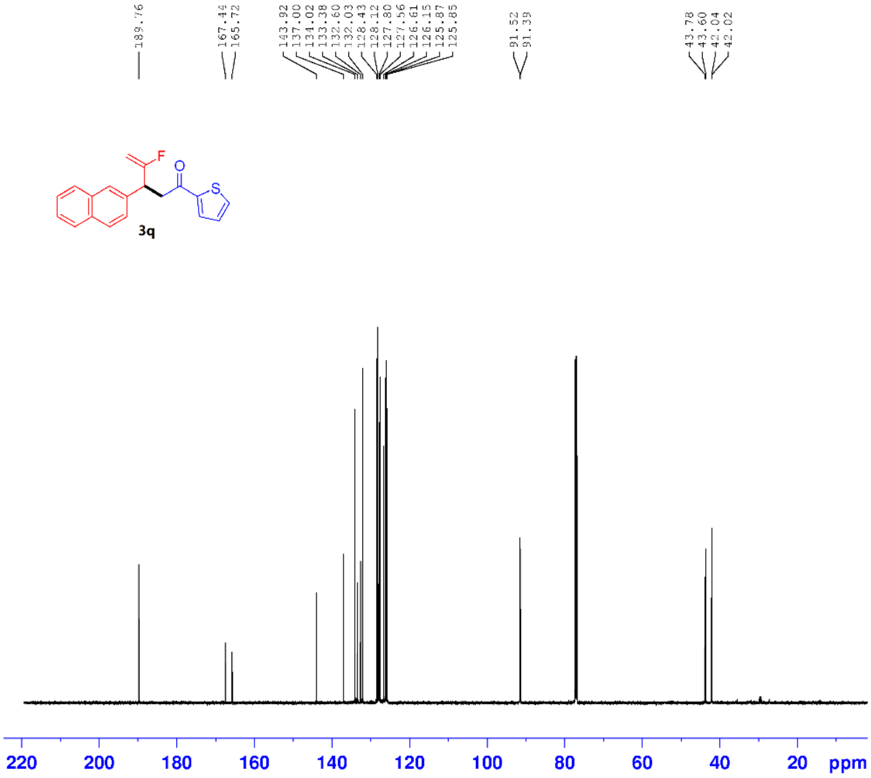
189.76
167.44
165.72
143.92
137.00
134.02
133.38
132.60
132.03
128.43
128.12
127.80
127.56
126.61
126.15
125.87
125.85
91.52
91.39
43.78
43.60
42.04
42.02
F
O
S
3q
220
200
180
160
140
120
100
80
60
40
20
ppm

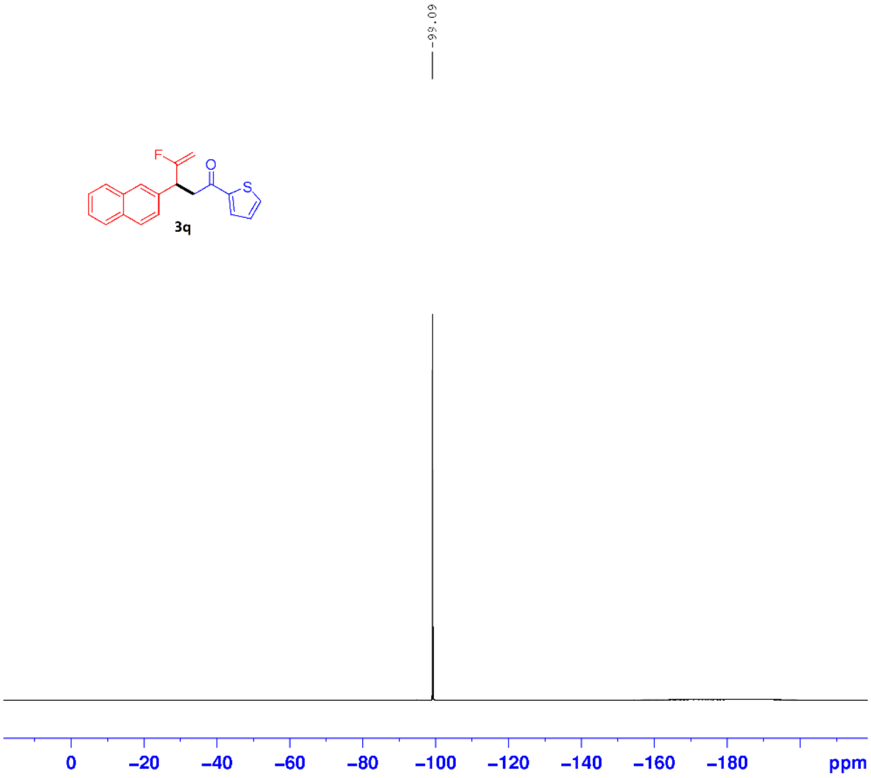
-99.09
F
O
S
3q
0
–20
–40
–60
–80
–100
–120
–140
–160
–180
ppm

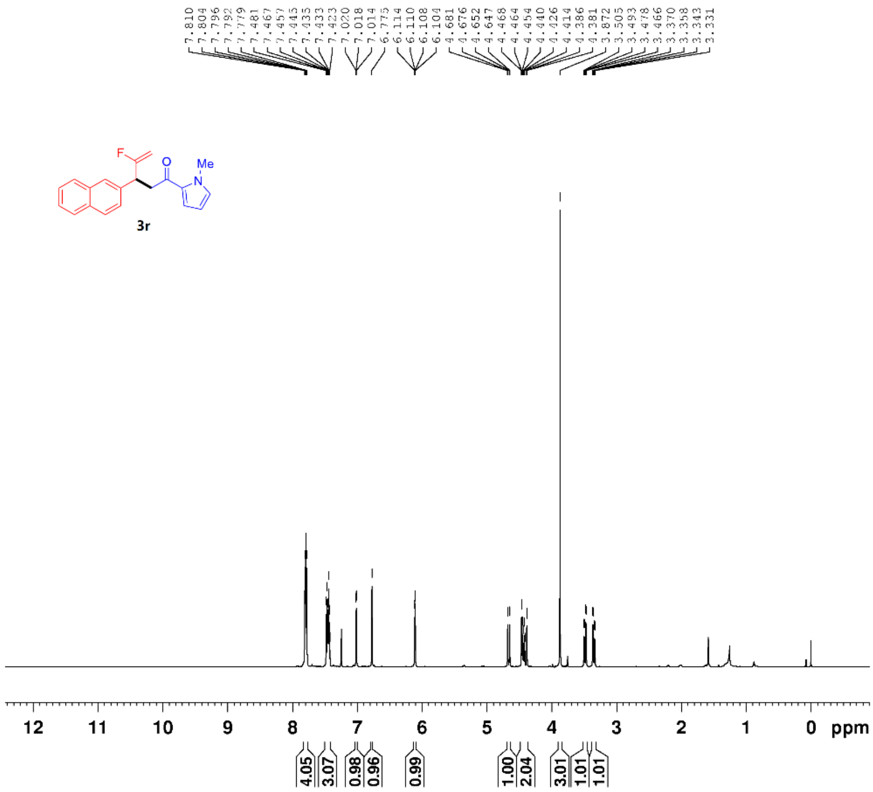

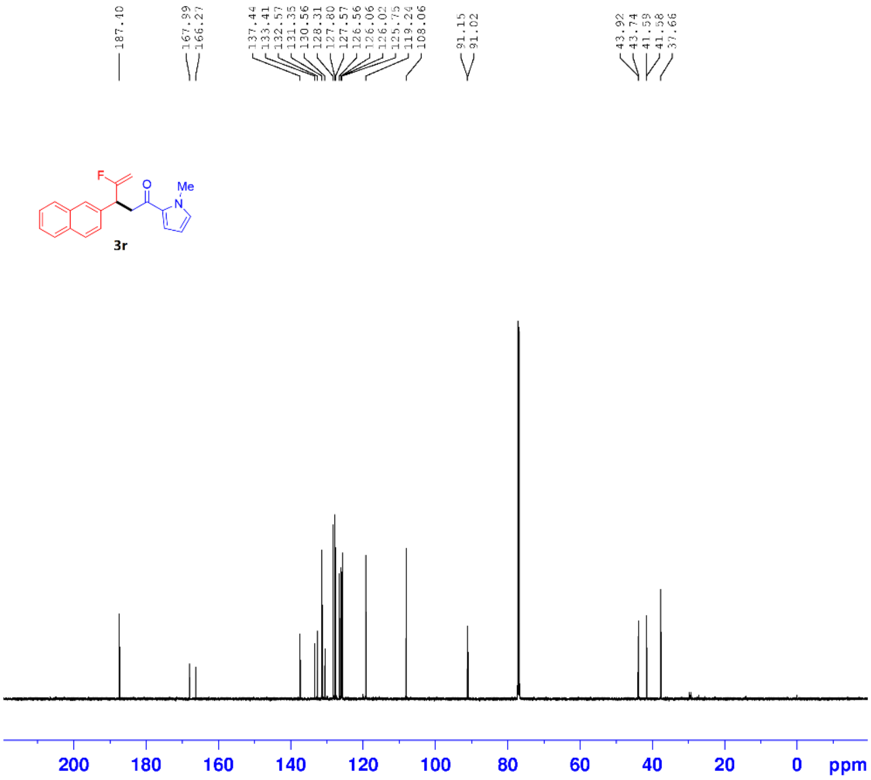
187.40
167.99
166.27
137.44
133.41
132.57
131.35
130.56
128.31
127.80
127.57
126.56
126.06
126.02
125.75
119.24
108.06
91.15
91.02
43.92
43.74
41.59
41.58
37.66
F
O
Me
N
3r
200
180
160
140
120
100
80
60
40
20
0
ppm

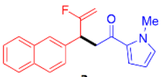

3r

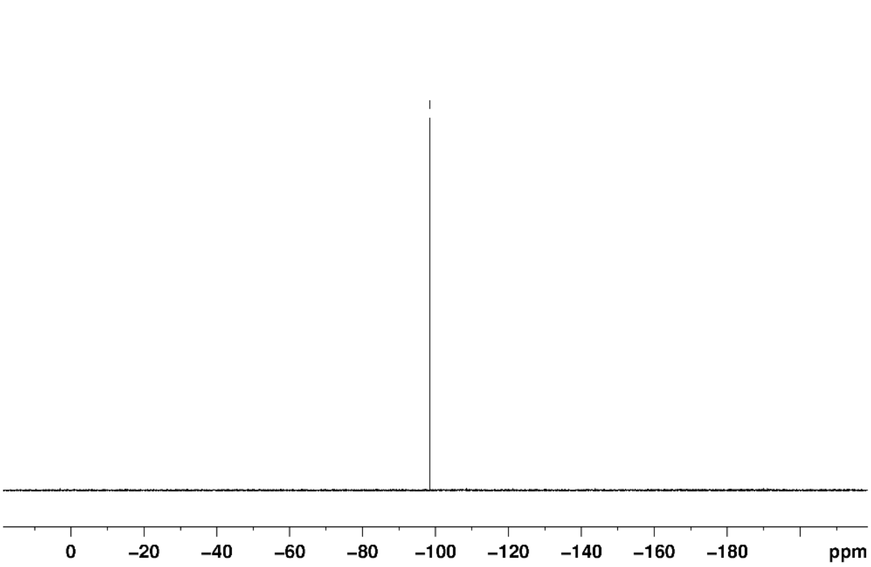

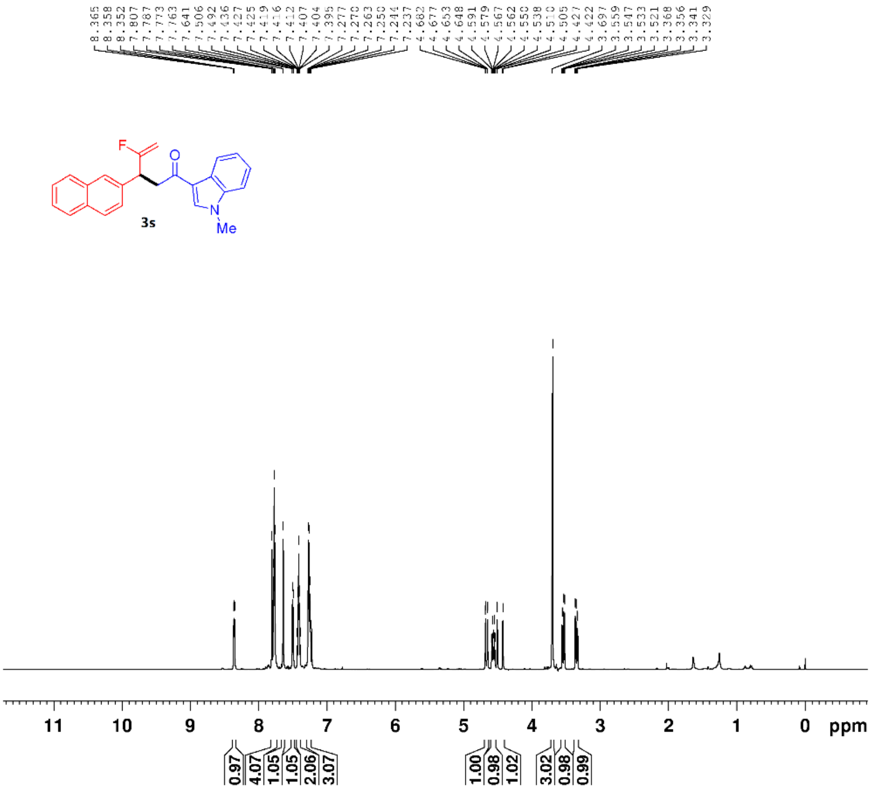

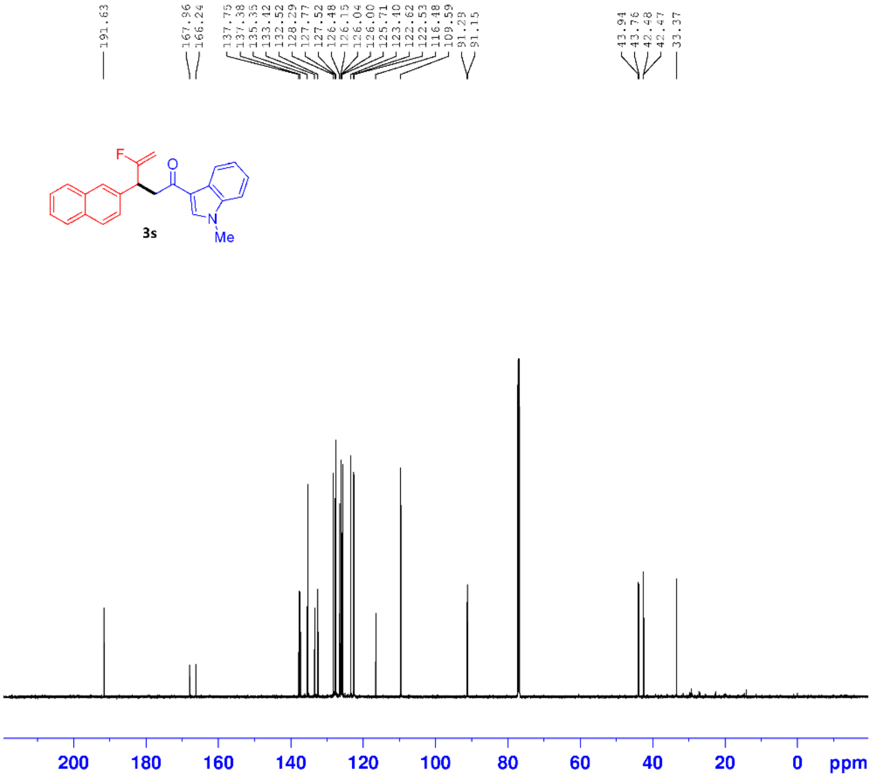
191.63
167.96
166.24
137.75
137.38
135.35
133.42
132.52
128.29
127.77
127.52
126.48
126.15
126.04
126.00
125.71
123.10
122.62
122.53
116.48
109.59
91.29
91.15
43.94
43.76
42.48
42.47
33.37
F
O
N
Me
3s
200
180
160
140
120
100
80
60
40
20
0
ppm

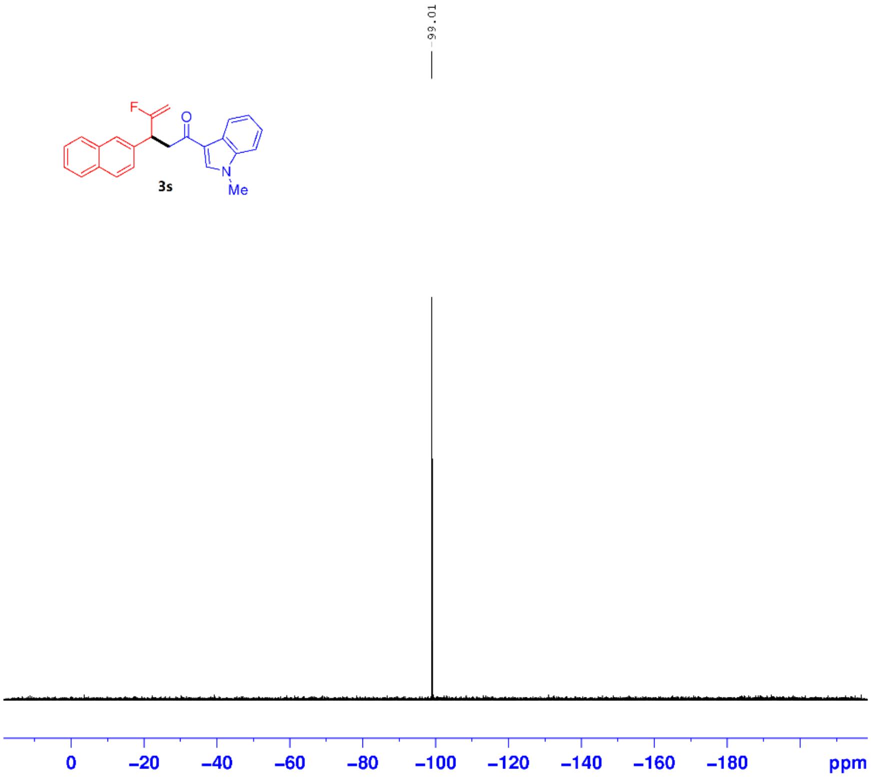

3s

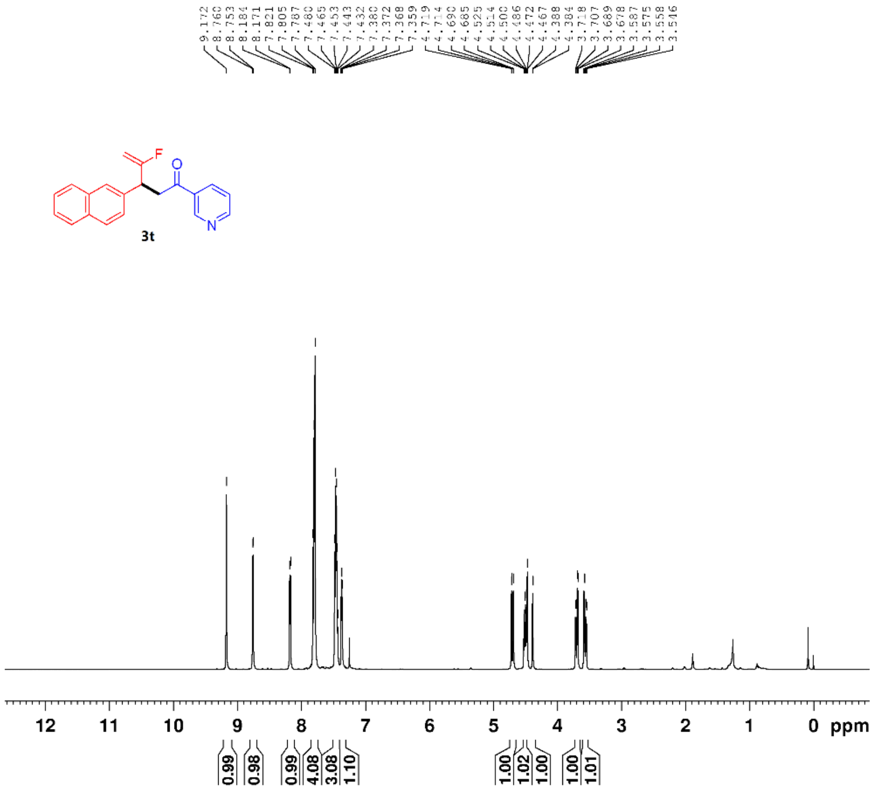
9.172
8.760
8.753
8.184
8.171
7.821
7.805
7.787
7.480
7.465
7.453
7.443
7.432
7.380
7.372
7.368
7.359
4.719
4.714
4.690
4.685
4.525
4.514
4.500
4.486
4.472
4.467
4.388
4.384
3.718
3.707
3.689
3.678
3.587
3.575
3.558
3.546
F
O
N
3t
12
11
10
9
8
7
6
5
4
3
2
1
0
ppm
0.99
0.98
0.99
4.08
3.08
1.10
1.00
1.02
1.00
1.00
1.01

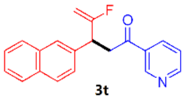

3t

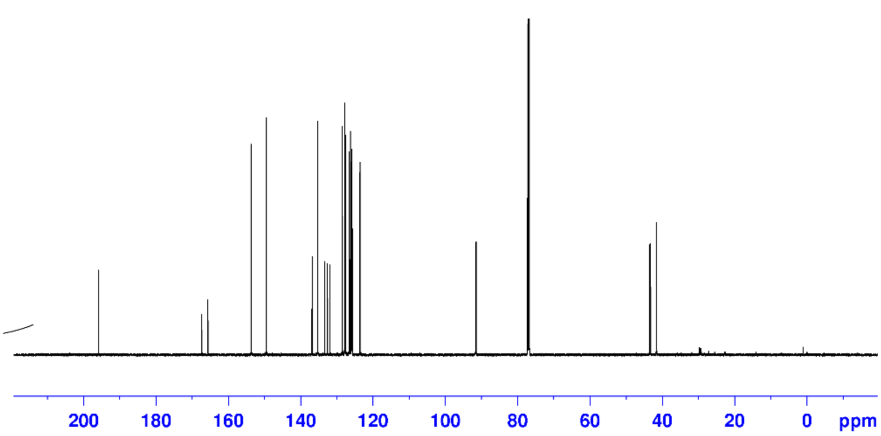
195.90
167.39
165.67
153.70
149.49
136.85
135.32
133.35
132.61
131.89
128.52
127.76
127.58
126.61
126.24
125.96
125.79
123.62
91.62
91.49
43.45
43.27
41.61
41.59
200
180
160
140
120
100
80
60
40
20
0
ppm

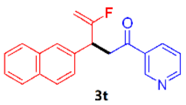

3t

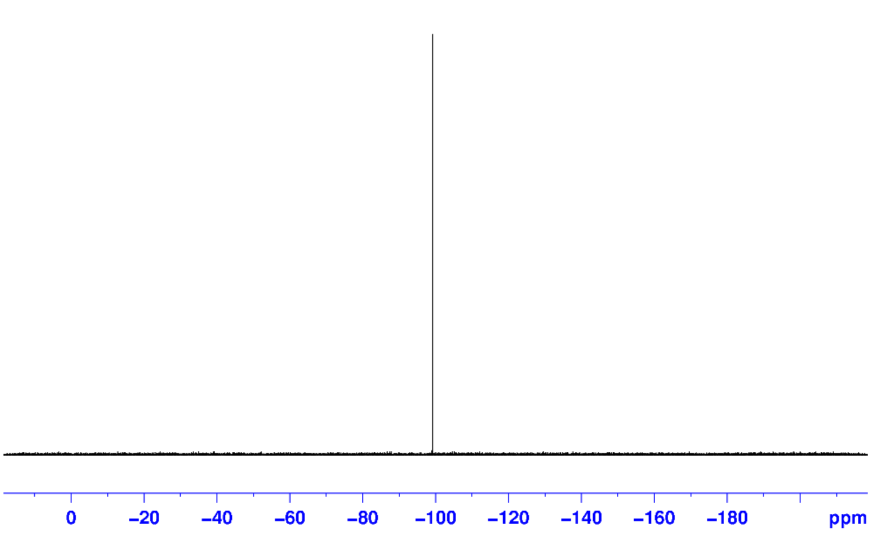

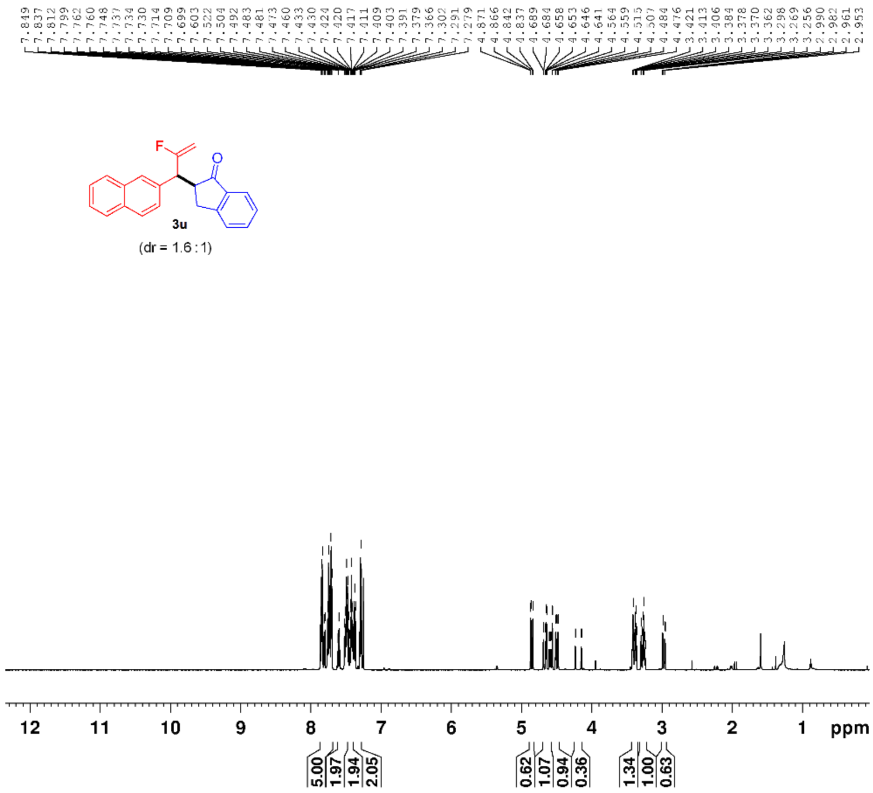

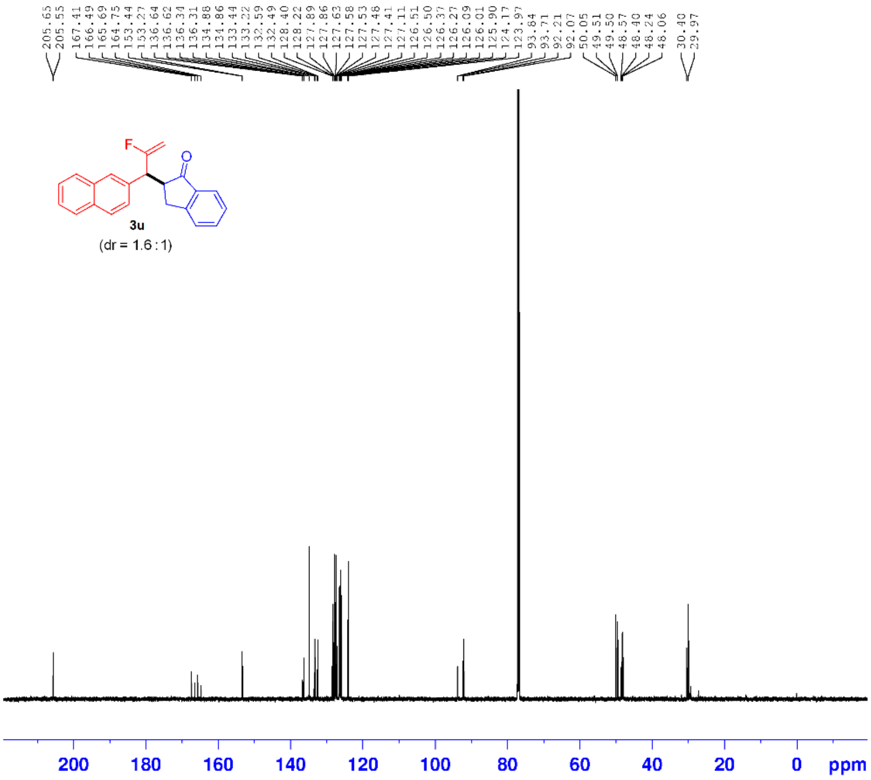

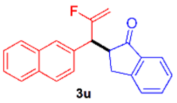

3u
(dr = 1.6 : 1)

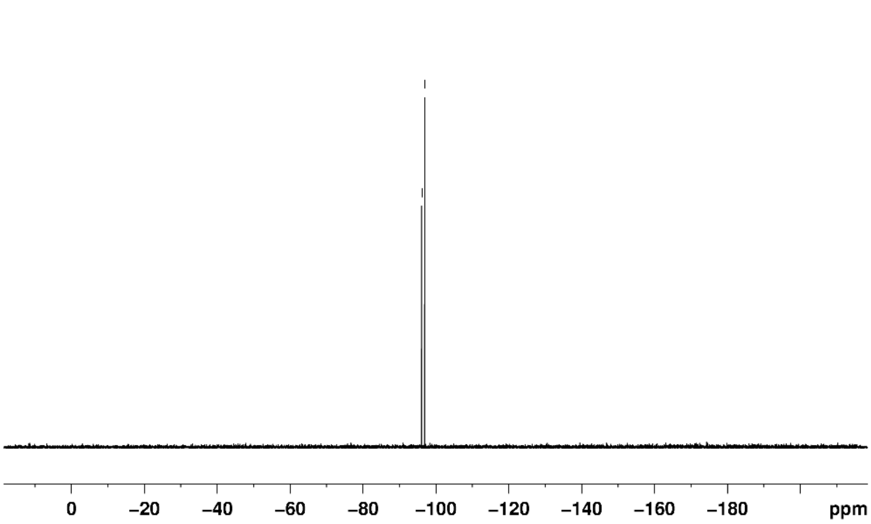

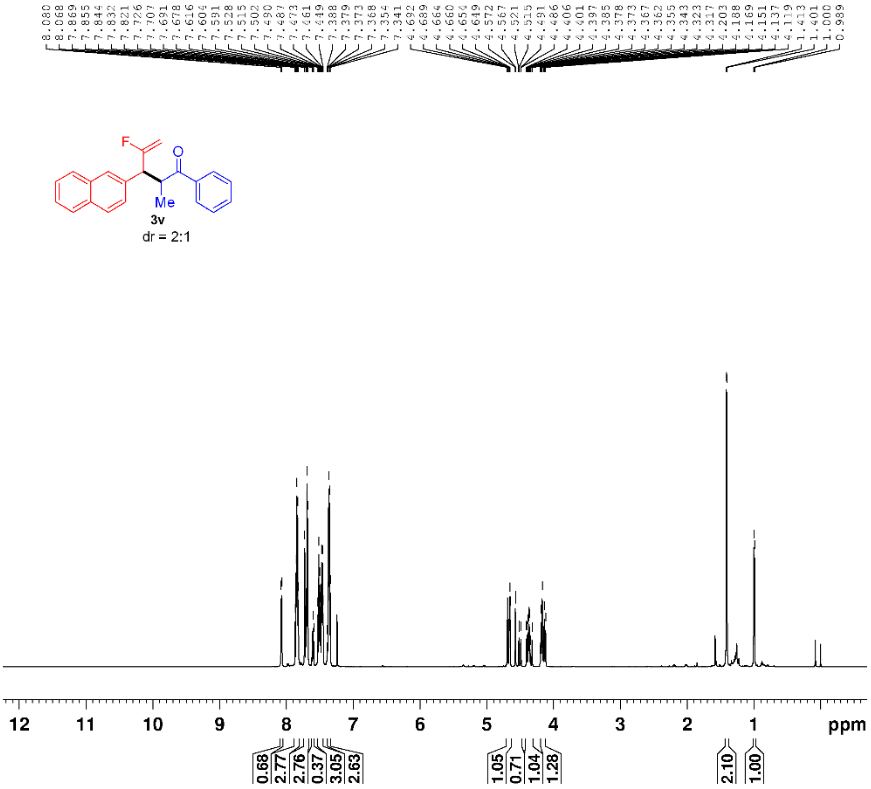

3v
dr = 2:1
ppm

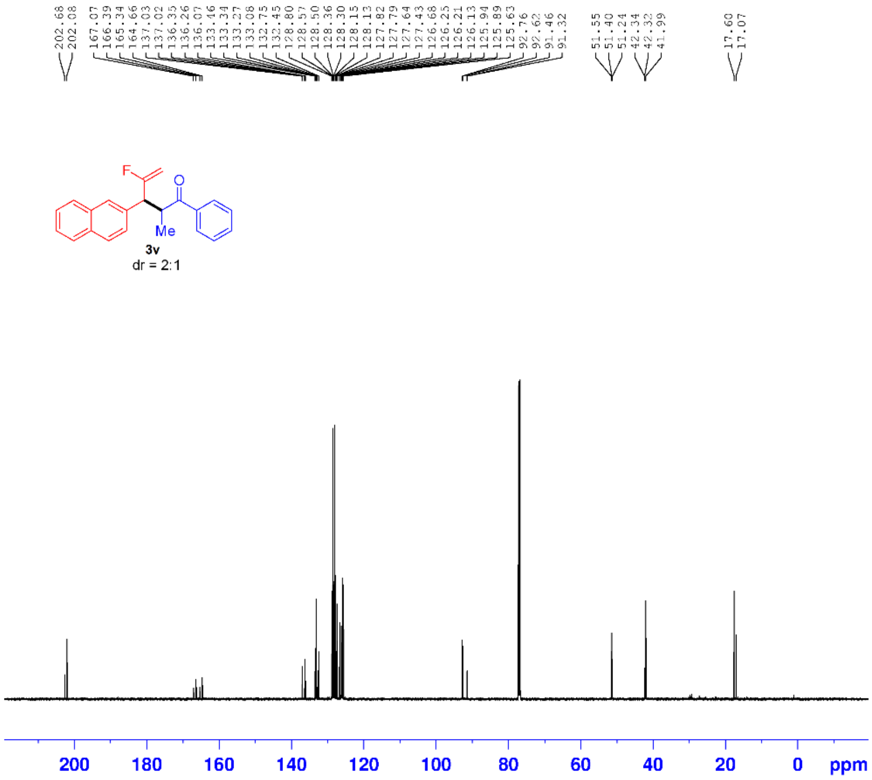
202.68
202.08
167.07
166.39
165.34
164.66
137.03
137.02
136.35
136.26
136.07
133.46
133.34
133.27
133.08
132.75
132.45
128.80
128.57
128.50
128.36
128.30
128.15
128.13
127.82
127.79
127.64
127.43
126.68
126.25
126.21
126.13
125.94
125.89
125.63
92.76
92.62
91.46
91.32
51.55
51.40
51.24
42.34
42.32
41.99
17.60
17.07
F
O
Me
3v
dr = 2:1
200
180
160
140
120
100
80
60
40
20
0
ppm

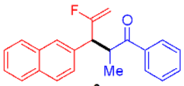

3v
dr = 2:1

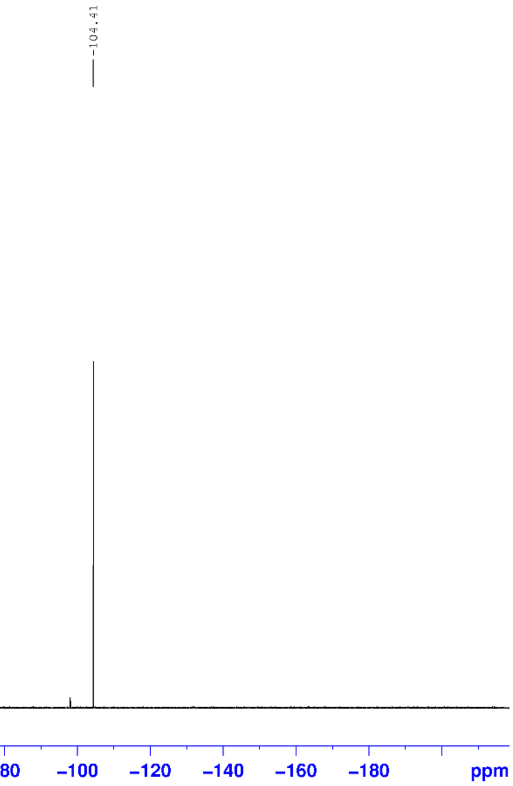

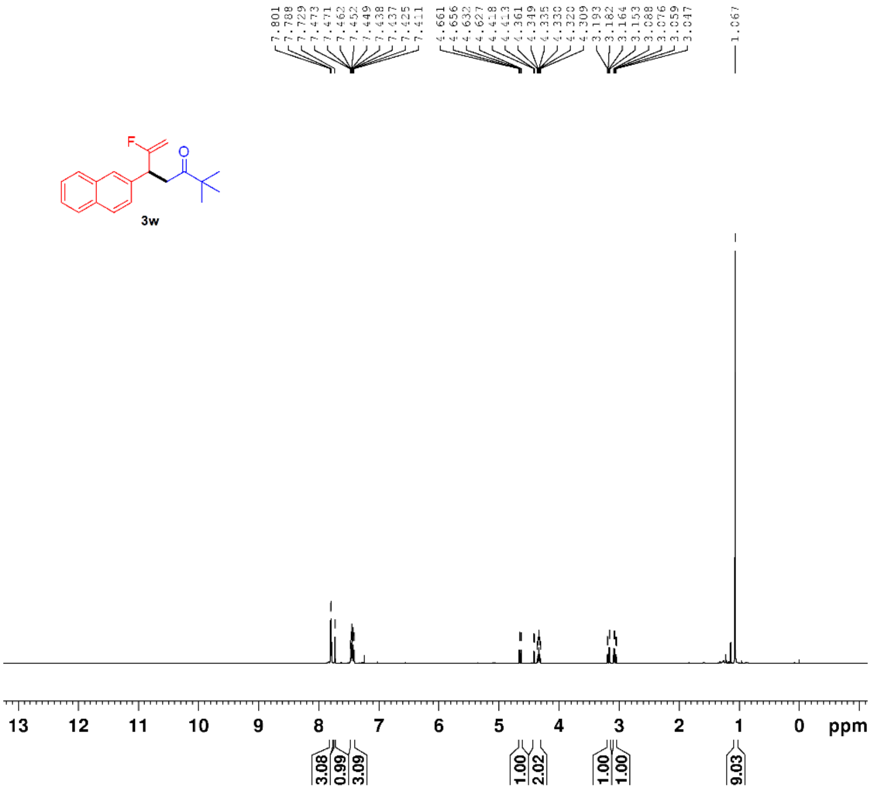

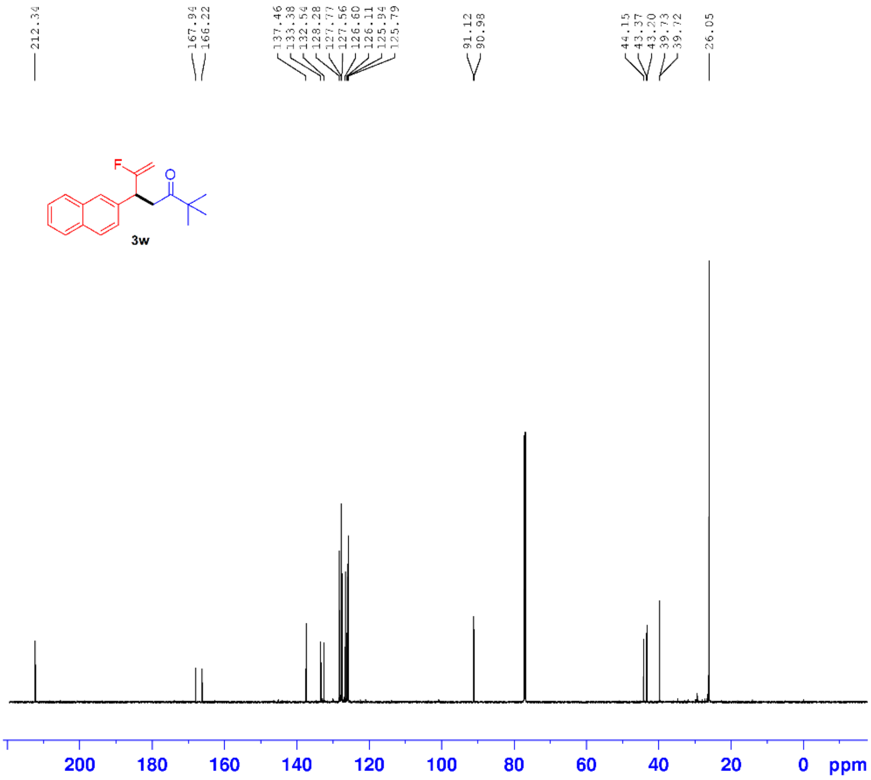

212.34
167.94
166.22
137.46
133.38
132.54
128.28
127.77
127.56
126.60
126.11
125.94
125.79
91.12
90.98
44.15
43.37
43.20
39.73
39.72
26.05
F
O
3w
200
180
160
140
120
100
80
60
40
20
0
ppm

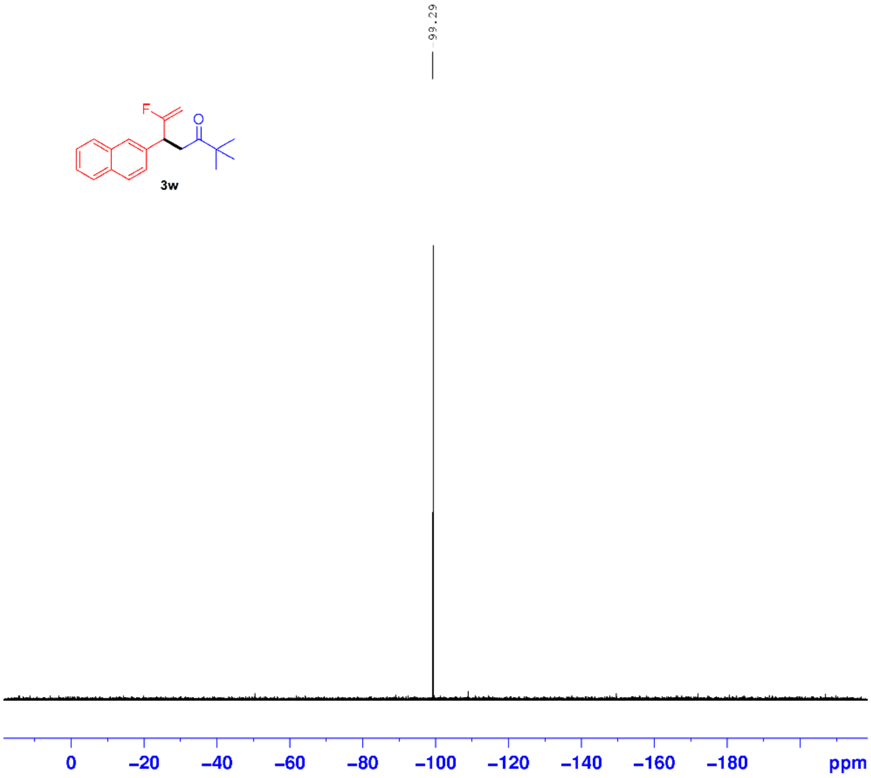
-99.29
F
O
3w
0
-20
-40
-60
-80
-100
-120
-140
-160
-180
ppm

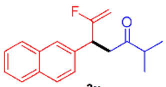

3x

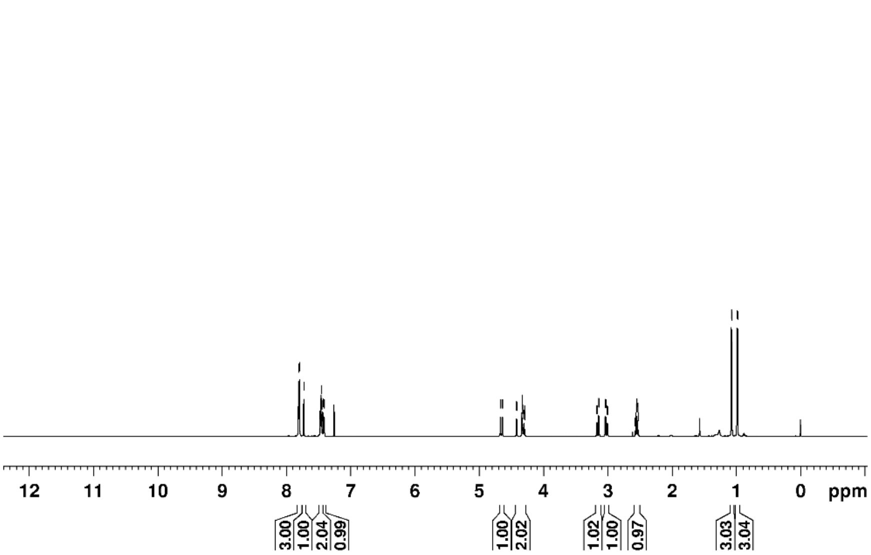
12
11
10
9
8
7
6
5
4
3
2
1
0
ppm
3.00
1.00
2.04
0.99
1.00
2.02
1.02
1.00
0.97
3.03
3.04

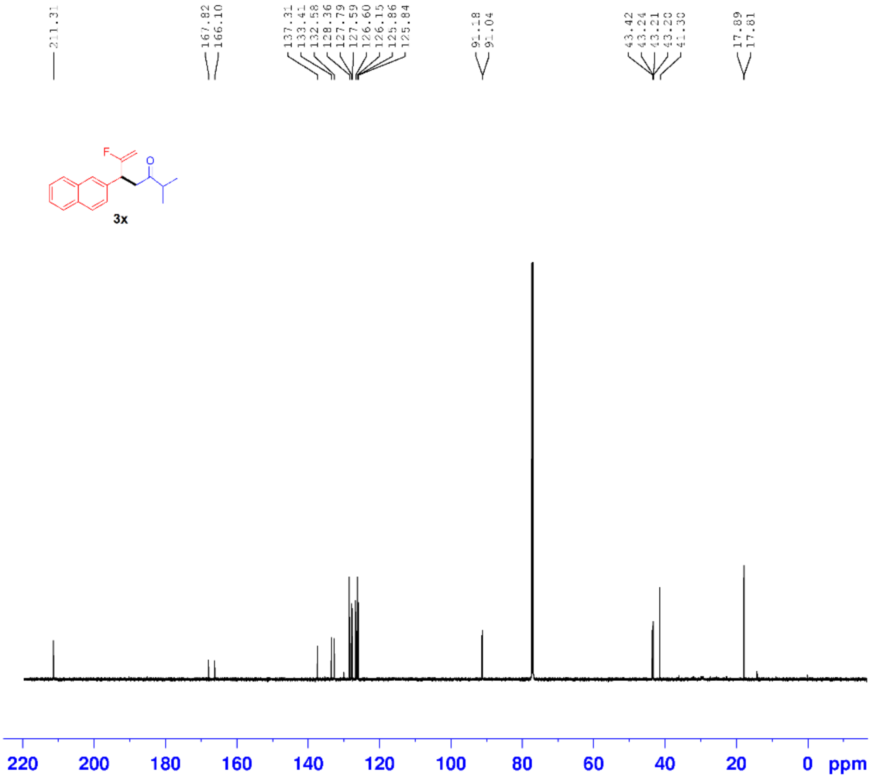
211.31
167.82
166.10
137.31
133.41
132.58
128.36
127.79
127.59
126.60
126.15
125.86
125.84
91.18
91.04
43.42
43.24
43.21
43.20
41.30
17.89
17.81
F
O
3x
220
200
180
160
140
120
100
80
60
40
20
0
ppm

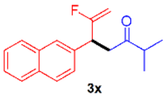

3x

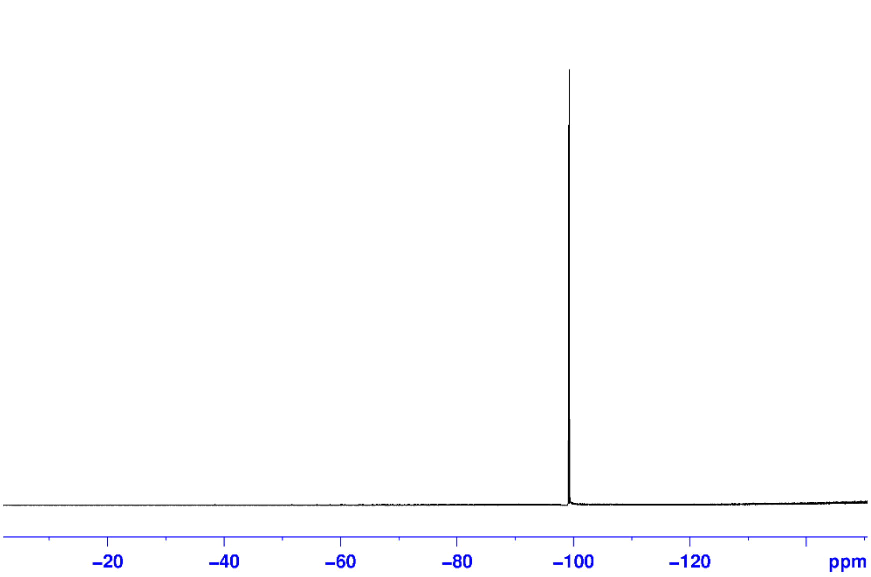

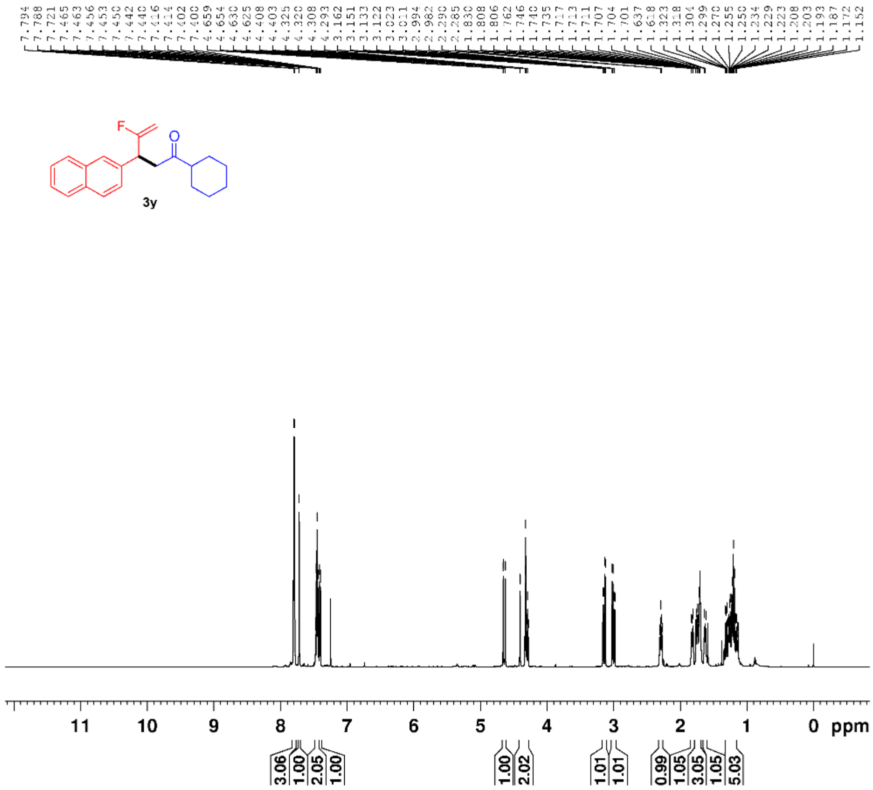

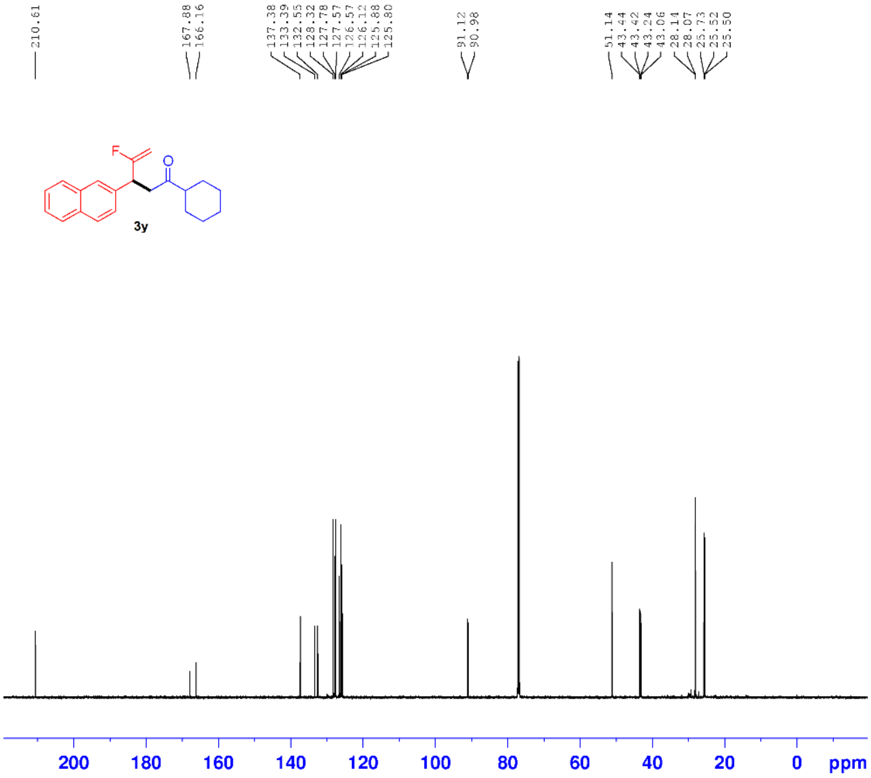
210.61
167.88
166.16
137.38
133.39
132.55
128.32
127.78
127.57
126.57
126.12
125.88
125.80
91.12
90.98
51.14
43.44
43.42
43.24
43.06
28.14
28.07
25.73
25.52
25.50
F
O
3y
200
180
160
140
120
100
80
60
40
20
0
ppm

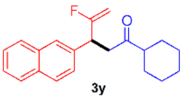

3y

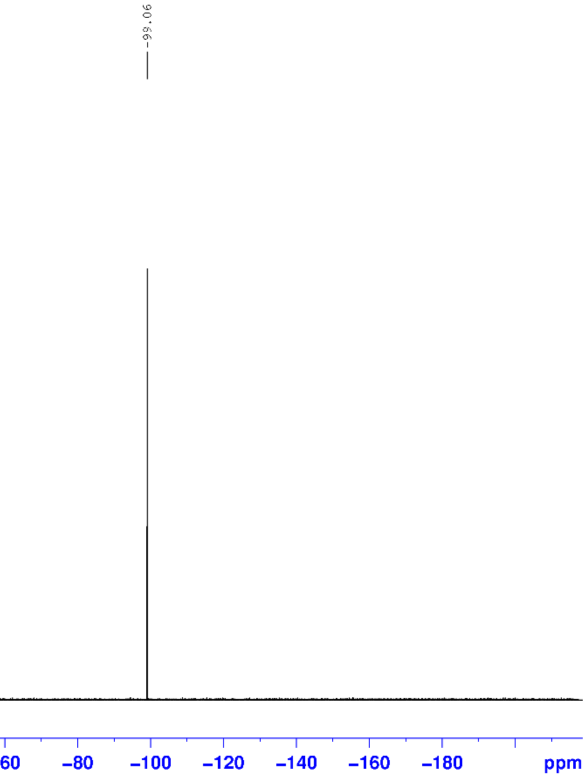

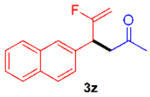

3z

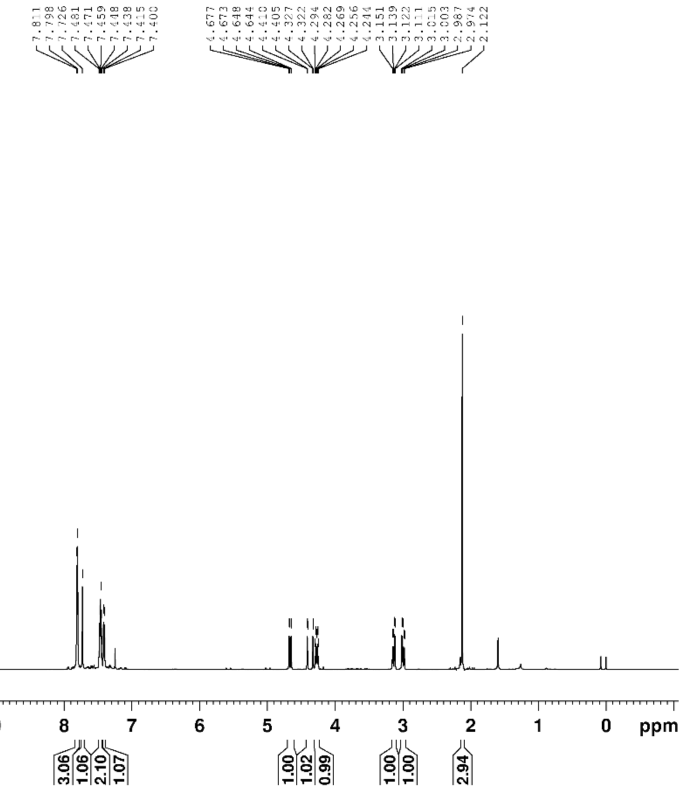

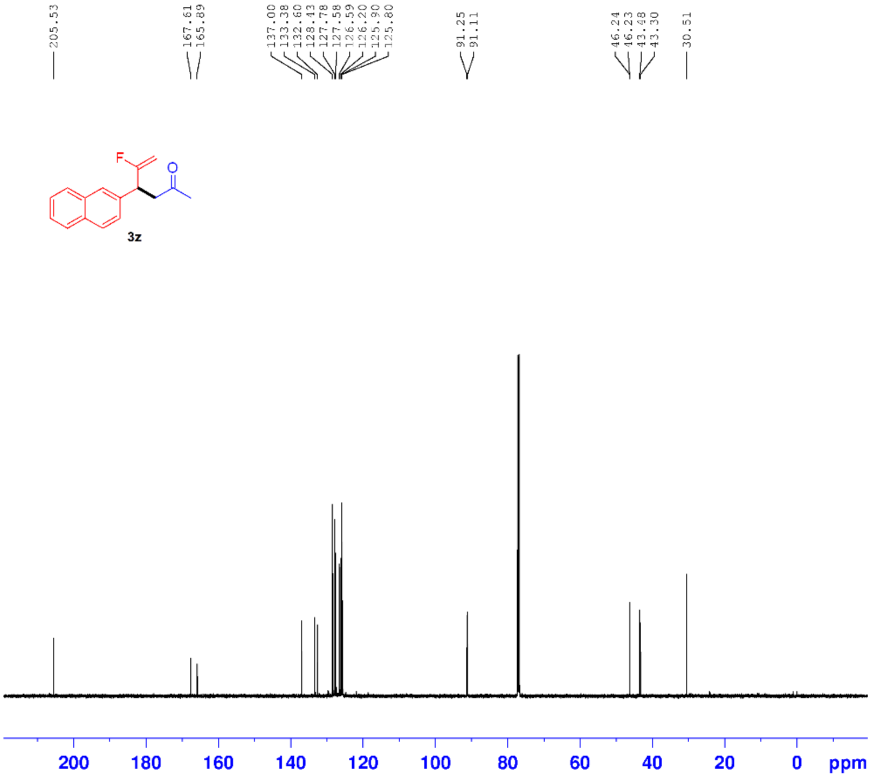
205.53
167.61
165.89
137.00
133.38
132.60
128.43
127.78
127.58
126.59
126.20
125.90
125.80
91.25
91.11
46.24
46.23
43.48
43.30
30.51
F
O
3z
200
180
160
140
120
100
80
60
40
20
0
ppm

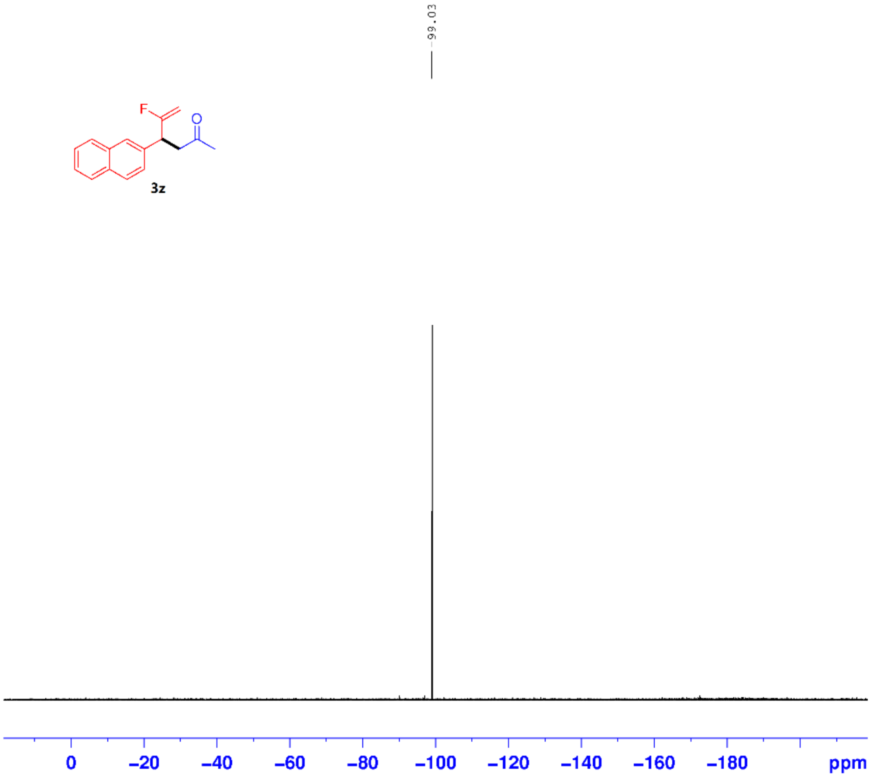

3z

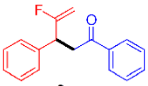

3aa

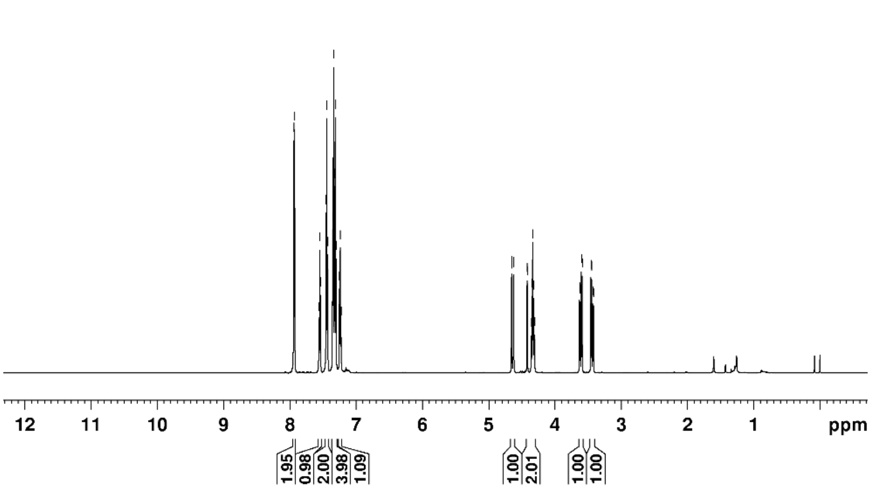

7.941
7.928
7.561
7.549
7.537
7.454
7.441
7.428
7.353
7.340
7.326
7.314
7.301
7.252
7.240
7.228
4.655
4.650
4.626
4.620
4.420
4.415
4.357
4.345
4.336
4.332
4.317
4.305
3.629
3.618
3.600
3.589
3.458
3.446
3.429
3.417
12
11
10
9
8
7
6
5
4
3
2
1
ppm
1.95
0.98
2.00
3.98
1.09
1.00
2.01
1.00
1.00

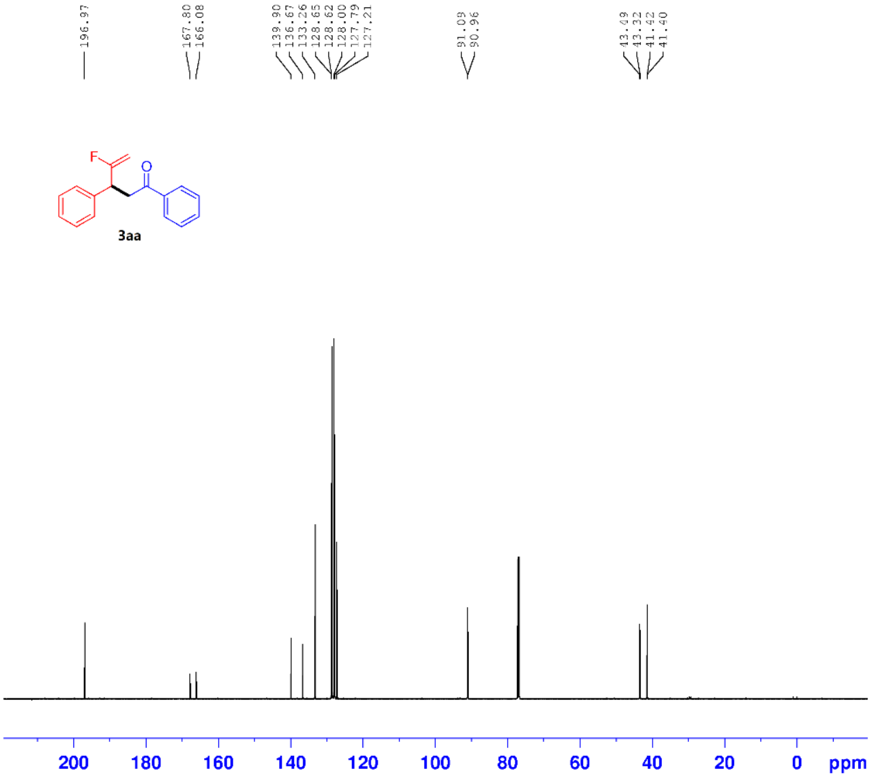

196.97
167.80
166.08
139.90
136.67
133.26
128.65
128.62
128.00
127.79
127.21
91.09
90.96
43.49
43.32
41.42
41.40
F
O
3aa
200
180
160
140
120
100
80
60
40
20
0
ppm

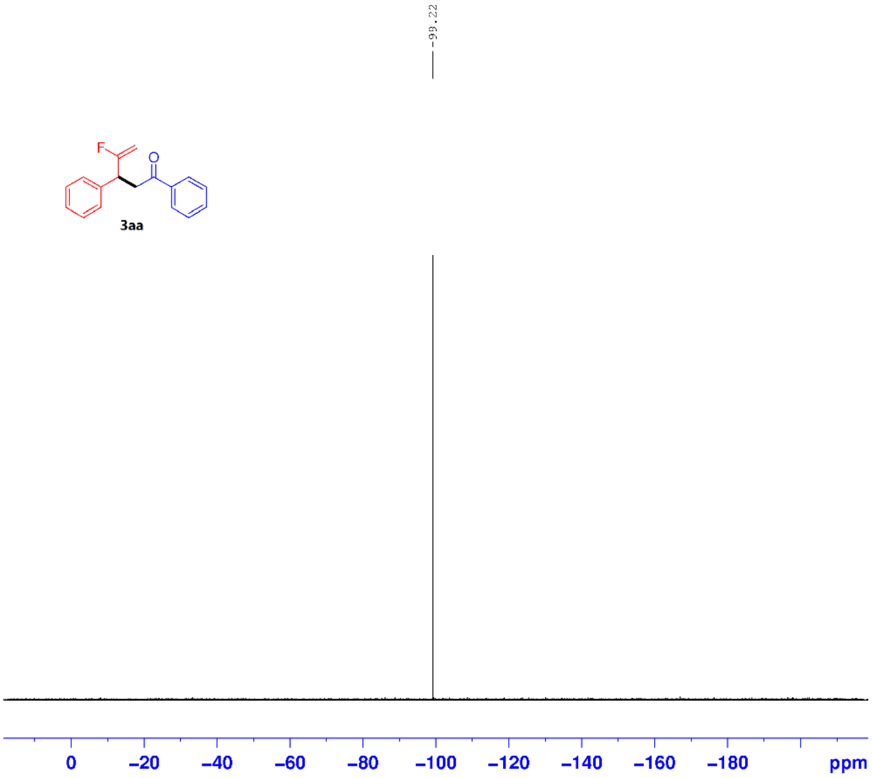
-99.22
F
O
3aa
0
–20
–40
–60
–80
–100
–120
–140
–160
–180
ppm

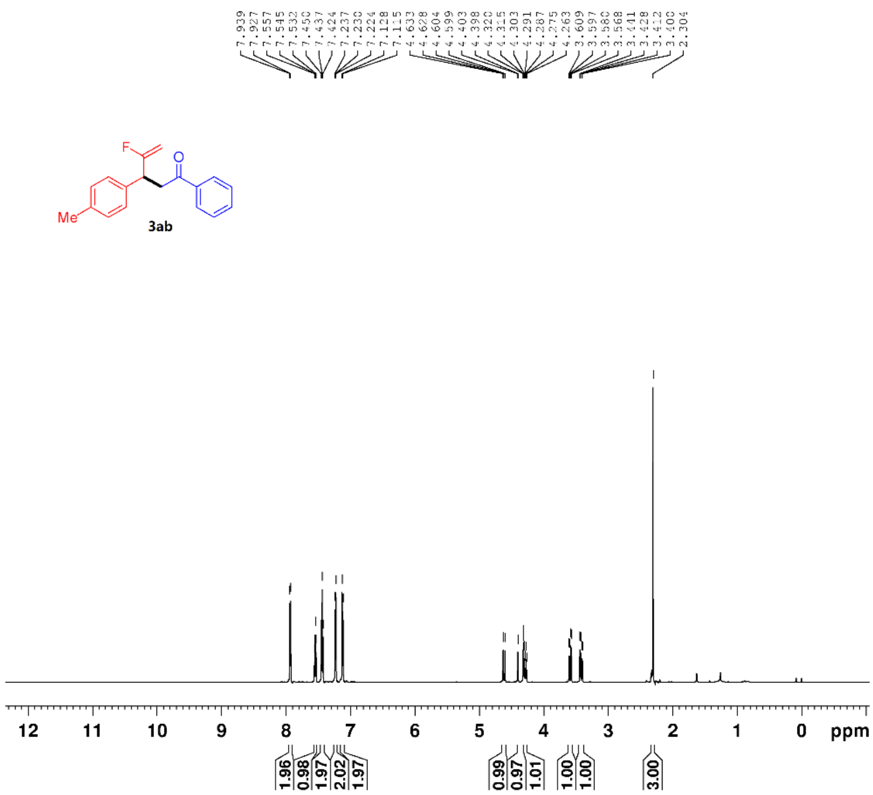

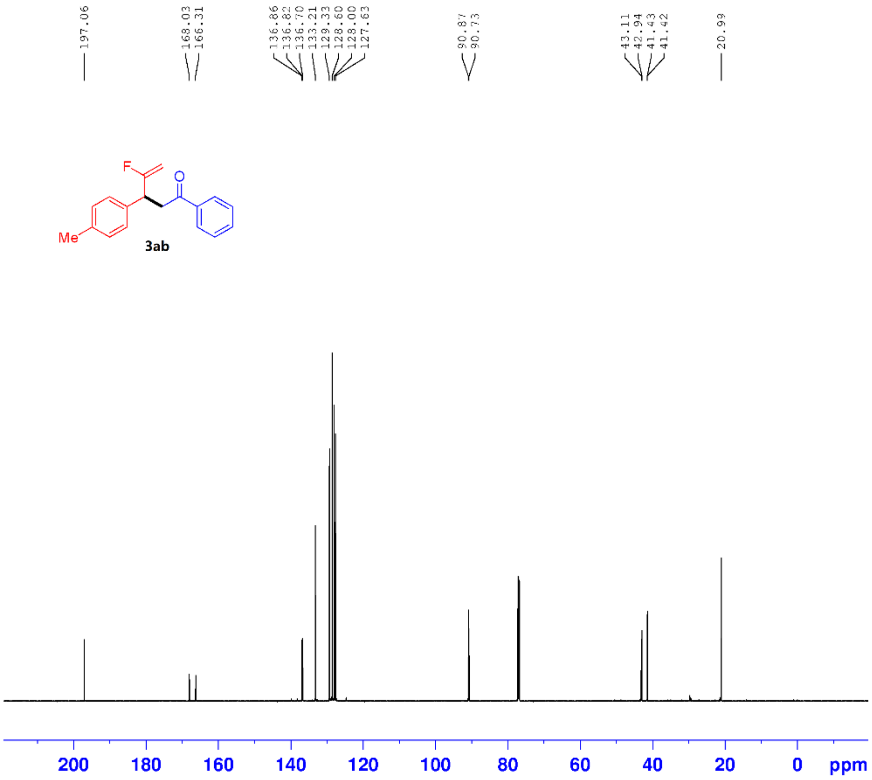
197.06
168.03
166.31
136.86
136.82
136.70
133.21
129.33
128.60
128.00
127.63
90.87
90.73
43.11
42.94
41.43
41.42
20.99
F
O
Me
3ab
200
180
160
140
120
100
80
60
40
20
0
ppm

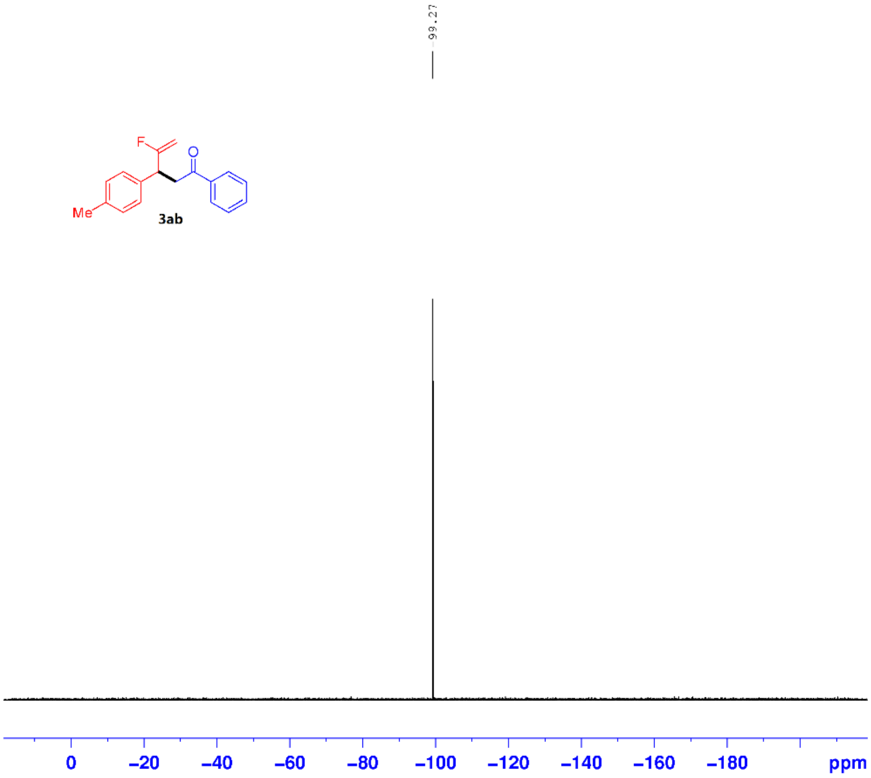

99.27
F
O
Me
3ab
0
–20
–40
–60
–80
–100
–120
–140
–160
–180
ppm

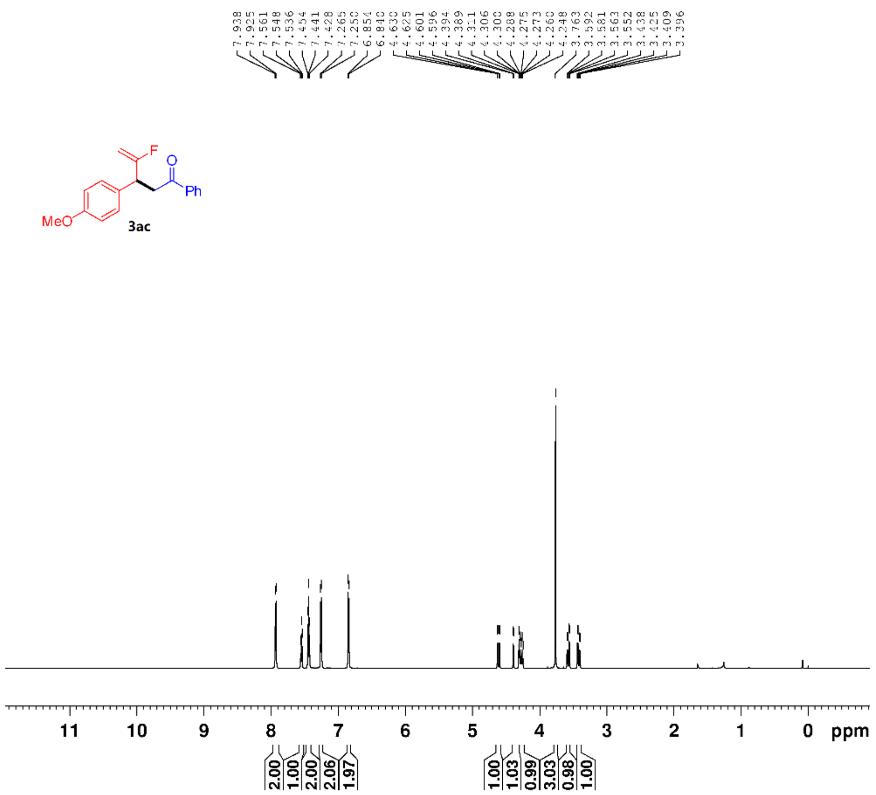

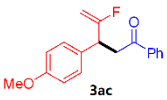
F
O
Ph
MeO

3ac

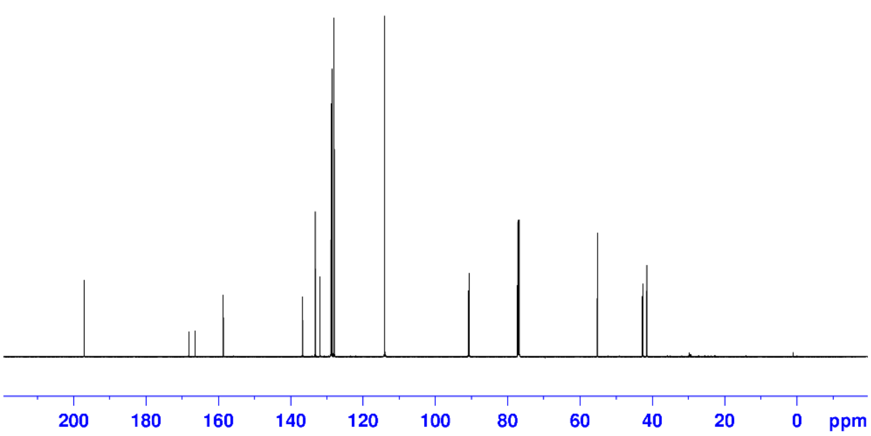
197.10
168.16
166.44
158.65
136.71
133.22
131.87
128.81
128.60
127.99
114.01
90.77
90.63
55.17
42.72
42.54
41.52
41.50
200
180
160
140
120
100
80
60
40
20
0
ppm

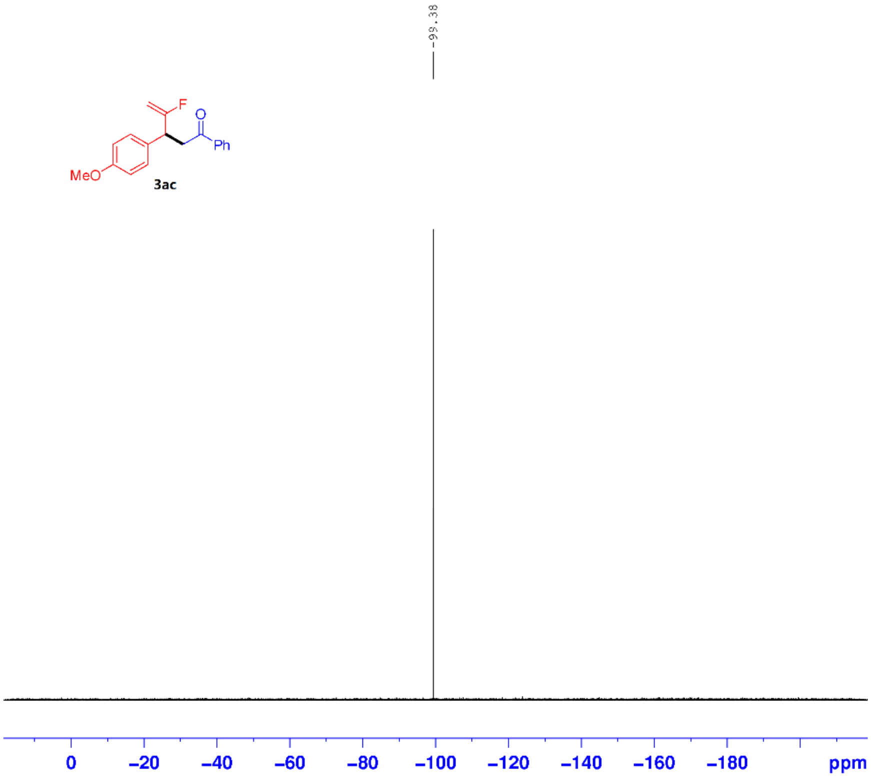

-99.38
F
O
Ph
MeO
3ac
0
-20
-40
-60
-80
-100
-120
-140
-160
-180
ppm

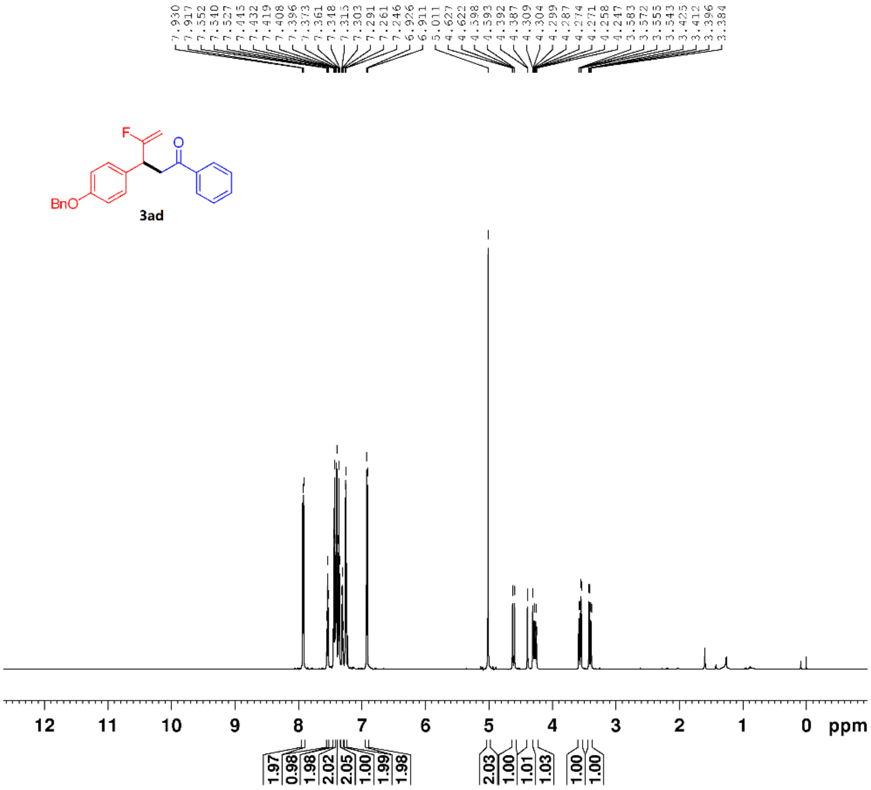

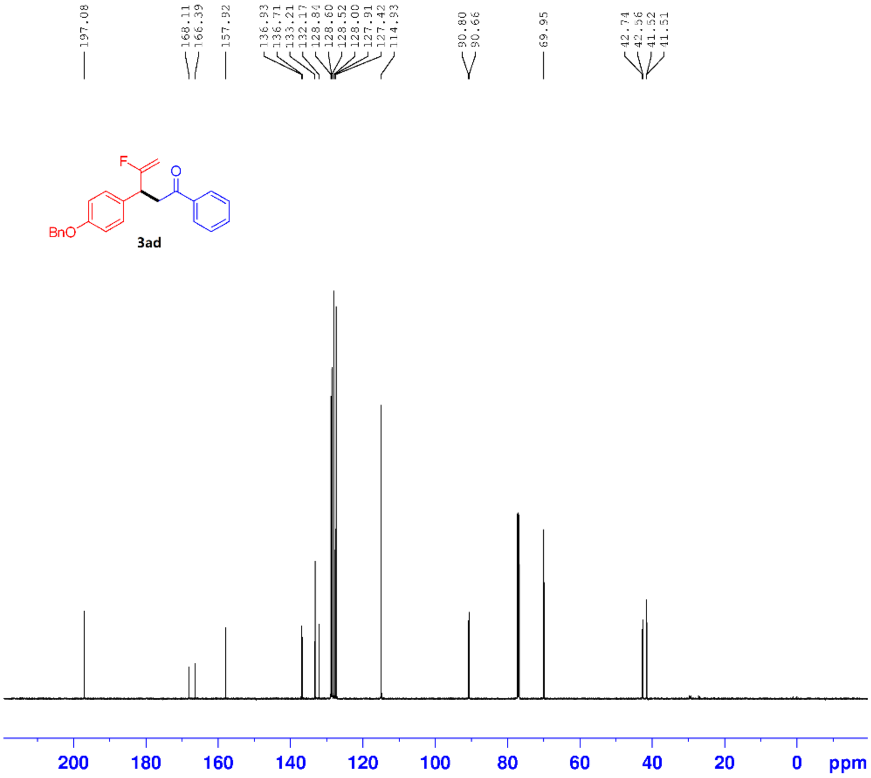

197.08
168.11
166.39
157.92
136.93
136.71
133.21
132.17
128.84
128.60
128.52
128.00
127.91
127.42
114.93
90.80
90.66
69.95
42.74
42.56
41.52
41.51
F
O
BnO
3ad
200
180
160
140
120
100
80
60
40
20
0
ppm

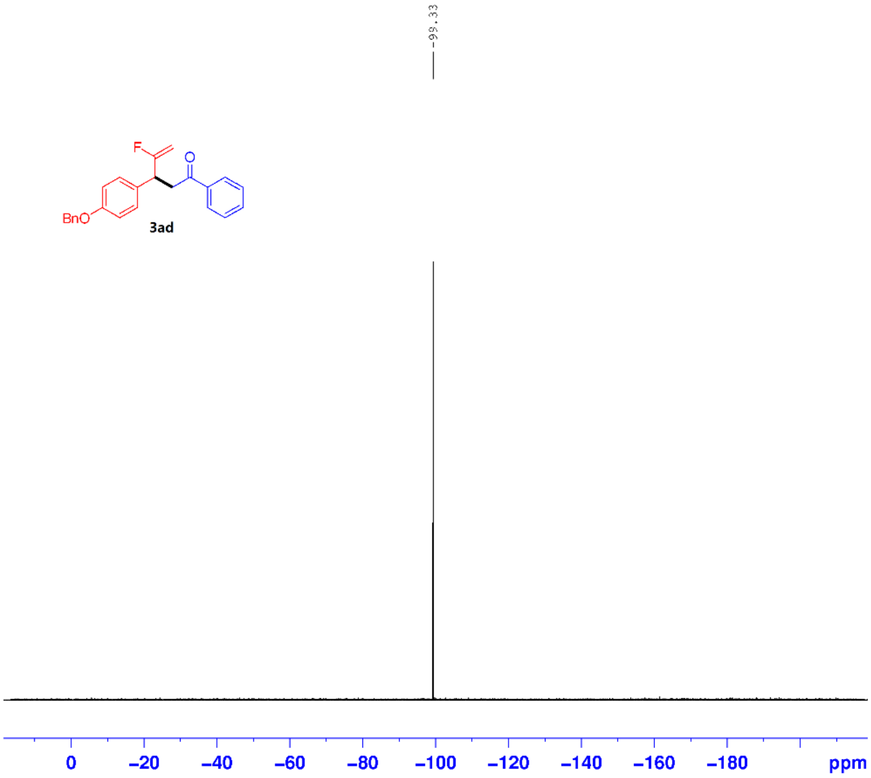
-99.33
F
O
BnO
3ad
0
-20
-40
-60
-80
-100
-120
-140
-160
-180
ppm

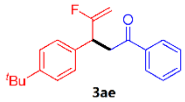

3ae

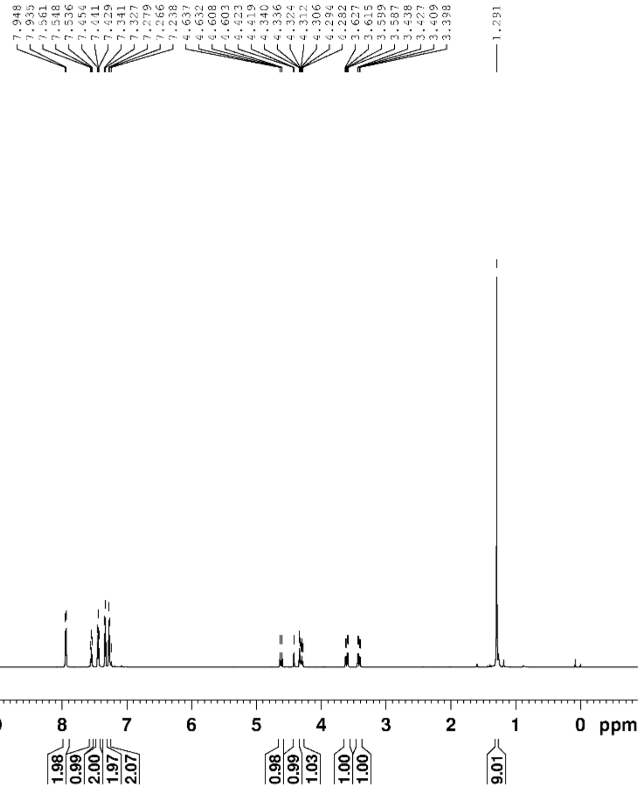

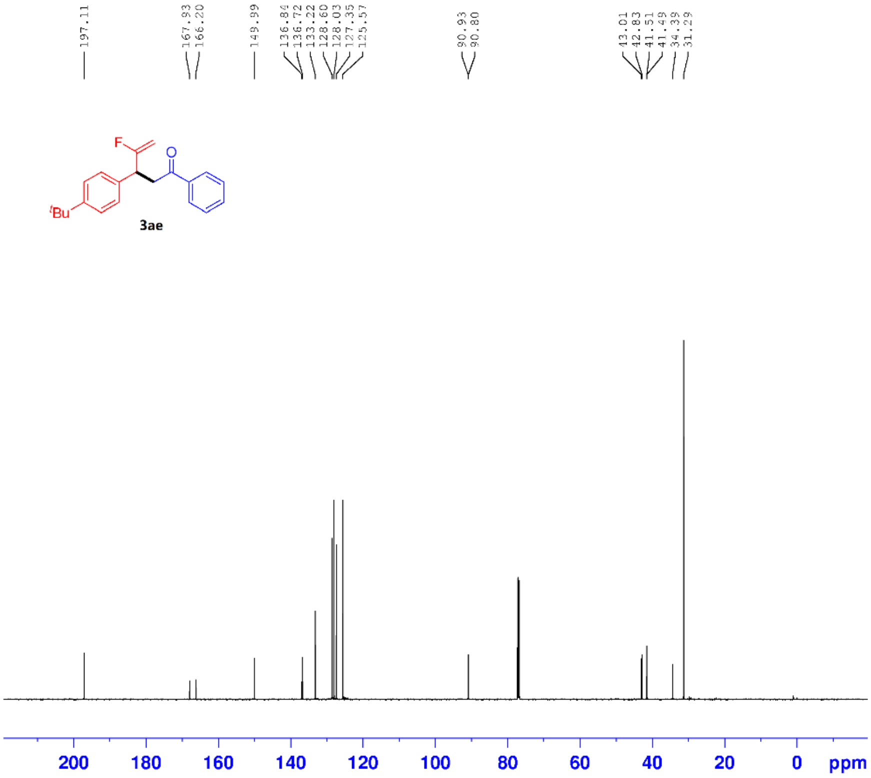
197.11
167.93
166.20
149.99
136.84
136.72
133.22
128.60
128.03
127.35
125.57
90.93
90.80
43.01
42.83
41.51
41.49
34.39
31.29
F
O
tBu
3ae
200
180
160
140
120
100
80
60
40
20
0
ppm

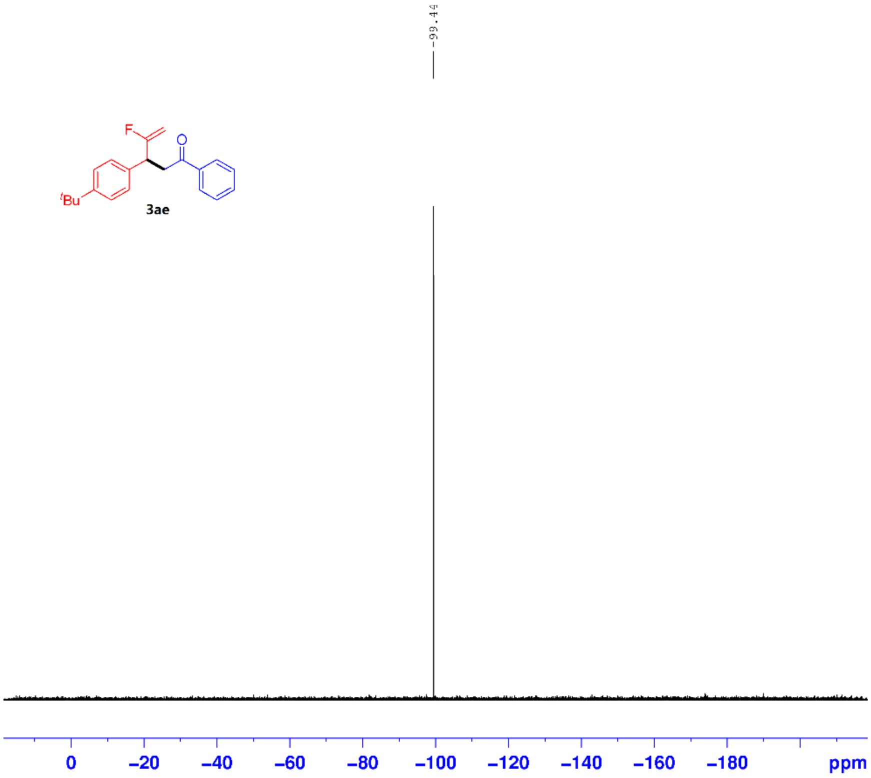
-99.44
F
O
^{t}Bu
3ae
0
-20
-40
-60
-80
-100
-120
-140
-160
-180
ppm

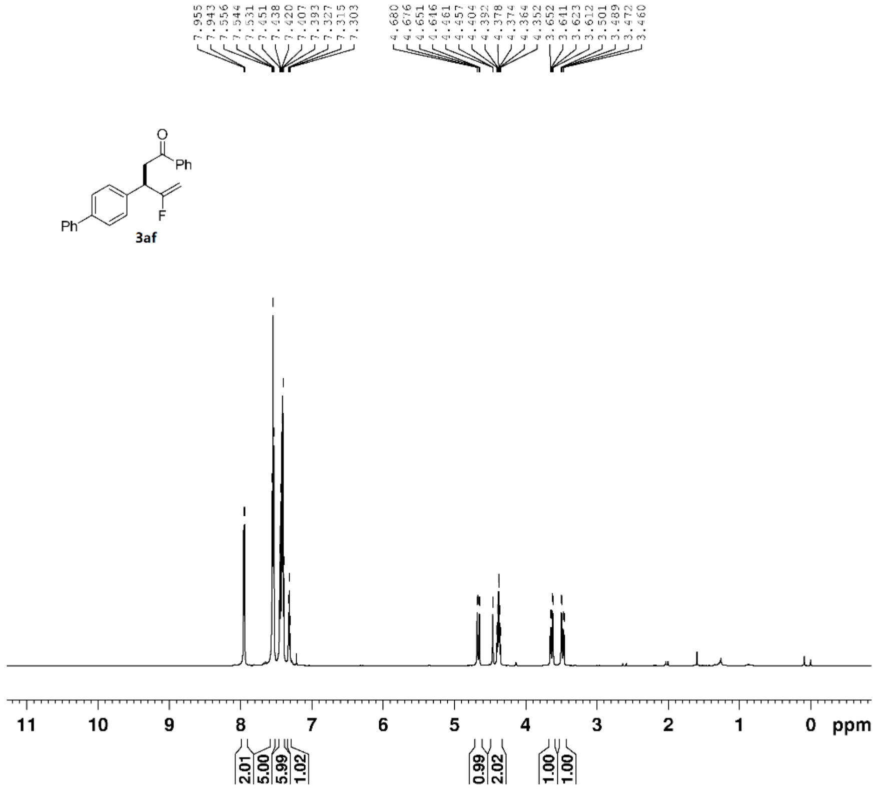

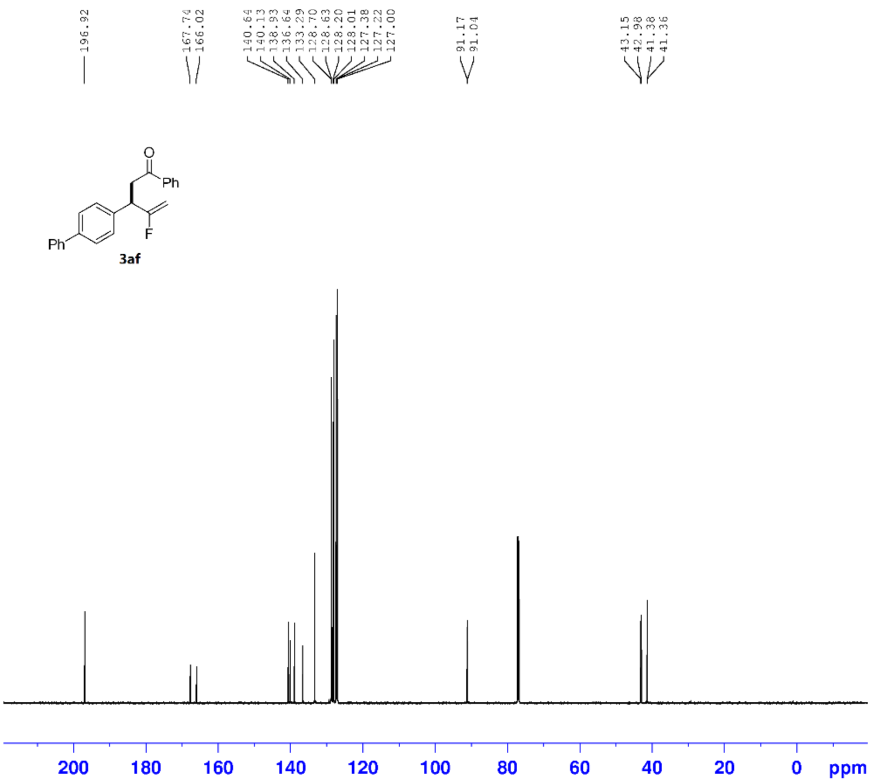
196.92
167.74
166.02
140.64
140.13
138.93
136.64
133.29
128.70
128.63
128.20
128.01
127.38
127.22
127.00
91.17
91.04
43.15
42.98
41.38
41.36
O
Ph
F
Ph
3af
200
180
160
140
120
100
80
60
40
20
0
ppm

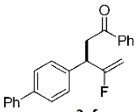

3af

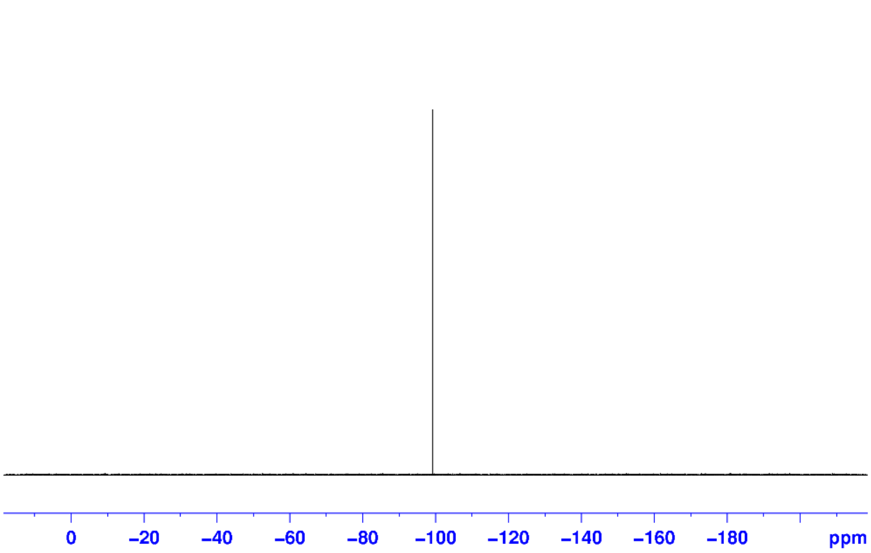

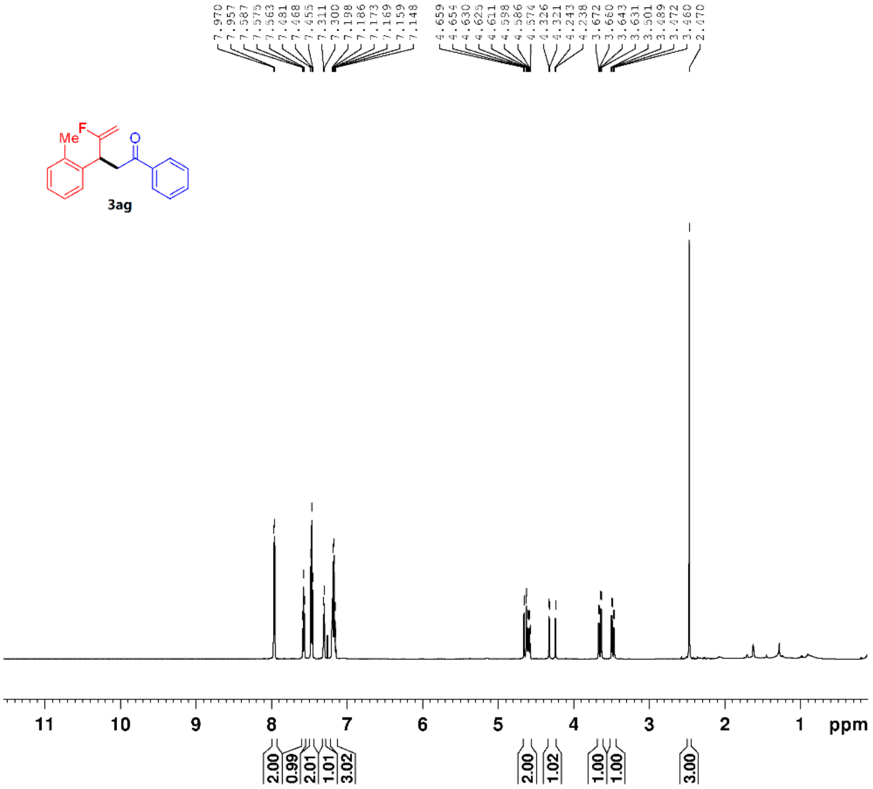
7.970
7.957
7.587
7.575
7.563
7.481
7.468
7.455
7.311
7.300
7.198
7.186
7.173
7.169
7.159
7.148
4.659
4.654
4.630
4.625
4.611
4.598
4.586
4.574
4.326
4.321
4.243
4.238
3.672
3.660
3.643
3.631
3.501
3.489
3.472
3.460
2.470
Me
F
O
3ag
11
10
9
8
7
6
5
4
3
2
1
ppm
2.00
0.99
2.01
1.01
3.02
2.00
1.02
1.00
1.00
3.00

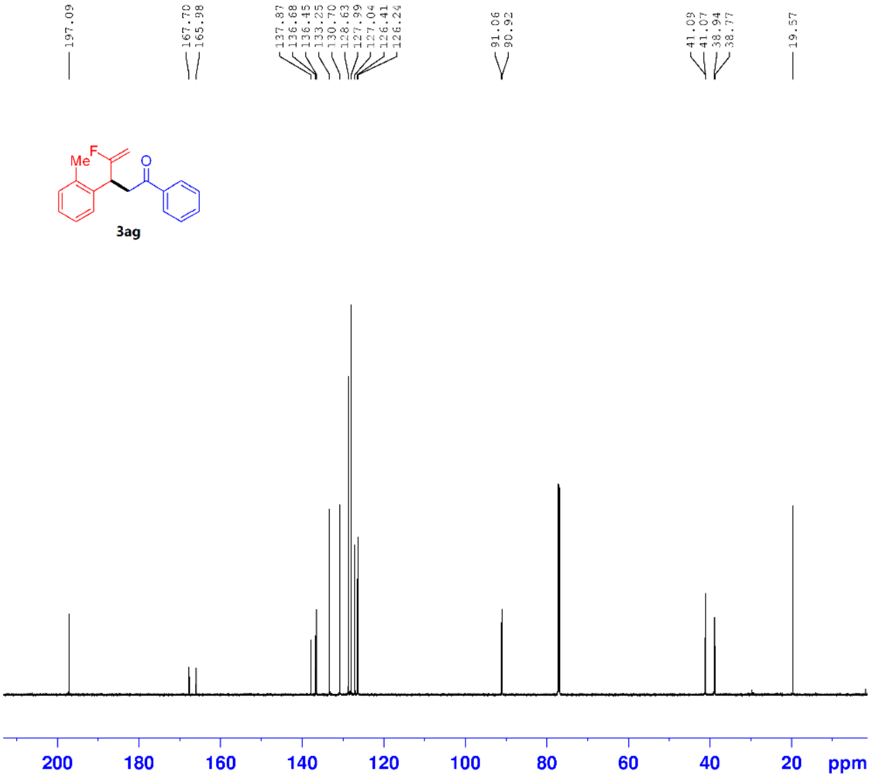
197.09
167.70
165.98
137.87
136.68
136.45
133.25
130.70
128.63
127.99
127.04
126.41
126.24
91.06
90.92
41.09
41.07
38.94
38.77
19.57
Me
F
O
3ag
200
180
160
140
120
100
80
60
40
20
ppm

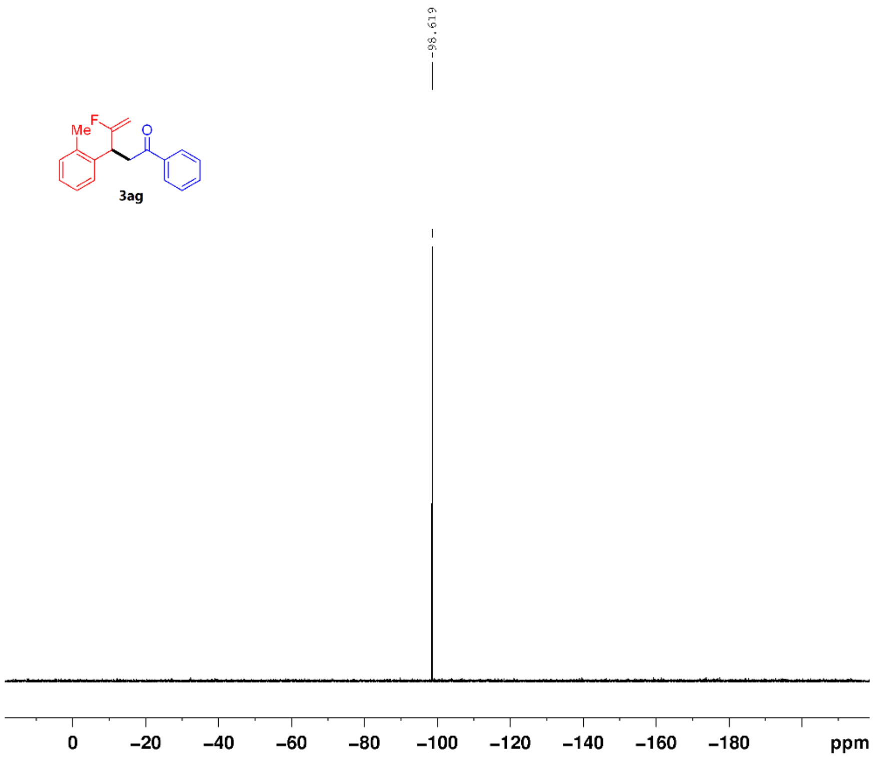

-98.619
Me
F
O
3ag
0
–20
–40
–60
–80
–100
–120
–140
–160
–180
ppm

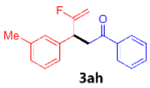

3ah

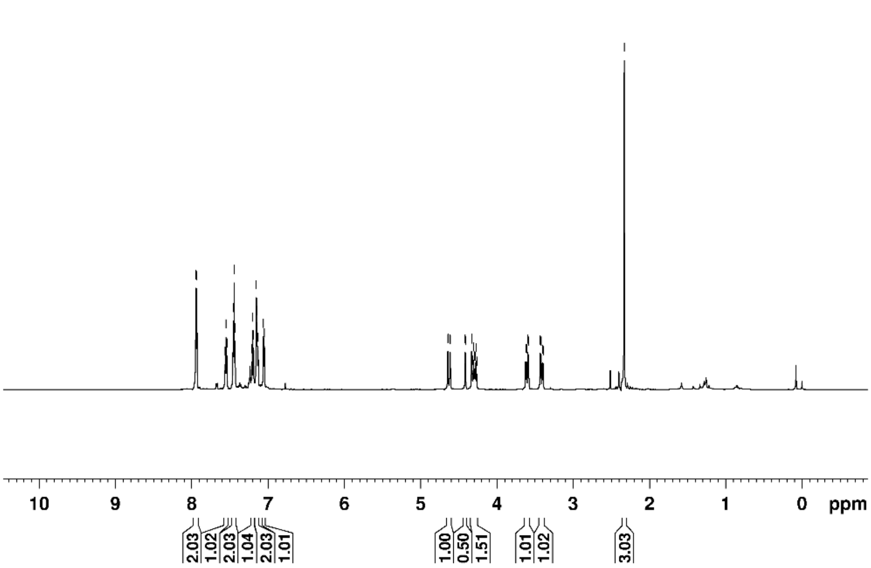

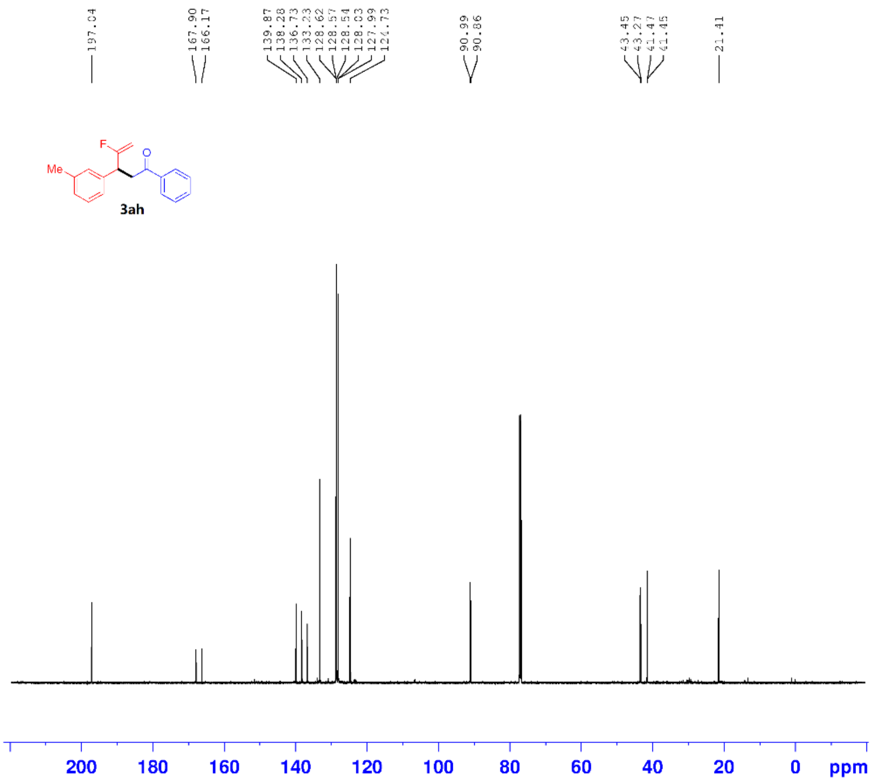
197.04
167.90
166.17
139.87
138.28
136.73
133.23
128.62
128.57
128.54
128.03
127.99
124.73
90.99
90.86
43.45
43.27
41.47
41.45
21.41
F
O
Me
3ah
200
180
160
140
120
100
80
60
40
20
0
ppm

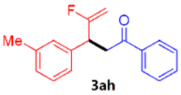

3ah

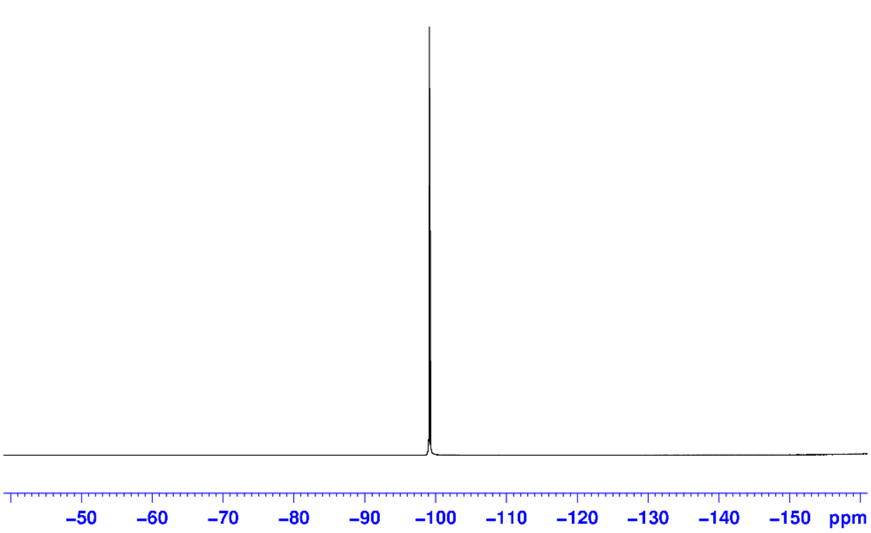

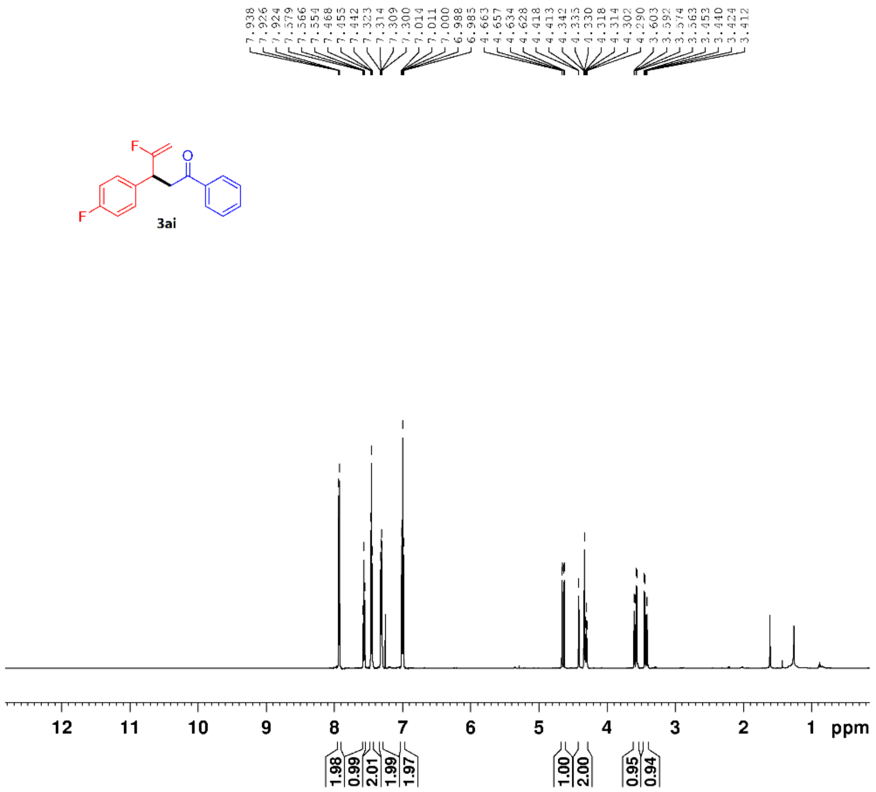
7.938
7.926
7.924
7.579
7.566
7.554
7.468
7.455
7.442
7.323
7.314
7.309
7.300
7.014
7.011
7.000
6.988
6.985
4.663
4.657
4.634
4.628
4.418
4.413
4.342
4.335
4.330
4.318
4.314
4.302
4.290
3.603
3.592
3.574
3.563
3.453
3.440
3.424
3.412
F
O
F
3ai
12
11
10
9
8
7
6
5
4
3
2
1
ppm
1.98
0.99
2.01
1.99
1.97
1.00
2.00
0.95
0.94

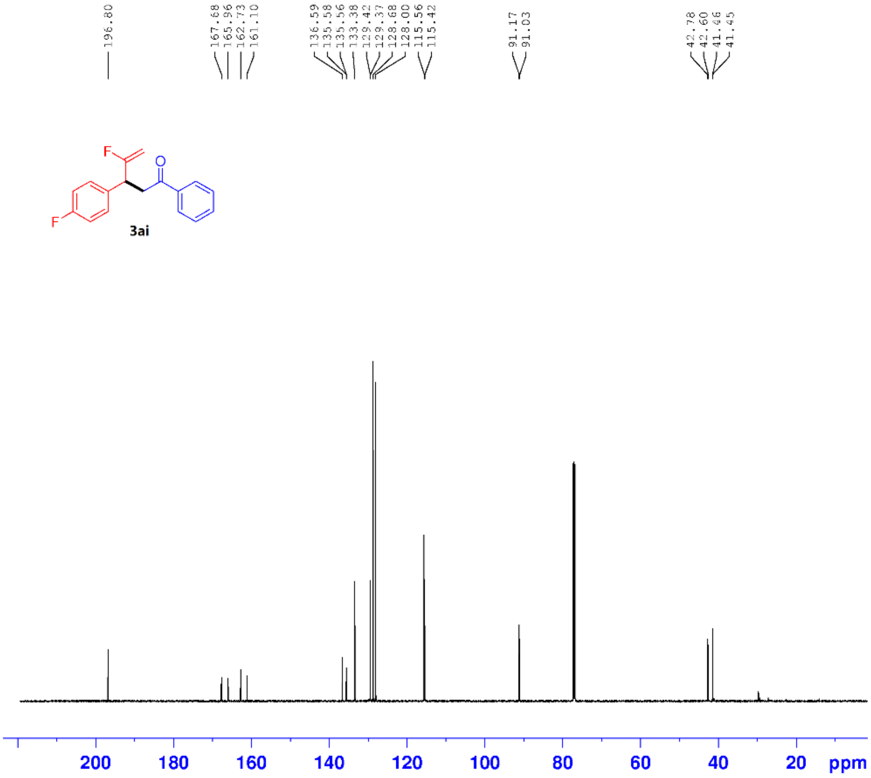
196.80
167.68
165.96
162.73
161.10
136.59
135.58
135.56
133.38
129.42
129.37
128.68
128.00
115.56
115.42
91.17
91.03
42.78
42.60
41.46
41.45
F
O
F
3ai
200
180
160
140
120
100
80
60
40
20
ppm

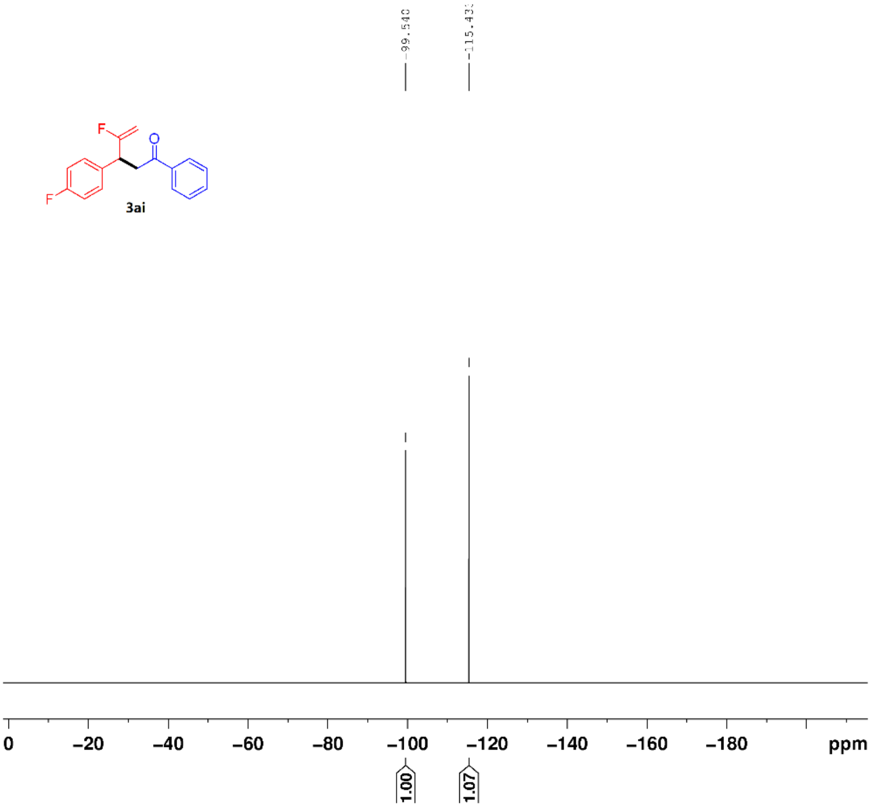

F
O
F
3ai
-99.540
-115.431
0
–20
–40
–60
–80
–100
–120
–140
–160
–180
ppm
1.00
1.07

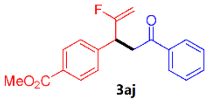

3aj

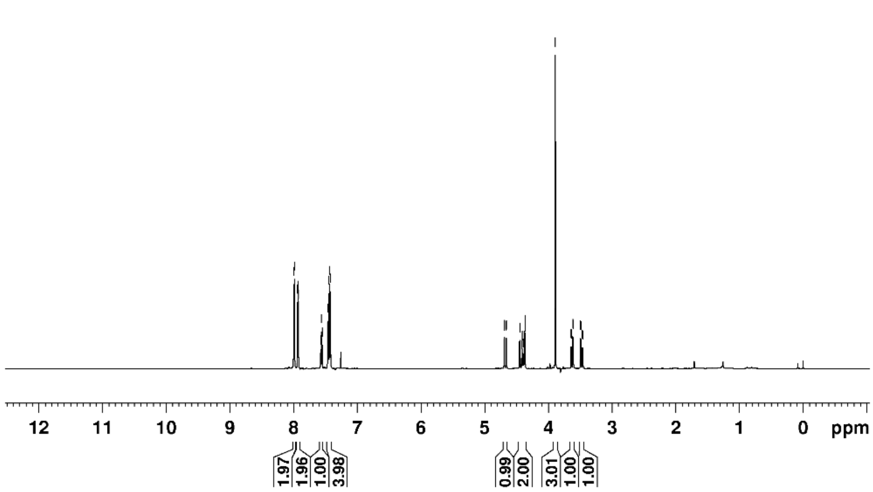

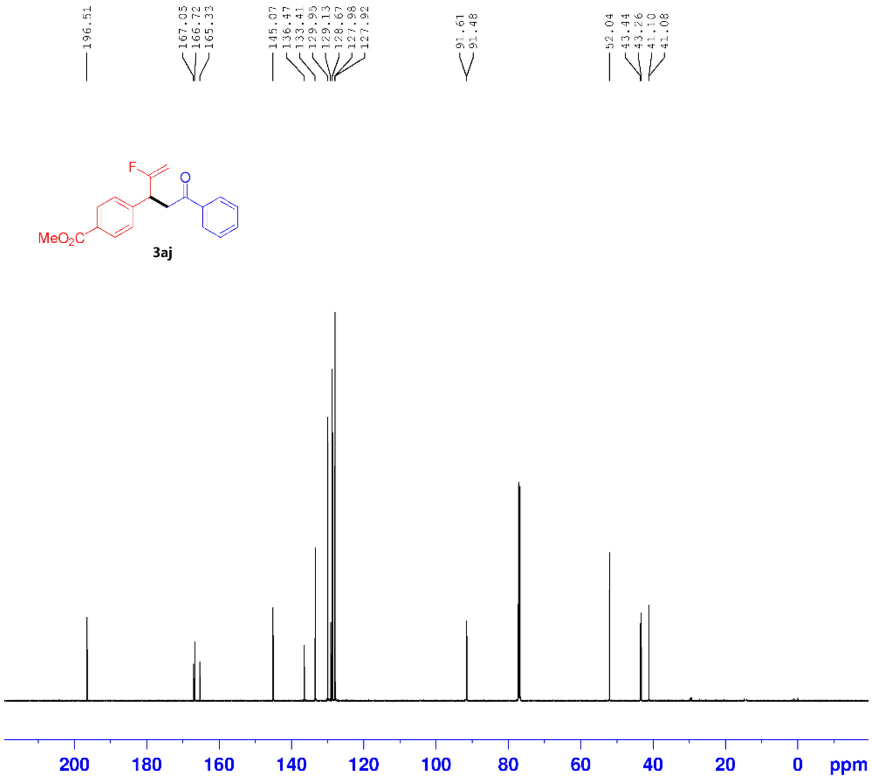
196.51
167.05
166.72
165.33
145.07
136.47
133.41
129.95
129.13
128.67
127.98
127.92
91.61
91.48
52.04
43.44
43.26
41.10
41.08
F
O
MeO2C
3aj
200
180
160
140
120
100
80
60
40
20
0
ppm

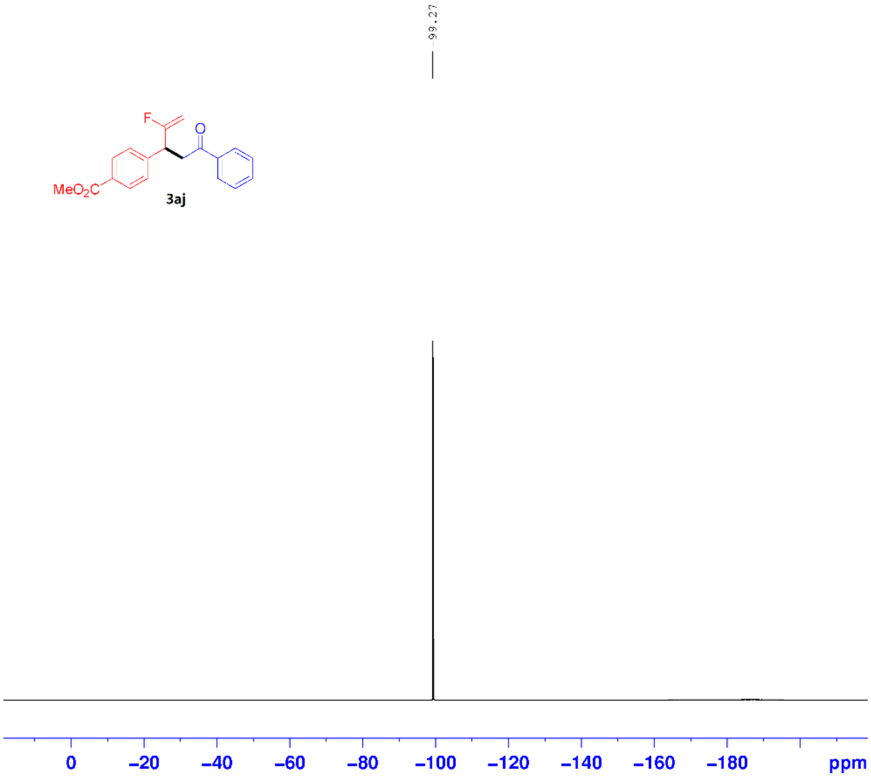
99.27
F
O
MeO2C
3aj
0
–20
–40
–60
–80
–100
–120
–140
–160
–180
ppm

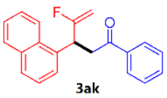

F
O

3ak

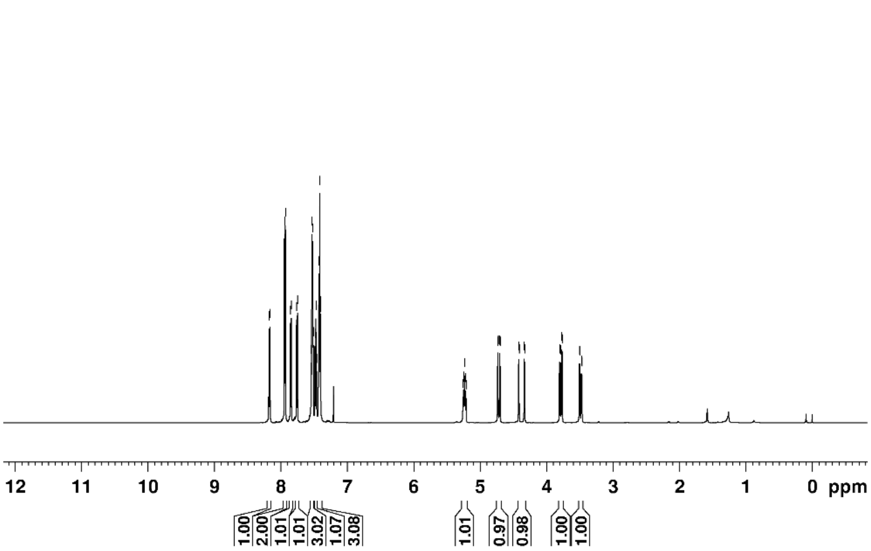

8.178
8.164
7.945
7.932
7.857
7.844
7.763
7.544
7.533
7.525
7.521
7.513
7.486
7.473
7.462
7.431
7.427
7.405
7.401
5.259
5.249
5.246
5.235
5.225
5.221
4.736
4.731
4.706
4.701
4.428
4.336
4.331
3.805
3.791
3.776
3.762
3.505
3.497
3.476
3.467
12
11
10
9
8
7
6
5
4
3
2
1
0 ppm
1.00
2.00
1.01
1.01
3.02
1.07
3.08
1.01
0.97
0.98
1.00
1.00

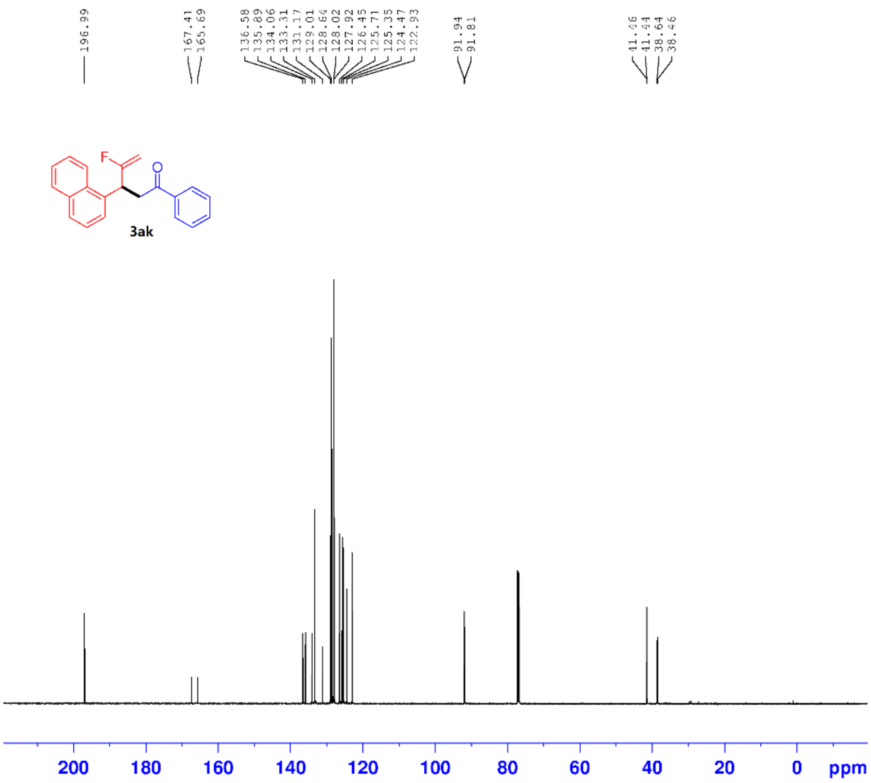
196.99
167.41
165.69
136.58
135.89
134.06
133.31
131.17
129.01
128.64
128.02
127.92
126.45
125.71
125.35
124.47
122.93
91.94
91.81
41.46
41.44
38.64
38.46
F
O
3ak
200
180
160
140
120
100
80
60
40
20
0
ppm

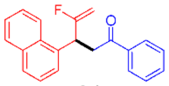

3ak

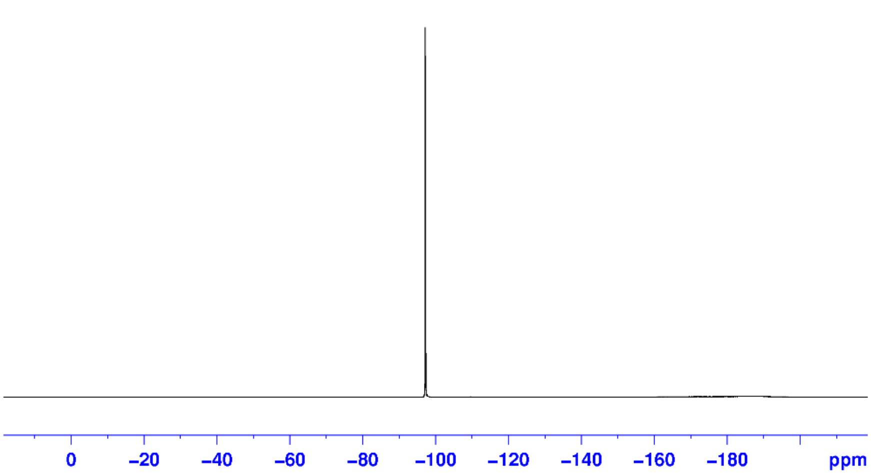

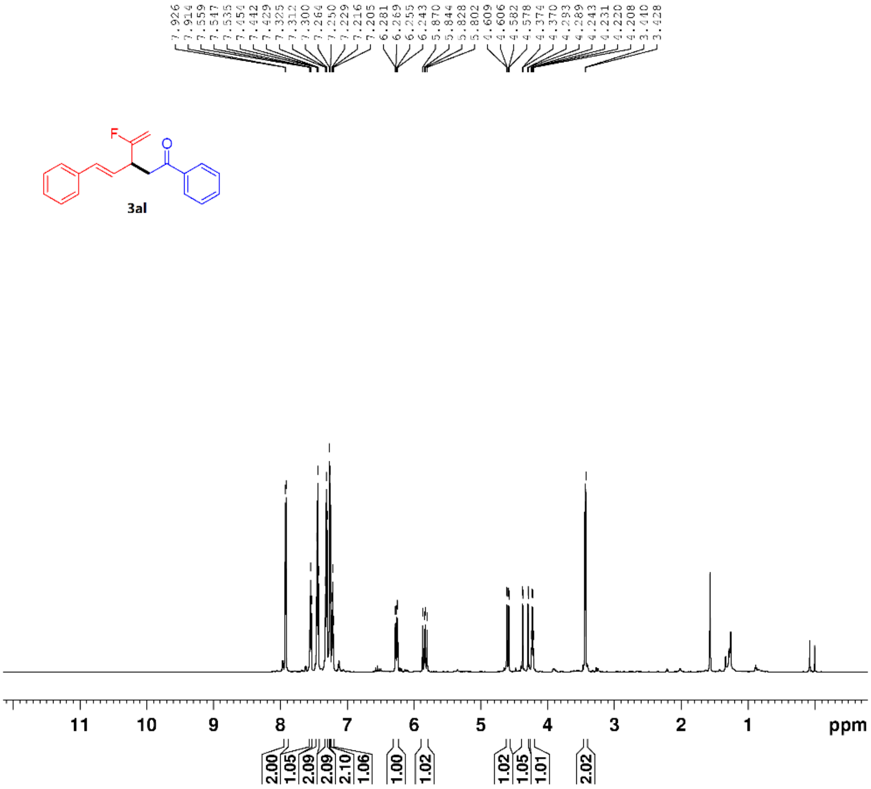

3al
ppm

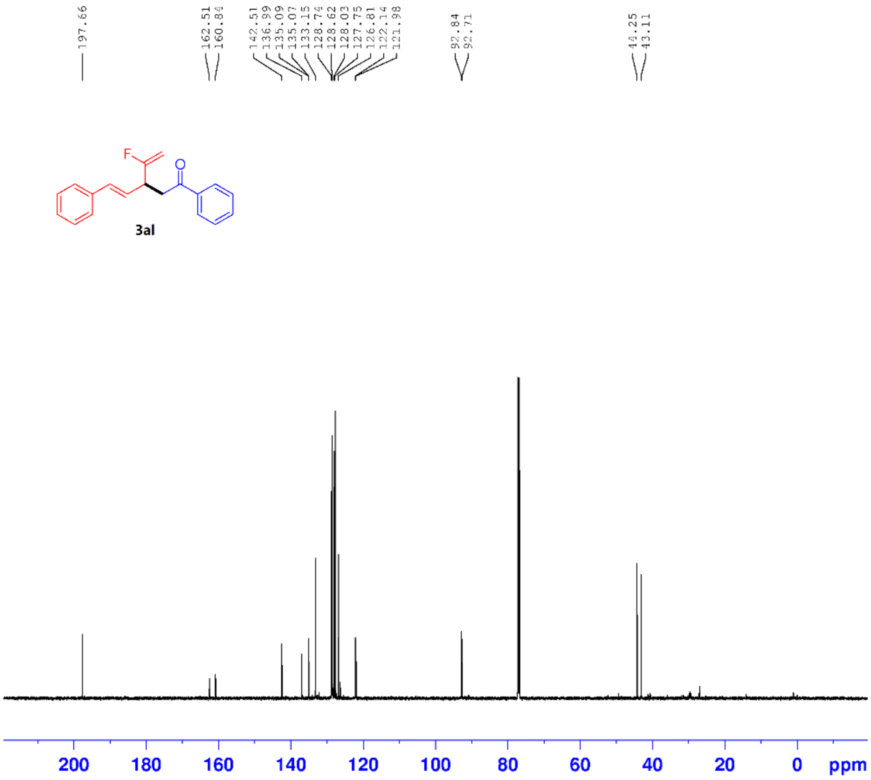
197.66
162.51
160.84
142.51
136.99
135.09
135.07
133.15
128.74
128.62
128.03
127.75
126.81
122.14
121.98
92.84
92.71
44.25
43.11
F
O
3al
200
180
160
140
120
100
80
60
40
20
0
ppm

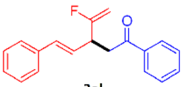

3al

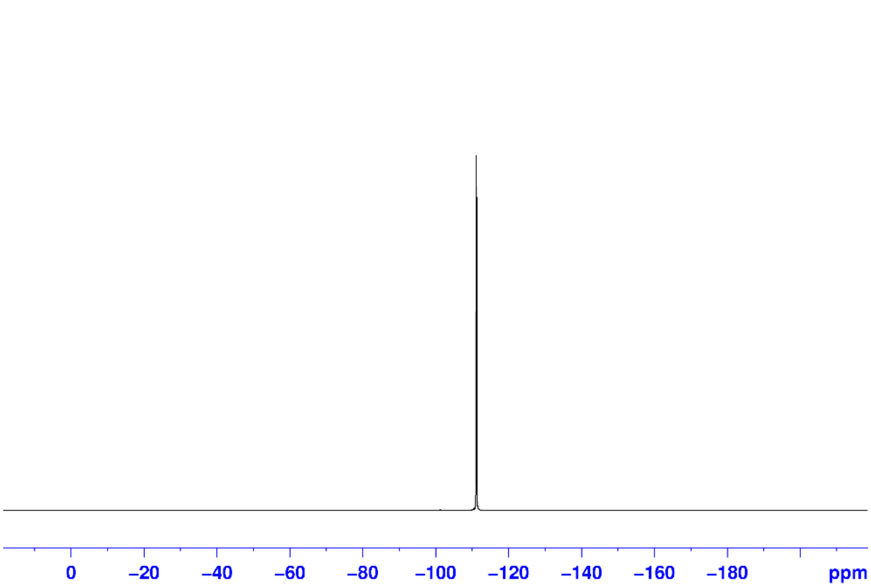

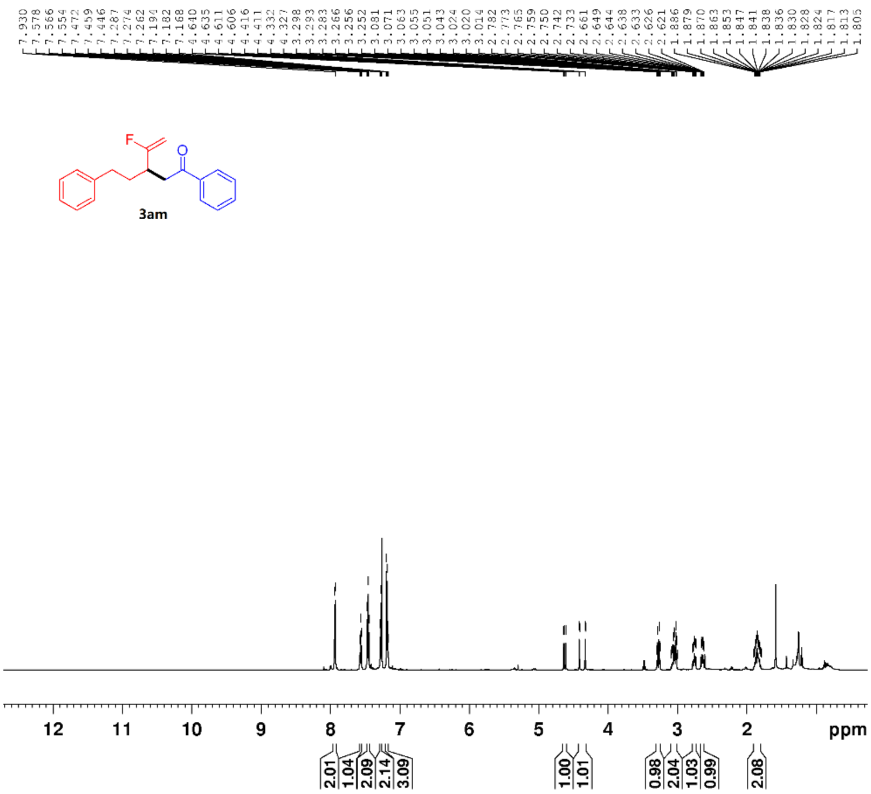

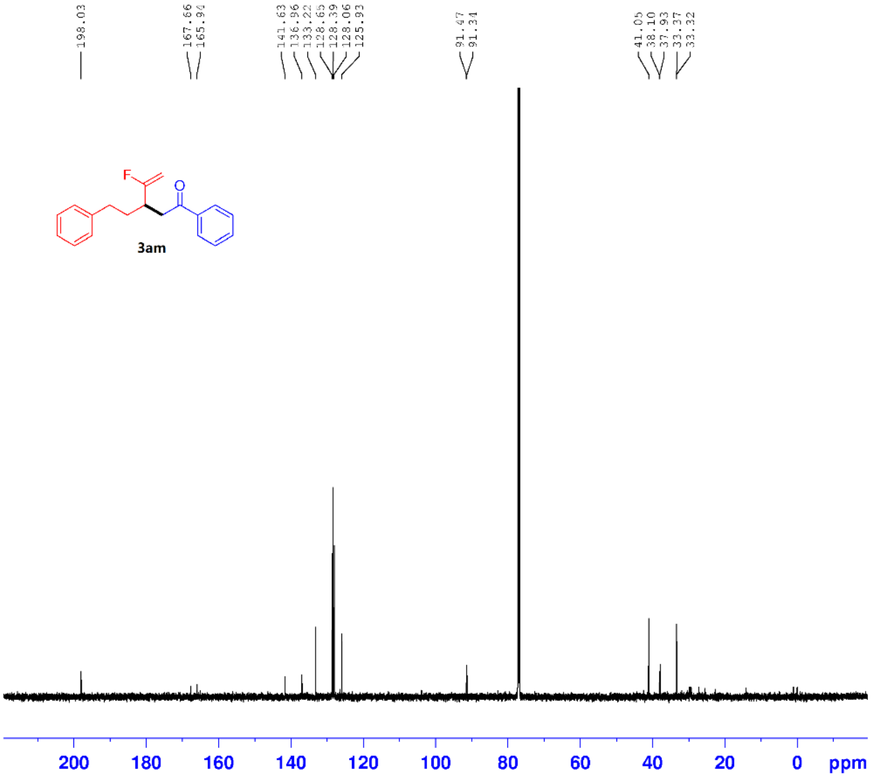
198.03
167.66
165.94
141.63
136.96
133.22
128.65
128.39
128.06
125.93
91.47
91.34
41.05
38.10
37.93
33.37
33.32
F
O
3am
200
180
160
140
120
100
80
60
40
20
0
ppm

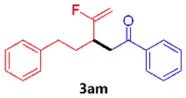

3am

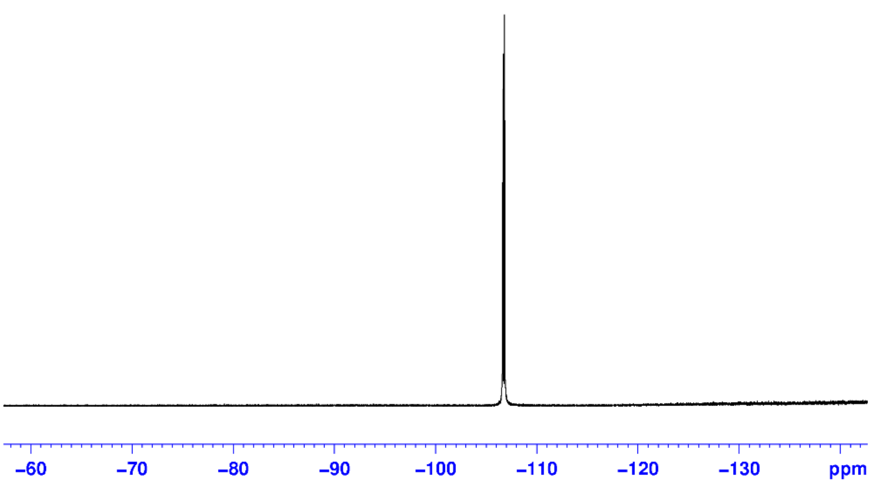

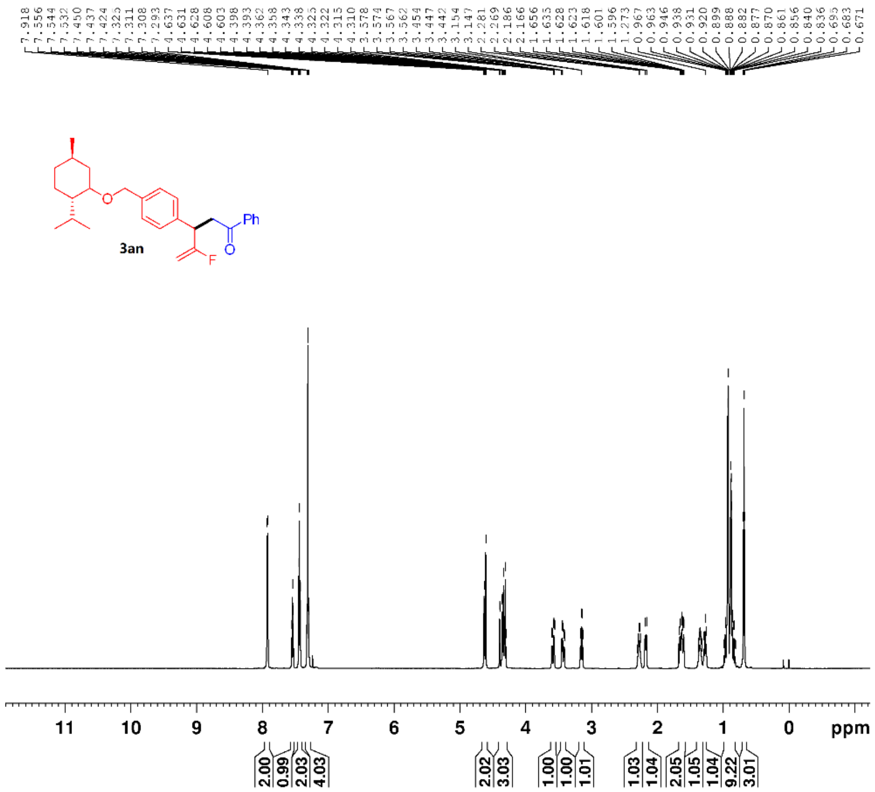

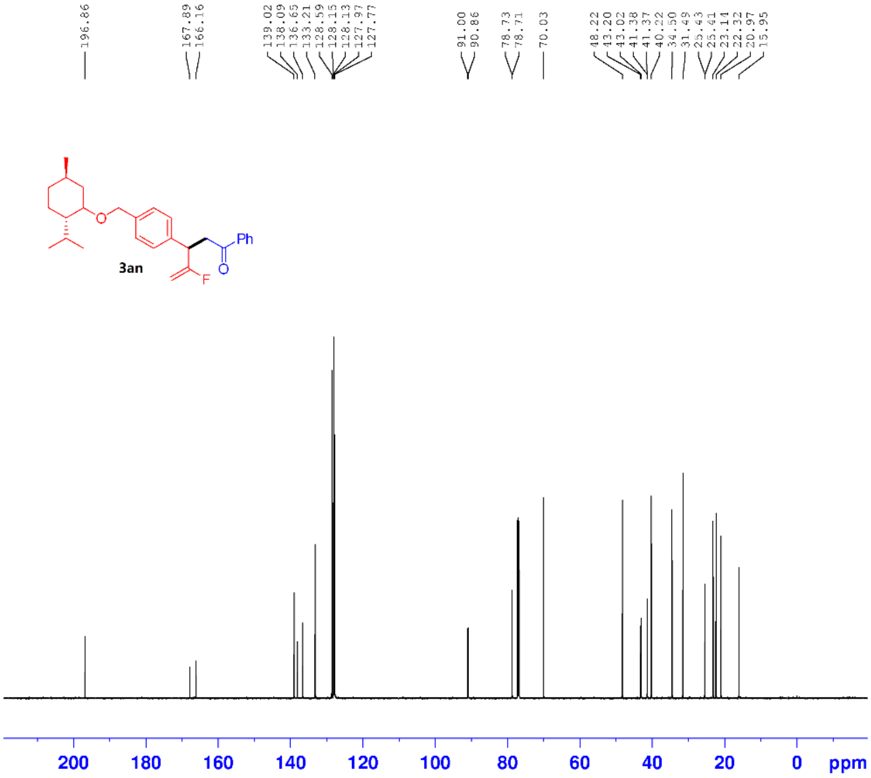
196.86
167.89
166.16
139.02
138.09
136.65
133.21
128.59
128.15
128.13
127.97
127.77
91.00
90.86
78.73
78.71
70.03
48.22
43.20
43.02
41.38
41.37
40.22
34.50
31.49
25.43
25.41
23.11
22.32
20.97
15.95
O
Ph
O
F
3an
200
180
160
140
120
100
80
60
40
20
0
ppm

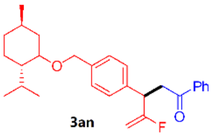

3an

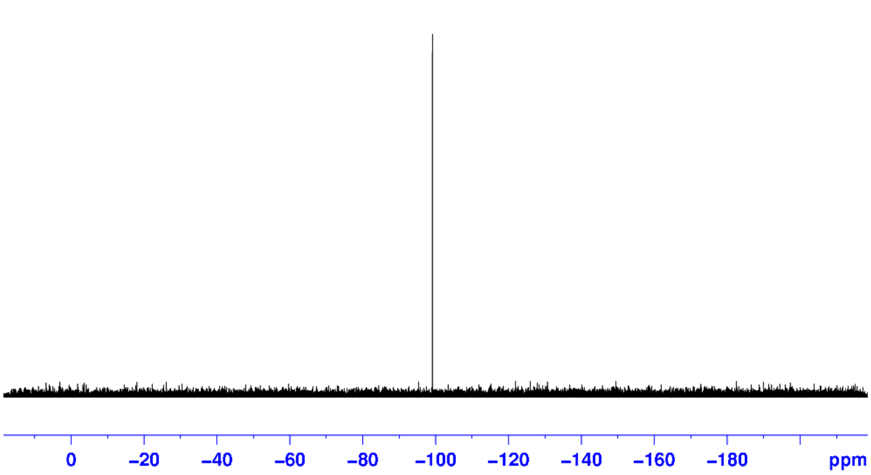

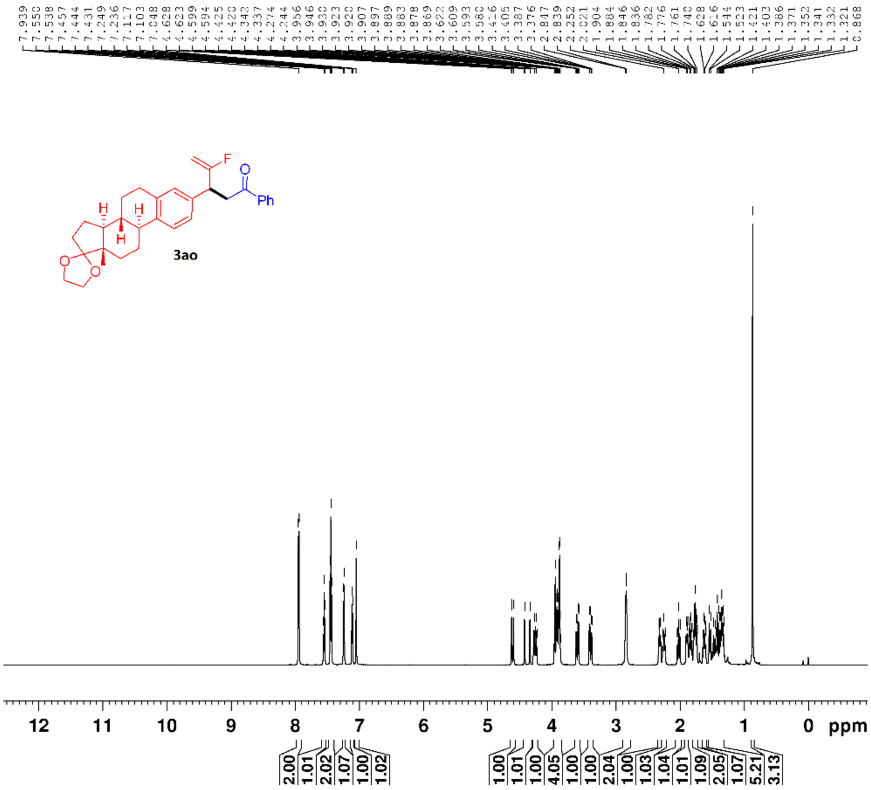

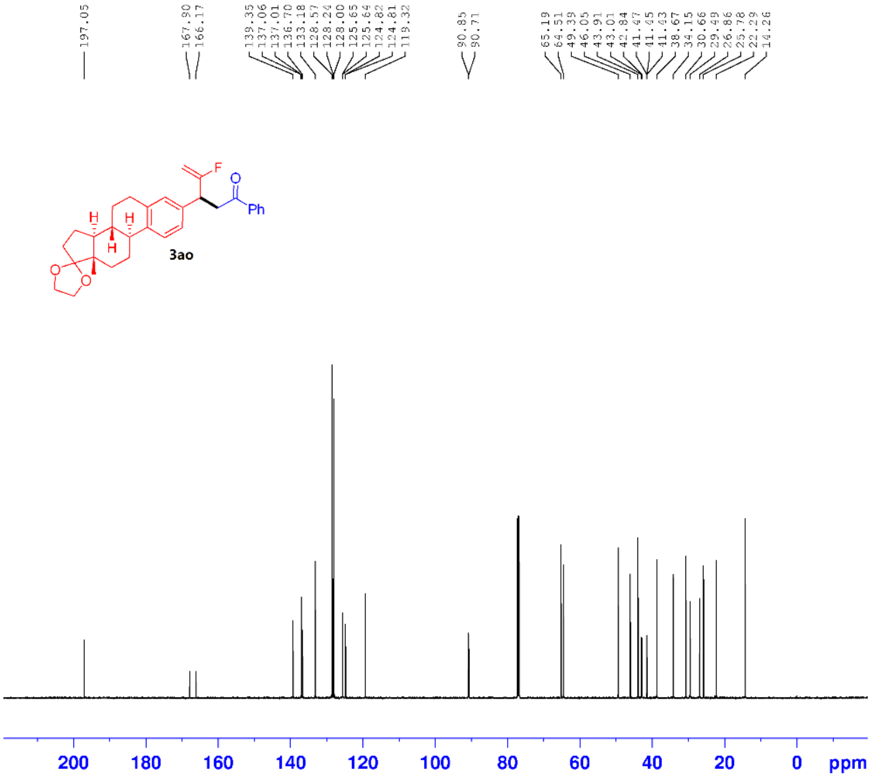
197.05
167.90
166.17
139.35
137.06
137.01
136.70
133.18
128.57
128.24
128.00
125.65
125.64
124.82
124.81
119.32
90.85
90.71
65.19
64.51
49.39
46.05
43.91
43.01
42.84
41.47
41.45
41.43
38.67
34.15
30.66
29.49
26.86
25.78
22.29
14.26
F
O
Ph
H
H
H
O
O
3ao
200
180
160
140
120
100
80
60
40
20
0
ppm

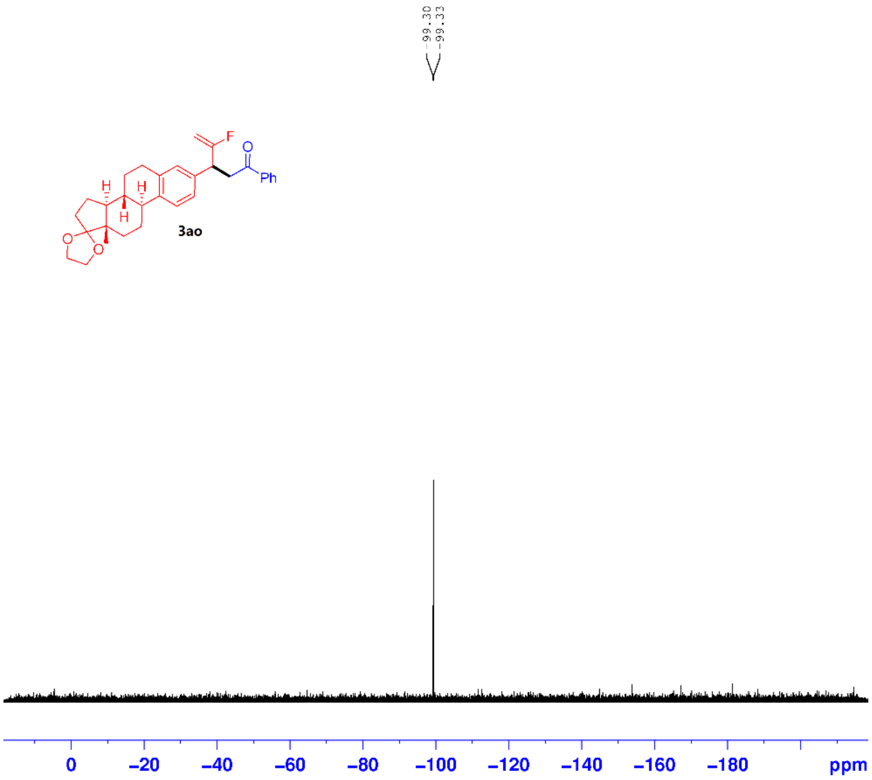
-99.30
-99.33
F
O
Ph
H
H
H
O
O
3ao
0
-20
-40
-60
-80
-100
-120
-140
-160
-180
ppm

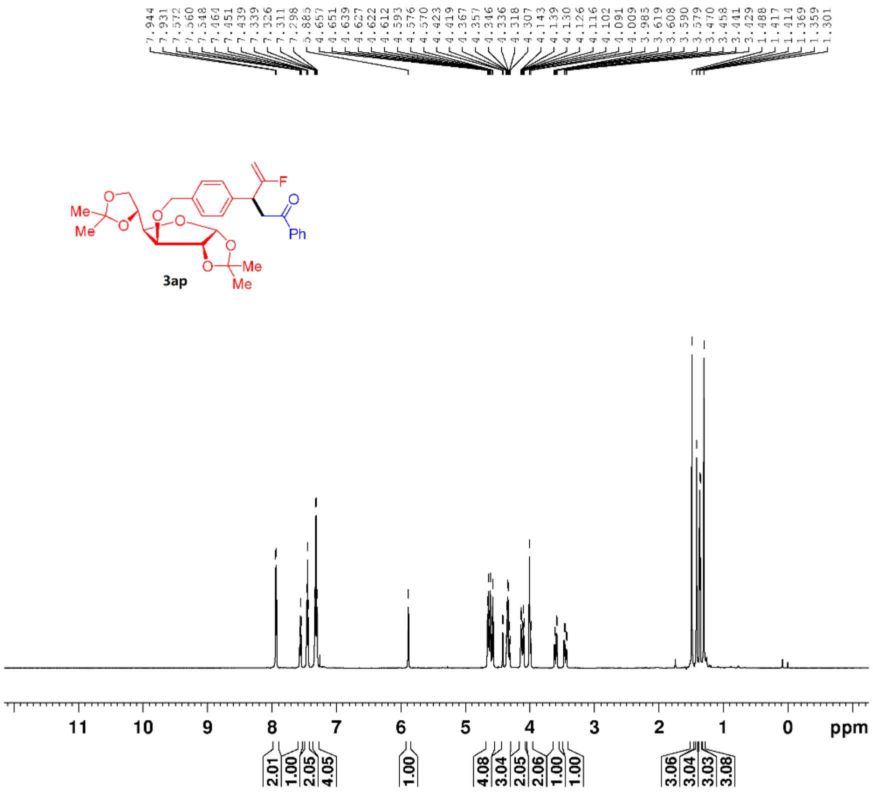

7.944
7.931
7.560
7.548
7.464
7.451
7.439
7.339
7.326
7.311
7.298
5.885
4.657
4.651
4.639
4.627
4.622
4.612
4.593
4.576
4.570
4.423
4.419
4.367
4.357
4.346
4.336
4.318
4.307
4.143
4.139
4.130
4.126
4.116
4.102
4.091
4.009
3.985
3.619
3.608
3.590
3.579
3.470
3.458
3.441
3.429
1.488
1.417
1.414
1.369
1.359
1.301
F
O
Ph
Me
Me
O
O
O
O
O
O
Me
Me
3ap
11
10
9
8
7
6
5
4
3
2
1
0
ppm
2.01
1.00
2.05
4.05
1.00
4.08
3.04
2.05
2.06
1.00
1.00
3.06
3.04
3.03
3.08

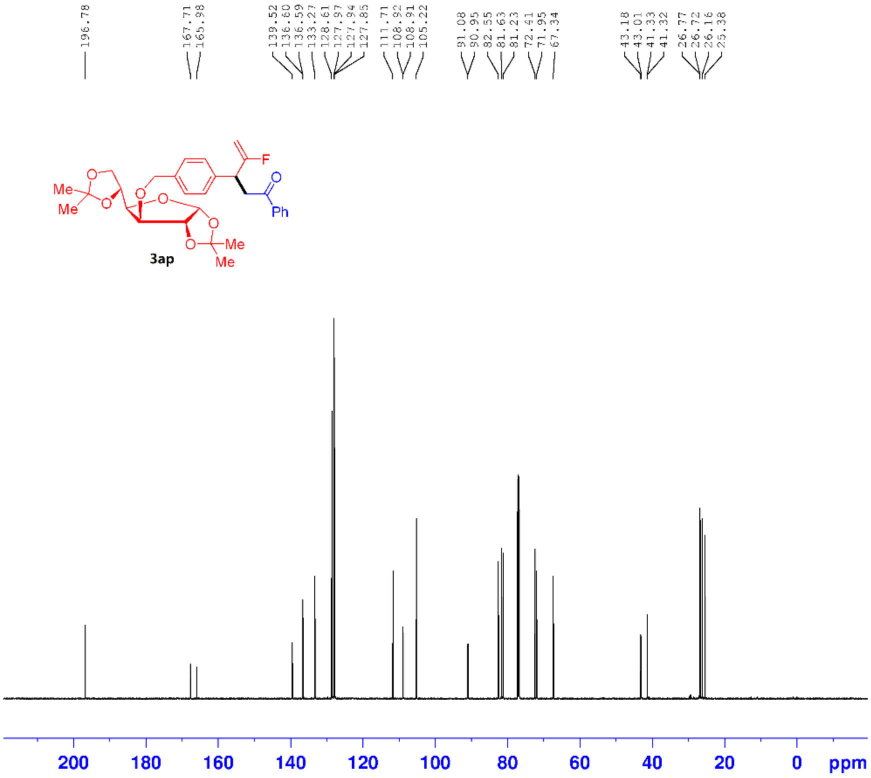
196.78
167.71
165.98
139.52
136.60
136.59
133.27
128.61
127.97
127.94
127.85
111.71
108.92
108.91
105.22
91.08
90.95
82.55
81.63
81.23
72.41
71.95
67.34
43.18
43.01
41.33
41.32
26.77
26.72
26.16
25.38
F
O
Ph
Me
Me
O
O
O
O
O
O
Me
Me
3ap
200
180
160
140
120
100
80
60
40
20
0
ppm

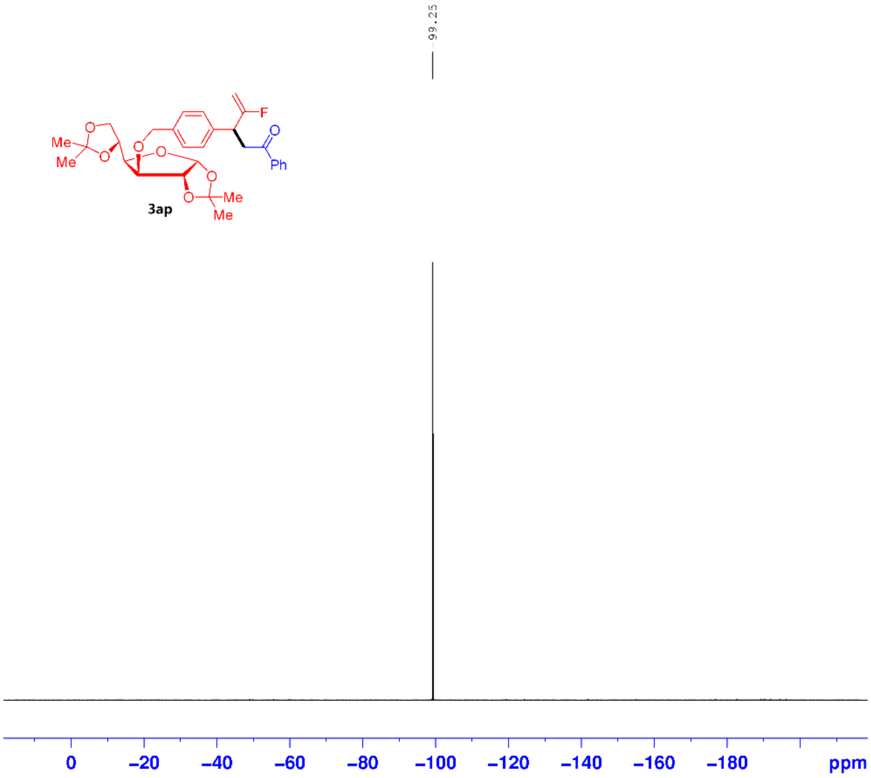

99.25
Me
Me
O
O
O
O
O
O
F
O
Ph
Me
Me
3ap
0
–20
–40
–60
–80
–100
–120
–140
–160
–180
ppm

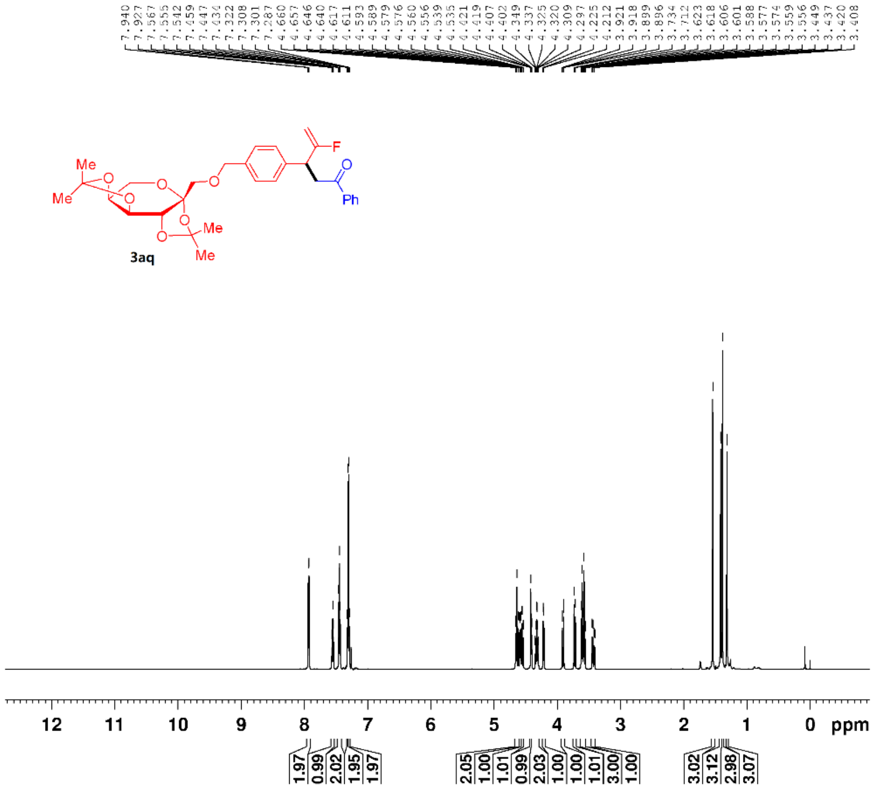
7.940 7.927 7.567 7.555 7.542 7.459 7.447 7.434 7.322 7.308 7.301 7.287 4.660 4.657 4.646 4.640 4.617 4.611 4.593 4.589 4.579 4.576 4.560 4.556 4.539 4.535 4.421 4.419 4.407 4.402 4.349 4.337 4.325 4.320 4.309 4.297 4.225 4.212 3.921 3.918 3.899 3.896 3.734 3.712 3.623 3.618 3.606 3.601 3.588 3.577 3.574 3.559 3.556 3.449 3.437 3.420 3.408
Me
Me
O
O
O
O
O
O
Me
Me
F
O
Ph
3aq
12 11 10 9 8 7 6 5 4 3 2 1 0 ppm
1.97 0.99 2.02 1.95 1.97
2.05 1.00 1.01 0.99 2.03 1.00 1.00 1.01 3.00 1.00
3.02 3.12 2.98 3.07

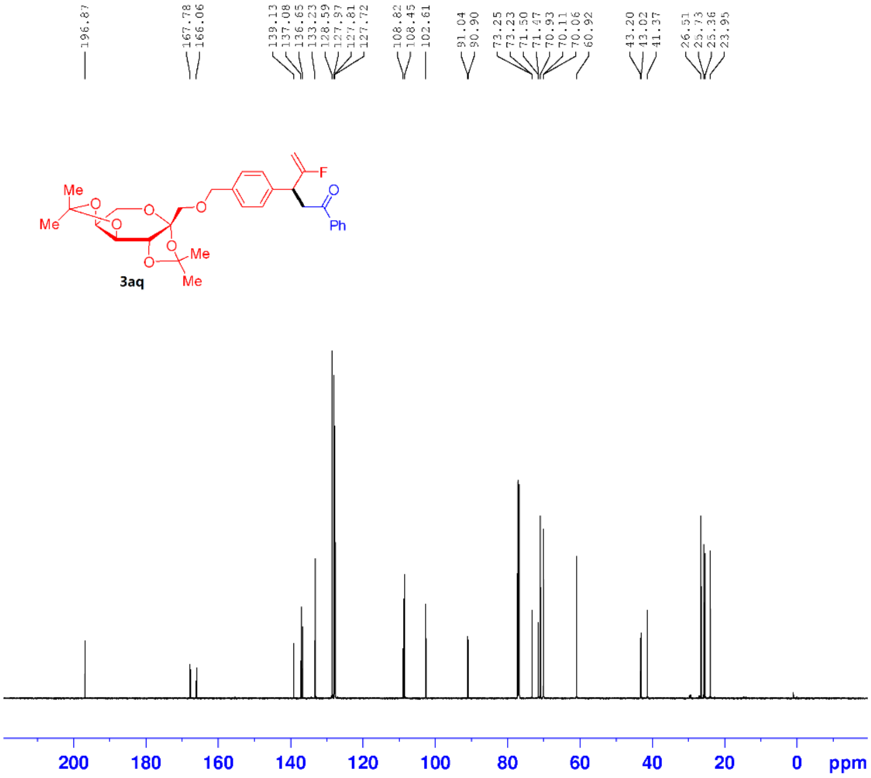
196.87
167.78
166.06
139.13
137.08
136.65
133.23
128.59
127.97
127.81
127.72
108.82
108.45
102.61
91.04
90.90
73.25
73.23
71.50
71.47
70.93
70.11
70.06
60.92
43.20
43.02
41.37
26.51
25.73
25.36
23.95
Me
O
O
Me
O
O
O
Me
Me
O
F
O
Ph
3aq
200
180
160
140
120
100
80
60
40
20
0
ppm

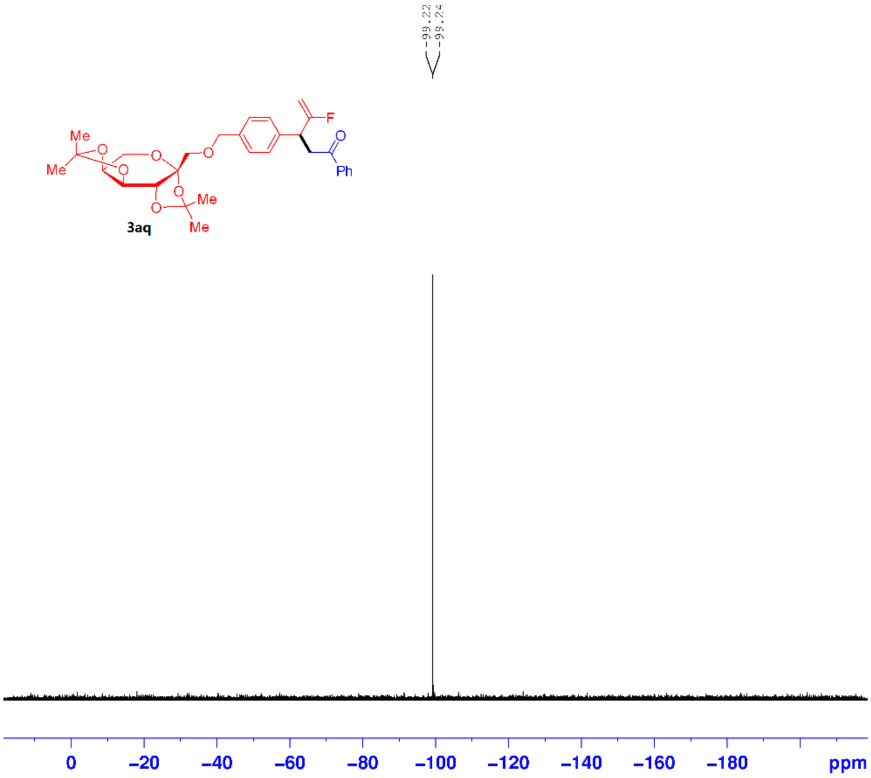
-99.22
-99.24
Me
Me
O
O
O
O
O
O
Me
Me
F
O
Ph
0
–20
–40
–60
–80
–100
–120
–140
–160
–180
ppm

3aq

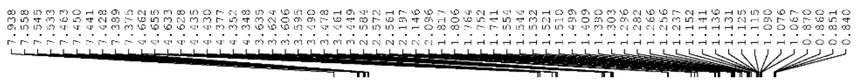

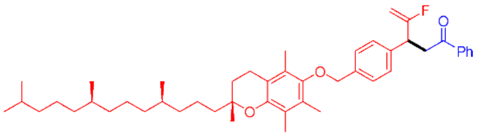

3ar

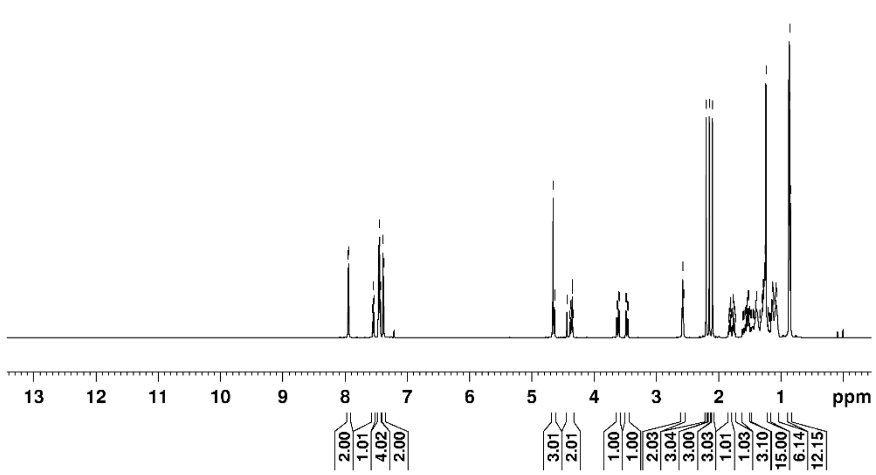

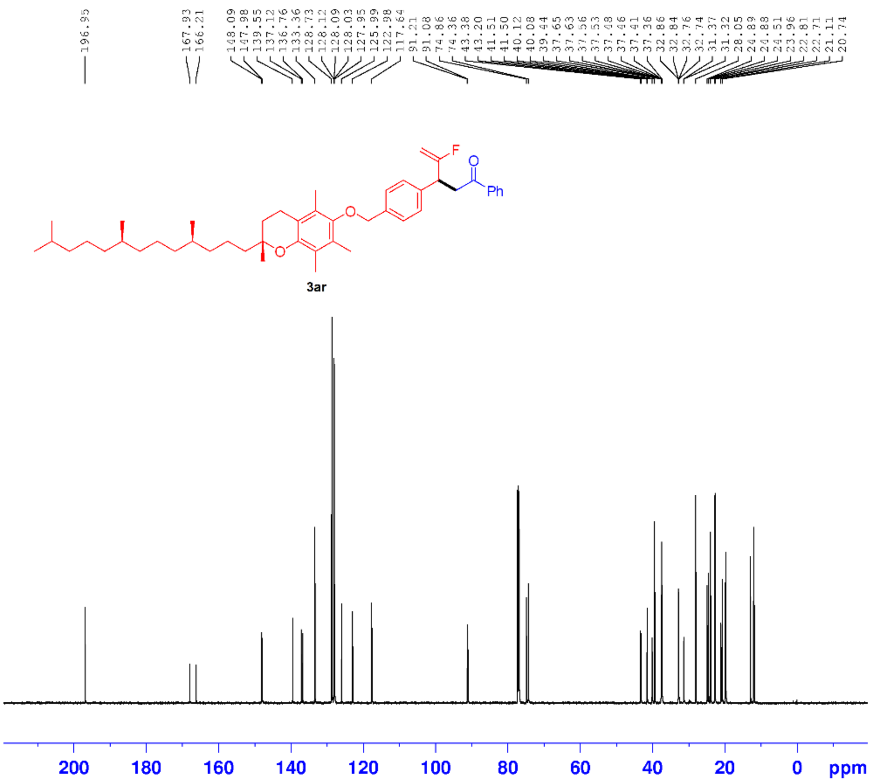
3ar

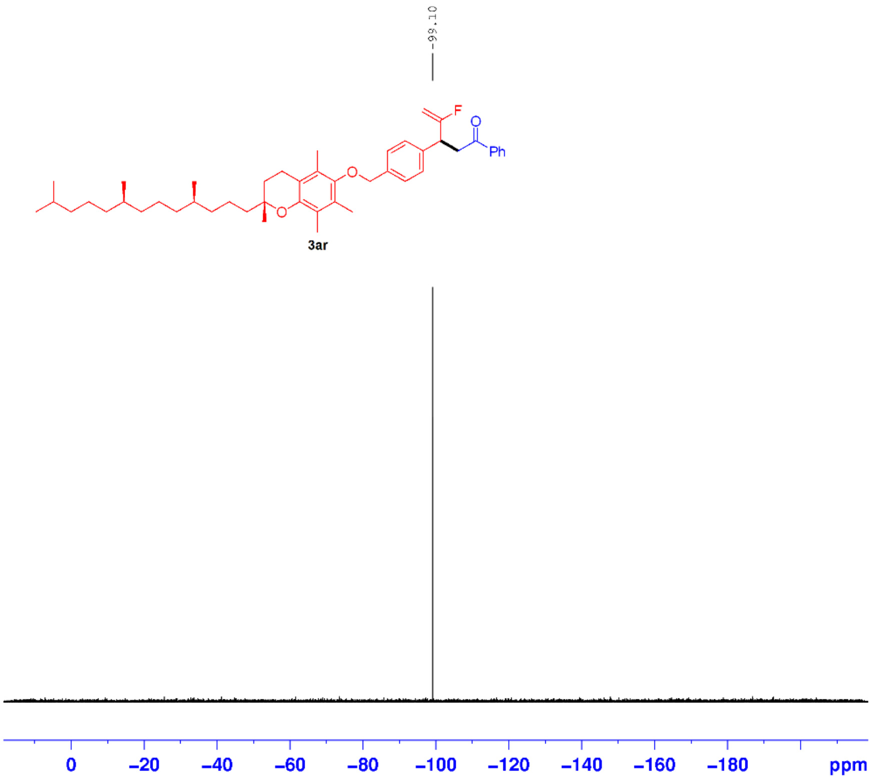
-99.10
F
O
Ph
O
O
3ar
0
-20
-40
-60
-80
-100
-120
-140
-160
-180
ppm

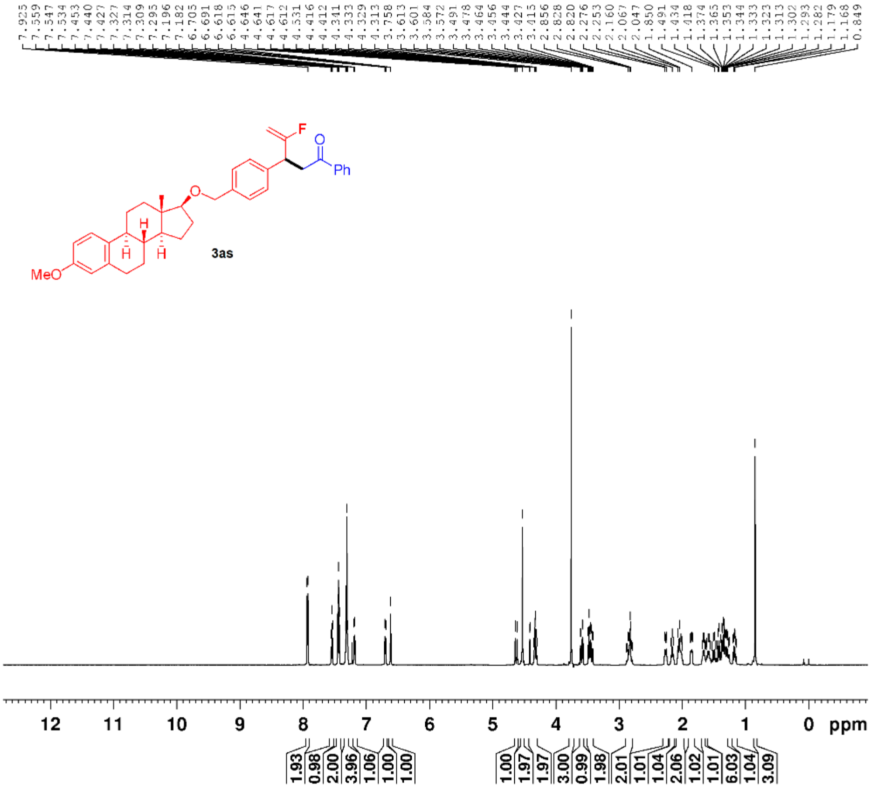

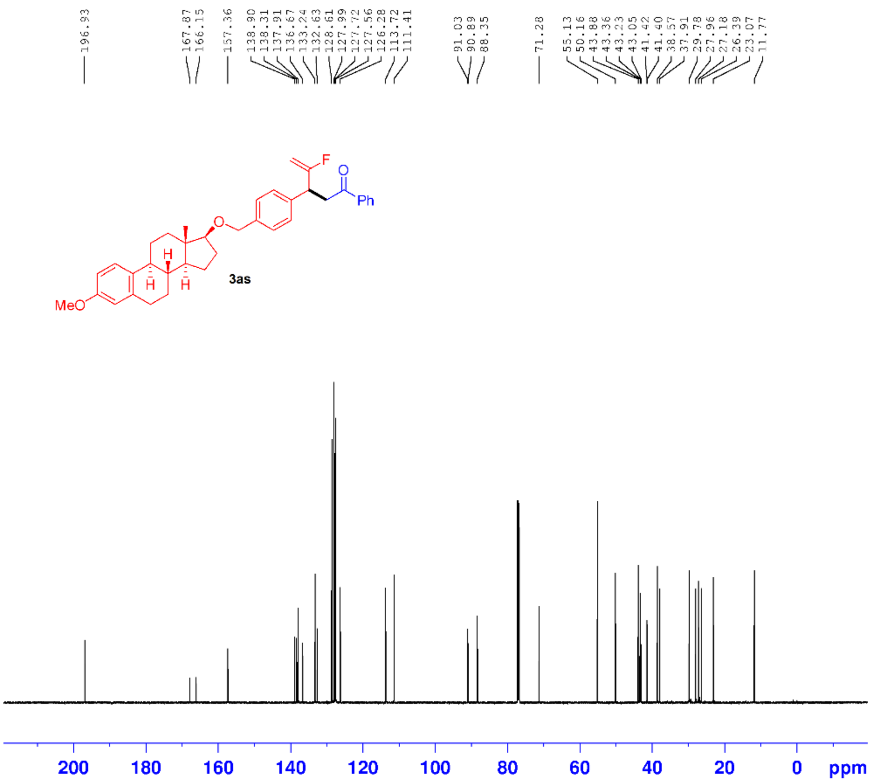
196.93
167.87
166.15
157.36
138.90
138.31
137.91
136.67
133.24
132.63
128.61
127.99
127.72
127.56
126.28
113.72
111.41
91.03
90.89
88.35
71.28
55.13
50.16
43.88
43.36
43.23
43.05
41.42
41.40
38.57
37.91
29.78
27.96
27.18
26.39
23.07
11.77
F
O
Ph
O
H
H
H
MeO
3as
200
180
160
140
120
100
80
60
40
20
0
ppm

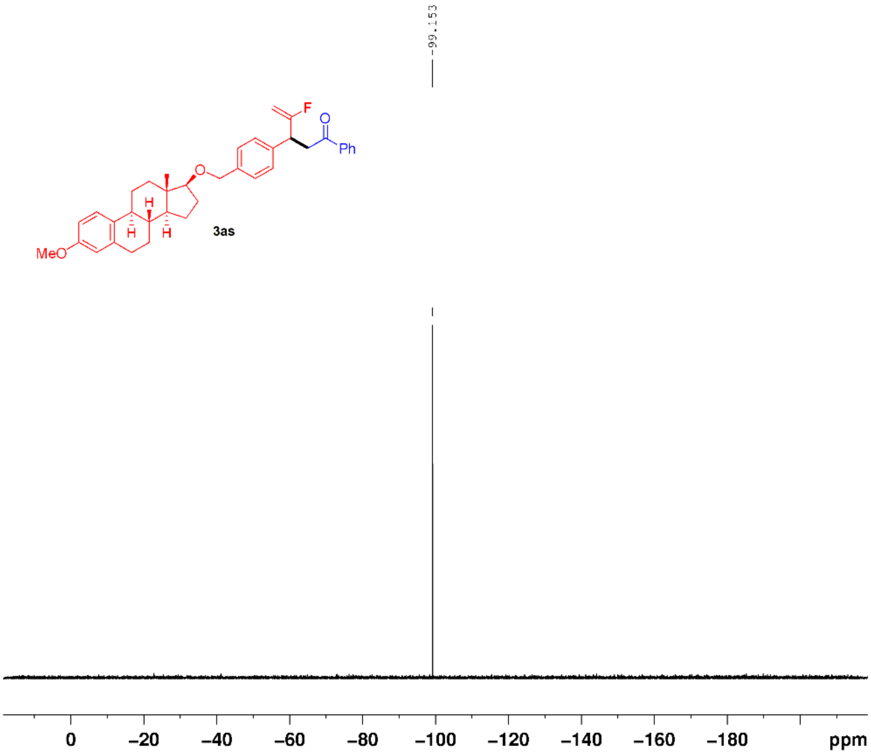
-99.153
F
O
Ph
O
H
H
H
MeO
3as
0
–20
–40
–60
–80
–100
–120
–140
–160
–180
ppm

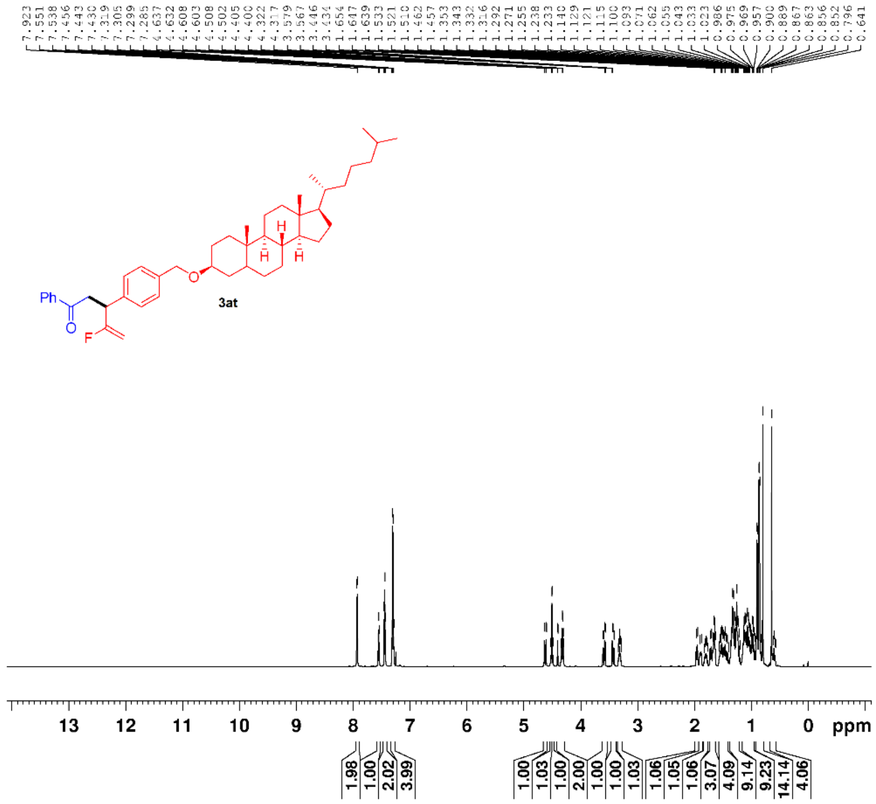

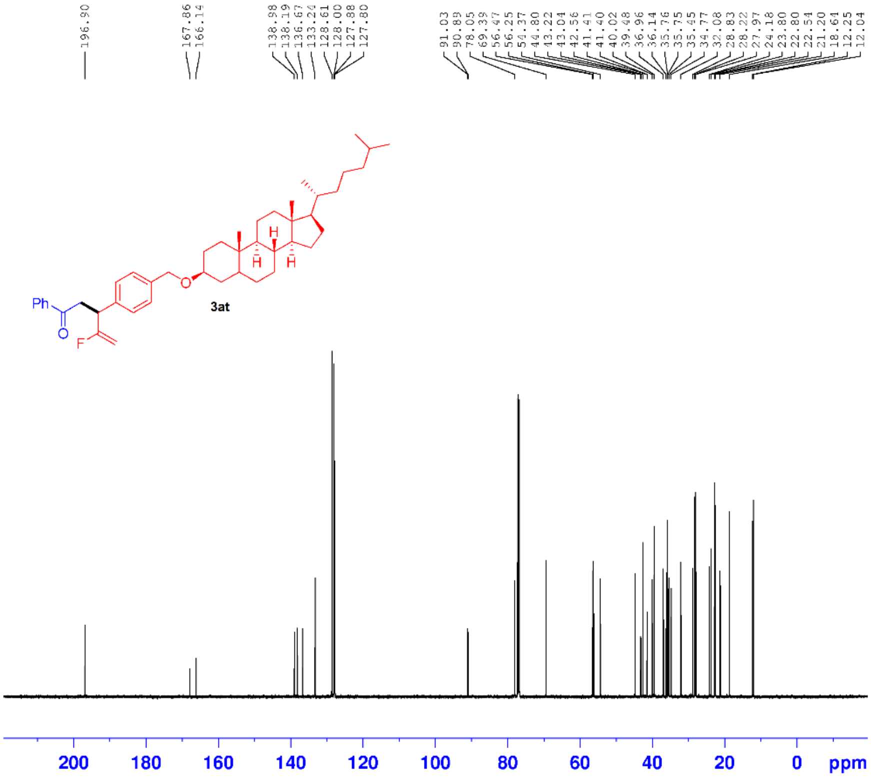
196.90
167.86
166.14
138.98
138.19
136.67
133.24
128.61
128.00
127.88
127.80
91.03
90.89
78.05
69.39
56.47
56.25
54.37
44.80
43.22
43.04
42.56
41.41
41.40
40.02
39.48
36.96
36.14
35.76
35.75
35.45
34.77
32.08
28.83
28.22
27.97
24.18
23.80
22.80
22.54
21.20
18.61
12.25
12.04
Ph
O
F
O
H
H
H
3at
200
180
160
140
120
100
80
60
40
20
0
ppm

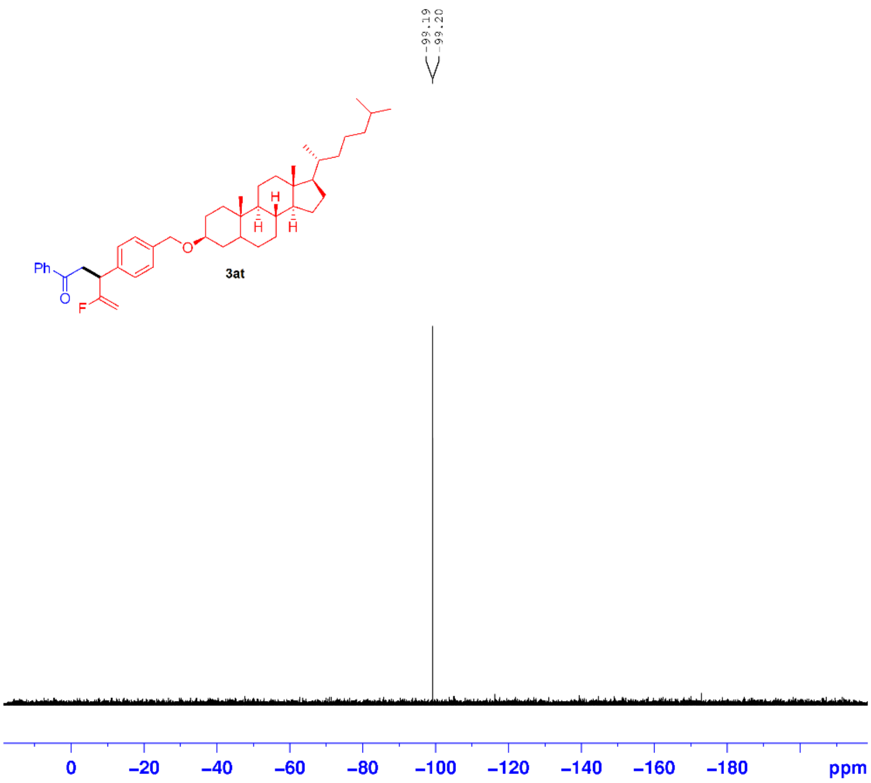

-99.19
-99.20
Ph
O
F
3at
H
H
H
0
-20
-40
-60
-80
-100
-120
-140
-160
-180
ppm

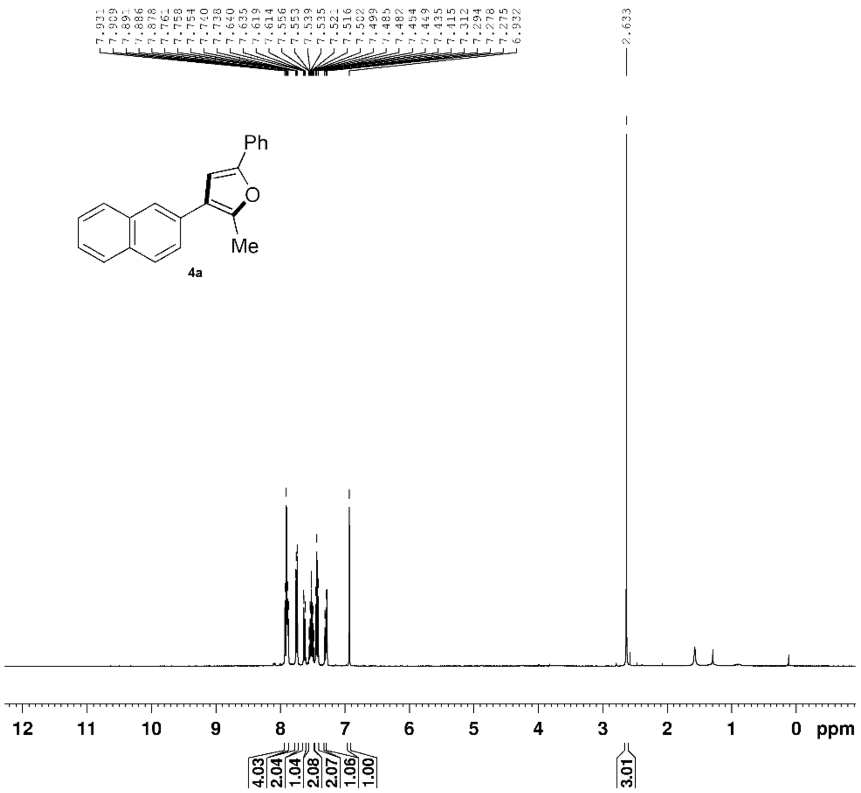

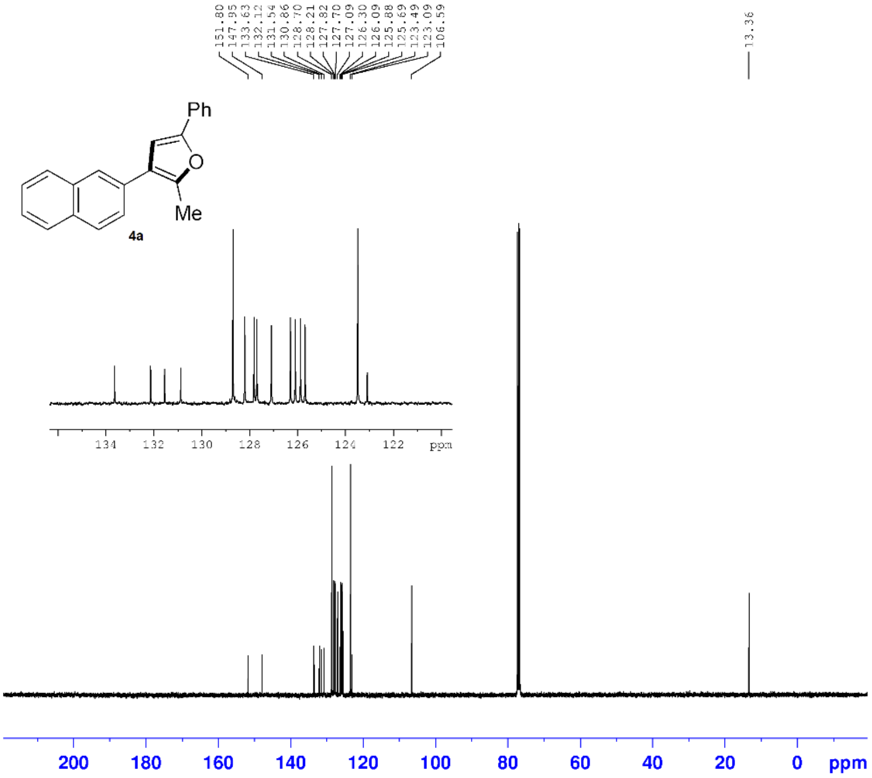

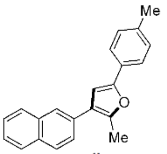
Me
O
Me

4b

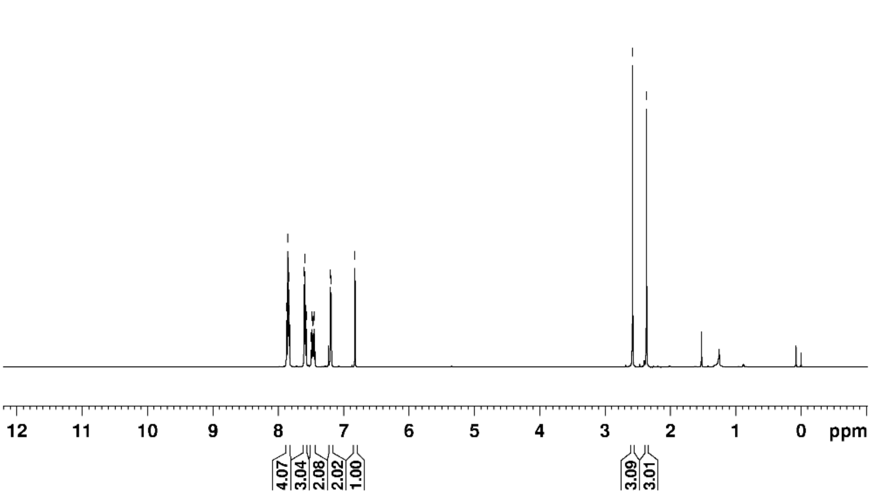
7.874
7.854
7.841
7.830
7.606
7.591
7.573
7.498
7.486
7.474
7.466
7.453
7.441
7.204
7.191
6.827
2.578
2.364
12 11 10 9 8 7 6 5 4 3 2 1 0 ppm
4.07
3.04
2.08
2.02
1.00
3.09
3.01

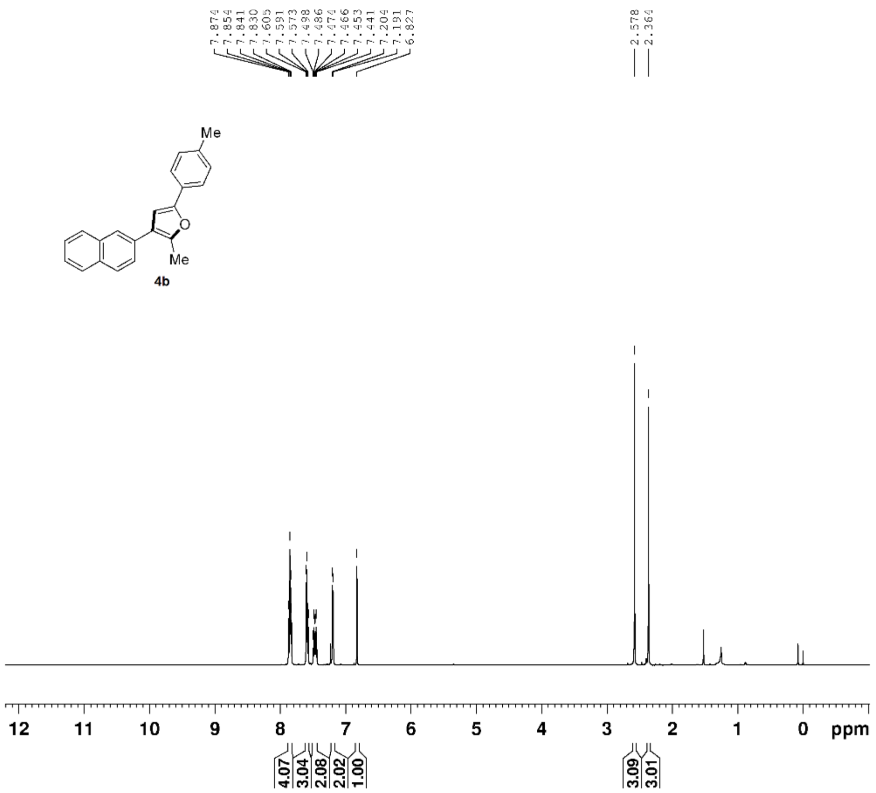

7.874
7.854
7.841
7.830
7.605
7.591
7.573
7.498
7.486
7.474
7.466
7.453
7.441
7.204
7.191
6.827
2.578
2.364
Me
O
Me
4b
12
11
10
9
8
7
6
5
4
3
2
1
0
ppm
4.07
3.04
2.08
2.02
1.00
3.09
3.01

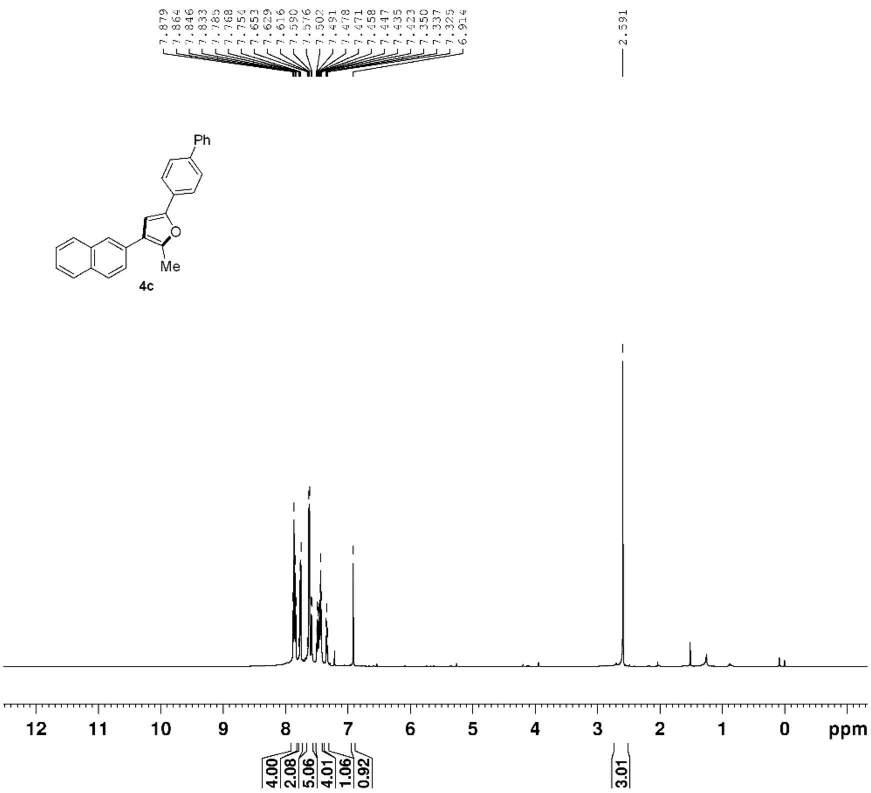
Ph
O
Me
4c
ppm

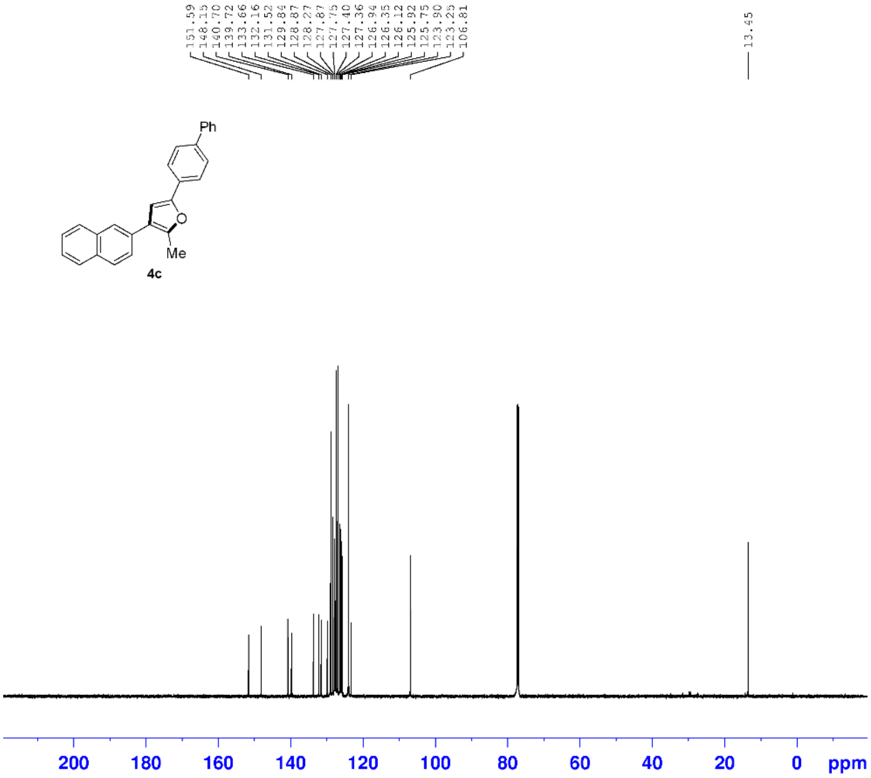
151.59
148.15
140.70
139.72
133.66
132.16
131.52
129.84
128.87
128.27
127.87
127.75
127.40
127.36
126.94
126.35
126.12
125.92
125.75
123.90
123.25
106.81
13.45
Ph
O
Me
4c
200
180
160
140
120
100
80
60
40
20
0
ppm

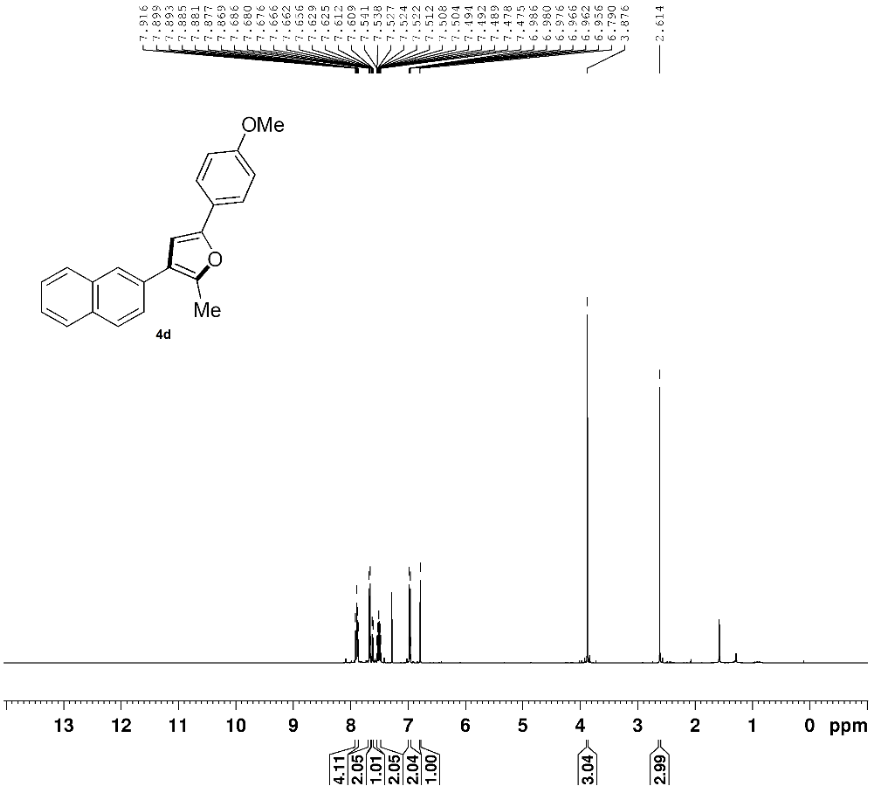

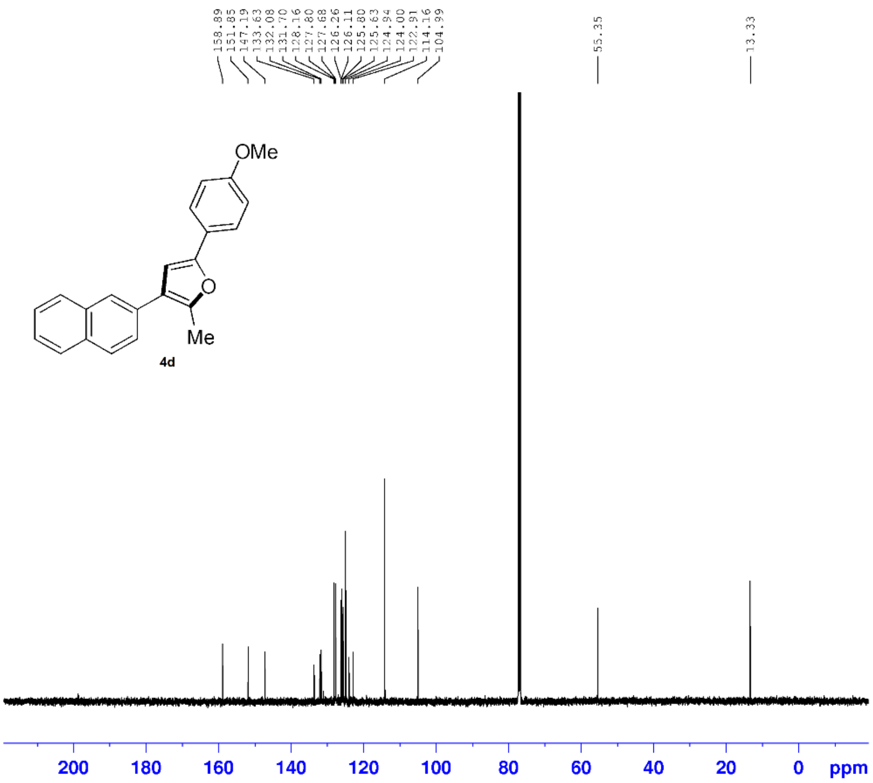

158.89
151.85
147.19
133.63
132.08
131.70
128.16
127.80
127.68
126.26
126.11
125.80
125.63
124.94
124.00
122.91
114.16
104.99
55.35
13.33
OMe
O
Me
4d
200
180
160
140
120
100
80
60
40
20
0
ppm

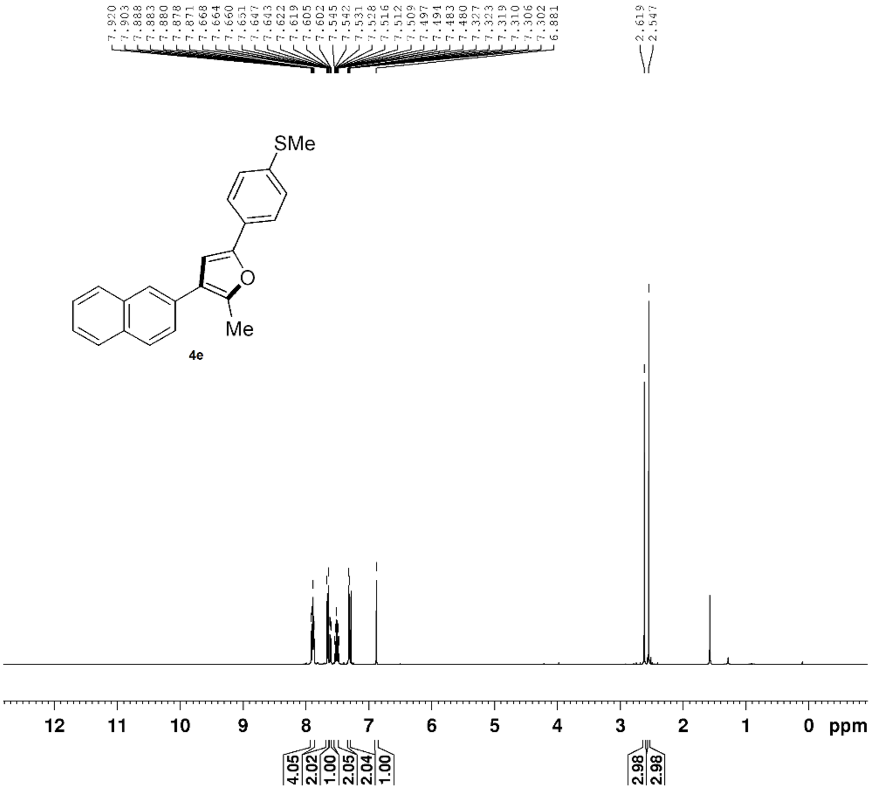

7.920
7.903
7.888
7.883
7.880
7.878
7.871
7.668
7.664
7.660
7.651
7.647
7.643
7.622
7.619
7.605
7.602
7.545
7.542
7.531
7.528
7.516
7.512
7.509
7.497
7.491
7.483
7.480
7.327
7.323
7.319
7.310
7.306
7.302
6.881
2.619
2.547
SMe
O
Me
4e
12
11
10
9
8
7
6
5
4
3
2
1
0
ppm
4.05
2.02
1.00
2.05
2.04
1.00
2.98
2.98

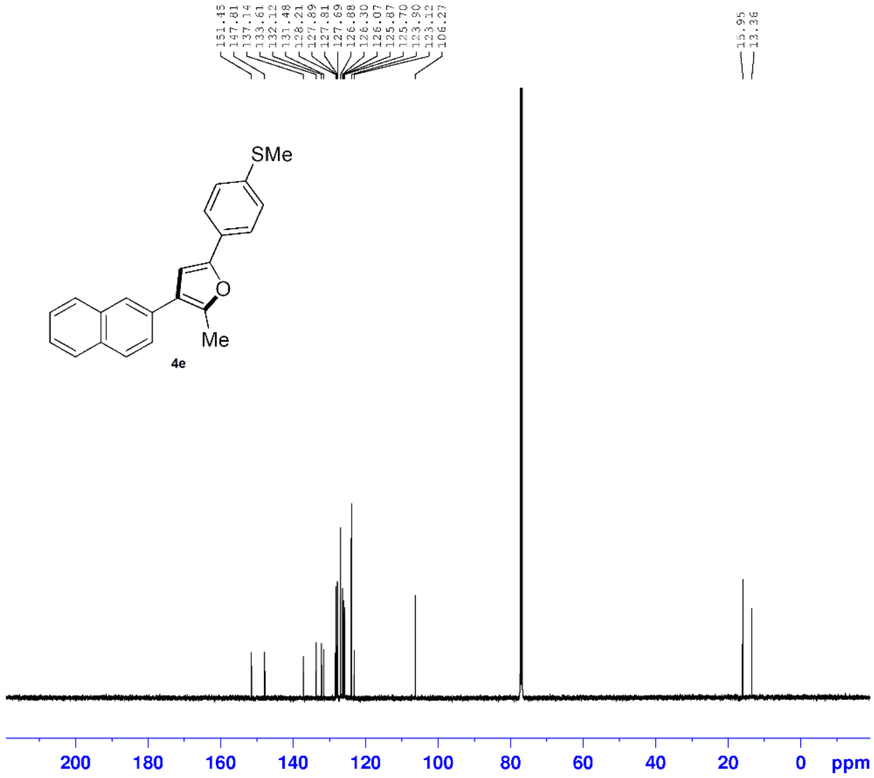
151.45
147.81
137.14
133.61
132.12
131.48
128.21
127.89
127.81
127.69
126.88
126.30
126.07
125.87
125.70
123.90
123.12
106.27
15.95
13.36
SMe
O
Me
4e
200
180
160
140
120
100
80
60
40
20
0
ppm

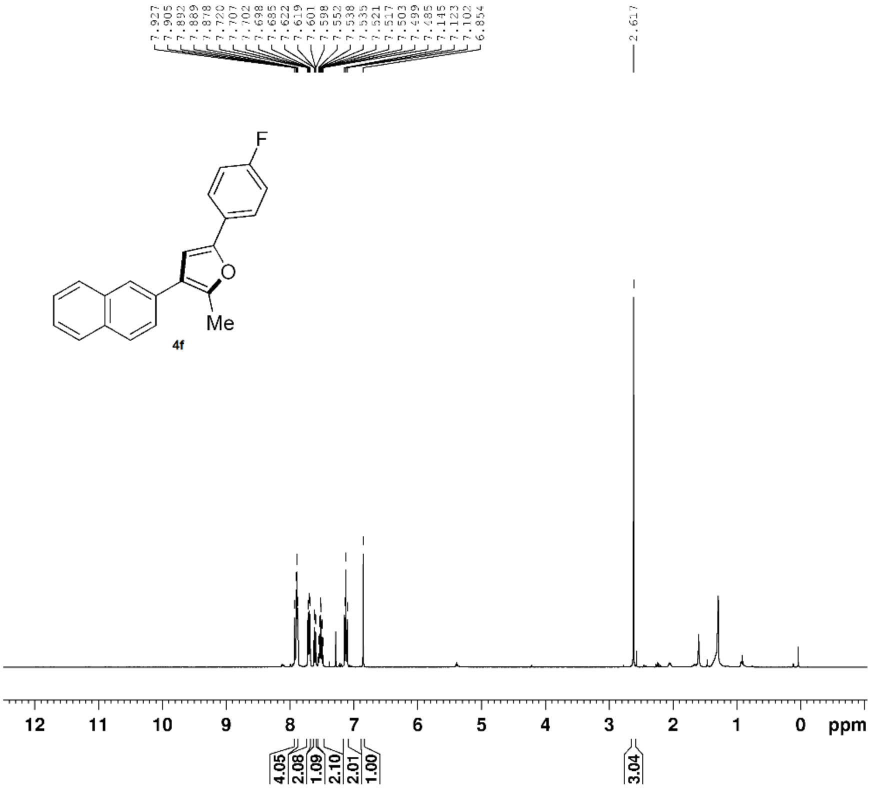

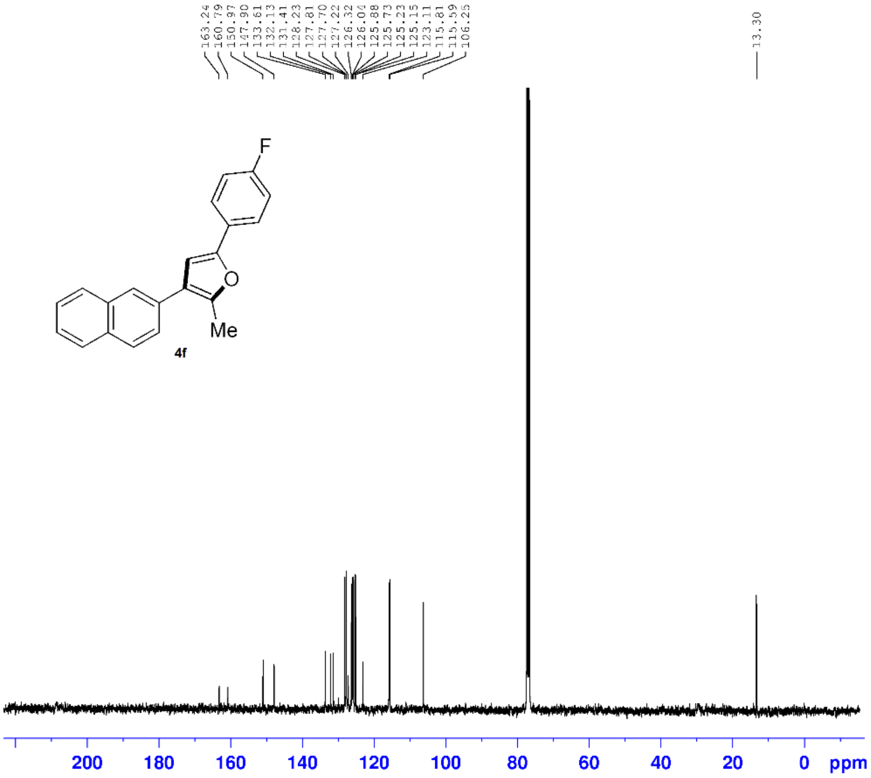
163.24
160.79
150.97
147.90
133.61
132.13
131.41
128.23
127.81
127.70
127.22
126.32
126.04
125.88
125.73
125.23
125.15
123.11
115.81
115.59
106.25
13.30
F
O
Me
4f
200
180
160
140
120
100
80
60
40
20
0
ppm

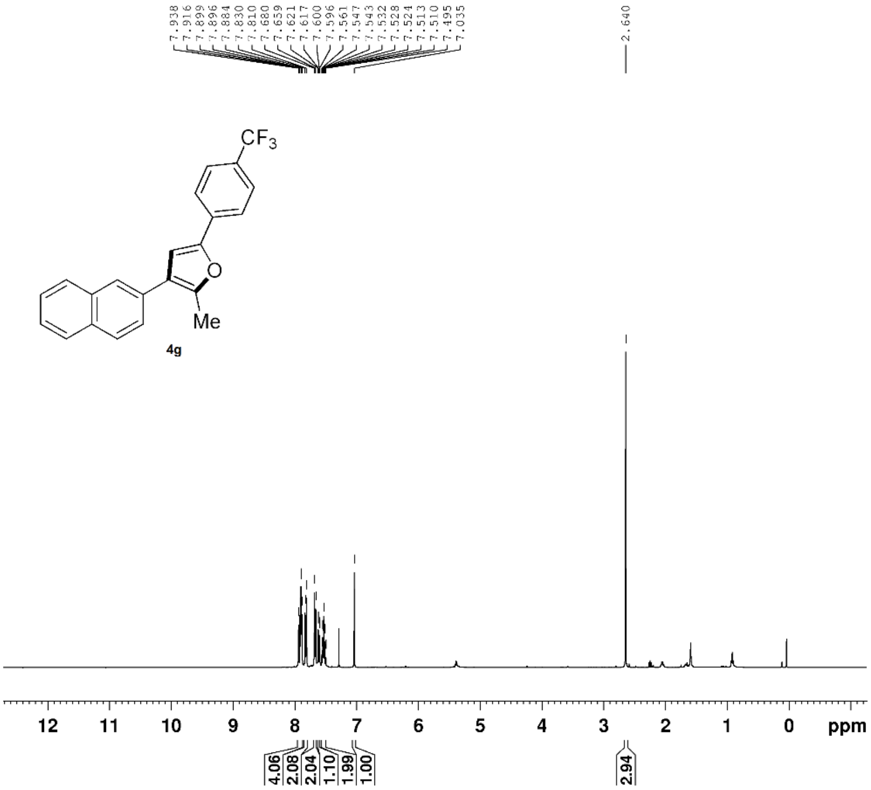

7.938
7.916
7.899
7.896
7.884
7.830
7.810
7.680
7.659
7.621
7.617
7.600
7.596
7.561
7.547
7.543
7.532
7.528
7.524
7.513
7.510
7.495
7.035
2.640
CF3
O
Me
4g
12
11
10
9
8
7
6
5
4
3
2
1
0
ppm
4.06
2.08
2.04
1.10
1.99
1.00
2.94

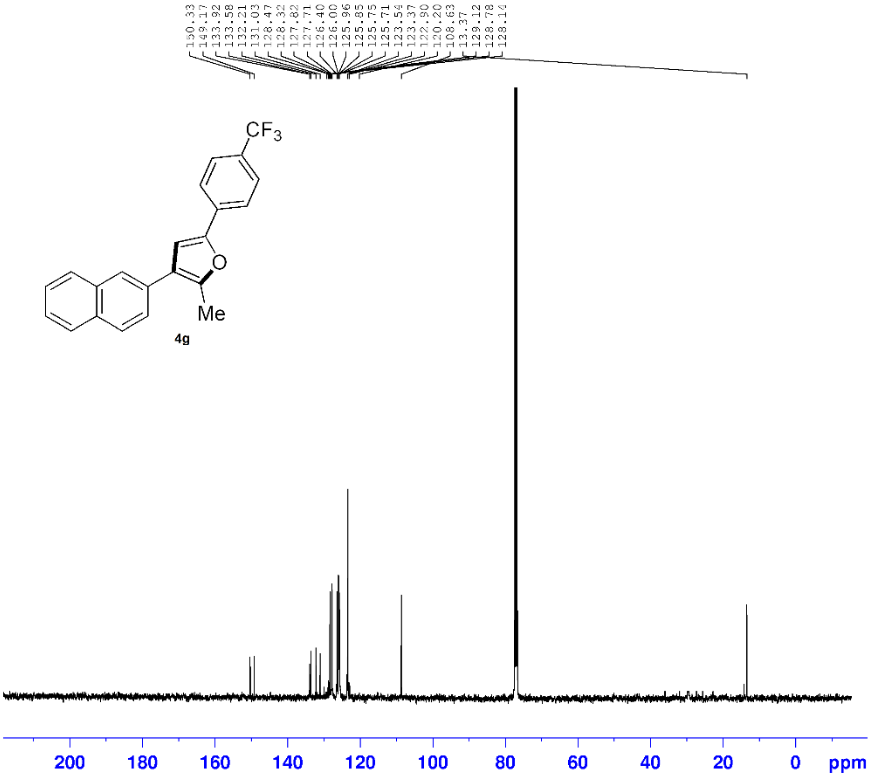

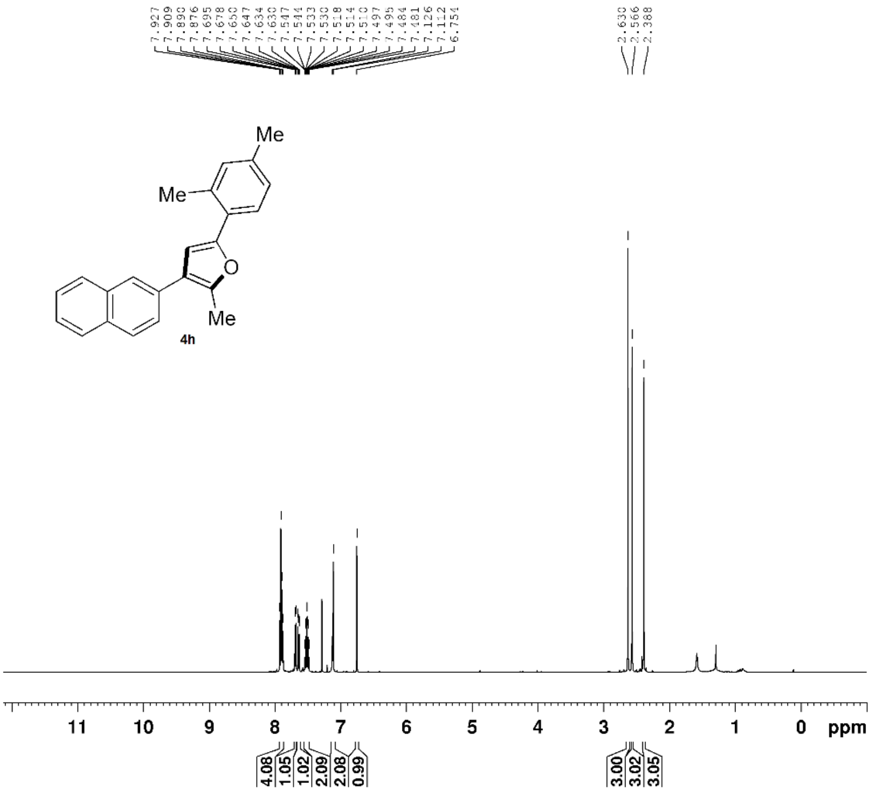

7.927
7.909
7.890
7.876
7.695
7.678
7.650
7.647
7.634
7.630
7.547
7.544
7.533
7.530
7.518
7.514
7.510
7.497
7.495
7.484
7.481
7.126
7.112
6.754
2.630
2.566
2.388
Me
Me
O
Me
4h
11
10
9
8
7
6
5
4
3
2
1
0
ppm
4.08
1.05
1.02
2.09
2.08
0.99
3.00
3.02
3.05

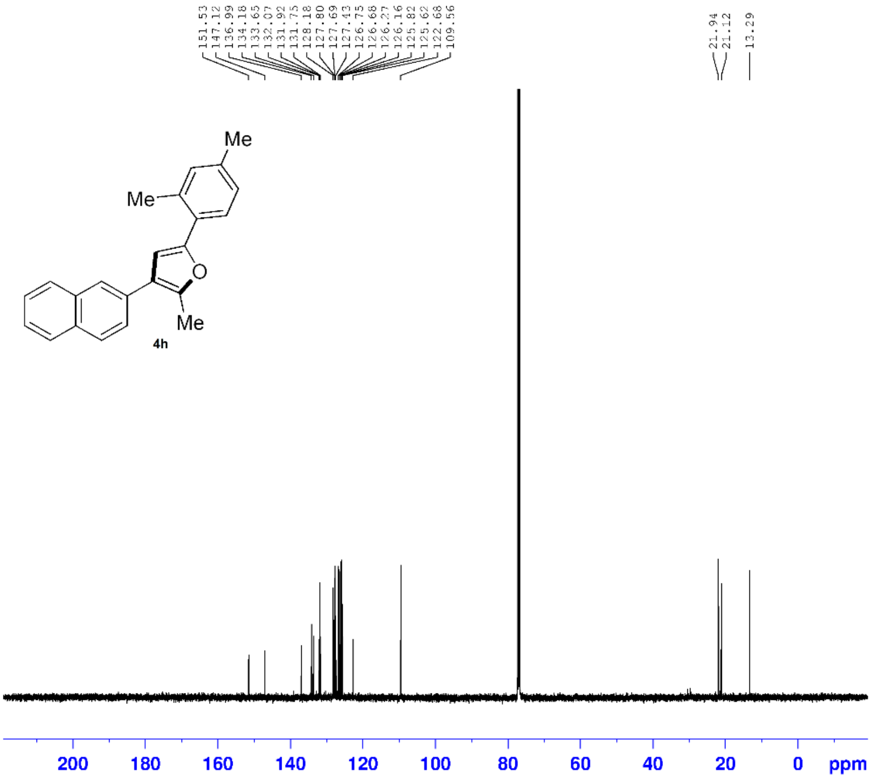

151.53
147.12
136.99
134.18
133.65
132.07
131.92
131.75
128.18
127.80
127.69
127.43
126.75
126.68
126.27
126.16
125.82
125.62
122.68
109.56
21.94
21.12
13.29
Me
Me
O
Me
4h
200
180
160
140
120
100
80
60
40
20
0
ppm

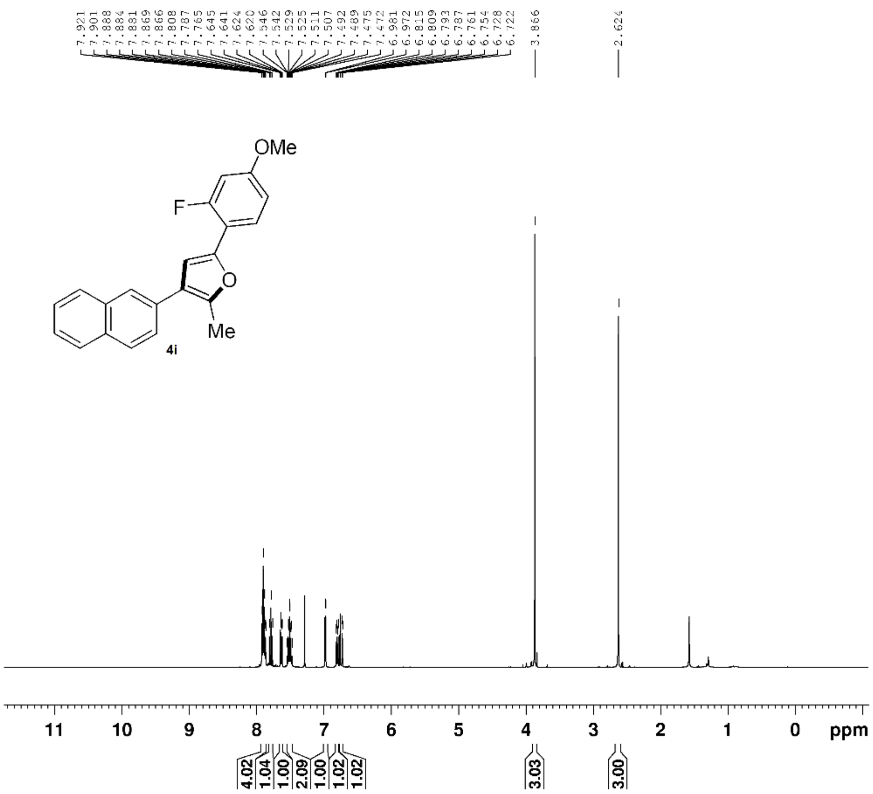

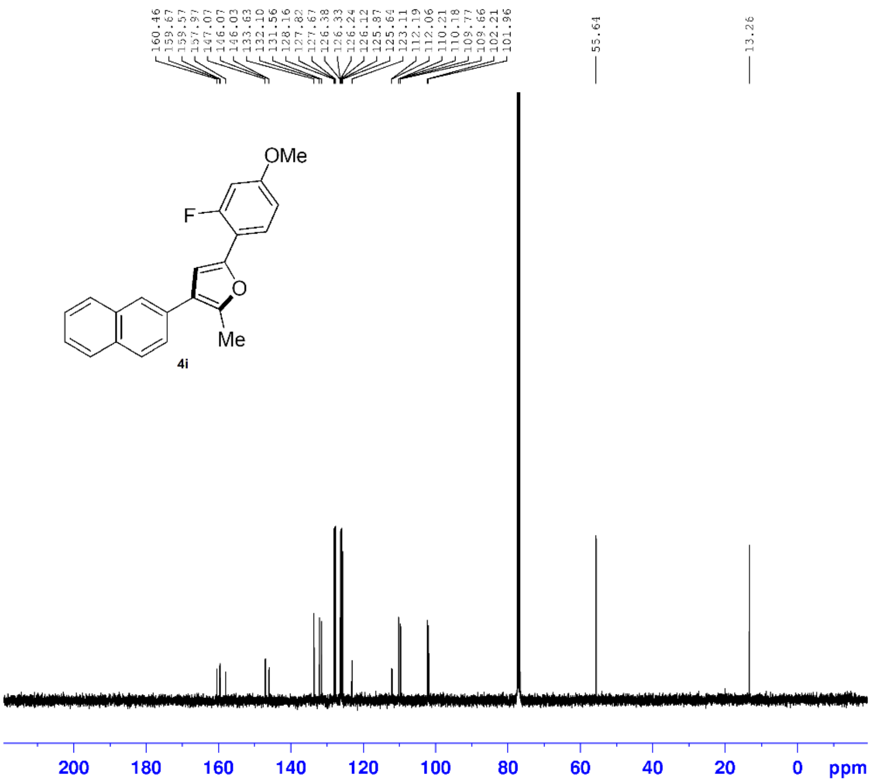

160.46
159.67
159.57
157.97
147.07
146.07
146.03
133.63
132.10
131.56
128.16
127.82
127.67
126.38
126.33
126.24
126.12
125.87
125.64
123.11
112.19
112.06
110.21
110.18
109.77
109.66
102.21
101.96
55.64
13.26
OMe
F
O
Me
4i
200
180
160
140
120
100
80
60
40
20
0
ppm

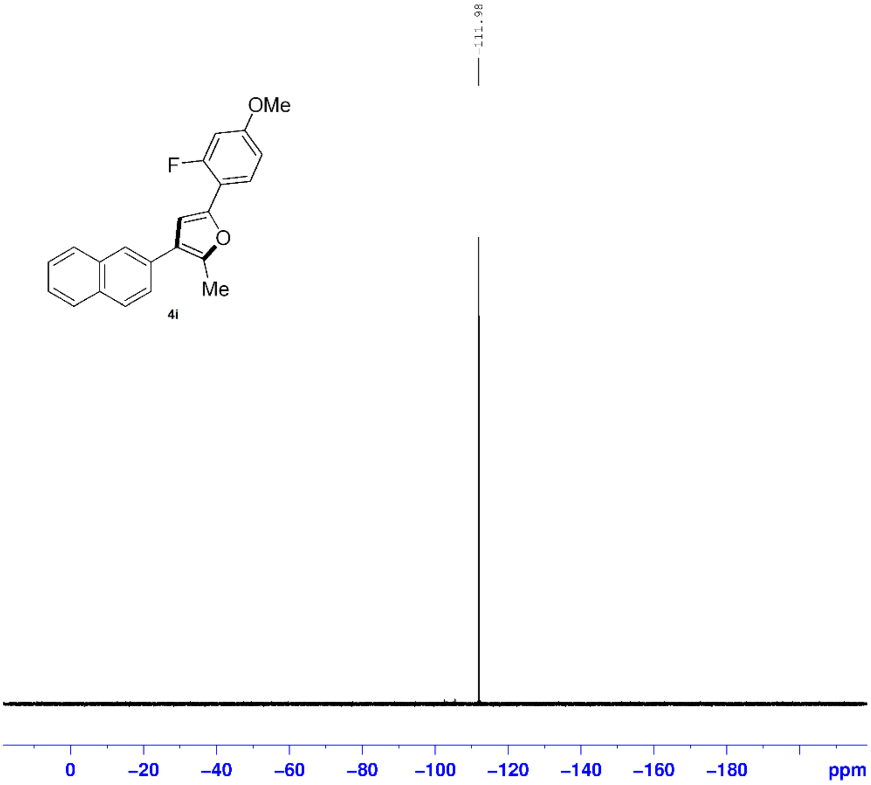
OMe
F
O
Me
4i
-111.98
0
–20
–40
–60
–80
–100
–120
–140
–160
–180
ppm

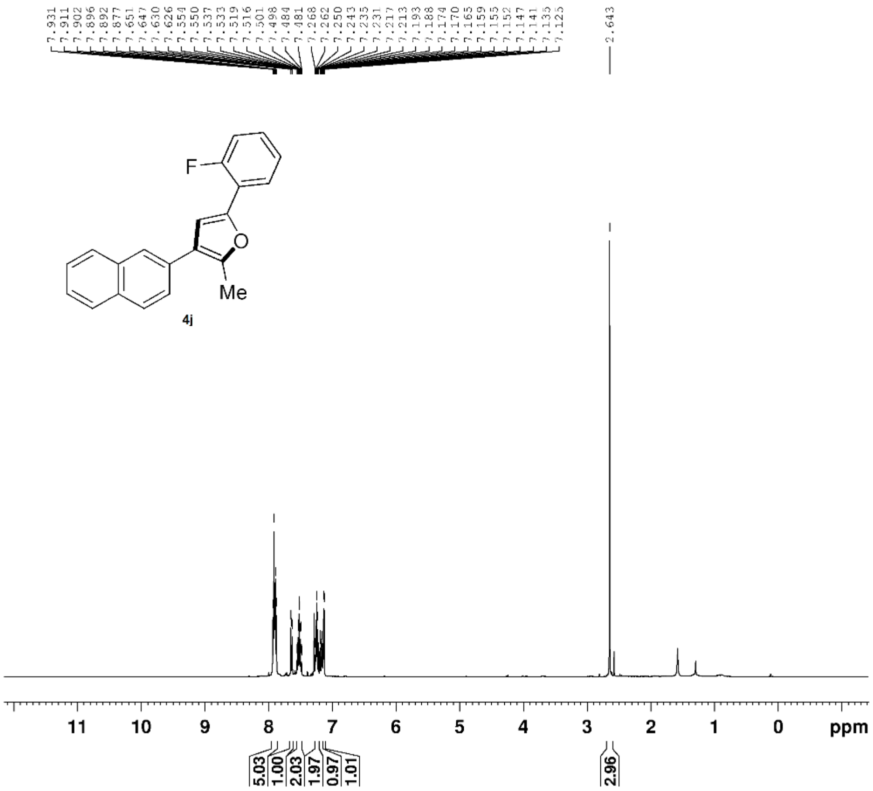

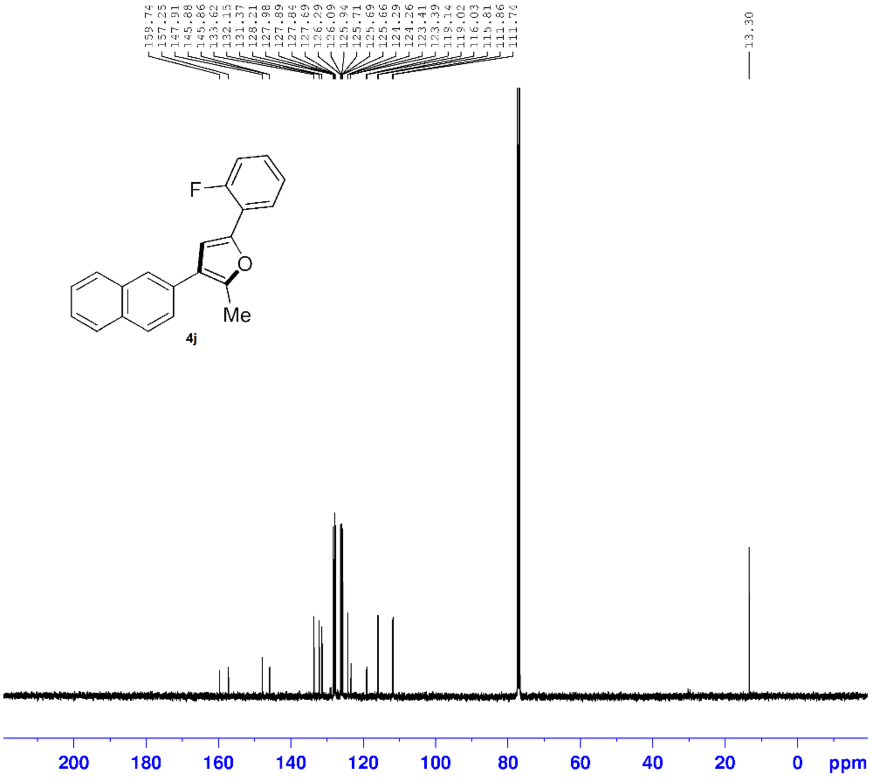
F
O
Me
4j
159.74
157.25
147.91
145.88
145.86
133.62
132.15
131.37
128.21
127.98
127.89
127.84
127.69
126.29
126.09
125.94
125.71
125.69
125.66
124.29
124.26
123.41
123.39
119.14
119.02
116.03
115.81
111.86
111.74
13.30
200
180
160
140
120
100
80
60
40
20
0
ppm

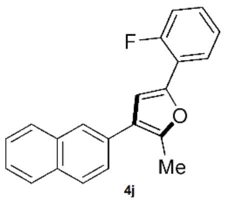

4j

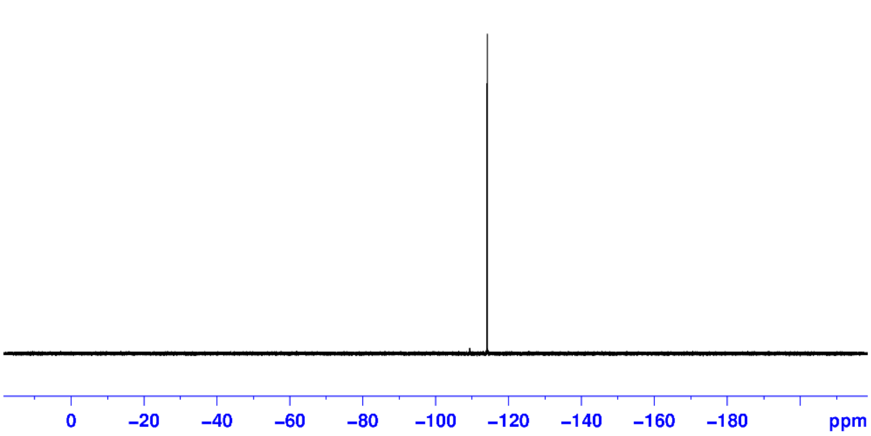

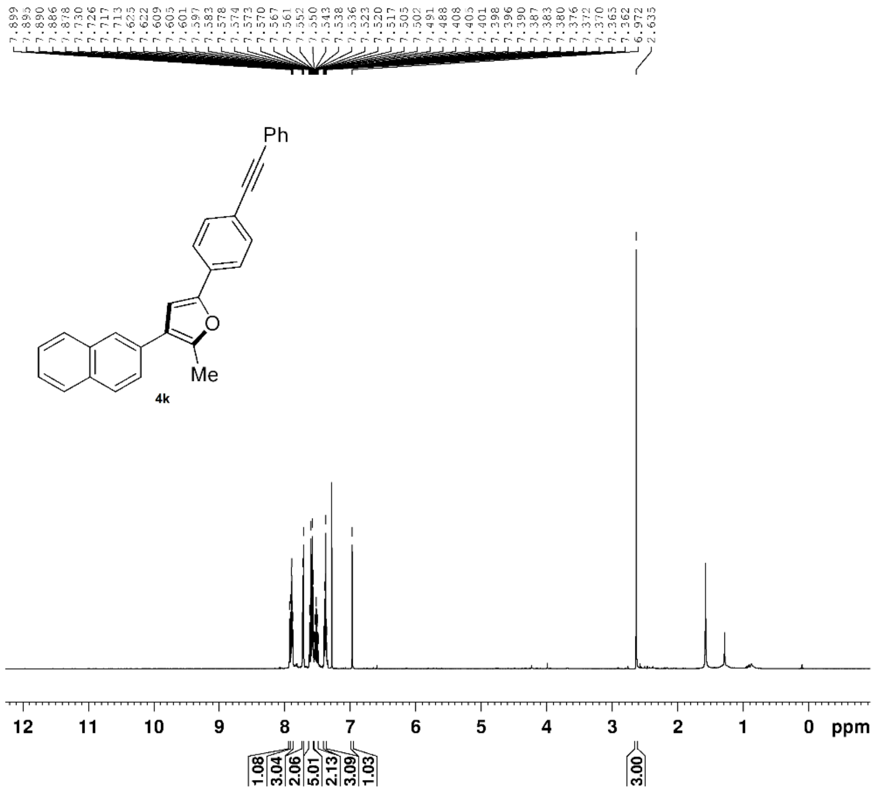
Ph
O
Me
4k
12
11
10
9
8
7
6
5
4
3
2
1
0
ppm
1.08
3.04
2.06
5.01
2.13
3.09
1.03
3.00

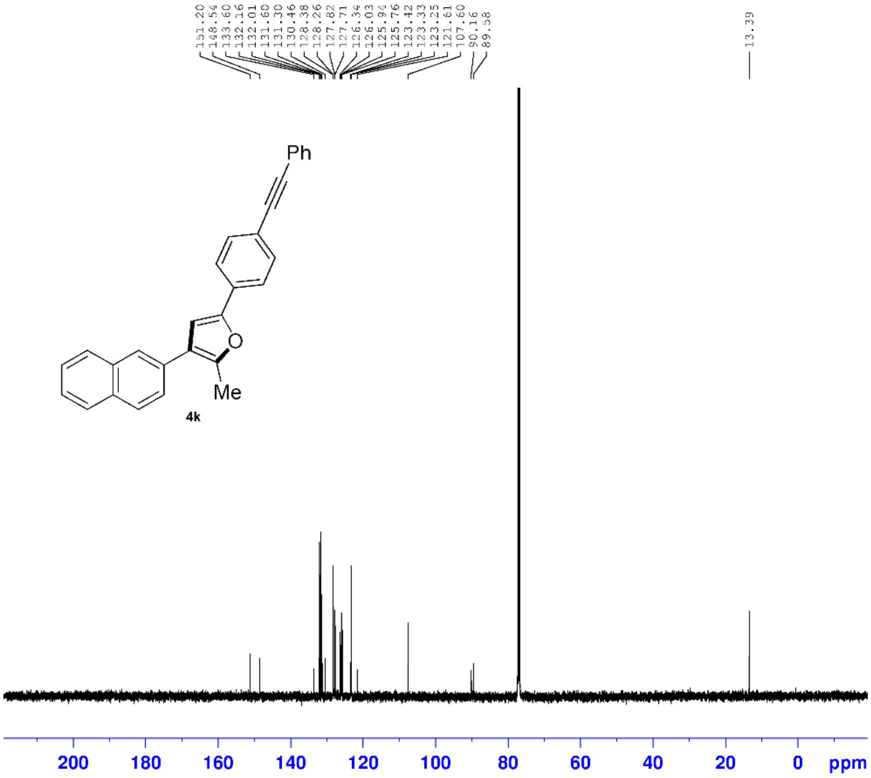

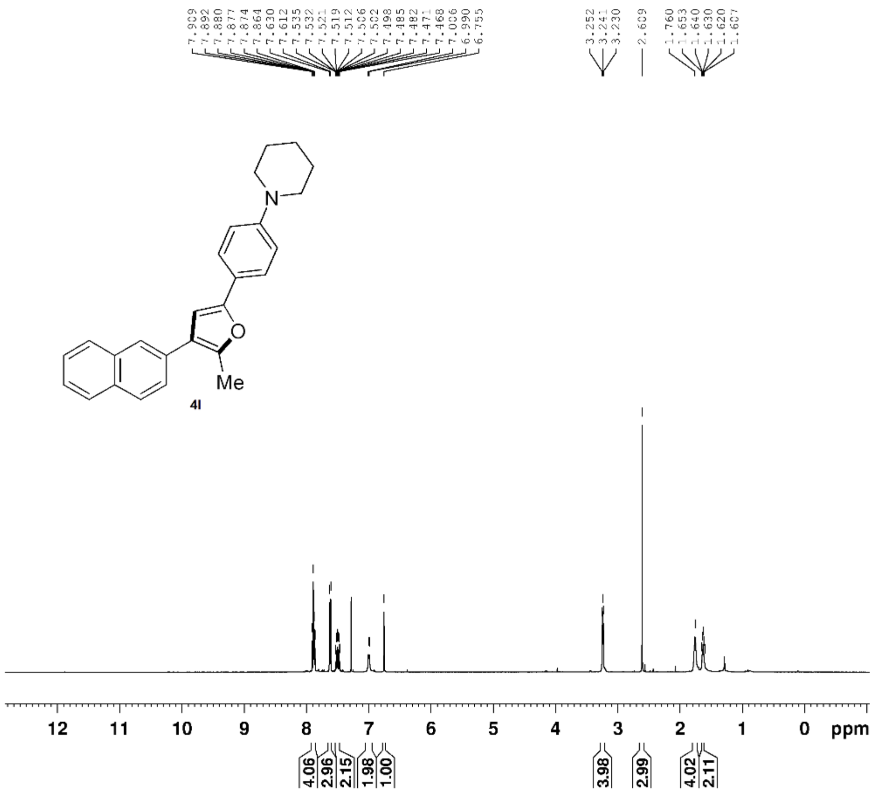

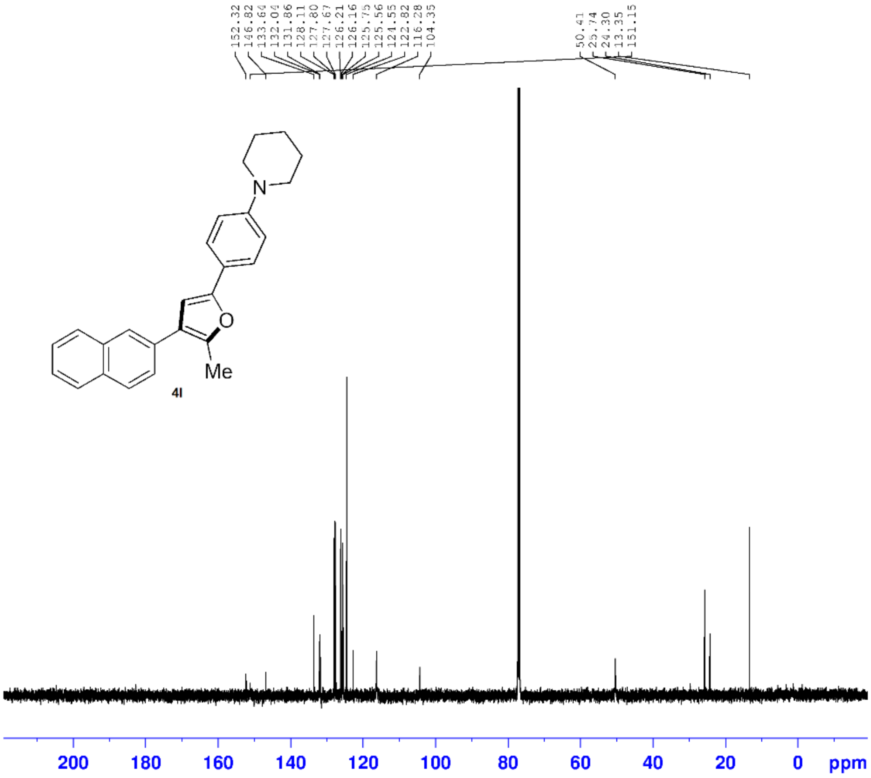
152.32
146.82
133.64
132.04
131.86
128.11
127.80
127.67
126.21
126.16
125.75
125.56
121.55
122.82
116.28
104.35
50.41
25.74
24.30
13.35
151.15
N
O
Me
4l
200
180
160
140
120
100
80
60
40
20
0
ppm

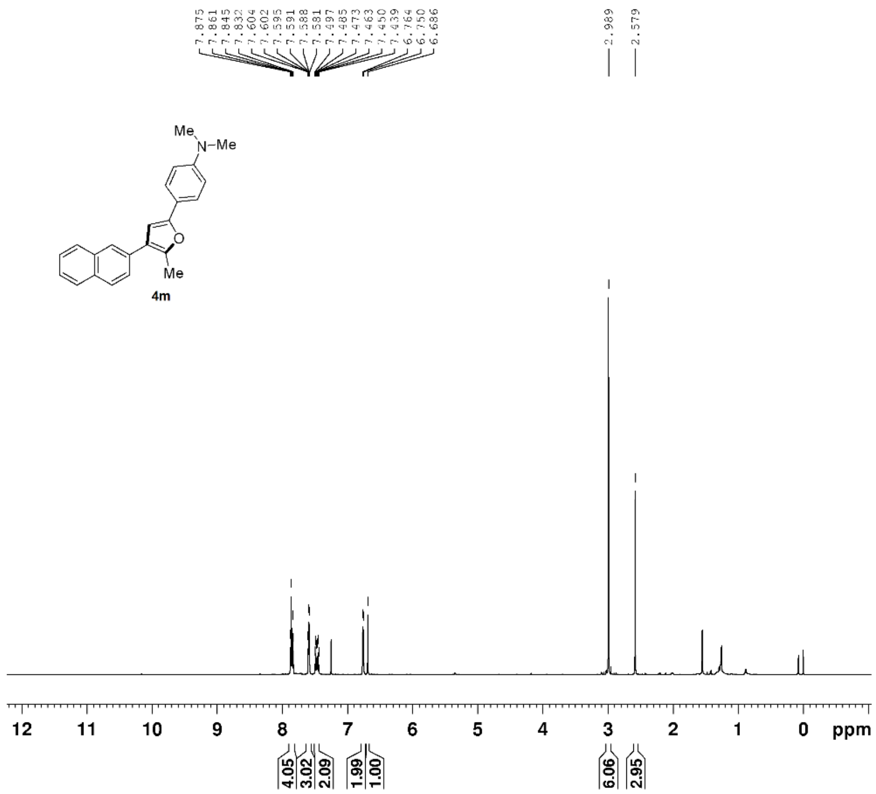

7.875
7.861
7.845
7.832
7.604
7.602
7.595
7.591
7.588
7.581
7.497
7.485
7.473
7.463
7.450
7.439
6.764
6.750
6.686
2.989
2.579
Me
N–Me
O
Me
4m
12
11
10
9
8
7
6
5
4
3
2
1
0
ppm
4.05
3.02
2.09
1.99
1.00
6.06
2.95

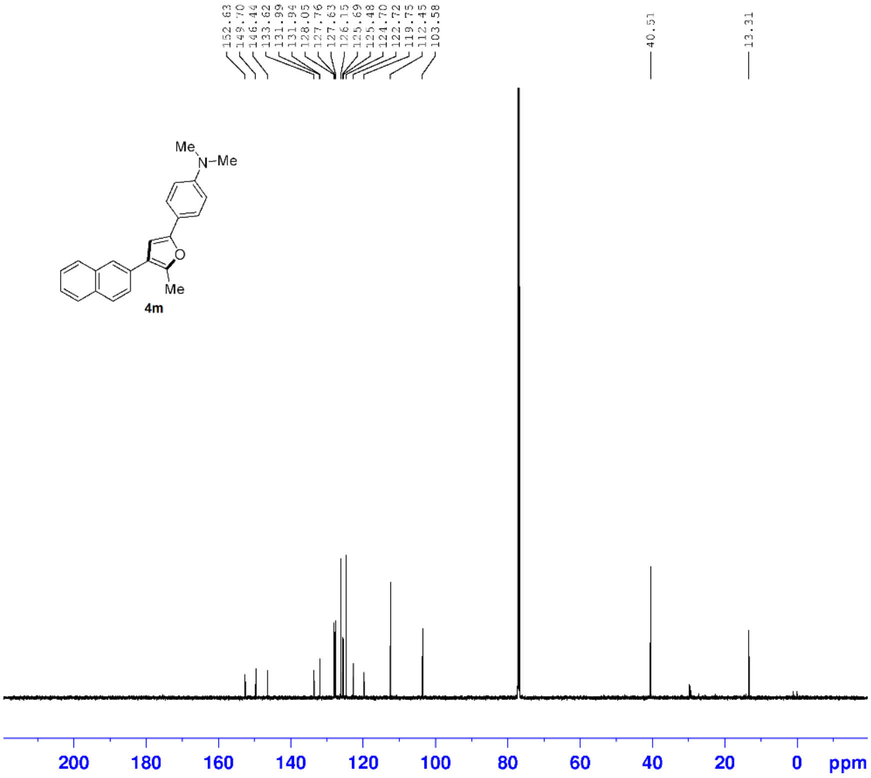
152.63
149.70
146.44
133.62
131.99
131.94
128.05
127.76
127.63
126.15
125.69
125.48
124.70
122.72
119.75
112.45
103.58
40.51
13.31
Me
N
Me
O
Me
4m
200
180
160
140
120
100
80
60
40
20
0
ppm

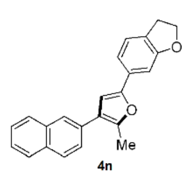
O
Me

4n

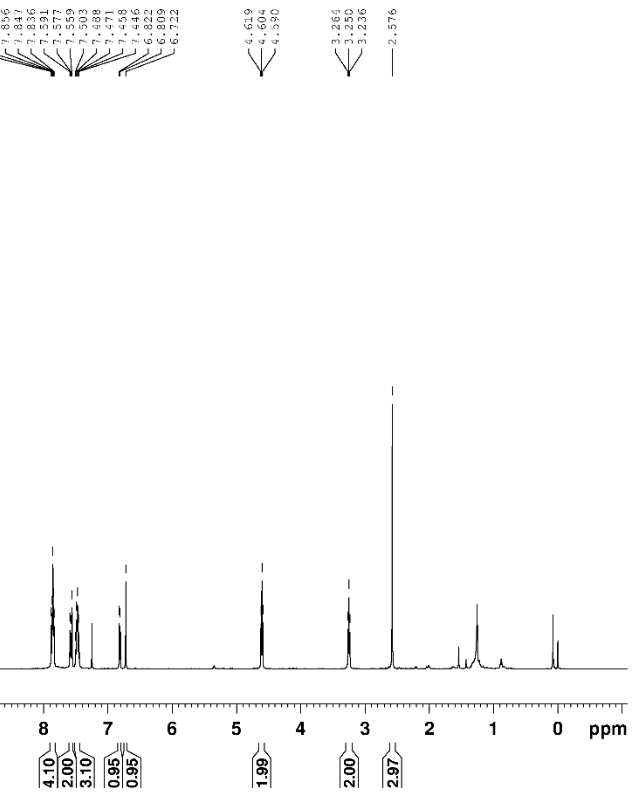
7.881
7.866
7.856
7.847
7.836
7.591
7.577
7.559
7.503
7.488
7.471
7.458
7.446
6.822
6.809
6.722
4.619
4.604
4.590
3.264
3.250
3.236
2.576
12
11
10
9
8
7
6
5
4
3
2
1
0
ppm
4.10
2.00
3.10
0.95
0.95
1.99
2.00
2.97

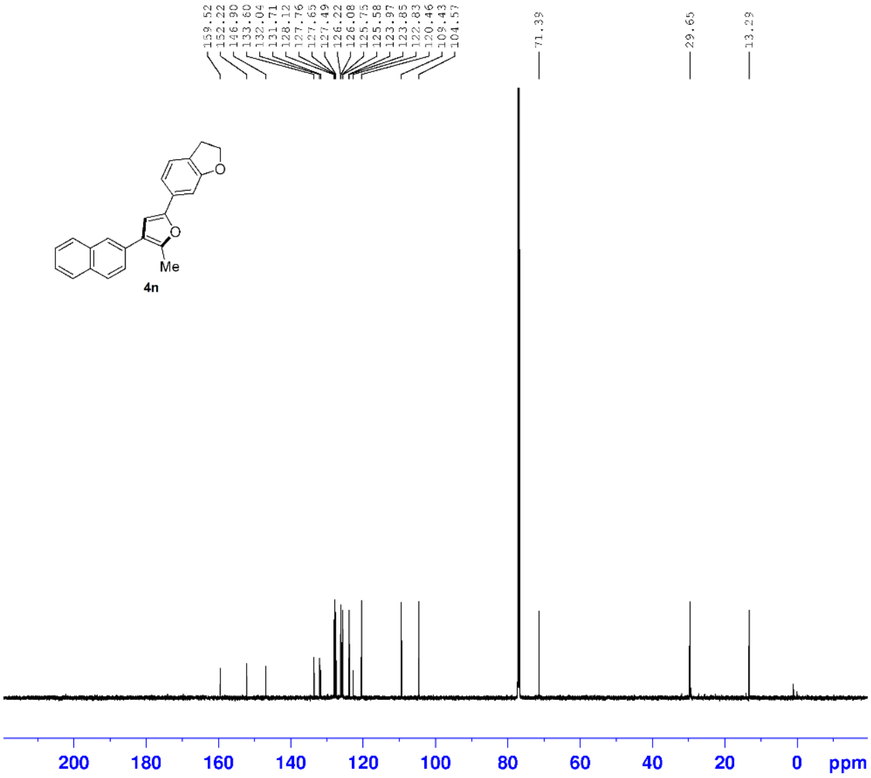
159.52
152.22
146.90
133.60
132.04
131.71
128.12
127.76
127.65
127.49
126.22
126.08
125.75
125.58
123.97
123.85
122.83
120.46
109.43
104.57
71.39
29.65
13.29
O
O
Me
4n
200
180
160
140
120
100
80
60
40
20
0
ppm

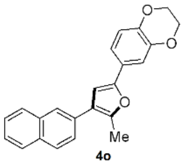
4o

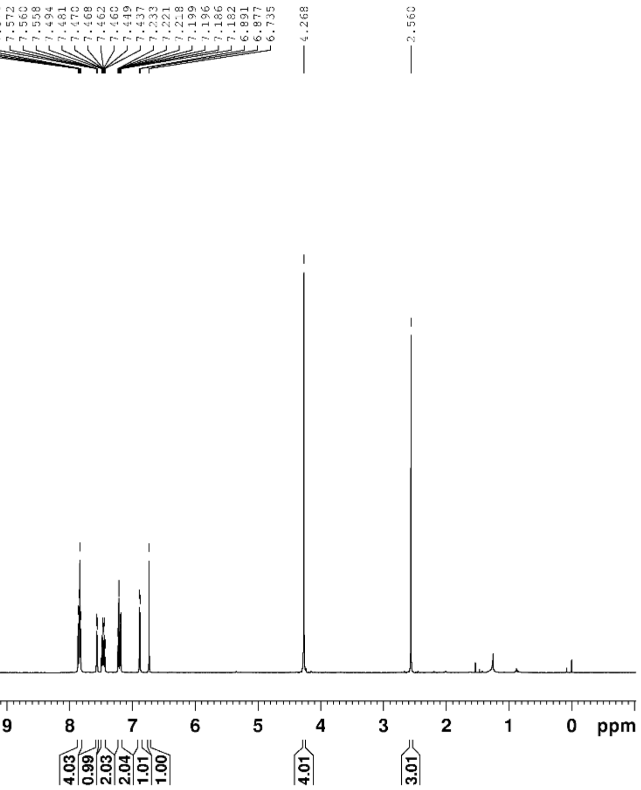

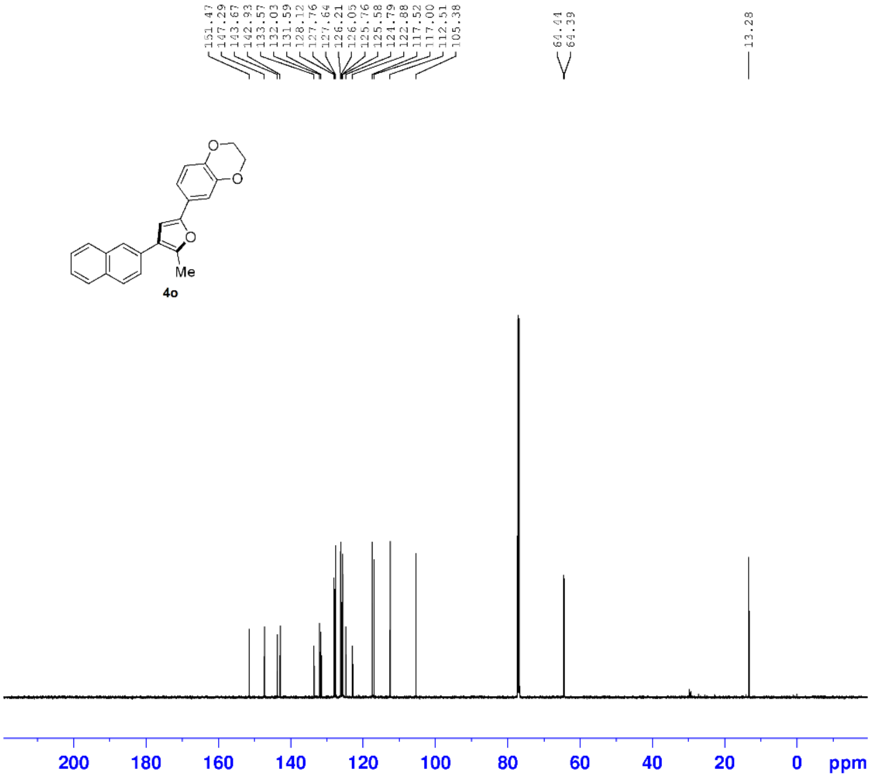

151.47
147.29
143.67
142.93
133.57
132.03
131.59
128.12
127.76
127.64
126.21
126.05
125.76
125.58
121.79
122.88
117.52
117.00
112.51
105.38
64.44
64.39
13.28
O
O
O
Me
4o
200
180
160
140
120
100
80
60
40
20
0
ppm

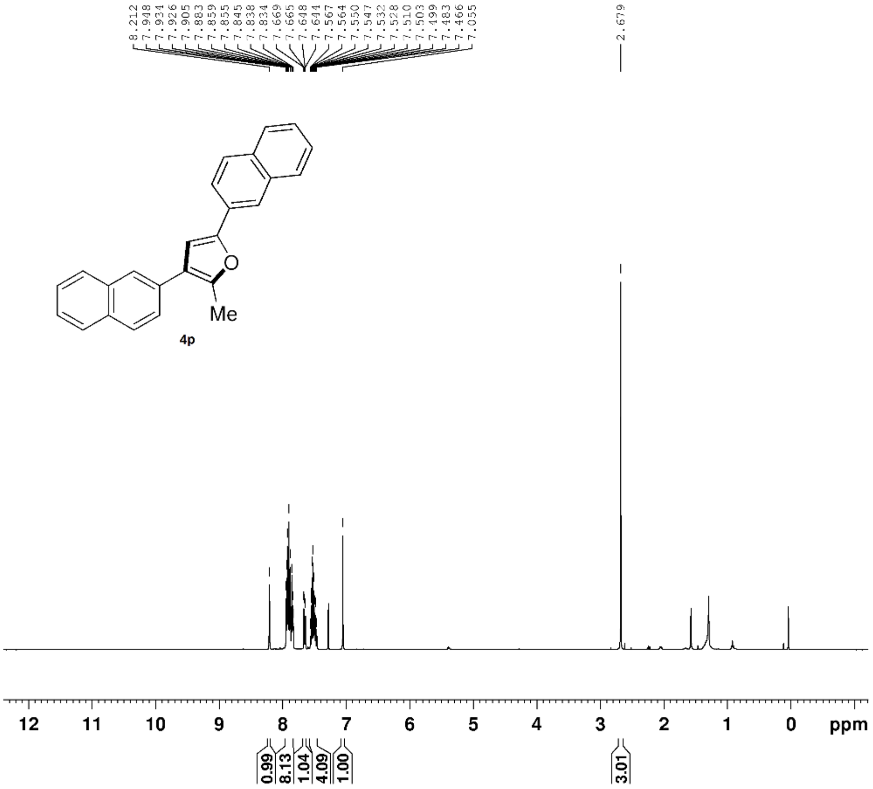
8.212
7.948
7.934
7.926
7.905
7.883
7.859
7.855
7.845
7.838
7.834
7.669
7.665
7.648
7.644
7.567
7.564
7.550
7.547
7.532
7.528
7.510
7.503
7.499
7.483
7.466
7.055
2.679
O
Me
4p
12 11 10 9 8 7 6 5 4 3 2 1 0 ppm
0.99
8.13
1.04
4.09
1.00
3.01

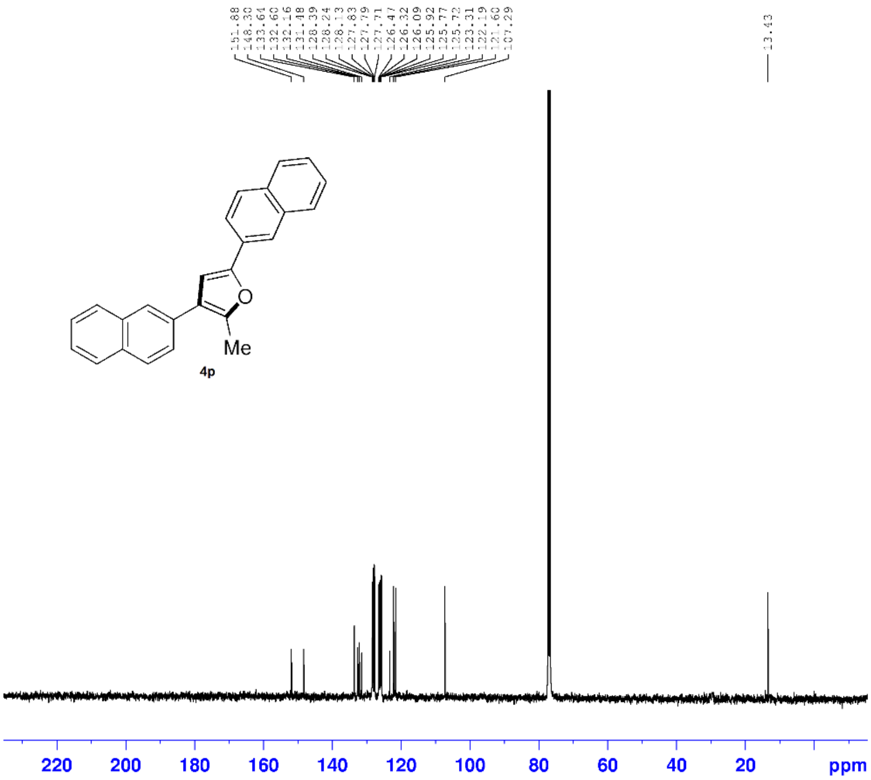
151.88
148.30
133.64
132.60
132.16
131.48
128.39
128.24
128.13
127.83
127.79
127.71
126.47
126.32
126.09
125.92
125.77
125.72
123.31
122.19
121.60
107.29
13.43
O
Me
4p
220
200
180
160
140
120
100
80
60
40
20
ppm

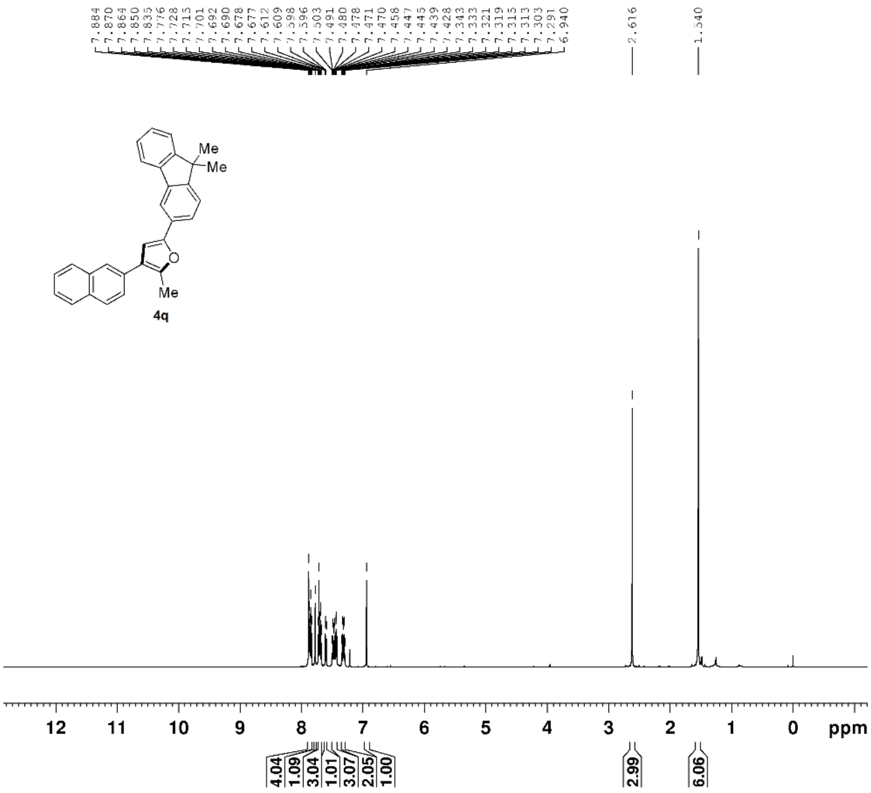

7.884
7.870
7.864
7.850
7.835
7.776
7.728
7.715
7.701
7.692
7.690
7.678
7.677
7.612
7.609
7.598
7.596
7.503
7.491
7.480
7.478
7.471
7.470
7.458
7.447
7.445
7.439
7.428
7.343
7.333
7.321
7.319
7.315
7.313
7.303
7.291
6.940
2.616
1.540
Me
Me
O
Me
4q
12
11
10
9
8
7
6
5
4
3
2
1
0
ppm
4.04
1.09
3.04
1.01
3.07
2.05
1.00
2.99
6.06

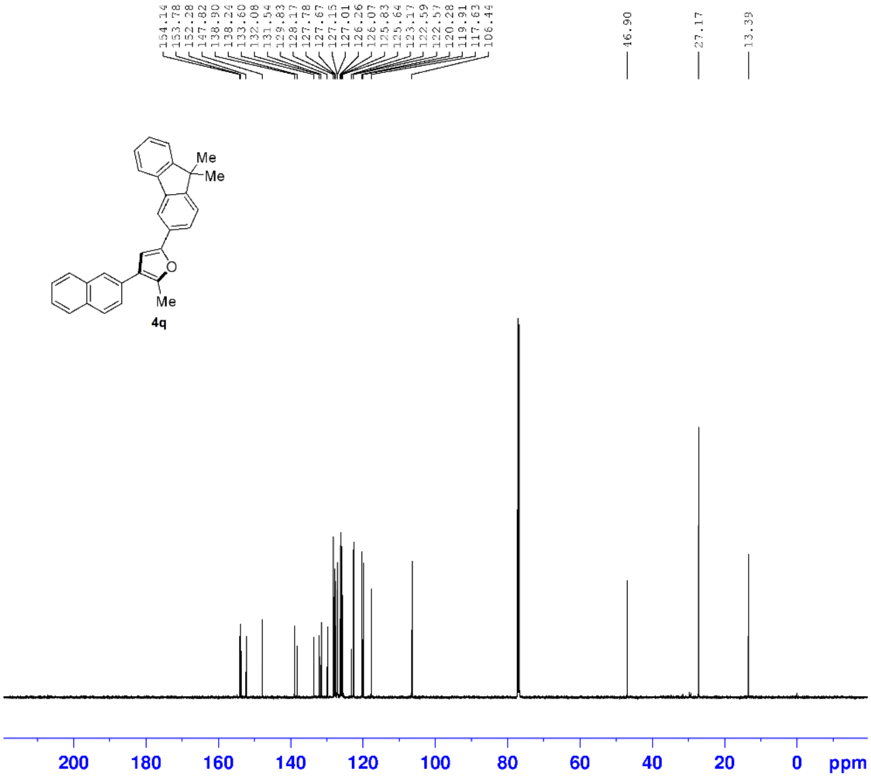
154.14
153.78
152.28
147.82
138.90
138.24
133.60
132.08
131.54
129.83
128.17
127.78
127.67
127.15
127.01
126.26
126.07
125.83
125.64
123.17
122.59
122.57
120.28
119.91
117.63
106.44
46.90
27.17
13.39
Me
Me
O
Me
4q
200
180
160
140
120
100
80
60
40
20
0
ppm

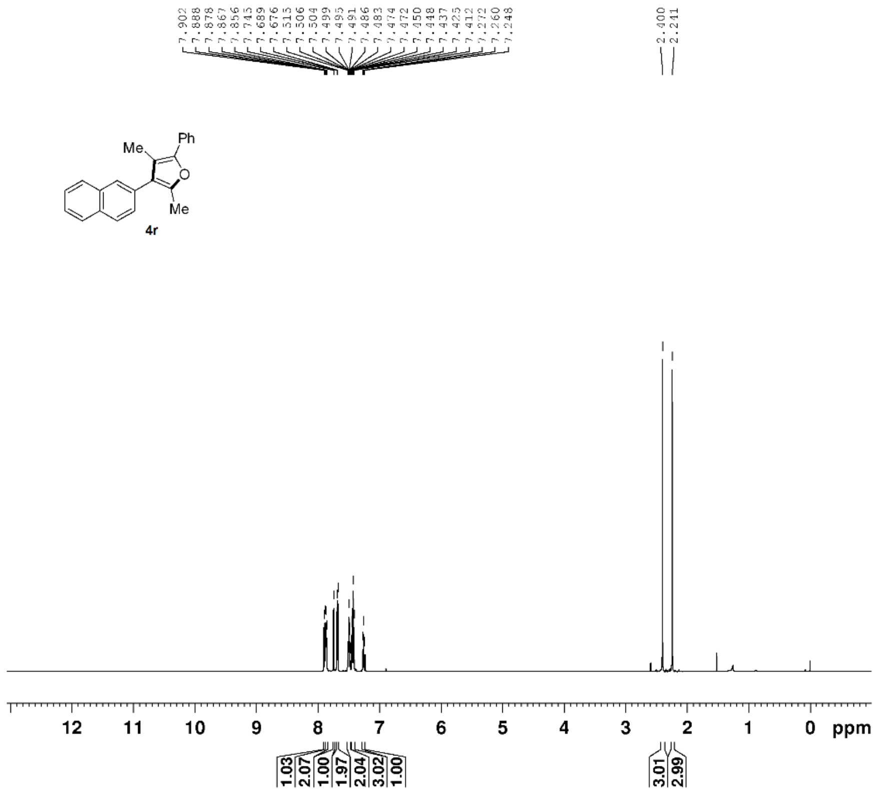

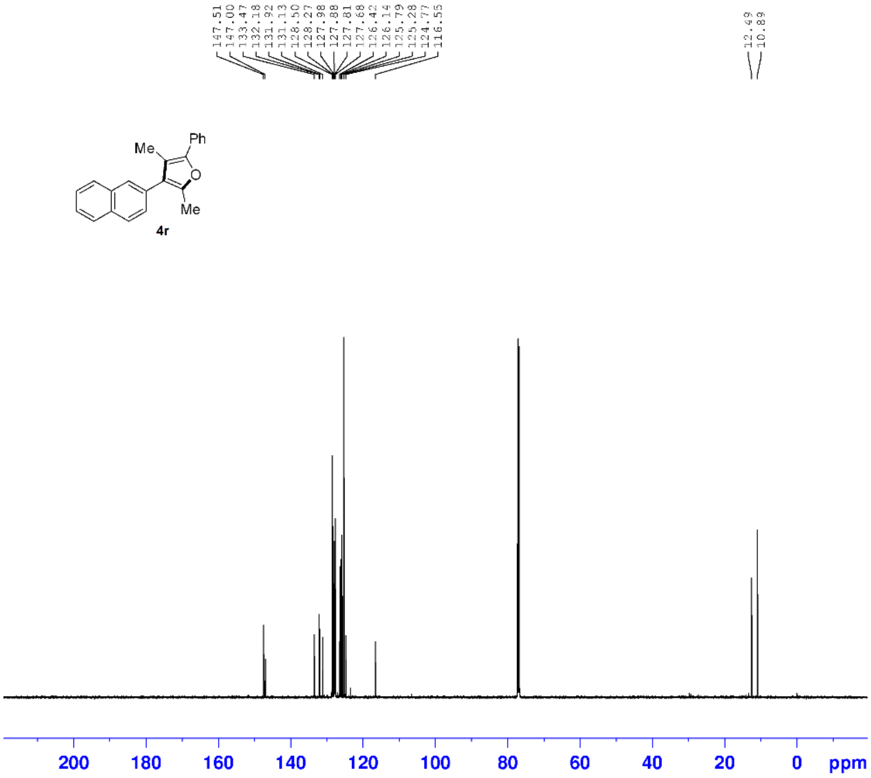
147.51
147.00
133.47
132.18
131.92
131.13
128.50
128.27
127.98
127.88
127.81
127.68
126.42
126.14
125.79
125.28
124.77
116.55
12.49
10.89
Me
Ph
O
Me
4r
200
180
160
140
120
100
80
60
40
20
0
ppm

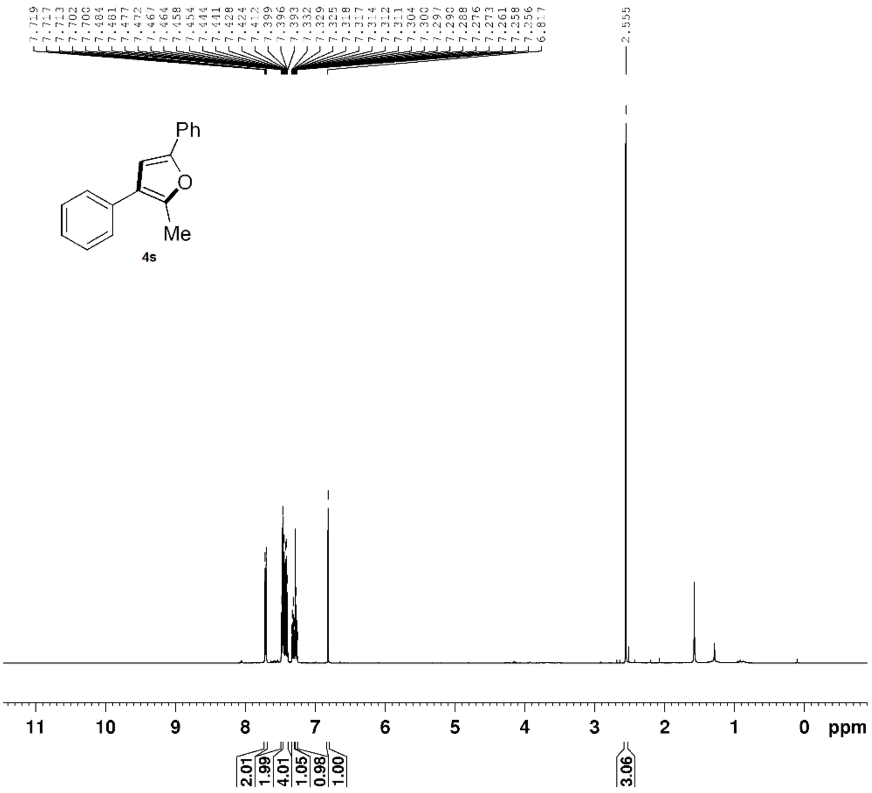

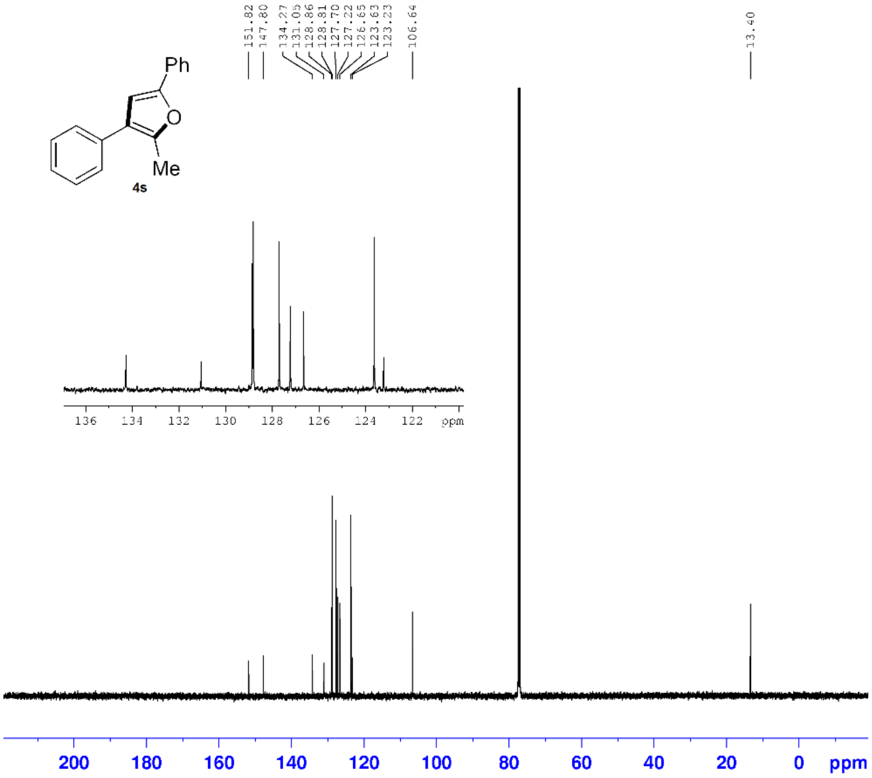
151.82
147.80
134.27
131.05
128.86
128.81
127.70
127.22
126.65
123.63
123.23
106.64
13.40
Ph
O
Me
4s
136 134 132 130 128 126 124 122 ppm
200 180 160 140 120 100 80 60 40 20 0 ppm

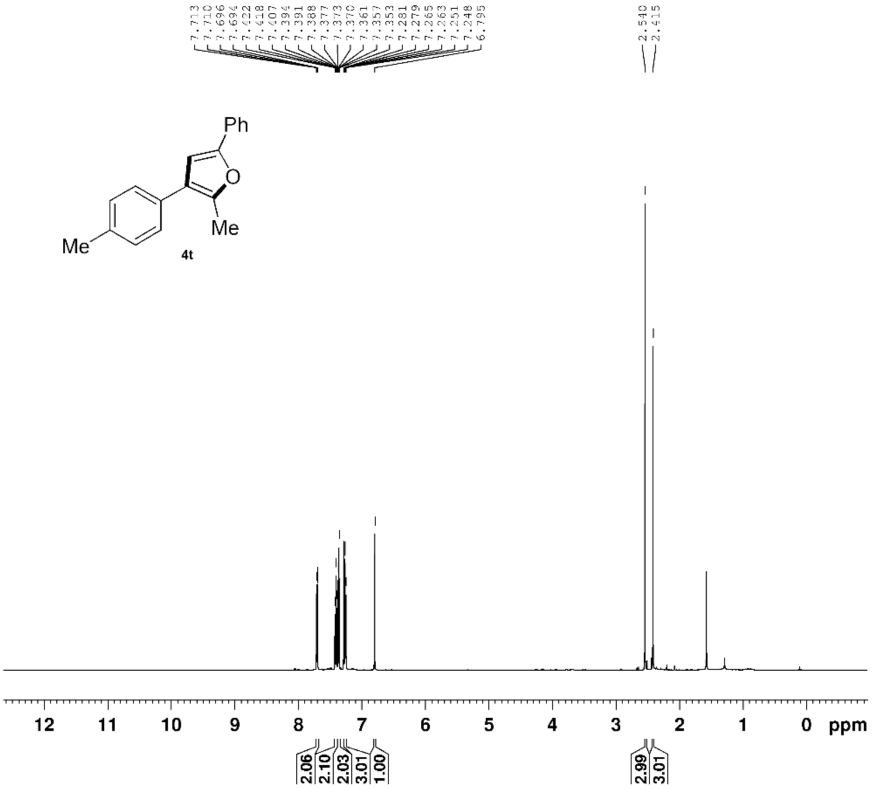
7.713
7.710
7.696
7.694
7.422
7.418
7.407
7.394
7.391
7.388
7.377
7.373
7.370
7.361
7.357
7.353
7.281
7.279
7.265
7.263
7.251
7.248
6.795
2.540
2.415
Ph
O
Me
Me
4t
12
11
10
9
8
7
6
5
4
3
2
1
0
ppm
2.06
2.10
2.03
3.01
1.00
2.99
3.01

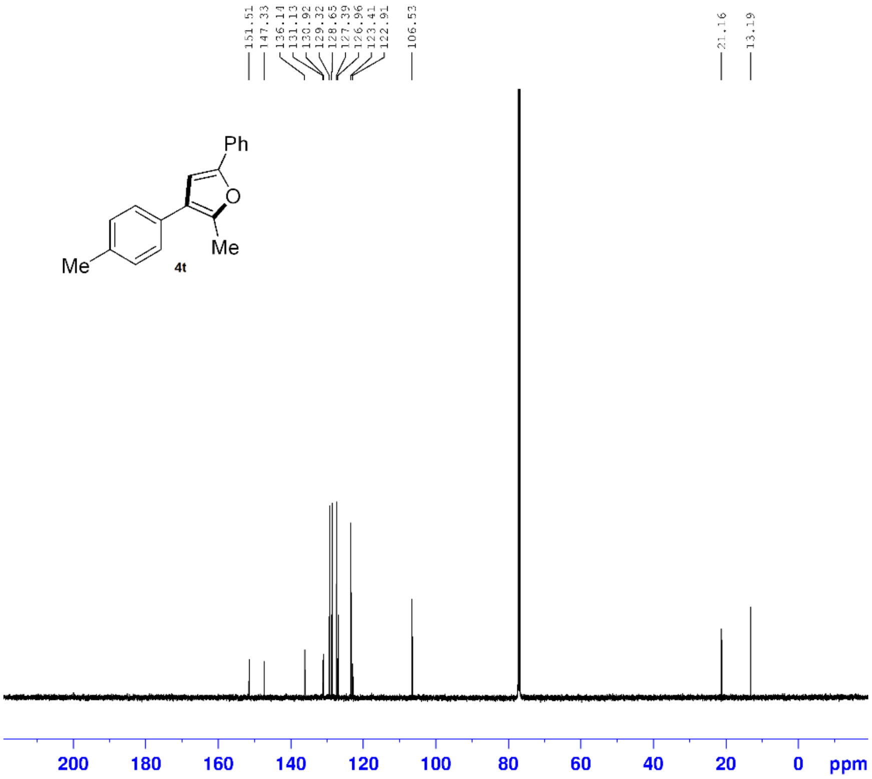
151.51
147.33
136.14
131.13
130.92
129.32
128.65
127.39
126.96
123.41
122.91
106.53
21.16
13.19
Ph
O
Me
Me
4t
200
180
160
140
120
100
80
60
40
20
0
ppm

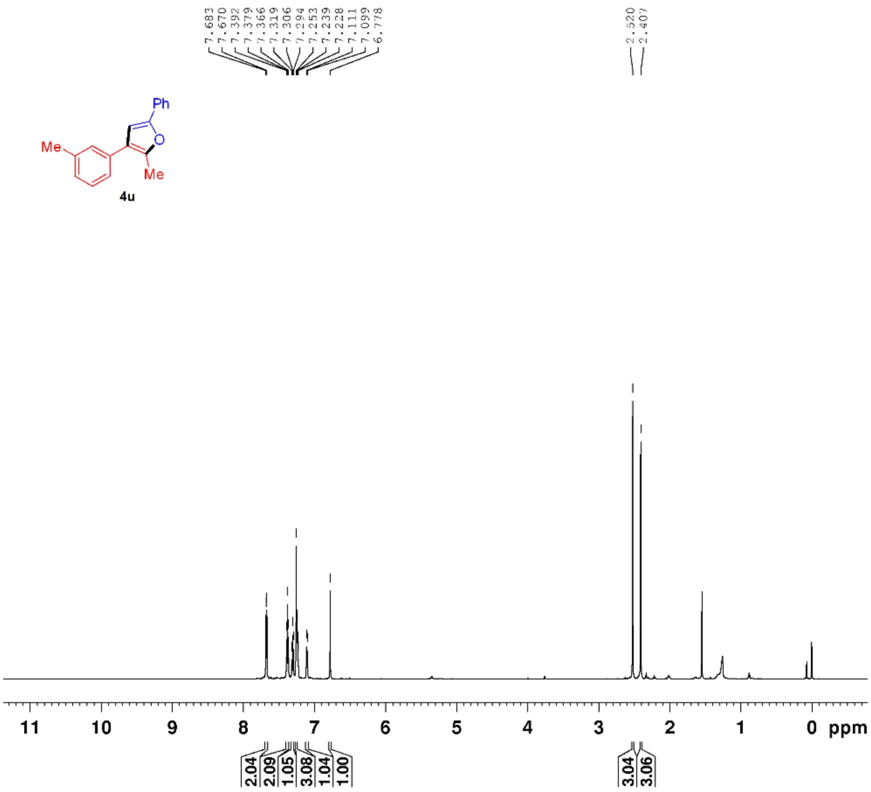

7.683
7.670
7.392
7.379
7.366
7.319
7.306
7.294
7.253
7.239
7.228
7.111
7.099
6.778
2.520
2.407
Ph
Me
O
Me
4u
11
10
9
8
7
6
5
4
3
2
1
0
ppm
2.04
2.09
1.05
3.08
1.04
1.00
3.04
3.06

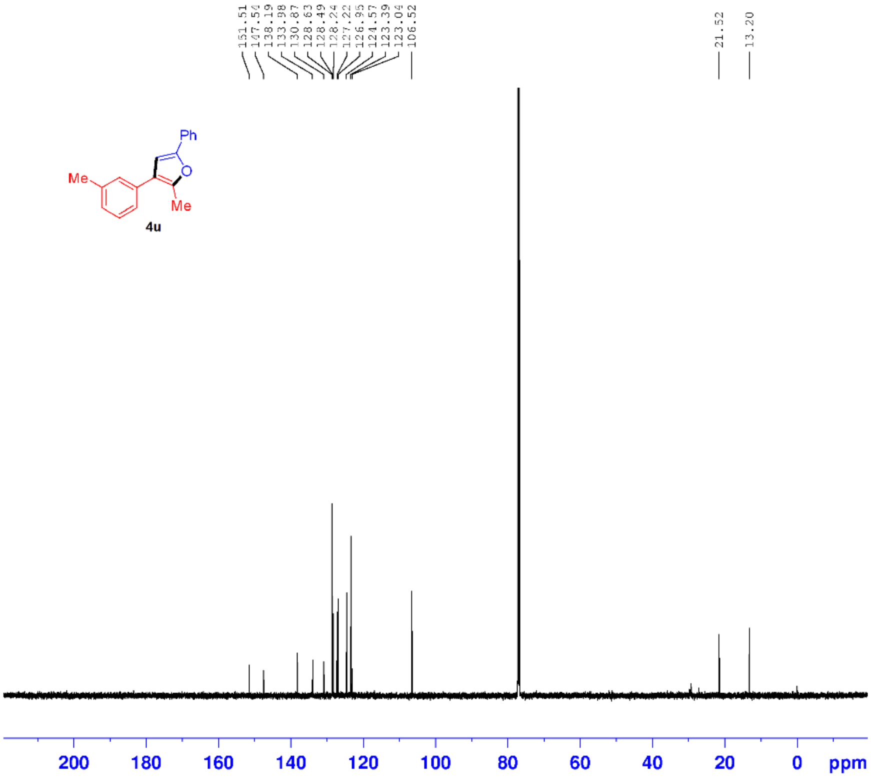
151.51
147.54
138.19
133.98
130.87
128.63
128.49
128.24
127.22
126.95
124.57
123.39
123.04
106.52
21.52
13.20
Ph
O
Me
Me
4u
200
180
160
140
120
100
80
60
40
20
0
ppm

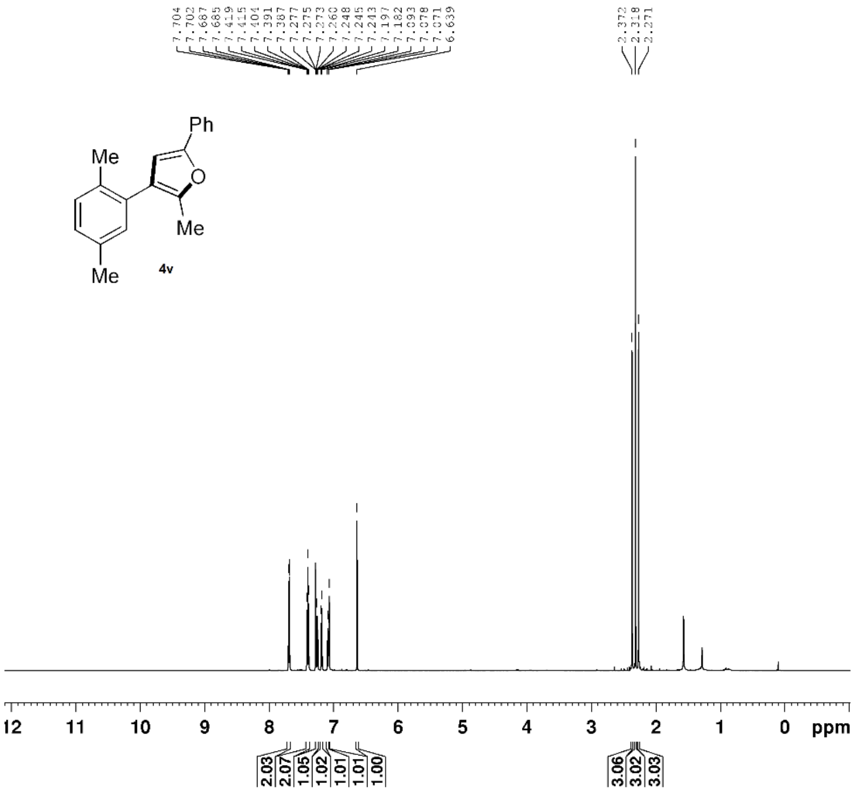
7.704
7.702
7.687
7.685
7.419
7.415
7.404
7.391
7.387
7.277
7.275
7.273
7.260
7.248
7.245
7.243
7.197
7.182
7.093
7.078
7.071
6.639
2.372
2.318
2.271
Ph
Me
O
Me
Me
4v
12
11
10
9
8
7
6
5
4
3
2
1
0
ppm
2.03
2.07
1.05
1.02
1.01
1.01
1.00
3.06
3.02
3.03

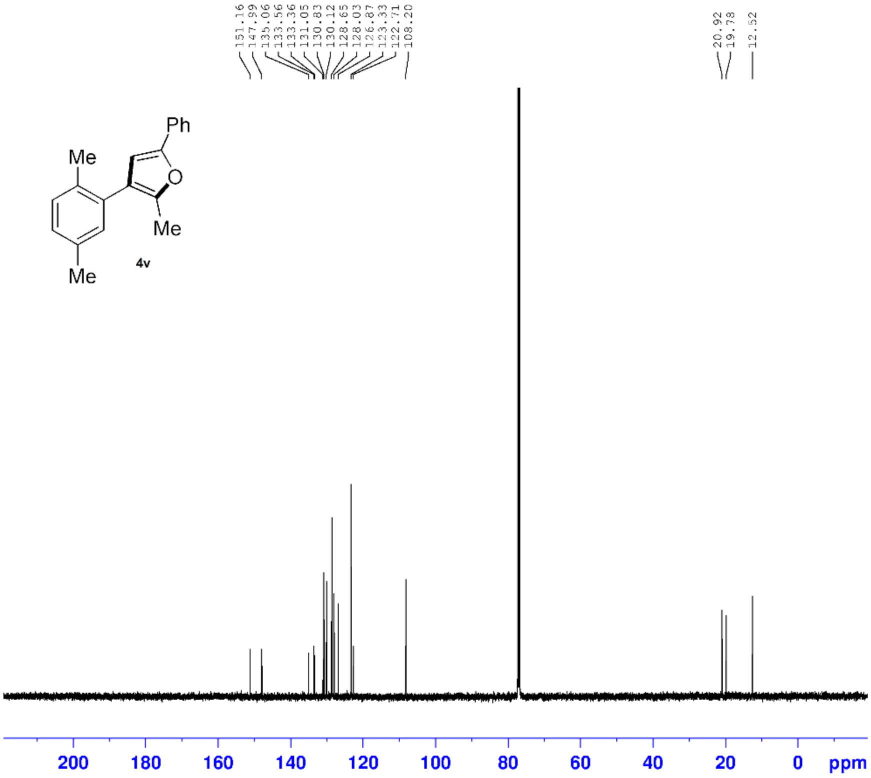
151.16
147.99
135.06
133.56
133.36
131.05
130.83
130.12
128.65
128.03
126.87
123.33
122.71
108.20
20.92
19.78
12.52
Ph
Me
O
Me
Me
4v
200
180
160
140
120
100
80
60
40
20
0
ppm

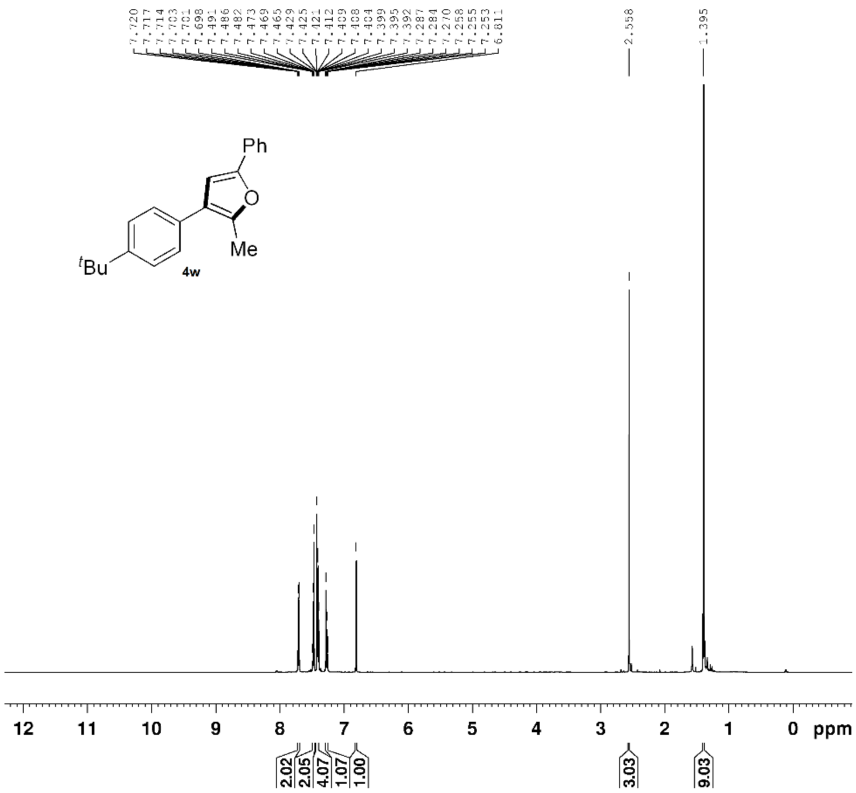

Ph
O
Me
tBu
4w
12 11 10 9 8 7 6 5 4 3 2 1 0 ppm
2.02
2.05
4.07
1.07
1.00
3.03
9.03
2.558
1.395

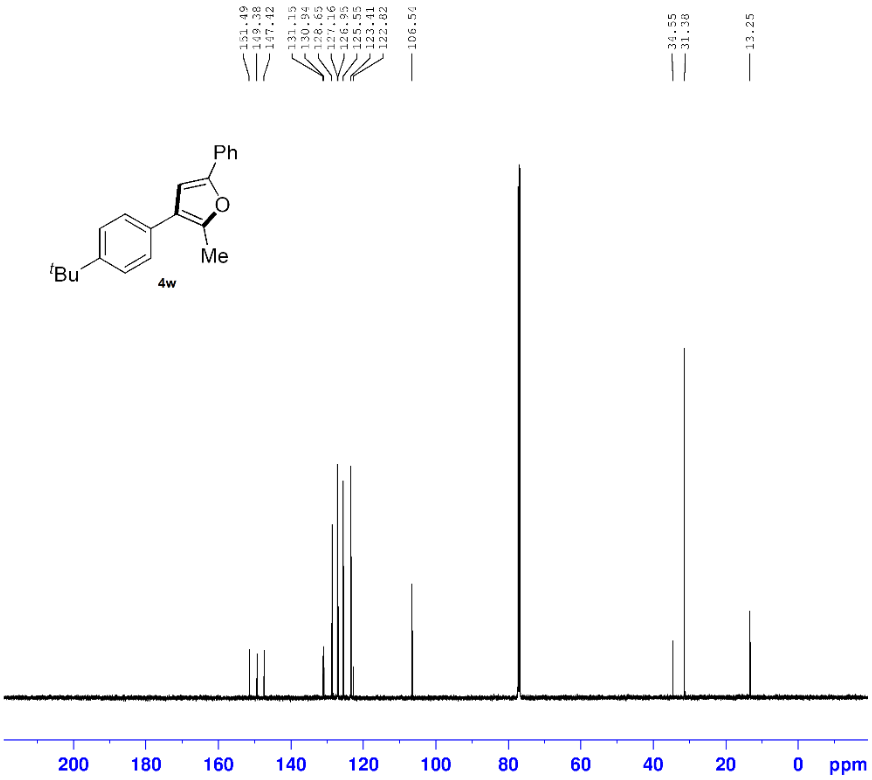
151.49
149.38
147.42
131.15
130.94
128.65
127.16
126.95
125.55
123.41
122.82
106.54
34.55
31.38
13.25
Ph
O
Me
tBu
4w
200
180
160
140
120
100
80
60
40
20
0
ppm

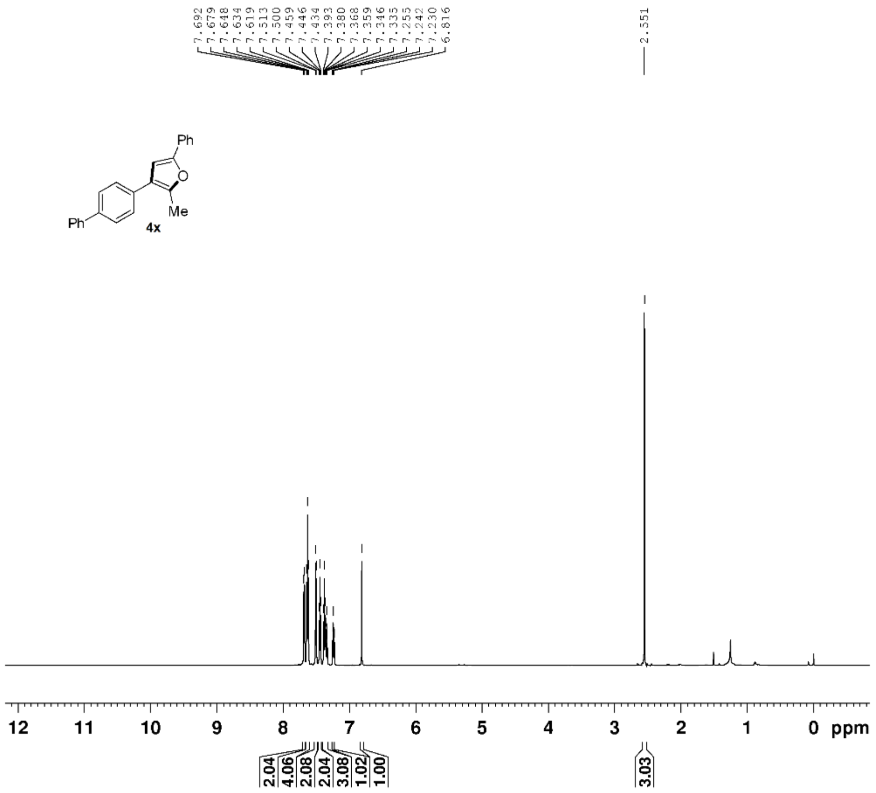

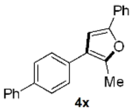
Ph
O
Me
Ph

4x

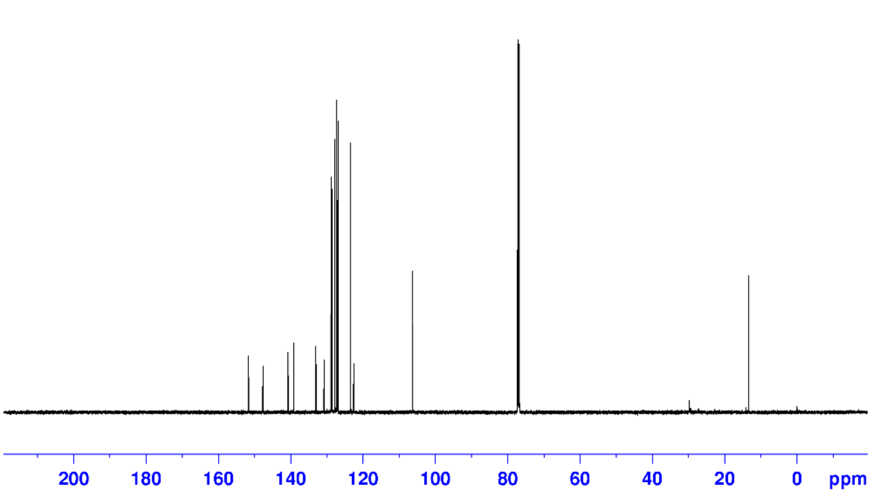
151.68
147.71
140.74
139.21
133.03
130.78
128.79
128.65
127.78
127.30
127.24
127.04
126.96
123.42
122.60
106.31
13.32
200
180
160
140
120
100
80
60
40
20
0
ppm

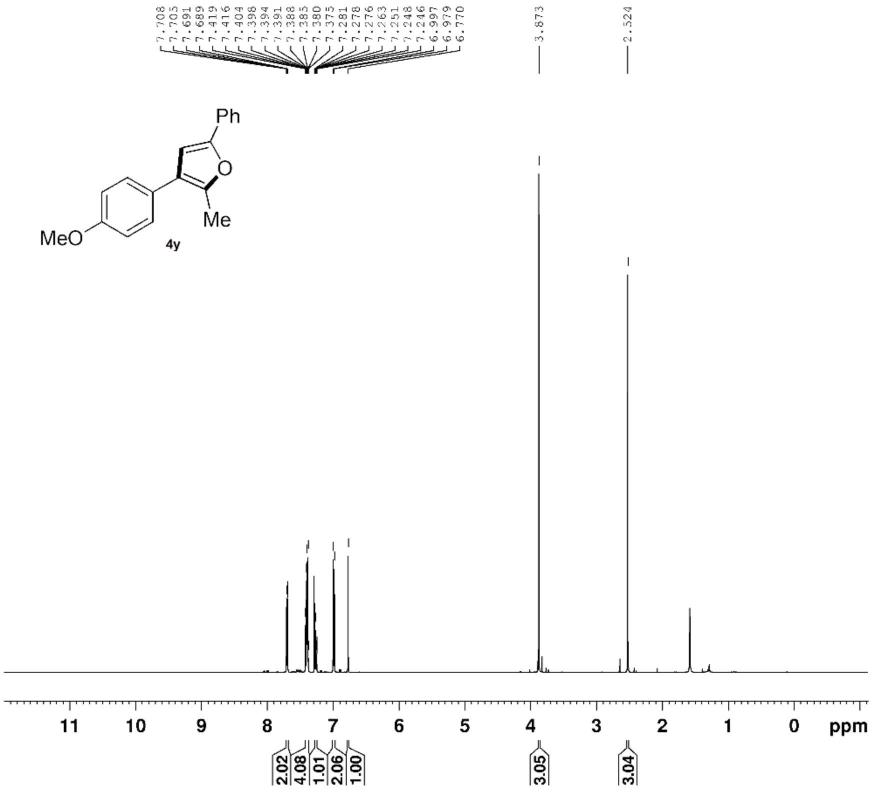
Ph
O
Me
MeO
4y
7.708
7.705
7.691
7.689
7.419
7.416
7.404
7.398
7.394
7.391
7.388
7.385
7.380
7.375
7.281
7.278
7.276
7.263
7.251
7.248
7.246
6.997
6.979
6.770
3.873
2.524
11
10
9
8
7
6
5
4
3
2
1
0
ppm
2.02
4.08
1.01
2.06
1.00
3.05
3.04

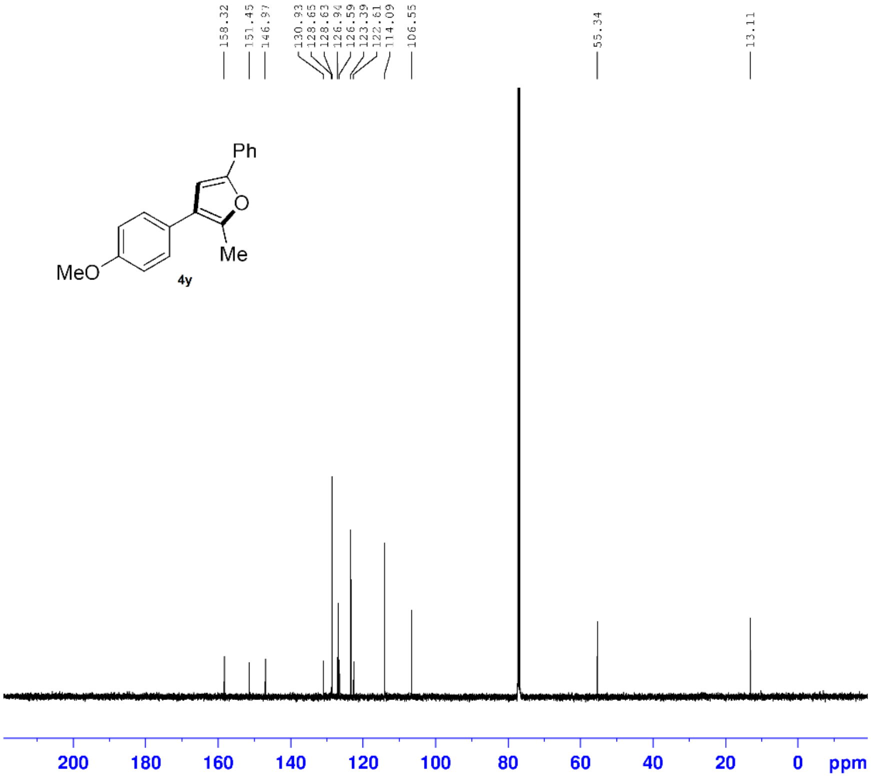
158.32
151.45
146.97
130.93
128.65
128.63
126.94
126.59
123.39
122.61
114.09
106.55
55.34
13.11
Ph
O
Me
MeO
4y
200
180
160
140
120
100
80
60
40
20
0
ppm

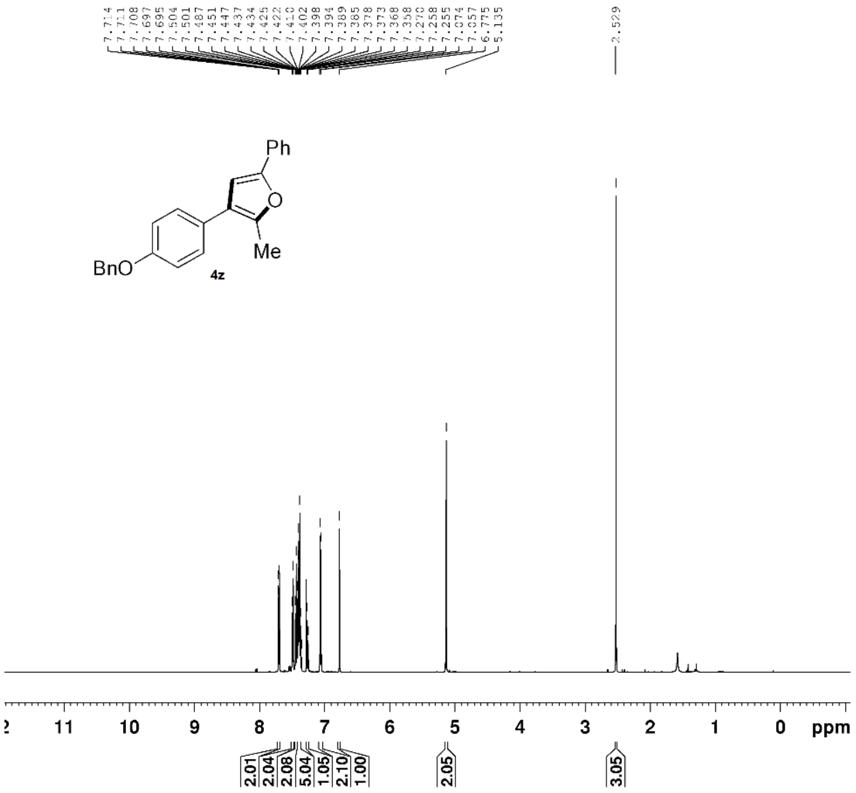

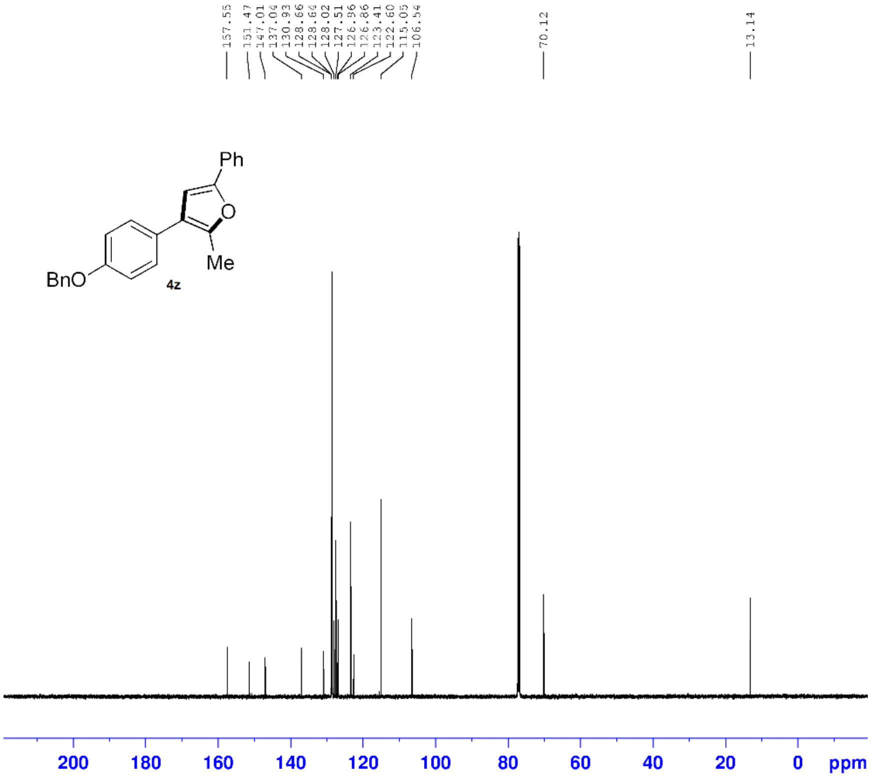
157.55
151.47
147.01
137.04
130.93
128.66
128.64
128.02
127.51
126.96
126.86
123.41
122.60
115.05
106.54
70.12
13.14
Ph
O
Me
BnO
4z
200
180
160
140
120
100
80
60
40
20
0
ppm

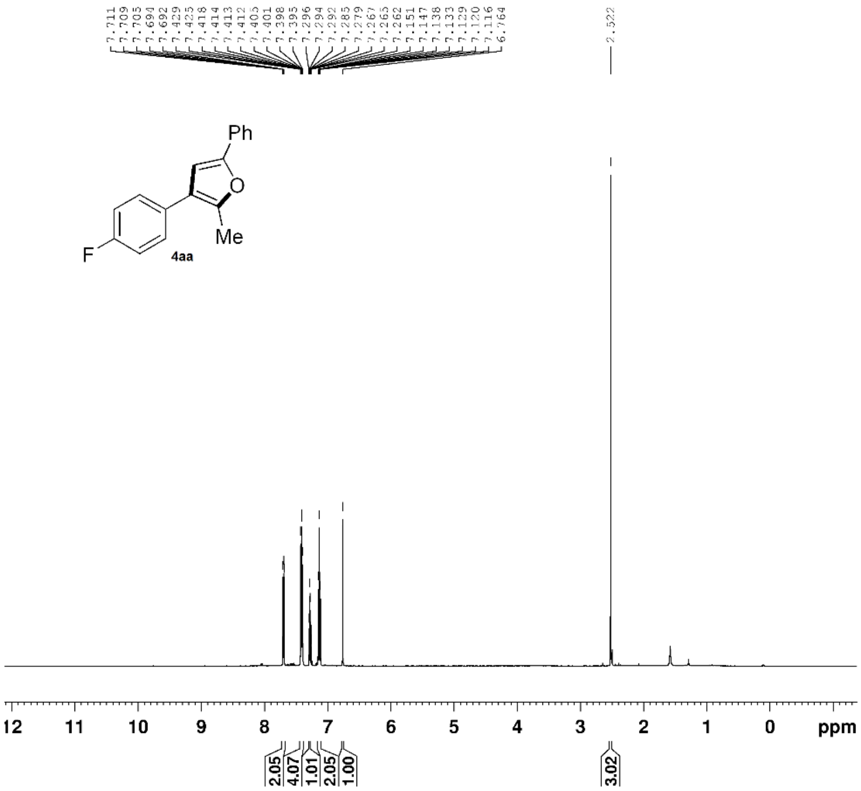
7.711
7.709
7.705
7.694
7.692
7.429
7.425
7.418
7.414
7.413
7.412
7.405
7.401
7.398
7.395
7.296
7.294
7.292
7.285
7.279
7.267
7.265
7.262
7.151
7.147
7.138
7.133
7.129
7.120
7.116
6.764
2.522
Ph
O
Me
F
4aa
12 11 10 9 8 7 6 5 4 3 2 1 0 ppm
2.05
4.07
1.01
2.05
1.00
3.02

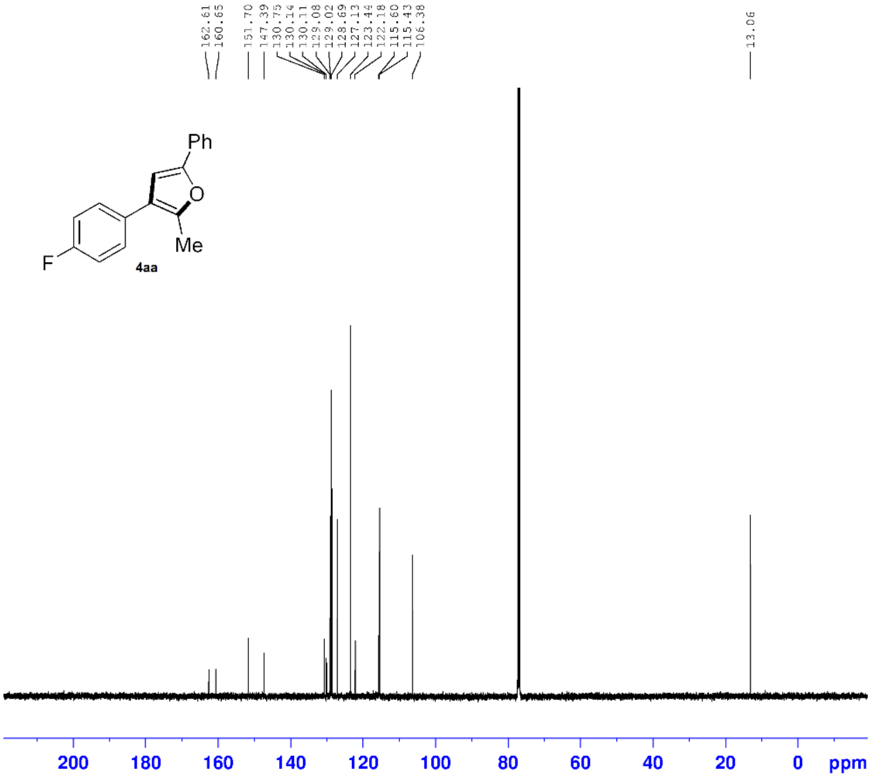
162.61
160.65
151.70
147.39
130.75
130.14
130.11
129.08
129.02
128.69
127.13
123.44
122.18
115.60
115.43
106.38
13.06
Ph
O
Me
F
4aa
200
180
160
140
120
100
80
60
40
20
0
ppm

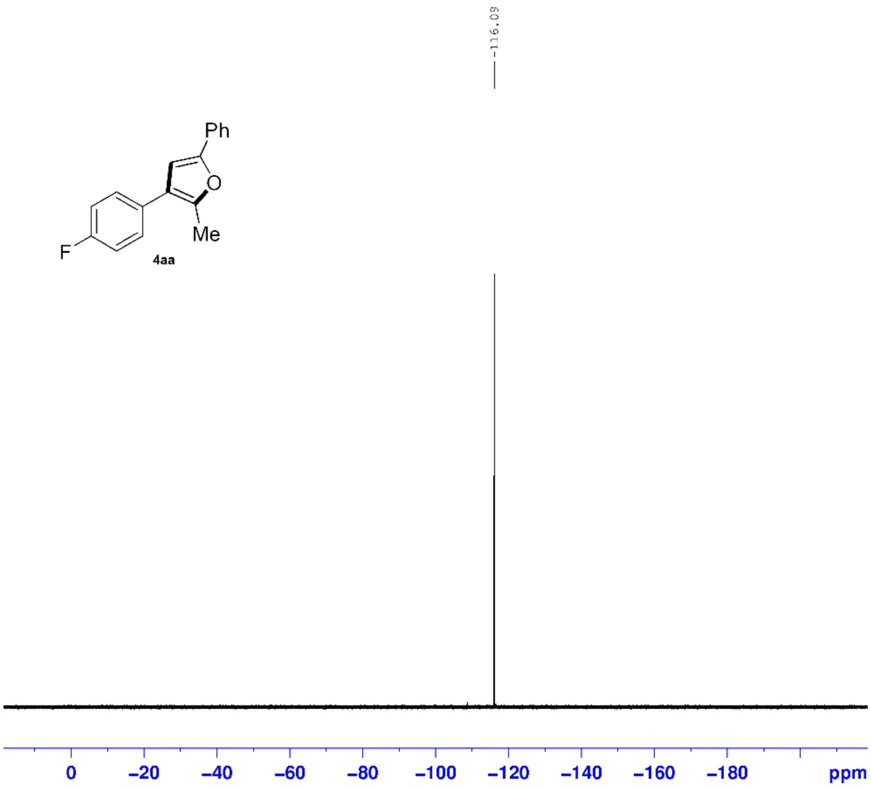
-116.03
Ph
O
Me
F
4aa
0
-20
-40
-60
-80
-100
-120
-140
-160
-180
ppm

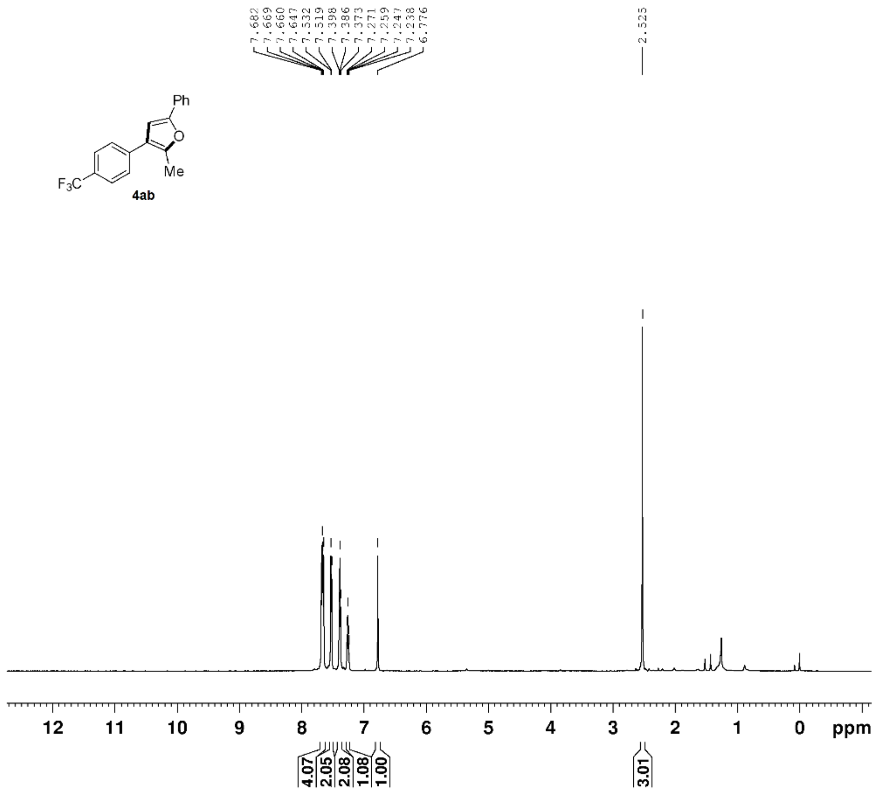

7.682
7.669
7.660
7.647
7.532
7.519
7.398
7.386
7.373
7.271
7.259
7.247
7.238
6.776
2.525
Ph
O
Me
F_3C
4ab
12
11
10
9
8
7
6
5
4
3
2
1
0
ppm
4.07
2.05
2.08
1.08
1.00
3.01

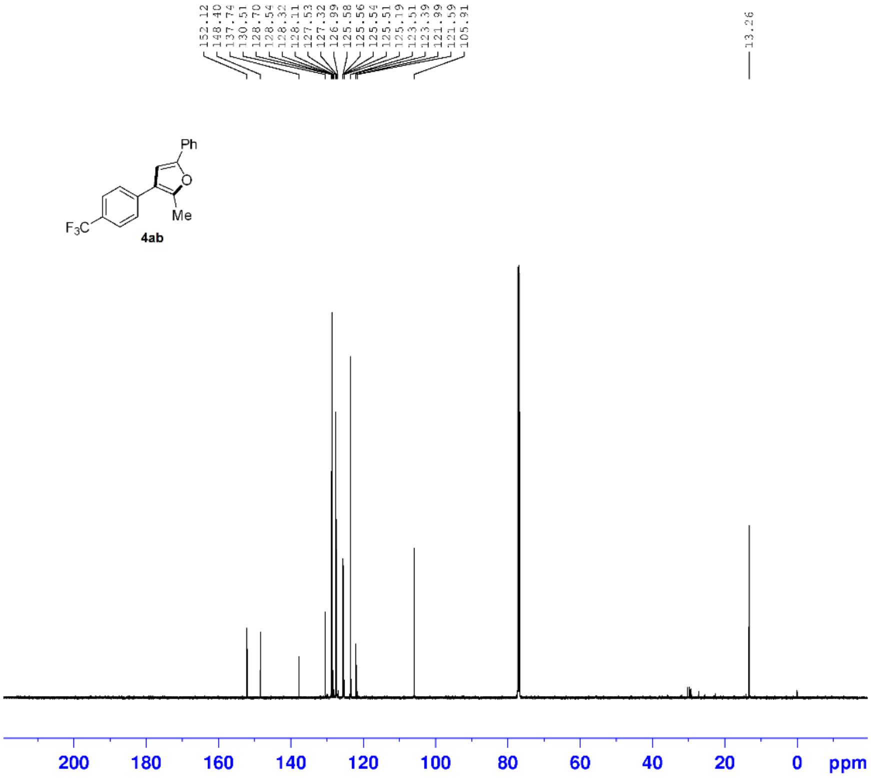
152.12
148.40
137.74
130.51
128.70
128.54
128.32
128.11
127.53
127.32
126.99
125.58
125.56
125.54
125.51
125.19
123.51
123.39
121.99
121.59
105.91
13.26
Ph
O
Me
F3C
4ab
200
180
160
140
120
100
80
60
40
20
0
ppm

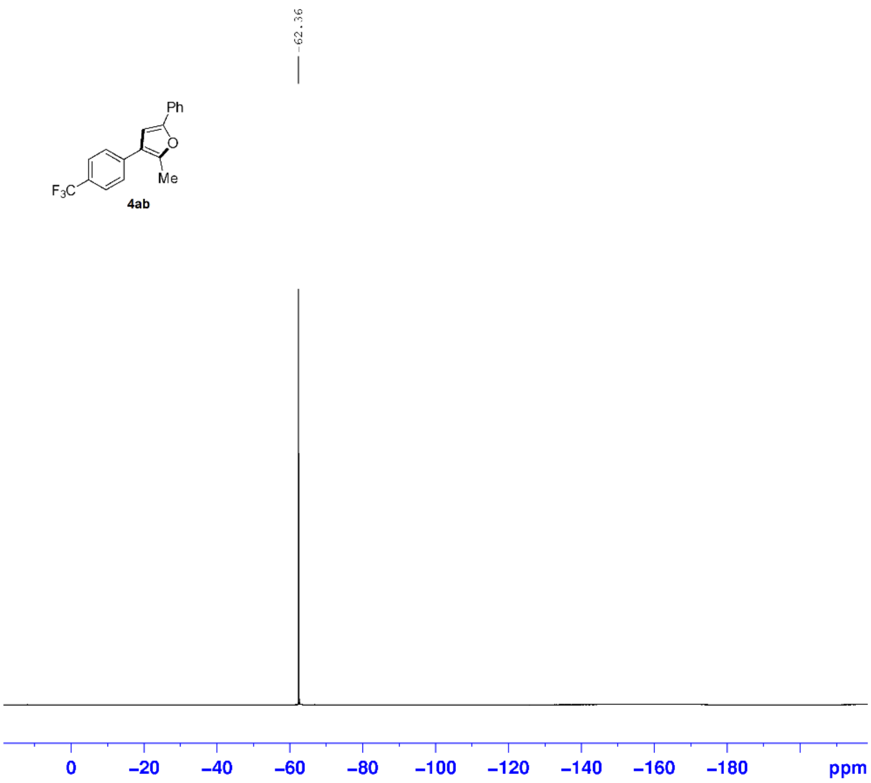
-62.36
Ph
O
Me
F3C
4ab
0
-20
-40
-60
-80
-100
-120
-140
-160
-180
ppm

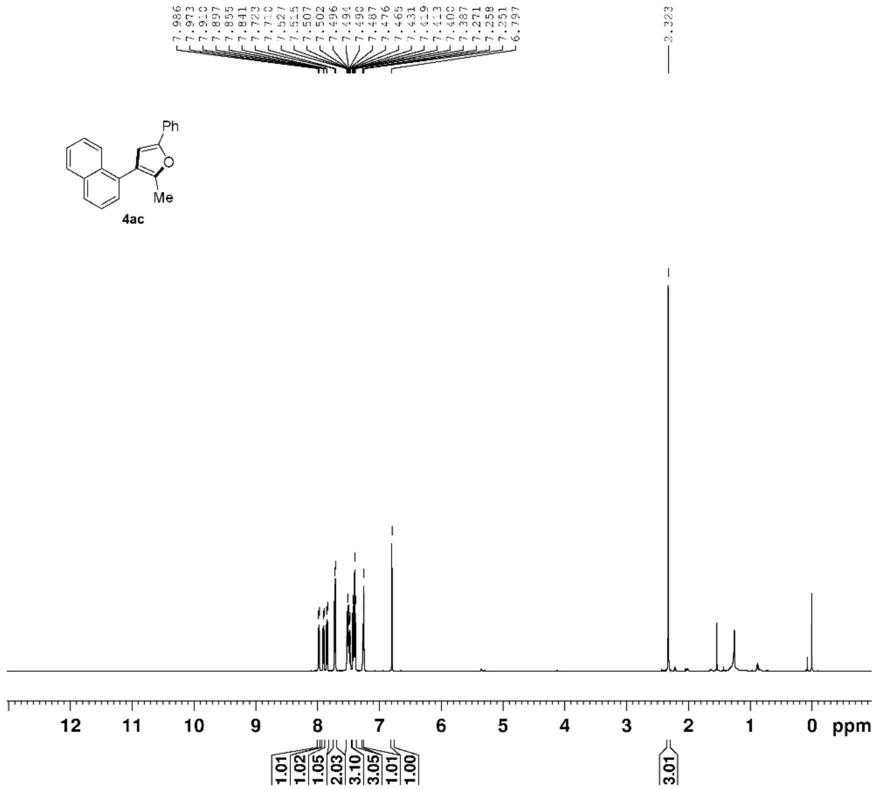

4ac

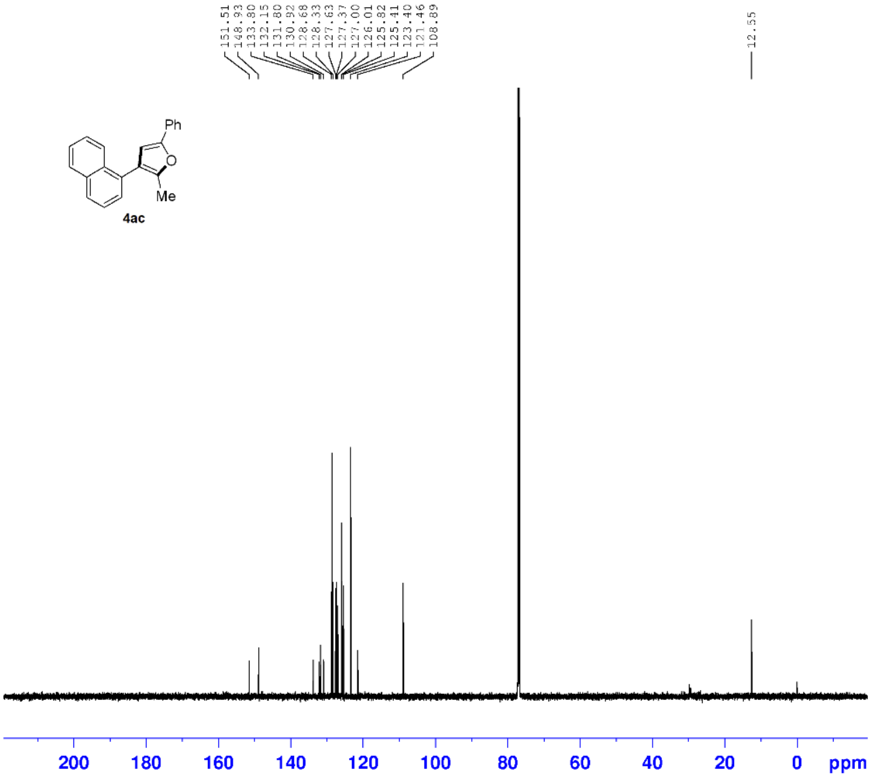
151.51
148.93
133.80
132.15
131.80
130.92
128.68
128.33
127.63
127.37
127.00
126.01
125.82
125.41
123.40
121.46
108.89
12.55
Ph
O
Me
4ac
200
180
160
140
120
100
80
60
40
20
0
ppm

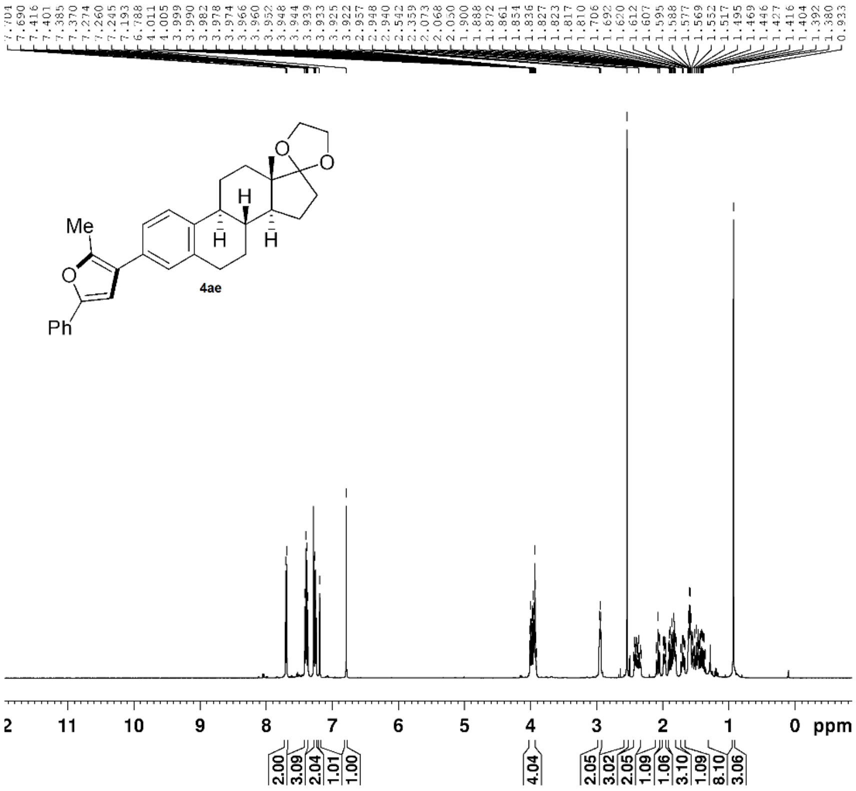

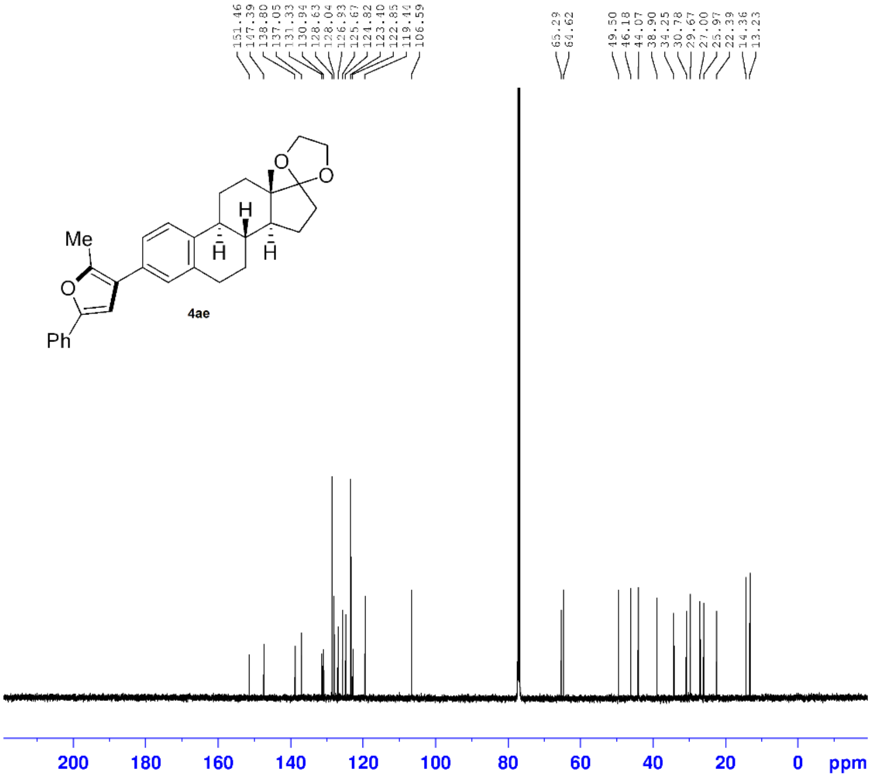
151.46
147.39
138.80
137.05
131.33
130.94
128.63
128.04
126.93
125.67
124.82
123.40
122.85
119.44
106.59
65.29
64.62
49.50
46.18
44.07
38.90
34.25
30.78
29.67
27.00
25.97
22.39
14.36
13.23
Me
O
Ph
H
O
O
4ae
200
180
160
140
120
100
80
60
40
20
0
ppm

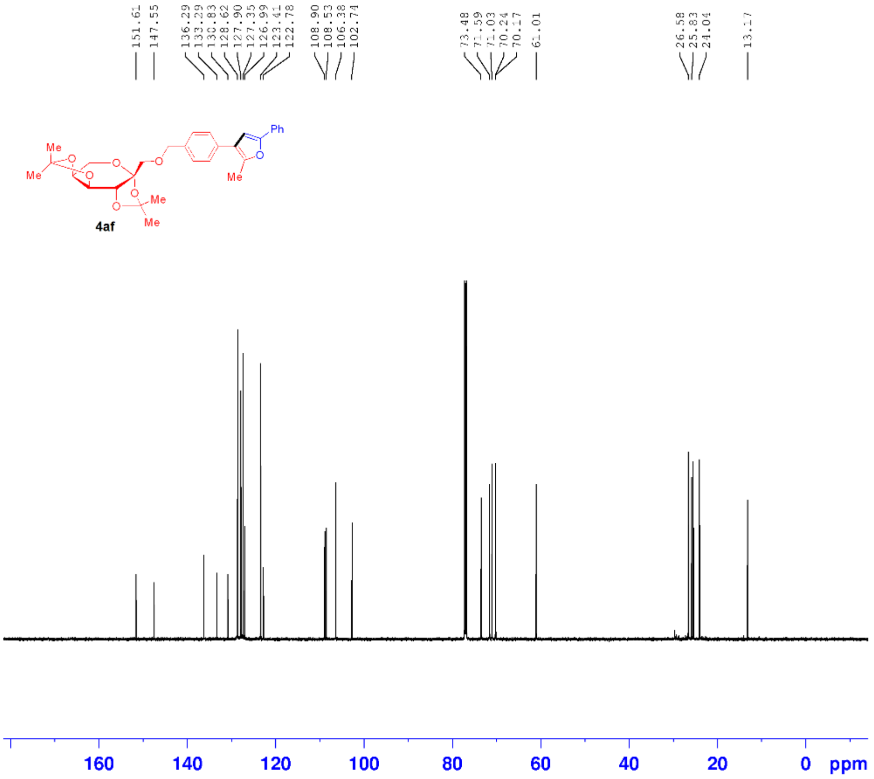
151.61
147.55
136.29
133.29
130.83
128.62
127.90
127.35
126.99
123.41
122.78
108.90
108.53
106.38
102.74
73.48
71.59
71.03
70.24
70.17
61.01
26.58
25.83
24.04
13.17
Ph
Me
Me
Me
Me
Me
4af
160
140
120
100
80
60
40
20
0
ppm

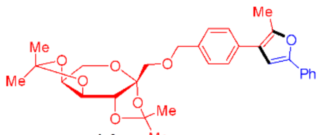

4af

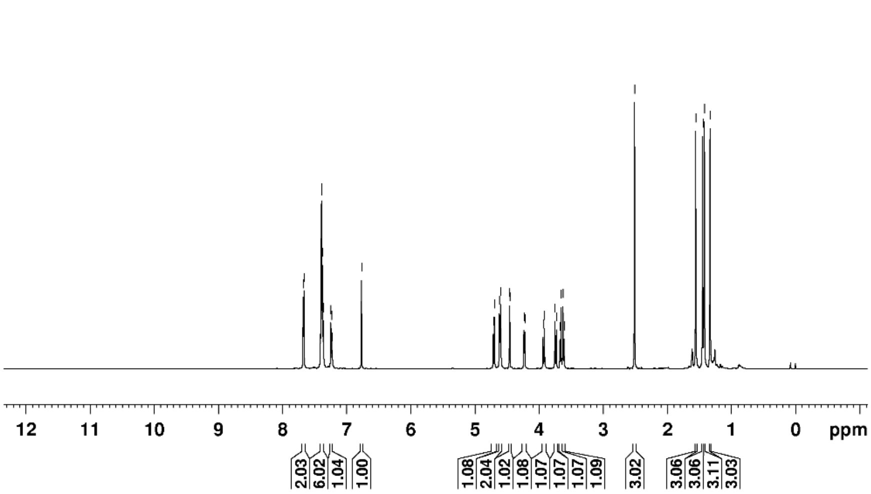

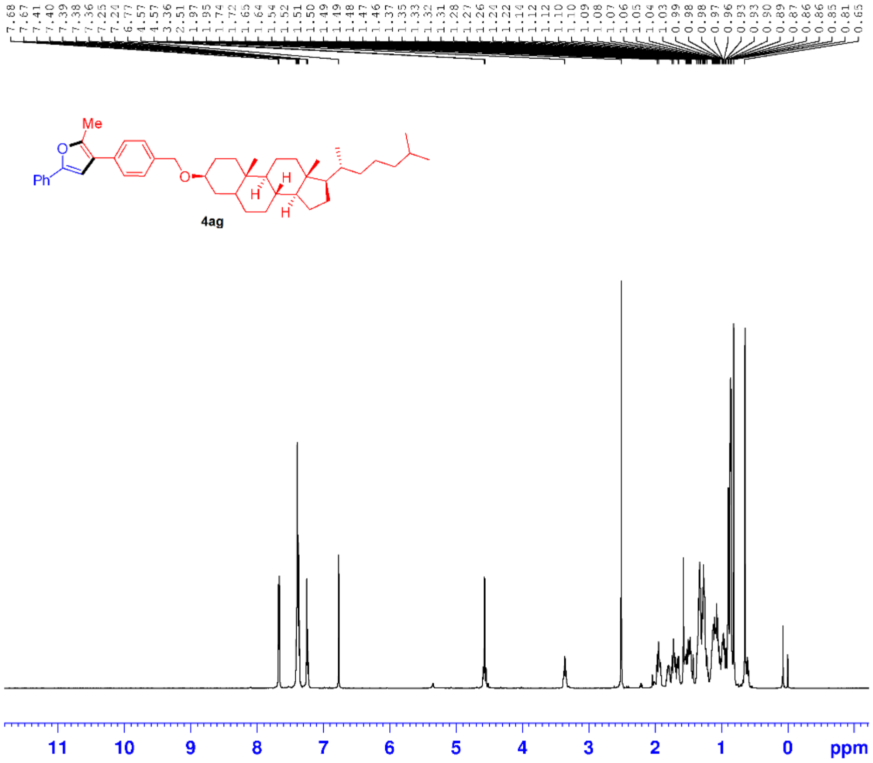

Me
O
Ph
4ag
H
H
H
11
10
9
8
7
6
5
4
3
2
1
0
ppm

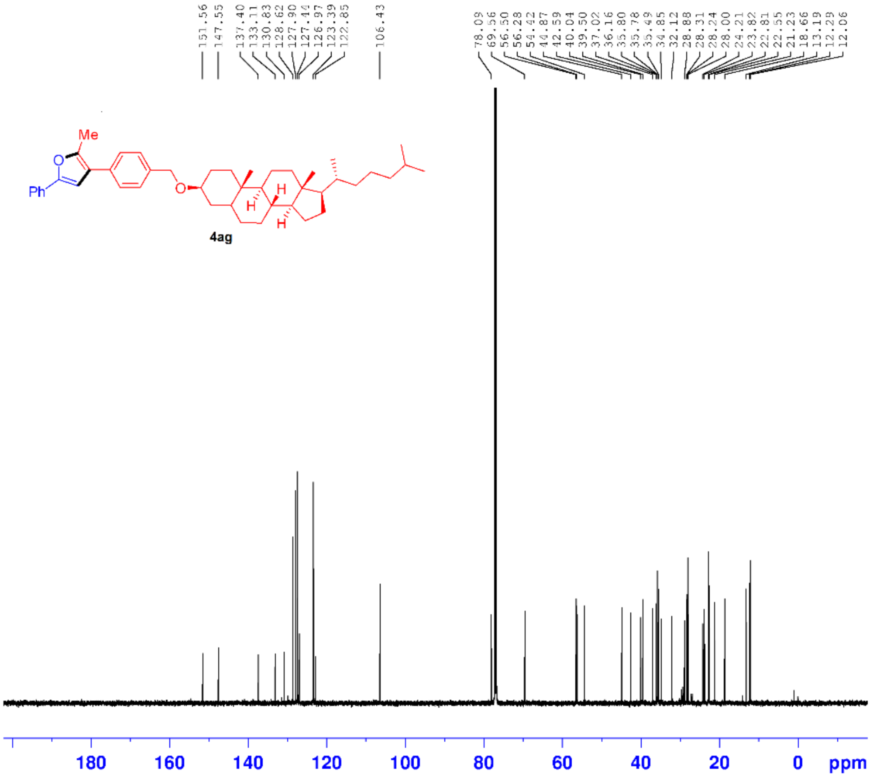

151.56
147.55
137.40
133.11
130.83
128.62
127.90
127.44
126.97
123.39
122.85
106.43
78.09
69.56
56.50
56.28
54.42
44.87
42.59
40.04
39.50
37.02
36.16
35.80
35.78
35.49
34.85
32.12
28.88
28.31
28.24
28.00
24.21
23.82
22.81
22.55
21.23
18.66
13.19
12.29
12.06
Me
O
Ph
O
H
H
H
4ag
180
160
140
120
100
80
60
40
20
0
ppm

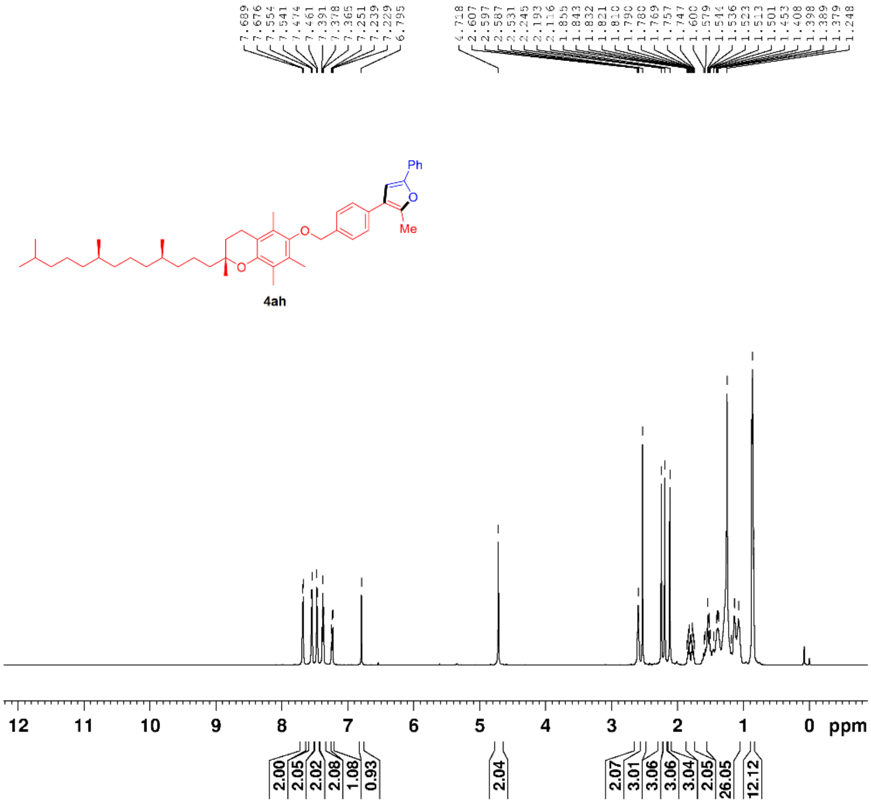

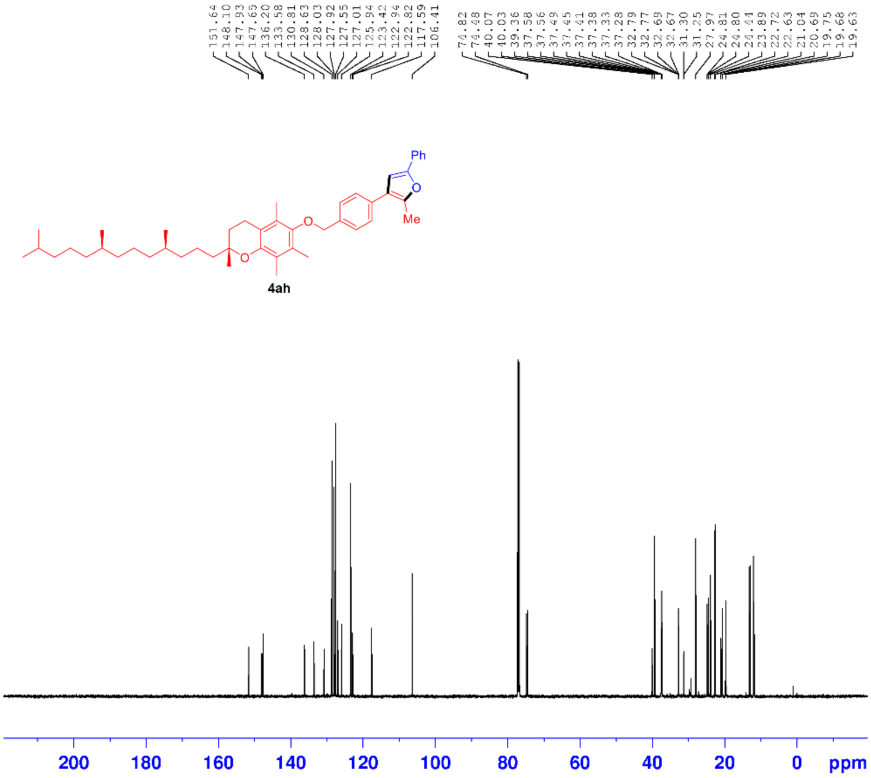

Supplement: SC-012-D1SC05451A-s001 [file SC-012-D1SC05451A-s001.pdf]
